# Supplementary material for: Arene C–H borylation strategy enabled by a non-classical boron cluster-based electrophile
Source: Nat Commun. 2023 Mar 25;14:1671. doi: 10.1038/s41467-023-37258-6 (PMC10039867; doi:10.1038/s41467-023-37258-6)
Supplement: Supplementary file 1 — Supplementary Information [file 41467_2023_37258_MOESM1_ESM.pdf]

Supplementary Information

Arene C–H Borylation Strategy Enabled by a Non-Classical Boron Cluster-Based Electrophile

Sangmin Kim,[1] Joseph W. Treacy,[1] Yessica A. Nelson,[1] Jordan A. M. Gonzalez,[1] Milan Gembicky,[2] K. N. Houk,[1] and Alexander M. Spokoyny[1,3,]*

[1]Department of Chemistry and Biochemistry, University of California, Los Angeles, Los Angeles, CA 90095, USA
[2]Department of Chemistry and Biochemistry, University of California, San Diego, La Jolla, CA 92093, USA
[3]California NanoSystems Institute, University of California, Los Angeles, Los Angeles, CA 90095, USA

spokoyny@chem.ucla.edu

Table of Contents

Supplementary Notes 2

Supplementary Methods 3

Supplementary Figures 13

Supplementary References 88

I. Supplementary Notes

All manipulations were carried out under aerobic conditions unless otherwise noted. All glassware was stored in a pre-heated oven prior to use. The solvents used for air- and moisture-sensitive manipulations (especially for electrochemical measurements) were dried and deoxygenated using literature procedures.[1] All arene substrates, triflic acid, ammonium cerium(IV) nitrate, diols, magnesium sulfate, cesium hydroxide hydrate, deuterated solvents and HPLC grade solvents were used as received without further purification unless otherwise noted. $[HNEt_3]_2[B_{10}H_{10}]$ was generously donated by Boron Specialties and used as received. The following compounds were prepared according to literature procedures: **1**[2] and **2**.[2]

^{1}H NMR, ^{13}C NMR, ^{11}B NMR and ^{19}F NMR spectra were recorded on Bruker AV400 spectrophotometers operating at 400 MHz, 101 MHz, 128 MHz and 376 MHz, respectively. All ^{1}H and ^{13}C NMR chemical shifts are reported in ppm relative to $SiMe_4$ using the ^{1}H (chloroform-*d*: 7.26 ppm; benzene-d_6: 7.16 ppm) and ^{13}C (chloroform-*d*: 77.16 ppm; benzene-d_6: 128.06 ppm) chemical shifts of the solvent as a standard.[3] ^{11}B NMR and ^{19}F chemical shifts are reported in ppm using an external BF_3•OEt_2 standard (0.0 ppm). ^{1}H NMR data for diamagnetic compounds are reported as follows: chemical shift, multiplicity (s = singlet, d = doublet, t = triplet, q = quartet, p = pentet, br = broad, m = multiplet, app = apparent, obsc = obscured), coupling constants (Hz), integration. ^{13}C NMR, ^{11}B NMR and ^{19}F NMR spectra are reported as follows: chemical shift. The carbon directly attached to the boron atom could not be detected by ^{13}C NMR spectroscopy due to the quadrupolar broadening.

High-resolution mass spectra were obtained at the UCLA Molecular Instrumentation Center (MIC) using a Waters LCT Premier TOF LC/MS.

All calculations were carried out with the Gaussian 16 software package.[4] Ground state geometries were optimized in *n*-hexane with the CPCM solvation model[5] using the ωB97X-D functional[6] and the 6-311+G(d,p) basis set for all atoms in the system. An ultrafine grid was applied to ensure calculation accuracy. Frequency calculations were carried out at the same level of theory to ensure that stationary points were truly minima or saddle points on the potential energy surface. Thermal corrections to free energies were calculated using Grimme's quasi-rigid rotor-harmonic oscillator approximation at 298 K[7] using the GoodVibes program.[8] Conformational searches were carried out using the CREST conformer-rotamer ensemble sampling tool,[9,10] version 2.7.1 with XTB version 6.2 RC2 (SAW190805).[11-13] Images of molecular structures were rendered in CYLview,[14] and the HOMO and LUMO were visualized using PyMOL.[15]

II. Supplementary Methods

Preparation of Cs_2[*closo*-$B_{10}H_{10}$] (1) from [$HNEt_3$]$_2$[$B_{10}H_{10}$]. In a typical experiment, a 20 mL scintillation vial was charged with a magnetic stir bar, 3.22 g (10 mmol) of [$HNEt_3$]$_2$[$B_{10}H_{10}$] and 5 mL distilled H_2O under N_2 or aerobic conditions. 3.36 g (20 mmol) of CsOH•H_2O was dissolved in a minimum amount of distilled H_2O, and the aqueous CsOH solution was slowly added to the solution of [$HNEt_3$]$_2$[$B_{10}H_{10}$] with vigorous stirring. After 24 h, precipitates from the aqueous solution were filtered using a glass frit, and the collected solid on the glass frit was washed with 2 x 10 mL of EtOH and 3 x 10 mL of Et_2O then dried under vacuum to give 3.2 g (8.3 mmol, 83%) of a white solid identified as **1**.

Preparation of 6-Tol-*nido*-$B_{10}H_{13}$ (2) from 1. In a typical experiment, a 20 mL scintillation vial was charged with a magnetic stir bar, 770 mg (2.0 mmol) of **1** and 5 mL toluene under aerobic conditions. Then, 0.90 mL of HOTf (10.0 mmol, 5.0 equiv) was added to the reaction mixture and the mixture was stirred for 3 h at 23 ºC. After 3 h, the volatiles were removed under vacuum and the resulting residue was purified by silica gel flash column chromatography using hexane as an eluent. Fractions were collected and the volatiles were removed under vacuum to give 378 mg (1.78 mmol, 89%) of a white solid identified as **2**.

Preparation of [NBu_4]$_2$[*closo*-$B_{10}H_{10}$] (41) from [$HNEt_3$]$_2$[$B_{10}H_{10}$]. In a typical experiment, a 20 mL scintillation vial was charged with a magnetic stir bar, 1.65 g (7.55 mmol) of [$HNEt_3$]$_2$[$B_{10}H_{10}$] and 10 mL distilled H_2O under aerobic conditions. 8.9 g (18.9 mmol, 2.5 equiv) of 55% aq. [NBu_4]OH solution was slowly added to a solution of [$HNEt_3$]$_2$[$B_{10}H_{10}$] with vigorous stirring. After 24 h, precipitates from the aqueous solution were filtered using a glass frit, and the collected solid on the glass frit was recrystallized from a hot EtOH solution. The resulting solids from the recrystallization were collected on the glass frit again, washed with Et_2O, and then dried under vacuum to give 2.2 g (3.65 mmol, 48%) of a white solid identified as **41**.

Optimization of cage-opening of 1 to generate 6-Ph-*nido*-$B_{10}H_{13}$. In a typical experiment, a 4-mL dram vial was charged with a magnetic stir bar, **1**, benzene and solvent under N_2 or aerobic conditions. Then, HOTf was added to the reaction mixture, and the mixture was stirred for 3 h at 23 ºC. After 3 h, the crude reaction mixture was filtered through a silica plug prepared using a Pasteur pipette packed with ~2 cm length of silica gel. The filter cake was then washed with 10 mL hexanes, and the filtrate was collected. Volatiles of the filtrate were removed under vacuum, and the resulting crude mixture was dissolved in $CDCl_3$ with 10 µL of mesitylene as an internal standard. The $CDCl_3$ solution was analyzed by ^{1}H NMR spectroscopy to obtain NMR spectroscopic yields of 6-Ph-*nido*-$B_{10}H_{13}$.

Supplementary Table 1. Screening of reaction conditions of the cage-opening of **1** to generate 6-Ph-*nido*-$B_{10}H_{13}$.

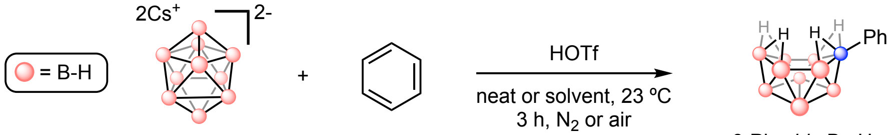

1

6-Ph-*nido*-$B_{10}H_{13}$
analyzed by ^{1}H NMR

Entry	**1** (equiv)	**1** (mmol)	Benzene (equiv)	Benzene (mmol)	Solvent (0.5 mL)	HOTf (equiv)	NMR yield (%)	Atm. (1 atm)
1	1	0.4	1	0.4	Hexanes	3	5	N_2
2	1	0.4	14	5.6	neat	5	***72***	N_2
3	1	0.4	1	0.4	Et_2O	5	0	N_2
4	1	0.4	1	0.4	Pentane	5	15	N_2
5	1	0.4	1	0.4	Hexanes	5	15	N_2
6	1	0.4	1	0.4	*n*-C_6F_{14}	5	11	N_2
7	1	0.4	1	0.4	*c*-C_6H_{12}	5	21	N_2
8	1	0.4	1	0.4	neat	5	8	N_2

9	2	0.8	1	0.4	c-C_6H_{12}	10	22	N_2
10	2	0.8	1	0.4	c-C_6H_{12}	10	22	N_2
11	1	0.4	14	5.6	neat	3	23	N_2
12	1	0.4	2	0.8	Hexanes	5	32	air
13	1	0.4	5	2	Hexanes	5	***56***	air
14	1	0.4	5	2	c-C_6H_{12}	5	57	air

The highest yield of 6-Ph-*nido*-$B_{10}H_{13}$ was obtained under neat conditions (Table 1, entry 2). For this reason, liquid substrates were used under neat conditions without additional solvents for the cage-opening reaction. Among conditions testing various solvents, 0.5 mL of hexanes or cyclohexane with 5 equiv of benzene afforded the highest yields of 6-Ph-*nido*-$B_{10}H_{13}$ (Table 1, entry 13). Therefore, 0.5 mL of hexanes was used as the solvent when solid substrates were examined during this study. Additionally, N_2 and aerobic conditions did not show significant differences in the yields, so aerobic conditions were adopted for further studies after the optimization. The major side products observed in the solvent conditions were 6-R-*nido*-$B_{10}H_{13}$ (R = alkyl from solvents, OTf or H) compounds.

Optimization of synthesis of PhBpin (4) from 1 by the sequential cage-opening and deconstruction steps. In a typical experiment, a 4-mL dram vial was charged with a magnetic stir bar, 154 mg of **1** and 0.5 mL of benzene under aerobic conditions. Then, 180 µL of HOTf was added to the reaction mixture, and the mixture was stirred for 3 h at 23 ºC. After 3 h, the crude reaction mixture was filtered through a silica plug prepared using a Pasteur pipette packed with ~2 cm length of silica gel. The filter cake was then washed with 10 mL hexanes and the filtrate was collected. Volatiles of the filtrate were removed under vacuum and the resulting crude mixture was dissolved in 10 mL MeCN. Then, a 25 mL Schlenk flask was charged with a magnetic stir bar, additive (CAN or *p*-BQ), pinacol, $MgSO_4$ and the crude MeCN solution under aerobic conditions. The reaction mixture was heated at 65 ºC and stirred for 24 h. The reaction mixture was then cooled down to room temperature, and an aliquot of 1 mL of the crude mixture was taken and transferred to a 20 mL scintillation vial. Volatiles were removed under vacuum using a rotovap, and the resulting residue was dissolved in CD_3Cl along with 10 µL of mesitylene as an internal standard. The CD_3Cl solution was analyzed by ^1H NMR spectroscopy.

Supplementary Table 2. Screening of reaction conditions to make **4** from **1** *via* two sequential steps.

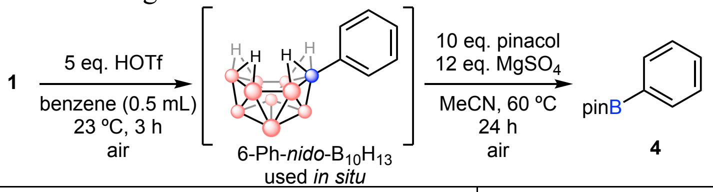

Entry	Deviations from conditions above	NMR yield of **4** (remaining 6-Ph-*nido*-$B_{10}H_{13}$)
1	none	24 (40)
2	10 mol% *p*-BQ	22 (38)
3	10 mol% CAN	48 (9)
4	20 mol% CAN	**71 (n.d.)**
5	20 mol% CAN and an N_2 atmosphere	63 (n.d.)
6	50 mol% CAN	45 (14)
7	6 equiv. *p*-BQ	37 (2)
8	20 mol% CAN and 2 equiv. pinacol	31 (n.d.)

p-BQ = 1,4-benzoquinone; CAN = cerium ammonium nitrate; n.d. = not detected.

Caution: (1) Triflic acid is a superacid and thus is very reactive, so special caution is required when it is added to any potentially reactive or flammable chemicals, for example, nitroarenes and so on. (2) The parent unfunctionalized decaborane, nido-$B_{10}H_{14}$, is known to be neurotoxic.

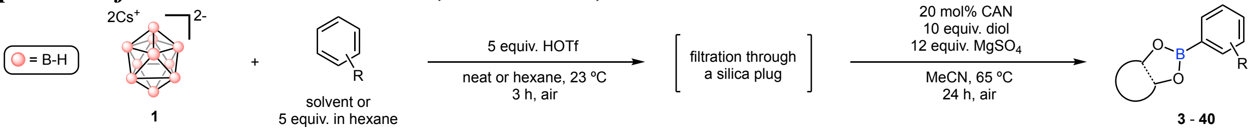

General Procedure for Borylation of Alkyl- and Halobenzene. In a typical experiment, a 4-mL dram vial was charged with a magnetic stir bar, 154 mg of **1** (0.40 mmol, 1.0 equiv), and 0.5 mL liquid substrates or 5.0 equiv of solid substrates (2.0 mmol) in 0.5 mL hexanes under aerobic conditions. Then, 180 μL of HOTf (2.0 mmol, 5.0 equiv) was added to the reaction mixture and the mixture was stirred for 3 h at 23 ºC. The reaction mixture was filtered through a silica plug prepared using a Pasteur pipette packed with ~2 cm length of silica gel. After the filtration, the filter cake was washed with 10 mL hexanes, and the filtrate was collected. Volatiles of the filtrate were removed under vacuum, and the resulting crude mixture was transferred to a 25 mL Schlenk flask charged with a magnetic stir bar, 44 mg of CAN (20 mol%, 0.04 mmol), 4.0 mmol of diol, 580 mg of magnesium sulfate (4.8 mmol, 12.0 equiv) and 10 mL MeCN. The reaction mixture was stirred at 65 ºC for 24 h unless otherwise noted. After 24 h, the reaction mixture was filtered through a pad of Celite, and the filtrate was dried under vacuum to remove volatiles. The crude mixture was purified by silica gel flash column chromatography using hexanes/EtOAc as eluents. Finally, the collected eluents were concentrated under vacuum to give the desired aryl boronic ester as the product, which was analyzed by NMR spectroscopy.

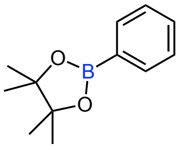

4

4,4,5,5-tetramethyl-2-phenyl-1,3,2-dioxaborolane, 4. 55 mg (0.270 mmol, 67%) of a colorless oil was obtained by flash column chromatography using an eluent (Hex:EtOAc = 25:1). ^{1}H NMR (400 MHz, $CDCl_3$, 23 ºC): δ 7.86 – 7.78 (m, 2H), 7.52 – 7.43 (m, 1H), 7.43 – 7.32 (m, 2H), 1.36 (s, 12H). ^{13}C NMR (101 MHz, $CDCl_3$) δ 134.88, 131.39, 127.85, 83.90, 25.01. The spectral data match with the previously reported data.[16]

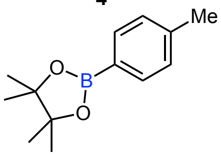

3

4,4,5,5-tetramethyl-2-(4-methylphenyl)-1,3,2-dioxaborolane, 3. 72 mg (0.33 mmol, 83%) of a colorless oil was obtained by flash column chromatography using an eluent (Hex:EtOAc = 25:1). ^{1}H NMR (400 MHz, $CDCl_3$) (for the major *para*-isomer) δ 7.71 (d, J = 8.1 Hz, 2H), 7.19 (d, J = 8.1 Hz, 2H), 2.37 (s, 3H), 1.34 (s, 12H). (for the minor *meta*-isomer) δ 7.64 (s, 1H), 7.63 – 7.59 (m, 1H), 7.28 (d. J = 1.3 Hz, 1H), 7.28 – 7.26 (m, 1H), 2.36 (s, 3H), 1.35 (s, 12H). ^{13}C NMR (101 MHz, $CDCl_3$) (for the major *para*-isomer) δ 141.54, 134.95, 128.66, 83.75, 25.00, 21.87. The spectral data match with the previously reported data.[16]

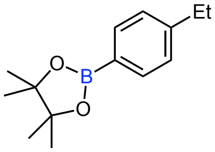

5

2-(4-ethylphenyl)-4,4,5,5-tetramethyl-1,3,2-dioxaborolane, 5. 52 mg (0.224 mmol, 56%) of a colorless oil was obtained by flash column chromatography using 100% Hex then Hex/EtOAc (25:1, R_f = 0.28) as eluents. ^{1}H NMR (400 MHz, $CDCl_3$, 23 ºC): (for the major *para*-isomer) δ 7.74 (d, J = 8.1 Hz, 2H), 7.21 (d, J = 8.1 Hz, 2H), 2.66 (q, J = 7.6 Hz, 2H), 1.34 (s, 12H), 1.24 (t, J = 7.6 Hz, 3H). (for the minor *meta*-isomer) δ 7.65 (s, 1H), 7.65 – 7.61 (m, 1H), 7.31 – 7.28 (m, 2H), 2.66 (q, J = 7.6 Hz, 2H), 1.35 (s, 12H), 1.24 (t, J = 7.6 Hz, 3H). ^{13}C NMR (101 MHz, $CDCl_3$, 23 ºC): (for the major *para*-isomer) δ 135.05, 127.49, 83.76, 29.25, 25.00, 15.61. The spectral data match with the previously reported data.[16]

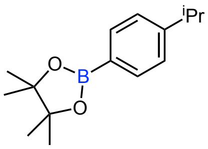

6

2-(4-isopropylphenyl)-4,4,5,5-tetramethyl-1,3,2-dioxaborolane, 6. 43 mg (0.175 mmol, 44%) of a colorless oil was obtained by flash column chromatography using 100% Hex then Hex/EtOAc (25:1, R_f = 0.31) as eluents. ^{1}H NMR (400 MHz, $CDCl_3$, 23 ºC): (for the major *para*-isomer) δ 7.76 (d, J = 8.2 Hz, 2H), 7.25 (d, J = 8.2 Hz, 2H), 2.92 (hept, J = 7.0 Hz, 1H), 1.34 (s, 12H), 1.26 (d, J = 7.0 Hz, 6H). (for the minor *meta*-isomer) δ 7.68 (s, 1H), 7.65 (dt, J = 6.6, 1.5 Hz, 1H), 7.33 (m, 2H), 2.91 (hept, J = 7.0 Hz, 1H), 1.36 (s, 12H), 1.27 (d, J = 6.8 Hz, 6H). (for the minor *ortho*-isomer, identifiable peaks only) δ 7.42 – 7.35 (m, 1H), 7.20 – 7.14 (m, 1H), 1.35 (s, 12H). ^{13}C NMR (101 MHz, $CDCl_3$, 23 ºC): (for the major

para-isomer) δ 152.45, 135.08, 126.05, 83.74, 34.49, 24.99, 23.98. The spectral data match with the previously reported data.[17]

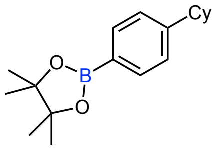

7

2-(4-cyclohexylphenyl)-4,4,5,5-tetramethyl-1,3,2-dioxaborolane, 7. 74 mg (0.259 mmol, 65%) of a colorless solid was obtained by flash column chromatography using 100% Hex then Hex/EtOAc (25:1, R_f = 0.23) as eluents. ^{1}H NMR (400 MHz, $CDCl_3$, 23 ºC): (for the major *para*-isomer) δ 7.75 (d, *J* = 8.2 Hz, 2H), 7.23 (d, *J* = 8.2 Hz, 2H), 2.57 – 2.46 (m, 1H), 1.91 – 1.79 (m, 4H), 1.79 – 1.70 (m, 1H), 1.50 – 1.36 (m, 4H), 1.33 (s, 12H), 1.31 – 1.23 (m, 1H). (for the minor *meta*-isomer) δ 7.67 – 7.61 (m, 2H), 7.32 – 7.29 (m, 2H), 2.57 – 2.46 (m, 1H), 1.91 – 1.79 (m, 4H), 1.79 – 1.70 (m, 1H), 1.50 – 1.36 (m, 4H), 1.35 (s, 12H), 1.31 – 1.23 (m, 1H). ^{13}C NMR (101 MHz, $CDCl_3$, 23 ºC): (for the major *para*-isomer) δ 151.64, 135.04, 126.48, 83.74, 45.00, 34.40, 27.01, 26.29, 24.99. The spectral data match with the previously reported data.[18]

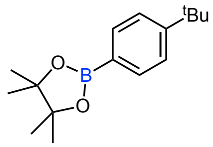

8

2-(4-(*tert*-butyl)phenyl)-4,4,5,5-tetramethyl-1,3,2-dioxaborolane, 8. 61 mg (0.234 mmol, 59%) of a colorless solid was obtained by flash column chromatography using 100% Hex then Hex/EtOAc (25:1, R_f = 0.25) as eluents. ^{1}H NMR (400 MHz, $CDCl_3$, 23 ºC): (for the major *para*-isomer) δ 7.79 (d, *J* = 8.4 Hz, 2H), 7.43 (d, *J* = 8.2 Hz, 2H), 1.35 (s, 12H), 1.34 (s, 9H). (for the minor *meta*-isomer) δ 7.86 (s, 1H), 7.66 (dt, *J* = 7.2, 1.2 Hz, 1H), 7.52 (ddd, *J* = 7.9, 2.2, 1.2 Hz, 1H), 7.33 (t, *J* = 7.3 Hz, 1H), 1.36 (s, 21H). ^{13}C NMR (101 MHz, $CDCl_3$, 23 ºC): (for the major *para*-isomer) δ 154.61, 134.83, 124.83, 83.78, 35.02, 31.33, 24.97. The spectral data match with the previously reported data.[19]

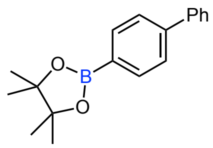

9

2-([1,1'-biphenyl]-4-yl)-4,4,5,5-tetramethyl-1,3,2-dioxaborolane, 9. 48 mg (0.171 mmol, 43%) of a colorless solid was obtained by flash column chromatography using 100% Hex then Hex/EtOAc (25:1) as eluents. ^{1}H NMR (400 MHz, $CDCl_3$, 23 ºC): δ 7.93 (d, *J* = 8.3 Hz, 2H), 7.67– 7.62 (m, 4H), 7.49 – 7.44 (m, 2H), 7.41 – 7.37 (m, 1H) 1.39 (s, 12H). ^{13}C NMR (101 MHz, $CDCl_3$, 23 ºC): δ 144.00, 141.11, 135.38, 128.89, 127.67, 127.34, 126.58, 83.93, 25.00. The spectral data match with the previously reported data.[20]

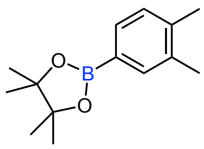

10

2-(3,4-dimethylphenyl)-4,4,5,5-tetramethyl-1,3,2-dioxaborolane, 10. 49 mg (0.211 mmol, 53%) of a colorless solid was obtained by flash column chromatography using 100% Hex then Hex/EtOAc (25:1, R_f = 0.25) as eluents. ^{1}H NMR (400 MHz, $CDCl_3$, 23 ºC): δ 7.59 (s, 1H), 7.56 (d, *J* = 7.5 Hz, 1H), 7.15 (d, *J* = 7.5 Hz, 1H), 2.28 (s, 3H), 2.27 (s, 3H), 1.34 (s, 12H). ^{13}C NMR (101 MHz, $CDCl_3$, 23 ºC): δ 136.07, 132.55, 129.31, 83.72, 24.98, 20.15, 19.61. The spectral data match with the previously reported data.[21]

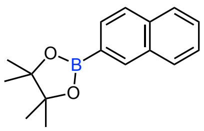

11

4,4,5,5-tetramethyl-2-(naphthalen-2-yl)-1,3,2-dioxaborolane, 11. 39 mg (0.153 mmol, 38%) of a colorless oil was obtained by flash column chromatography using 100% Hex then Hex/EtOAc (25:1) as eluents. ^{1}H NMR (400 MHz, $CDCl_3$, 23 ºC): δ 8.42 (d, *J* = 1.3 Hz, 1H), 7.91 (dd, *J* = 7.8, 1.5 Hz, 1H), 7.89 (dd, *J* = 8.2, 1.2 Hz, 1H), 7.87 – 7.83 (m, 2H), 7.60 – 7.45 (m, 2H), 1.42 (s, 12H). ^{13}C NMR (101 MHz, $CDCl_3$, 23 ºC): δ 136.38, 135.16, 132.94, 130.53, 128.78, 127.83, 127.11, 127.09, 125.92, 84.04, 25.05. The spectral data match with the previously reported data.[22]

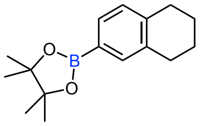

12

4,4,5,5-tetramethyl-2-(5,6,7,8-tetrahydronaphthalen-2-yl)-1,3,2-dioxaborolane, 12. 55 mg (0.213 mmol, 53%) of a colorless oil was obtained by flash column chromatography using 100% Hex then Hex/EtOAc (25:1, R_f = 0.30) as eluents. ^{1}H NMR (400 MHz, $CDCl_3$, 23 ºC): δ 7.53 (s, 1H), 7.52 (d, *J* = 7.0 Hz, 1H), 7.08 (d, *J* = 8.3 Hz, 1H), 2.78 (dq, *J* = 6.7, 3.1 Hz, 4H), 1.85 – 1.74 (m, 4H), 1.34 (s, 12H). ^{13}C NMR (101 MHz, $CDCl_3$, 23 ºC): δ 140.92, 136.66, 135.84, 131.86, 128.78, 83.71, 29.81, 29.31, 24.97, 23.38, 23.24. The spectral data match with the previously reported data.[23]

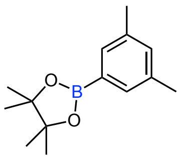

13

2-(3,5-dimethylphenyl)-4,4,5,5-tetramethyl-1,3,2-dioxaborolane, 13. 67 mg (0.289 mmol, 72%) of a colorless solid was obtained by flash column chromatography using 100% Hex then Hex/EtOAc (25:1) as eluents. ^{1}H NMR (400 MHz, $CDCl_3$, 23 ºC): (for the major *meta*-isomer) δ 7.47 (s, 2H), 7.12 (s, 1H), 2.34 (s, 6H), 1.36 (s, 12H). (for the minor *ortho*-isomer) δ 7.69 (d, J = 8.2 Hz, 1H), 7.01 (s, 1H), 7.00 (d, J = 8.2 Hz, 1H), 2.53 (s, 3H), 2.33 (s, 3H), 1.35 (s, 12H). ^{13}C NMR (101 MHz, $CDCl_3$, 23 ºC): (for the major *meta*-isomer) δ 137.27, 133.11, 132.52, 83.78, 24.97, 21.25. The spectral data match with the previously reported data.[24]

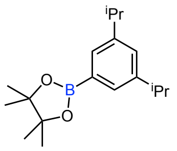

14

2-(3,5-diisopropylphenyl)-4,4,5,5-tetramethyl-1,3,2-dioxaborolane, 14. 58 mg (0.201 mmol, 50%) of a colorless solid was obtained by flash column chromatography using 100% Hex then Hex/EtOAc (25:1, R_f = 0.25) as eluents. ^{1}H NMR (400 MHz, $CDCl_3$, 23 ºC): δ 7.53 (d, J = 2.0 Hz, 2H), 7.21 (t, J = 1.9 Hz, 1H), 2.93 (hept, J = 6.9 Hz, 2H), 1.37 (s, 12H), 1.29 (d, J = 7.0 Hz, 12H). ^{13}C NMR (101 MHz, $CDCl_3$, 23 ºC): δ 148.27, 130.59, 127.82, 83.73, 34.35, 25.01, 24.23. The spectral data match with the previously reported data.[25]

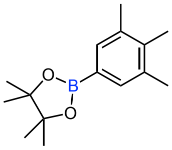

15

4,4,5,5-tetramethyl-2-(3,4,5-trimethylphenyl)-1,3,2-dioxaborolane, 15. 63 mg (0.256 mmol, 64%) of a colorless solid was obtained by flash column chromatography using 100% Hex then Hex/EtOAc (25:1) as eluents. ^{1}H NMR (400 MHz, $CDCl_3$, 23 ºC): δ 7.50 (s, 2H), 2.33 (s, 6H), 2.23 (s, 3H), 1.38 (s, 12H). ^{13}C NMR (101 MHz, $CDCl_3$, 23 ºC): δ 138.89, 135.91, 134.05, 83.64, 24.93, 20.41, 15.73. The spectral data match with the previously reported data.[26]

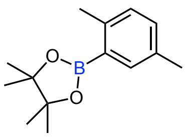

16

2-(2,5-dimethylphenyl)-4,4,5,5-tetramethyl-1,3,2-dioxaborolane, 16. 73 mg (0.314 mmol, 79%) of a colorless oil was obtained by flash column chromatography using 100% Hex then Hex/EtOAc (25:1) as eluents. ^{1}H NMR (400 MHz, $CDCl_3$, 23 ºC): δ 7.58 (d, J = 2.0 Hz, 1H), 7.14 (dd, J = 7.7, 2.1 Hz, 1H), 7.10 – 7.03 (m, 1H), 2.50 (s, 3H), 2.31 (s, 3H), 1.35 (s, 12H). ^{13}C NMR (101 MHz, $CDCl_3$, 23 ºC): δ 141.85, 136.49, 134.04, 131.69, 129.93, 83.49, 25.03, 21.83, 20.92. The spectral data match with the previously reported data.[24]

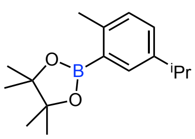

17

2-(5-isopropyl-2-methylphenyl)-4,4,5,5-tetramethyl-1,3,2-dioxaborolane, 17. 69 mg (0.265 mmol, 66%) of a colorless solid was obtained by flash column chromatography using 100% Hex then Hex/EtOAc (25:1, R_f = 0.32) as eluents. ESI+: *m/z* calculated for $C_{16}H_{25}BO_2$ ($[M+H]^+$) 260.1948, found *m/z* 260.1909. ^{1}H NMR (400 MHz, $CDCl_3$, 23 ºC): δ 7.62 (d, J = 2.3 Hz, 1H), 7.21 (dd, J = 7.8, 2.2 Hz, 1H), 7.11 (d, J = 7.9 Hz, 1H), 2.90 (hept, J = 6.9 Hz, 1H), 2.51 (s, 3H), 1.35 (s, 12H), 1.25 (d, J = 6.8 Hz, 6H). ^{13}C NMR (101 MHz, $CDCl_3$, 23 ºC): δ 145.15, 142.40, 134.21, 130.05, 128.78, 83.46, 77.48, 77.16, 76.84, 33.82, 25.04, 24.24, 21.91. ^{11}B NMR (128 MHz, $CDCl_3$) δ 31.67 (br s).

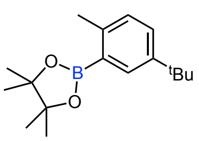

18

2-(5-(*tert*-butyl)-2-methylphenyl)-4,4,5,5-tetramethyl-1,3,2-dioxaborolane, 18. 44 mg (0.160 mmol, 40%) of a colorless oil was obtained by flash column chromatography using 100% Hex then Hex/EtOAc (25:1, R_f = 0.46) as eluents. ^{1}H NMR (400 MHz, $CDCl_3$, 23 ºC): δ 7.79 (t, J = 2.0 Hz, 1H), 7.37 (dd, J = 8.0, 2.2 Hz, 1H), 7.12 (d, J = 8.0 Hz, 1H), 2.52 (s, 3H), 1.36 (s, 12H), 1.34 (s, 9H). ^{13}C NMR (101 MHz, $CDCl_3$, 23 ºC): δ 147.28, 142.01, 132.54, 129.77, 128.02, 83.41, 34.41, 31.57, 25.04, 21.81. The spectral data match with the previously reported data.[24]

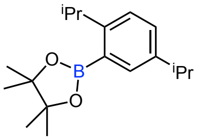

19

2-(2,5-diisopropylphenyl)-4,4,5,5-tetramethyl-1,3,2-dioxaborolane, 19. 54 mg (0.187 mmol, 47%) of a colorless oil was obtained by flash column chromatography using 100% Hex then Hex/EtOAc (25:1, R_f = 0.37) as eluents. DART-Orbitrap: *m/z* calculated for $C_{18}H_{30}BO_2$ ($[M+H]^+$) 289.2339, found *m/z* 289.2327. ^{1}H NMR (400 MHz, $CDCl_3$, 23 ºC): δ 7.57 (s, 1H), 7.27-7.25 (m, 2H), 3.65 (p, J = 6.9 Hz, 1H), 2.89 (p, J = 6.9 Hz, 1H), 1.35 (s, 12H), 1.25 (d, J = 7.0 Hz, 6H), 1.23 (d, J = 6.8 Hz, 6H). ^{13}C NMR (101 MHz, $CDCl_3$, 23 ºC): δ 153.01, 145.05, 133.99, 128.87, 124.61, 83.43, 33.78, 31.31, 24.97, 24.63, 24.19. ^{11}B NMR (128 MHz, $CDCl_3$, 23 ºC): δ 32.75.

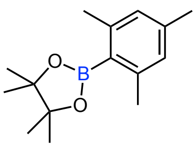

20

2-mesityl-4,4,5,5-tetramethyl-1,3,2-dioxaborolane, 20. The deconstruction step was conducted for 48 h instead of 24 h. 82 mg (0.33 mmol, 83%) of a colorless oil was obtained by flash column chromatography using 100% Hex then Hex/EtOAc (25:1) as eluents. ^{1}H NMR (400 MHz, $CDCl_3$, 23 ºC): δ 6.78 (s, 2H), 2.38 (s, 6H), 2.25 (s, 3H), 1.38 (s, 12H). ^{13}C NMR (101 MHz, $CDCl_3$, 23 ºC): δ 142.25, 139.04, 127.57, 83.57, 25.07, 22.31, 21.35. The spectral data match with the previously reported data.[24]

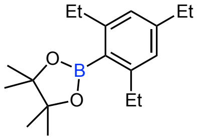

21

4,4,5,5-tetramethyl-2-(2,4,6-triethylphenyl)-1,3,2-dioxaborolane, 21. The deconstruction step was conducted for 48 h instead of 24 h. 58 mg (0.201 mmol, 50%) of a light yellow oil was obtained by flash column chromatography using 100% Hex then Hex/EtOAc (25:1) as eluents. ^{1}H NMR (400 MHz, $CDCl_3$, 23 ºC): δ 6.83 (s, 2H), 2.67 (q, J = 7.6 Hz, 4H), 2.58 (q, J = 7.6 Hz, 2H), 1.38 (s, 12H), 1.21 (t, J = 7.6 Hz, 6H), 1.20 (t, J = 7.6 Hz, 3H). ^{13}C NMR (101 MHz, $CDCl_3$, 23 ºC): δ 148.50, 145.52, 124.97, 83.66, 29.58, 29.02, 25.10, 17.09, 15.67. The spectral data match with the previously reported data.[24]

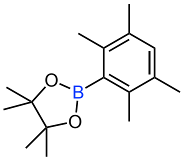

22

4,4,5,5-tetramethyl-2-(2,3,5,6-tetramethylphenyl)-1,3,2-dioxaborolane, 22. The deconstruction step was conducted for 60 h instead of 24 h. 51 mg (0.196 mmol, 49%) of a colorless solid was obtained by flash column chromatography using 100% Hex then Hex/EtOAc (25:1, R_f = 0.34) as eluents. ^{1}H NMR (400 MHz, $CDCl_3$, 23 ºC): δ 6.89 (s, 1H), 2.25 (s, 6H), 2.17 (s, 6H), 1.40 (s, 12H). ^{13}C NMR (101 MHz, $CDCl_3$, 23 ºC): δ 136.76, 133.18, 132.47, 83.83, 25.24, 19.76, 19.05. The spectral data match with the previously reported data.[24]

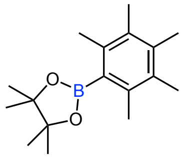

23

4,4,5,5-tetramethyl-2-(2,3,4,5,6-pentamethylphenyl)-1,3,2-dioxaborolane, 23. The deconstruction step was conducted for 48 h instead of 24 h. 35 mg (0.128 mmol, 32%) of a colorless solid was obtained by flash column chromatography using 100% Hex then Hex/EtOAc (25:1, R_f = 0.30) as eluents. ^{1}H NMR (400 MHz, $CDCl_3$, 23 ºC): δ 2.30 (s, 6H), 2.21 (s, 3H), 2.17 (s, 6H), 1.41 (s, 12H). ^{13}C NMR (101 MHz, $CDCl_3$, 23 ºC): δ 136.01, 135.81, 131.91, 83.66, 25.21, 20.48, 16.74, 16.09. The spectral data match with the previously reported data.[27]

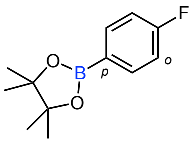

24

2-(4-fluorophenyl)-4,4,5,5-tetramethyl-1,3,2-dioxaborolane, 24. 35 mg (0.158 mmol, 39%) of a colorless oil was obtained by flash column chromatography using 100% Hex then Hex/EtOAc (25:1) as eluents. ^{1}H NMR (400 MHz, $CDCl_3$, 23 ºC): (for the major *para*-isomer) δ 7.80 (dd, J = 8.7, 6.2 Hz, 2H), 7.09 – 7.00 (m, 2H), 1.34 (s, 12H). ^{13}C NMR (101 MHz, $CDCl_3$, 23 ºC): (for the major *para*-isomer) δ 137.08, 114.98 (d, J = 20.4 Hz), 84.05, 25.01. *The carbon *ipso* to F is missing. ^{19}F{^{1}H} NMR (376 MHz, $CDCl_3$, 23 ºC): (*para*-isomer) -108.44, (*ortho*-isomer) -102.62. The spectral data match with the previously reported data.[28]

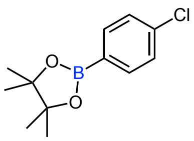

25

2-(4-chlorophenyl)-4,4,5,5-tetramethyl-1,3,2-dioxaborolane, 25. 21 mg (0.088 mmol, 22%) of a colorless oil was obtained by flash column chromatography using 100% Hex then Hex/EtOAc (25:1) as eluents. ^{1}H NMR (400 MHz, $CDCl_3$, 23 ºC): (for the major *para*-isomer) δ 7.73 (d, J = 8.3 Hz, 2H), 7.34 (d, J = 8.4 Hz, 2H), 1.34 (s, 12H). ^{13}C NMR (101 MHz, $CDCl_3$, 23 ºC): (for the major *para*-isomer) δ 137.67, 136.26, 128.15, 84.15, 77.48, 77.16, 76.84, 25.00. The spectral data match with the previously reported data.[28]

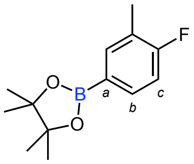

26

2-(4-fluoro-3-methylphenyl)-4,4,5,5-tetramethyl-1,3,2-dioxaborolane, 26. 51 mg (0.216 mmol, 54%) of a colorless oil was obtained by flash column chromatography using 100% Hex then Hex/EtOAc (25:1, R_f = 0.18) as eluents. ^{1}H NMR (400 MHz, $CDCl_3$, 23 ºC): (for the major *a*-isomer) δ 7.65 (d, J = 8.4 Hz, 1H), 7.61 (ddd, J = 7.9, 5.7, 1.7 Hz, 1H), 6.99 (dd, J = 10.0, 8.1 Hz, 1H), 2.27 (d, J = 2.0 Hz, 3H), 1.34 (s, 12H). ^{13}C NMR (101 MHz, $CDCl_3$, 23 ºC): (for the major *a*-isomer) δ 138.52, 134.39, 114.57, 83.97, 25.00, 14.27. *Two carbons in the benzene ring are missing. ^{19}F{^{1}H} NMR (376 MHz, $CDCl_3$, 23 ºC): (*a*-isomer) -112.9, (*b*-isomer) -119.0, (*c*-isomer) -106.9. The spectral data match with the previously reported data.[28]

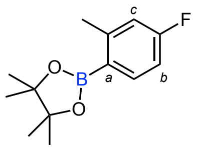

27

2-(4-fluoro-2-methylphenyl)-4,4,5,5-tetramethyl-1,3,2-dioxaborolane, 27. 55 mg (0.233 mmol, 58%) of a colorless oil was obtained by flash column chromatography using 100% Hex then Hex/EtOAc (25:1, R_f = 0.15) as eluents. ^{1}H NMR (400 MHz, $CDCl_3$, 23 ºC): (for the major *a*-isomer) δ 7.75 (t, *J* = 7.4 Hz, 1H), 6.88 – 6.83 (m, 4H), 2.54 (s, 3H), 1.34 (s, 12H). (for the minor *b*-isomer) δ 7.62 (t, *J* = 7.0 Hz, 1H), 6.95 (d, *J* = 9.0 Hz, 1H), 6.84 (d, peak overlapped, *J* not determined, 1H), 2.36 (s, 3H), 1.36 (s, 12H). (for the minor *c*-isomer) δ 7.22 (td, *J* = 8.0, 6.4 Hz, 1H), 6.93 (d, *J* = 9.3 Hz, 1H), 6.81 (d, *J* = 9.3 Hz, 1H), 2.45 (s, 3H), 1.39 (s, 12H). ^{13}C NMR (101 MHz, $CDCl_3$, 23 ºC): (for the major *a*-isomer) δ 166.00, 163.52, 138.23 (d, *J* = 8.9 Hz), 116.75 (d, *J* = 19.7 Hz), 111.86 (d, *J* = 19.7 Hz), 83.62, 25.03, 22.30. ^{19}F{^{1}H} NMR (376 MHz, $CDCl_3$, 23 ºC): (*a*-isomer) -110.2, (*b*-isomer) -103.8, (*c*-isomer) -104.4. The spectral data match with the previously reported data.[19,29]

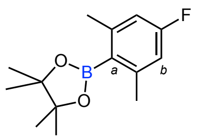

28

2-(4-fluoro-2,6-dimethylphenyl)-4,4,5,5-tetramethyl-1,3,2-dioxaborolane (*a*-isomer), 2-(2-fluoro-4,6-dimethylphenyl)-4,4,5,5-tetramethyl-1,3,2-dioxaborolane (*b*-isomer), 28. 66 mg (0.264 mmol, 66%) of a colorless oil was obtained by flash column chromatography using 100% Hex then Hex/EtOAc (25:1, R_f = 0.37) as eluents. ^{1}H NMR (400 MHz, $CDCl_3$, 23 ºC): (for the major *a*-isomer) δ 6.66 (d, *J* = 9.9 Hz, 1H), 2.40 (s, 6H), 1.38 (s, 12H). (for the minor *b*-isomer) δ 6.75 (s, 1H), 6.65 (d, *J* = 10.0 Hz, 1H), 2.41 (s, 3H), 2.28 (s, 3H), 1.37 (s, 12H). ^{13}C NMR (101 MHz, $CDCl_3$, 23 ºC): (for the major *a*-isomer) δ 163.62 (d, *J* = 246.7 Hz), 144.99 (d, *J* = 8.1 Hz), 113.52 (d, *J* = 19.8 Hz), 83.86, 25.07, 22.39 (d, *J* = 2.1 Hz). (for the minor *b*-isomer) δ 167.07 (d, *J* = 244.7 Hz), 145.14 (d, *J* = 8.5 Hz), 142.26 (d, *J* = 9.4 Hz), 126.40 (d, *J* = 2.3 Hz), 112.81 (d, *J* = 24.4 Hz), 83.82, 24.93, 22.15 (d, *J* = 2.2 Hz), 21.38 (d, *J* = 2.2 Hz). ^{19}F{^{1}H} NMR (376 MHz, $CDCl_3$, 23 ºC): (*a*-isomer) -113.6, (*b*-isomer) -105.2. The spectral data match with the previously reported data.[27]

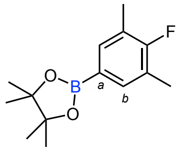

29

2-(4-fluoro-3,5-dimethylphenyl)-4,4,5,5-tetramethyl-1,3,2-dioxaborolane, 29. 54 mg (0.216 mmol, 54%) of a colorless oil was obtained by flash column chromatography using 100% Hex then Hex/EtOAc (25:1, R_f = 0.46) as eluents. ESI+: *m/z* calculated for $C_{14}H_{21}BFO_2$ ($[M+H]^+$) 251.1619, found *m/z* 251.1635. ^{1}H NMR (400 MHz, $CDCl_3$, 23 ºC): (for the major *a*-isomer) δ 7.48 (d, *J* = 7.2 Hz, 2H), 2.26 (d, *J* = 2.2 Hz, 6H), 1.34 (s, 12H). (for the minor *b*-isomer) δ 7.42 (d, *J* = 7.5 Hz, 2H), 6.99 (t, *J* = 7.0 Hz, 1H), 2.45 (d, *J* = 2.7 Hz, 3H), 2.26 (s, 3H), 1.34 (s, 12H). ^{13}C NMR (101 MHz, $CDCl_3$, 23 ºC): (for the major *a*-isomer) δ 162.44 (d, *J* = 248.5 Hz), 135.86 (d, *J* = 5.8 Hz), 129.54 (dd, *J* = 293.8, 4.4 Hz), 124.02 (d, *J* = 17.3 Hz), 83.90, 24.98, 14.49 (d, *J* = 4.6 Hz). ^{19}F{^{1}H} NMR (376 MHz, $CDCl_3$, 23 ºC): (*a*-isomer) -117.3, (*b*-isomer) -122.1.

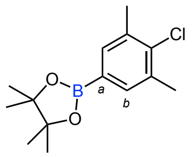

30

2-(4-chloro-3,5-dimethylphenyl)-4,4,5,5-tetramethyl-1,3,2-dioxaborolane, 30. 41 mg (0.154 mmol, 38%) of a colorless oil was obtained by flash column chromatography using 100% Hex then Hex/EtOAc (25:1, R_f = 0.33) as eluents. ESI+: *m/z* calculated for $C_{14}H_{21}BClO_2$ ($[M+H]^+$) 267.1323, found *m/z* 267.1367. ^{1}H NMR (400 MHz, $CDCl_3$, 23 ºC): (for the major *a*-isomer) δ 7.51 (s, 2H), 2.38 (s, 6H), 1.34 (s, 12H). (for the minor *b*-isomer) δ 7.55 (d, *J* = 7.6 Hz, 1H), 7.06 (d, *J* = 7.1 Hz, 1H), 2.60 (s, 3H), 2.39 (s, 3H), 1.34 (s, 12H). ^{13}C NMR (101 MHz, $CDCl_3$, 23 ºC): (for the major *a*-isomer) δ 138.28, 135.78, 134.79, 84.04, 24.99, 20.59. ^{11}B NMR (128 MHz, $CDCl_3$, 23 ºC): δ 31.05 (br s).

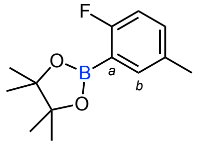

31

2-(2-fluoro-5-methylphenyl)-4,4,5,5-tetramethyl-1,3,2-dioxaborolane, 31. 35 mg (0.148 mmol, 37%) of a colorless oil was obtained by flash column chromatography using 100% Hex then Hex/EtOAc (25:1, R_f = 0.38&0.29) as eluents. ^{1}H NMR (400 MHz, $CDCl_3$, 23 ºC): (for the major *a*-isomer) δ 7.52 (dd, *J* = 5.9, 2.6 Hz, 1H), 7.21 (ddd, *J* = 8.1, 5.4, 2.5 Hz, 1H), 6.91 (t, *J* = 8.8 Hz, 1H), 2.31 (s, 3H), 1.36 (s, 12H). (for the minor *b*-isomer) δ 7.42 (dd, *J* = 9.4, 3.0 Hz, 1H), 7.10 (dd, *J* = 8.4, 5.3 Hz, 1H), 6.98 (td, *J* = 8.4, 3.0 Hz, 1H), 2.49 (s, 3H), 1.34 (s, 12H). ^{13}C NMR (101 MHz, $CDCl_3$, 23 ºC): (for the major *a*-isomer) δ 137.08, 133.93, 132.94, 115.21, 83.98, 24.96, 20.55. *The carbon *ipso* to F is missing. ^{19}F{^{1}H} NMR (376 MHz, $CDCl_3$, 23 ºC): (*a*-isomer) -108.3, (*b*-isomer) -119.7. The spectral data match with the previously reported data.[30]

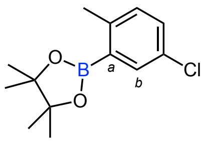

32

2-(5-chloro-2-methylphenyl)-4,4,5,5-tetramethyl-1,3,2-dioxaborolane (*a*-isomer), 2-(2-chloro-5-methylphenyl)-4,4,5,5-tetramethyl-1,3,2-dioxaborolane (*b*-isomer), 32. 26 mg (0.103 mmol, 26%) of a colorless oil was obtained by flash column chromatography using 100% Hex then Hex/EtOAc (25:1) as eluents. ^{1}H NMR (400 MHz, $CDCl_3$, 23 ºC): (for the major *a*-isomer) δ 7.71 (d, *J* = 2.5 Hz, 1H), 7.26 (dd, *J* = 8.1, 2.5 Hz, 1H), 7.08 (dt, *J* = 8.2, 0.6 Hz, 1H), 2.49 (s, 3H), 1.34 (s, 12H). (for the minor *b*-isomer) δ 7.48 (d, *J* = 2.3 Hz, 1H), 7.22 (d, *J* = 8.2 Hz, 1H), 7.13 (ddt, *J* = 8.1, 2.3, 0.7 Hz, 1H), 2.30 (s, 3H), 1.37 (s, 12H). ^{13}C NMR (101 MHz, $CDCl_3$, 23 ºC): (for the major *a*-isomer) δ 143.24, 135.60, 135.48, 131.40, 130.94, 130.72, 83.93, 25.02, 21.68. (for the major *b*-isomer) δ 137.07, 136.64, 132.76, 129.32, 84.25, 24.95, 20.79. *Two carbons are missing. The spectral data match with the previously reported data.[31]

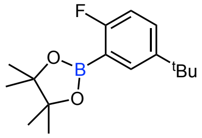

33

2-(5-(*tert*-butyl)-2-fluorophenyl)-4,4,5,5-tetramethyl-1,3,2-dioxaborolane, 33. 29 mg (0.104 mmol, 26%) of a yellow oil was obtained by flash column chromatography using 100% Hex then Hex/EtOAc (25:1, R_f = 0.35) as eluents. **24** was detected as a minor side product. ESI-: *m/z* calculated for $C_{16}H_{23}BFO_2$ ($[M-H]^-$) 277.1775, found *m/z* 277.1796. ^{1}H NMR (400 MHz, $CDCl_3$, 23 ºC): δ 7.72 (dd, *J* = 5.7, 2.7 Hz, 1H), 7.44 (ddd, *J* = 8.8, 5.3, 2.8 Hz, 1H), 6.95 (t, *J* = 8.9 Hz, 1H), 1.37 (s, 12H), 1.33 (s, 9H). ^{13}C NMR (101 MHz, $CDCl_3$, 23 ºC): 165.52 (d, *J* = 248.9 Hz), 146.22 (d, *J* = 3.4 Hz), 133.32 (d, *J* = 7.8 Hz), 130.48 (d, *J* = 8.7 Hz), 114.78 (d, *J* = 23.8 Hz), 83.91, 34.47, 31.65, 24.97. ^{19}F{^{1}H} NMR (376 MHz, $CDCl_3$, 23 ºC): -108.3.

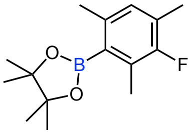

34

2-(3-fluoro-2,4,6-trimethylphenyl)-4,4,5,5-tetramethyl-1,3,2-dioxaborolane, 34. 68 mg (0.257 mmol, 64%) of a colorless oil was obtained by flash column chromatography using 100% Hex then Hex/EtOAc (25:1, R_f = 0.11) as eluents. ^{1}H NMR (400 MHz, $CDCl_3$, 23 ºC): δ 6.77 (dd, *J* = 6.9, 0.7 Hz, 1H), 2.33 (s, 3H), 2.30 (d, *J* = 2.5 Hz, 3H), 2.20 (d, *J* = 1.3 Hz, 3H), 1.39 (s, 12H). ^{13}C NMR (101 MHz, $CDCl_3$, 23 ºC): 158.10 (d, *J* = 240.5 Hz), 136.77 (d, *J* = 4.6 Hz), 130.04 (d, *J* = 4.6 Hz), 127.89 (d, *J* = 16.1 Hz), 125.31 (d, *J* = 18.5 Hz), 83.91, 25.08, 21.60, 14.76 (d, *J* = 4.5 Hz), 14.18 (d, *J* = 5.5 Hz). ^{19}F{^{1}H} NMR (376 MHz, $CDCl_3$, 23 ºC): -127.4. The spectral data match with the previously reported data.[24]

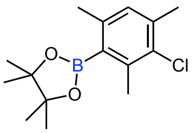

35

2-(3-chloro-2,4,6-trimethylphenyl)-4,4,5,5-tetramethyl-1,3,2-dioxaborolane, 35. 44 mg (0.157 mmol, 39%) of a colorless oil was obtained by flash column chromatography using 100% Hex then Hex/EtOAc (25:1, R_f = 0.29) as eluents. ^{1}H NMR (400 MHz, $CDCl_3$, 23 ºC): δ 6.86 (s, 1H), 2.42 (s, 3H), 2.32 (s, 3H), 2.32 (s, 3H), 1.39 (s, 12H). ^{13}C NMR (101 MHz, $CDCl_3$, 23 ºC): 139.41, 139.14, 137.07, 132.19, 129.76, 84.05, 77.48, 77.16, 76.84, 25.11, 21.74, 21.00, 20.53. The spectral data match with the previously reported data.[24]

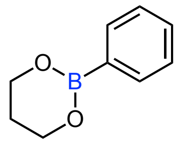

36

2-phenyl-1,3,2-dioxaborinane, 36. 28 mg (0.173 mmol, 39%) of a colorless oil was obtained by flash column chromatography using Hex/EtOAc (10:1, R_f = 0.11) as an eluent. ^{1}H NMR (400 MHz, $CDCl_3$, 23 ºC): 7.78 (d, *J* = 8.2 Hz, 2H), 7.42 (t, *J* = 7.3 Hz, 1H), 7.35 (t, *J* = 7.1 Hz, 2H), 4.17 (t, *J* = 5.5 Hz, 4H), 2.06 (p, *J* = 5.5 Hz, 2H). ^{13}C NMR (101 MHz, $CDCl_3$, 23 ºC): 133.76, 130.72, 127.69, 62.10, 27.58. The spectral data match with the previously reported data.[32]

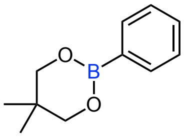

37

5,5-dimethyl-2-phenyl-1,3,2-dioxaborinane, 37. 45 mg (0.237 mmol, 59%) of a colorless oil was obtained by flash column chromatography using Hex/EtOAc (25:1, R_f = 0.38) as an eluent. ^{1}H NMR (400 MHz, $CDCl_3$, 23 ºC): 7.81 (d, *J* = 6.7 Hz, 2H), 7.43 (t, *J* = 7.3 Hz, 1H), 7.36 (t, *J* = 7.6 Hz, 2H), 3.78 (s, 4H), 1.03 (s, 6H). ^{13}C NMR (101 MHz, $CDCl_3$, 23 ºC): 133.96, 130.81, 127.71, 72.45, 32.03, 22.05. The spectral data match with the previously reported data.[33]

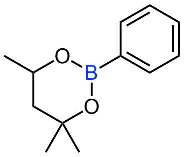

38

4,4,6-trimethyl-2-phenyl-1,3,2-dioxaborinane, 38. 44 mg (0.216 mmol, 54%) of a colorless oil was obtained by flash column chromatography using Hex/EtOAc (25:1, R_f = 0.34 at Hex:EA = 50:1) as an eluent. ^{1}H NMR (400 MHz, $CDCl_3$, 23 ºC): 7.82 (d, *J* = 6.5 Hz, 2H), 7.40 (t, *J* = 7.3 Hz, 1H), 7.34 (t, *J* = 7.1 Hz, 2H), 4.35 (ddh, *J* = 12.3, 6.2, 2.9 Hz, 1H), 1.87 (dd, *J* = 13.9, 3.0 Hz, 1H), 1.64 – 1.56 (m, 1H), 1.39 (s, 3H), 1.37 (s, 3H), 1.35 (d, *J* = 6.2 Hz, 3H). ^{13}C NMR (101 MHz, $CDCl_3$, 23 ºC): 133.89, 130.46,

127.58, 71.10, 65.11, 46.20, 31.45, 28.32, 23.38. The spectral data match with the previously reported data.[33]

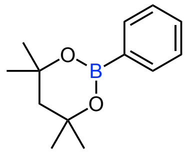
39

4,4,6,6-tetramethyl-2-phenyl-1,3,2-dioxaborinane, 39. 41 mg (0.188 mmol, 47%) of a colorless solid was obtained by flash column chromatography using Hex/EtOAc (50:1, R_f = 0.47) as an eluent. ^{1}H NMR (400 MHz, $CDCl_3$, 23 ºC): 7.90 – 7.83 (m, 2H), 7.46 – 7.39 (m, 1H), 7.39 – 7.31 (m, 2H), 1.93 (s, 2H), 1.45 (s, 12H). ^{13}C NMR (101 MHz, $CDCl_3$, 23 ºC): 133.95, 130.40, 127.54, 70.91, 49.13, 31.97. The spectral data match with the previously reported data.[34]

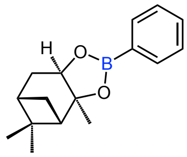
40

(3a*S*,4*S*,6*S*,7a*R*)-3a,5,5-trimethyl-2-phenylhexahydro-4,6-methanobenzo[*d*][1,3,2]dioxaborole, 40. 57 mg (0.223 mmol, 56%) of a colorless solid was obtained by flash column chromatography using Hex/EtOAc (25:1, R_f = 0.43) as an eluent. ^{1}H NMR (400 MHz, $CDCl_3$, 23 ºC): 7.85 (d, J = 6.6 Hz, 2H), 7.48 (t, J = 7.4 Hz, 1H), 7.39 (t, J = 7.2 Hz, 2H), 4.47 (dd, J = 8.8, 2.1 Hz, 1H), 2.44 (ddt, J = 14.4, 8.8, 2.4 Hz, 1H), 2.31 – 2.22 (m, 1H), 2.18 (dd, J = 6.1, 5.0 Hz, 1H), 2.10 – 1.89 (m, 2H), 1.51 (s, 3H), 1.33 (s, 3H), 1.25 (d, J = 10.8 Hz, 1H), 0.91 (s, 3H). ^{13}C NMR (101 MHz, $CDCl_3$, 23 ºC): 134.91, 131.30, 127.86, 86.34, 78.37, 51.55, 39.66, 38.31, 35.70, 28.84, 27.24, 26.62, 24.18. The spectral data match with the previously reported data.[35]

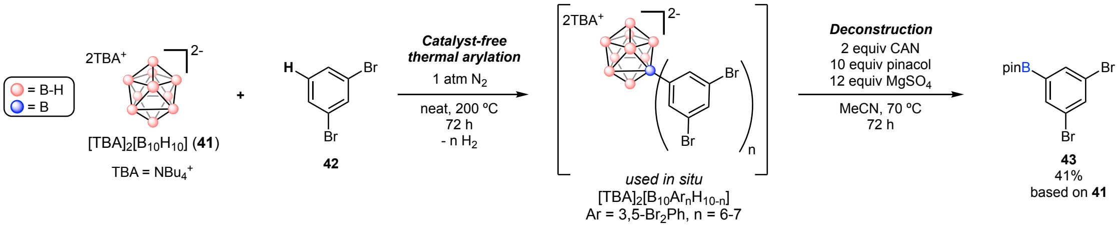

Synthesis of 43. In an N_2 atmosphere, 200 mg (1.0 equiv., 0.33 mmol) of **41** and 0.6 mL 1,3-dibromobzene (**42**) were added to a thick-walled 10-mL microwave vial with a septum. The vial was connected to a Schlenk line to release the pressure which can be made by H_2 evolution during the reaction. The reaction mixture was heated at 200 ºC and stirred for 72 h. The reaction mixture was cooled down to room temperature and the resulting liquid was washed with hexane (10 mL x 3) to remove an excess amount of remaining 1,3-dibromobenzene to give a slightly brownish gummy goo. Using 10 mL MeCN, this gummy residue was transferred to a Schlenk flask which was filled with 362 mg (0.66 mmol, 2 equiv) of CAN, 390 mg (3.3 mmol, 10 equiv) of pinacol, and 477 mg (3.96 mmol, 12 equiv) of $MgSO_4$ in air. Under aerobic conditions, the reaction mixture was heated at 70 ºC and stirred for 72 h. The crude mixture was then filtered through a Celite plug and condensed *in vacuo*. The resulting residue was purified by silica gel flash column chromatography using an eluent (Hex:EA = 25:1, R_f = 0.26). Some fractions contained **43** as well as an impurity, thus the second flash column chromatography was performed to obtain more amount of **43**. Then, fractions were collected, and the volatiles were removed under vacuum. Finally, the resulting solid was further dried to give 49 mg (0.135 mmol, 41 %) of a yellowish oil identified as **43** by NMR spectroscopy. ^{1}H NMR (400 MHz, C_6D_6, 23 ºC): δ 7.84 (d, J = 1.9 Hz, 2H), 7.74 (t, J = 1.8 Hz, 1H), 1.34 (s, 13H). ^{13}C{^{1}H} NMR (101 MHz, C_6D_6, 23 ºC): δ 136.54, 136.01, 122.88, 84.55, 24.85. The spectral data match with the previously reported data.[36]

Preparation of 45 by Borylation of 1,3,5-Tri-isopropylbenzene. A 4-mL dram vial was charged with a magnetic stir bar, 0.40 mmol **1** (154 mg) and 0.5 mL 1,3,5-tri-isopropylenzene under aerobic conditions. The solution was cooled down to 0 ºC in an ice bath. Then, 2.0 mmol (180 µL) triflic acid was added to the reaction mixture, and the mixture was stirred for 3 h at 0 ºC. The reaction mixture was filtered through a silica plug prepared using a Pasteur pipette packed with ~2 cm length of silica gel. After the filtration, the filter cake was washed with 10 mL hexanes, and the filtrate was collected. Volatiles of the filtrate were removed under vacuum, and the resulting crude mixture was transferred to a 25 mL Schlenk flask charged with a magnetic stir bar, 0.08 mmol (20 mol%, 44 mg) CAN, 4.0 mmol (478 mg) pinacol, 4.8 mmol (580

mg) magnesium sulfate and 10 mL MeCN. The reaction mixture was stirred at 65 ºC for 48 h. After 48 h, the reaction mixture was cooled down to room temperature and filtered through a pad of Celite. The filtrate was then exposed to vacuum to remove volatiles. The crude mixture was purified by silica gel flash column chromatography using 100% hexanes then hexanes/EtOAc (25:1) as eluents (R_f = 0.27). Finally, the collected eluents were concentrated under vacuum to give 19 mg (0.0575 mmol, 14%) of a colorless solid identified as **45**.

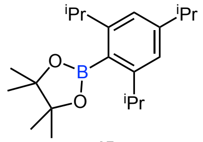

45

4,4,5,5-tetramethyl-2-(2,4,6-triisopropylphenyl)-1,3,2-dioxaborolane, 45. ^{1}H NMR (400 MHz, $CDCl_3$, 23 ºC): 6.94 (s, 2H), 2.98 (hept, J = 6.9 Hz, 2H), 2.86 (hept, J = 6.8 Hz, 1H), 1.39 (s, 12H), 1.26 (d, J = 6.8 Hz, 12H), 1.23 (d, J = 6.8 Hz, 6H). ^{13}C NMR (101 MHz, $CDCl_3$, 23 ºC): 152.04, 149.89, 119.79, 83.77, 34.65, 34.17, 25.14, 24.59, 24.16. The spectral data match with the previously reported data.[33]

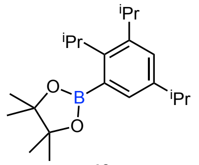

46

4,4,5,5-tetramethyl-2-(2,3,5-triisopropylphenyl)-1,3,2-dioxaborolane, 46. **46** was obtained as a very minor impurity in **45**. Only identifiable chemical shifts are reported herein. ^{1}H NMR (400 MHz, $CDCl_3$, 23 ºC): 7.63 (s, 1H), 7.21 (s, 1H), 3.66 (hept, J = 7.0 Hz, 1H), 3.33 – 3.17 (m, 2H), 1.33 (s, 12H).

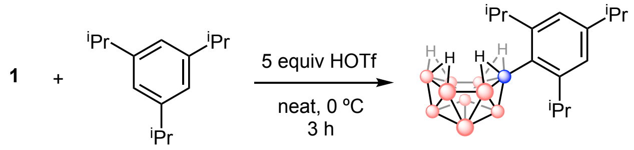

44

47
16%

Synthesis of 47, 6-[2,4,6-{$(CH_3)_2CH$}$_3C_6H_2$]-*nido*-$B_{10}H_{13}$. Under aerobic conditions, a 20 mL scintillation vial was charged with 2.6 mmol (1 g) of **1** and 5 mL 1,3,5-tri-isopropylbenzene. The solution was cooled down to 0 ºC using an ice bath. Once the solution was cooled down, 5 equiv HOTf (13 mmol, 1.15 mL) was added to the solution, and the reaction mixture was stirred at 0 ºC for 3 h. The crude mixture was purified by silica gel flash column chromatography using an eluent (Hex:EA = 25:1, R_f = 0.31). Fractions were collected, and the volatiles were removed under vacuum. Finally, the resulting solid was further dried to give 135 mg (0.416 mmol, 16 %) of a colorless solid identified as **47** by NMR spectroscopy. A single crystal of **47** was prepared by slow evaporation of a concentrated benzene solution of **47** under air at room temperature. ESI-: *m/z* calculated for $C_{15}H_{35}B_{10}$ ($[M-H]^-$) 323.3742, found *m/z* 323.3726. ^{1}H NMR (400 MHz, C_6D_6, 23 ºC): δ 7.09 (s, 2H), 3.04 (m, 2H), 2.75 (hept, J = 6.5 Hz, 1H), 1.20 (d, J = 7.0 Hz, 6H), 1.17 (d, J = 6.7 Hz, 12H), 4.77 – 0.90 (9H, br), -0.65 (s, 2H), -2.07 (s, 2H). ^{13}C{^{1}H} NMR (101 MHz, C_6D_6, 23 ºC): δ 154.24, 151.41, 121.69, 34.78, 33.78, 24.68, 24.05. ^{11}B NMR (128 MHz, C_6D_6, 23 ºC) δ 22.06 (B6, s), 8.94 (B1,B3), 6.22 (B9), 2.13 (B8,B10), -4.23 (B5B7), -32.13 (B2), -38.51 (B4) (all doublets).

NMR monitoring study of deconstruction of 2 in a J. Young NMR tube. A J. Young NMR tube was charged with 10.6 mg of **1** (1.0 equiv, 0.05 mmol), 11.8 mg of pinacol (5 equiv, 0.25 mmol) and 0.4 mL CD_3CN. The solution was frozen by liquid nitrogen and the headspace of the J. Young NMR tube was evacuated using a Schlenk line. After evacuation, the solution was slowly thawed to room temperature. This freeze-pump-thaw method was repeated two more times for complete degassing. The reaction mixture was then heated up at 65 ºC using an oil bath and the reaction progress was monitored by ^{1}H and ^{11}B NMR spectroscopy. Full ranged stacked ^{1}H and ^{11}B NMR spectra can be found on page S83-84 (Fig. S142 and Fig. S143).

III. Supplementary Figures

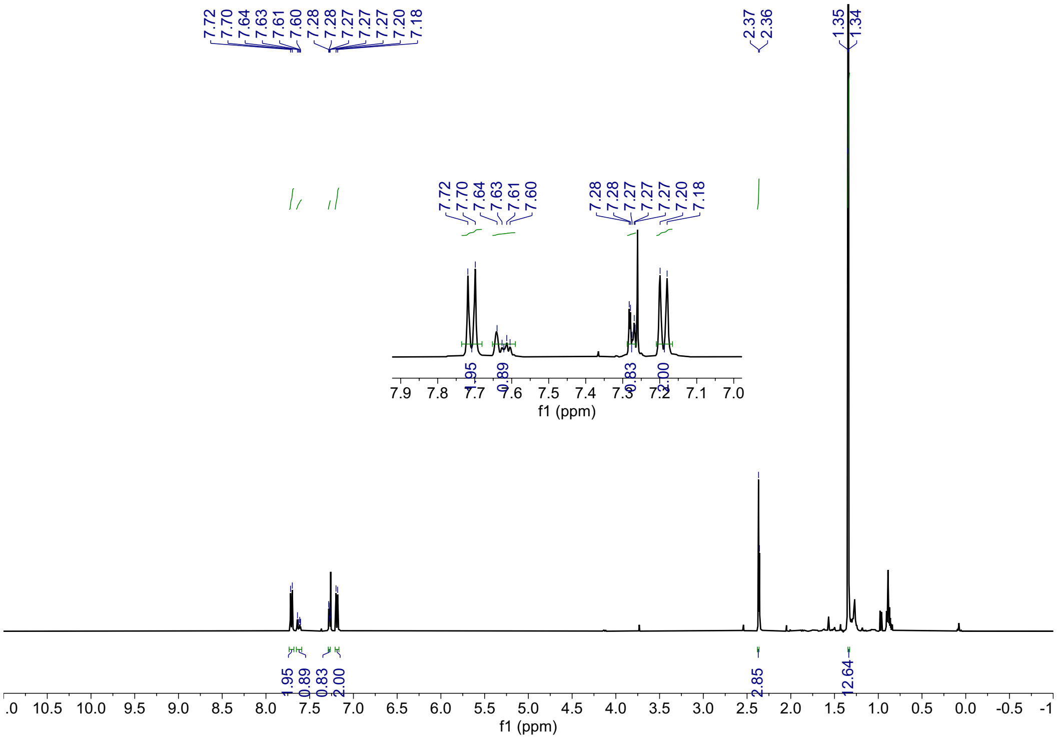

Supplementary Figure 1. ^{1}H NMR spectrum of **3** in $CDCl_3$.

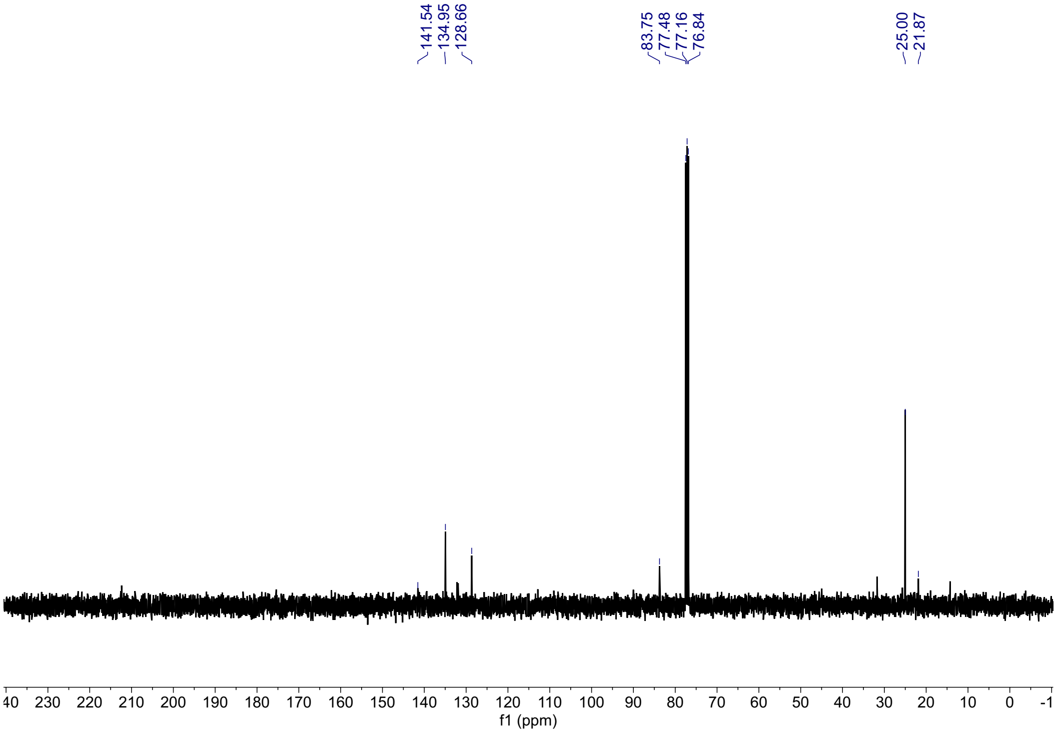

Supplementary Figure 2. ^{13}C NMR spectrum of **3** in $CDCl_3$.

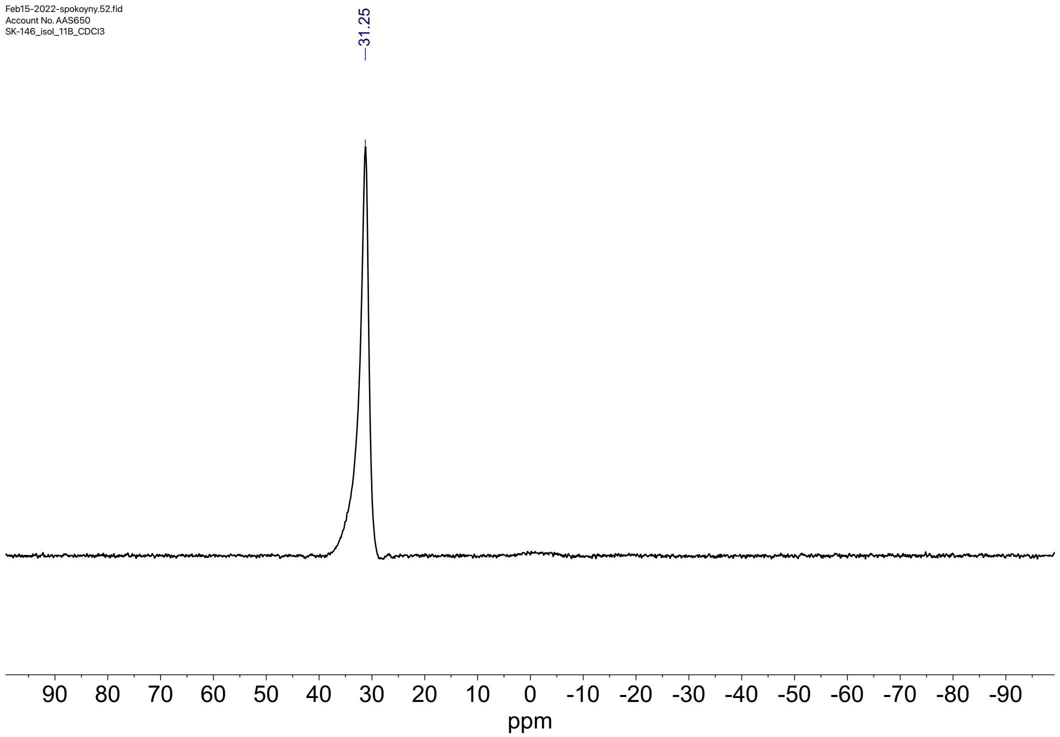

Supplementary Figure 3. ^{11}B NMR spectrum of **3** in $CDCl_3$.

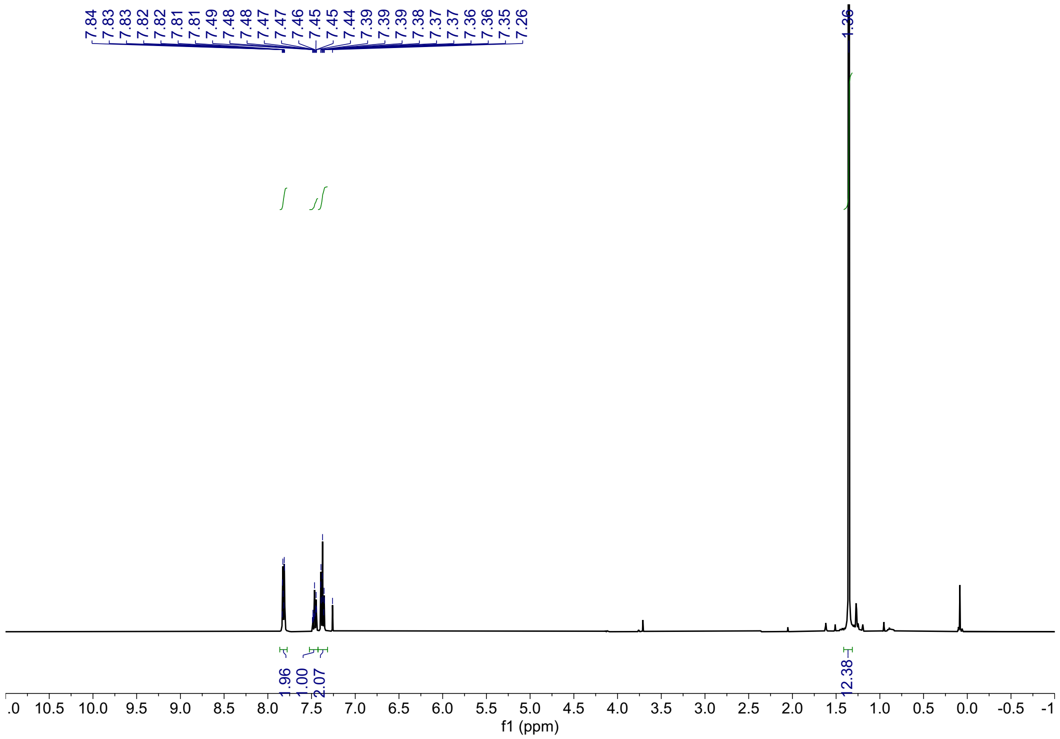

Supplementary Figure 4. ^{1}H NMR spectrum of **4** in $CDCl_3$.

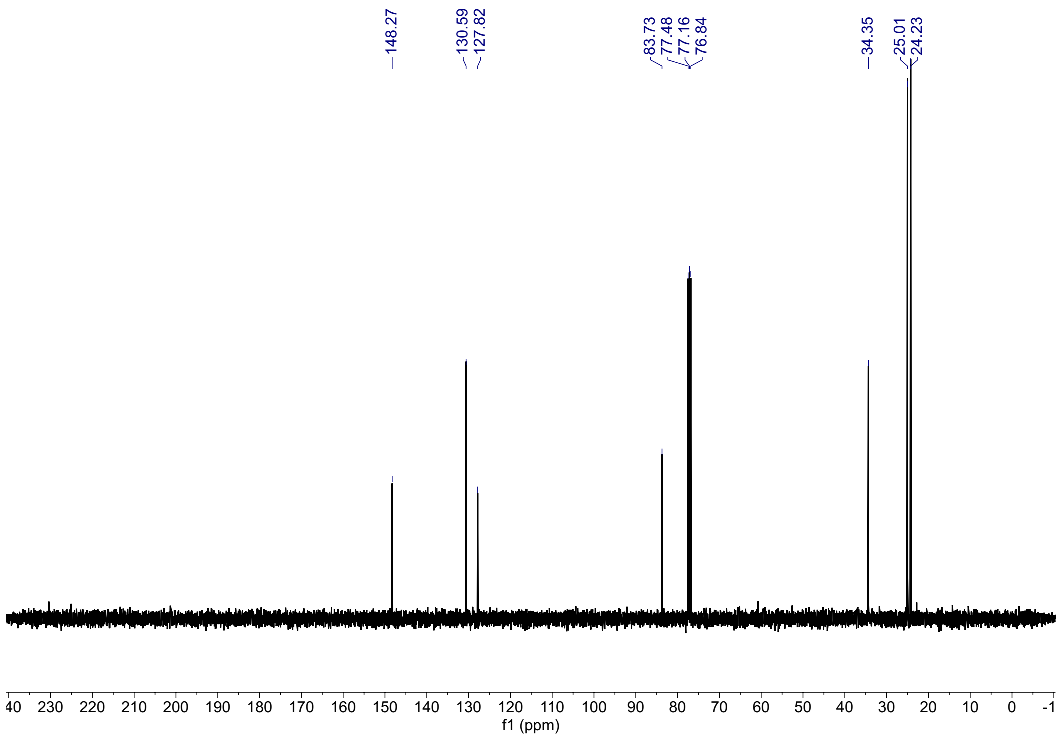

Supplementary Figure 5. ^{13}C NMR spectrum of **4** in $CDCl_3$.

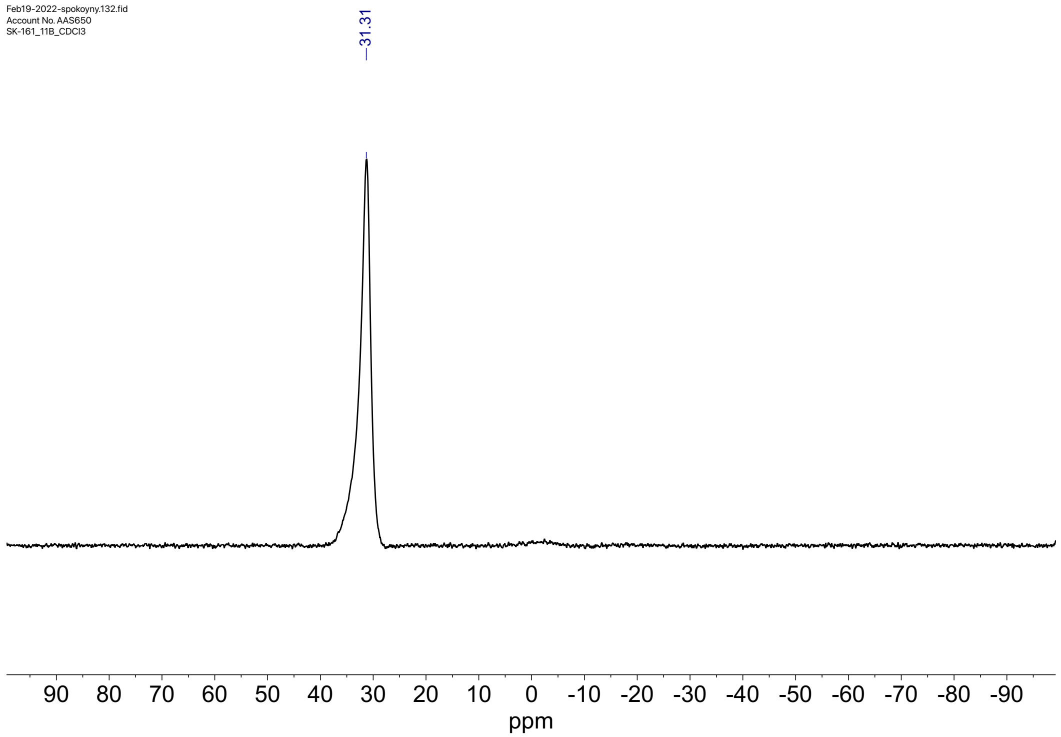

Supplementary Figure 6. ^{11}B NMR spectrum of **4** in $CDCl_3$.

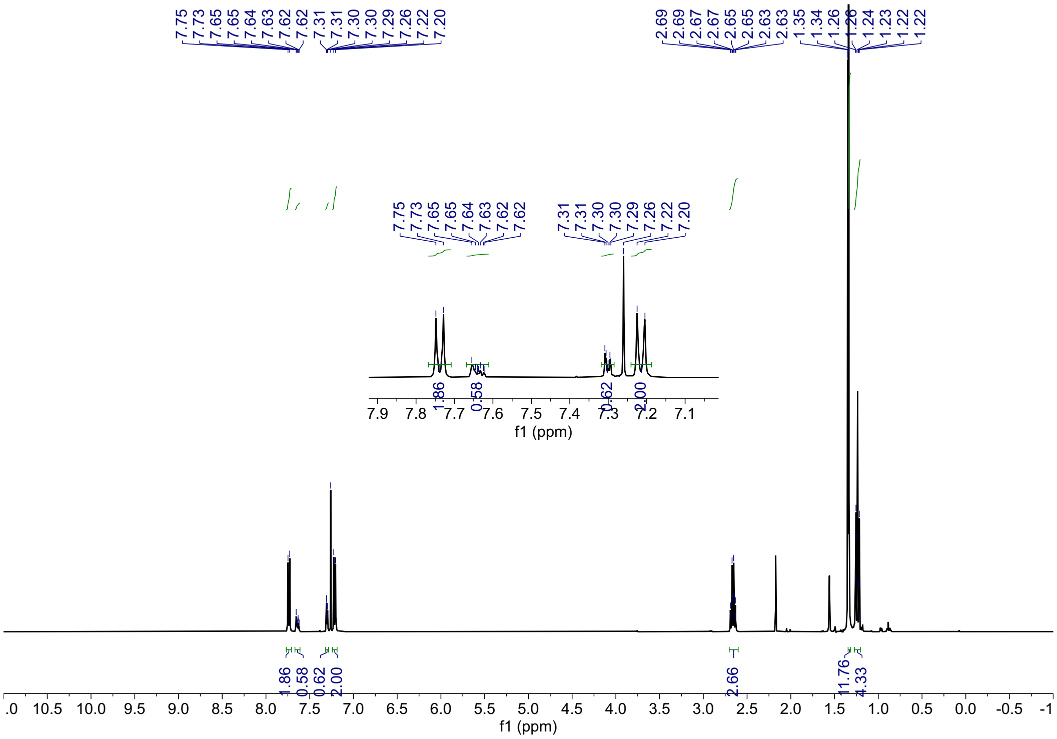

Supplementary Figure 7. ^{1}H NMR spectrum of **5** in $CDCl_3$.

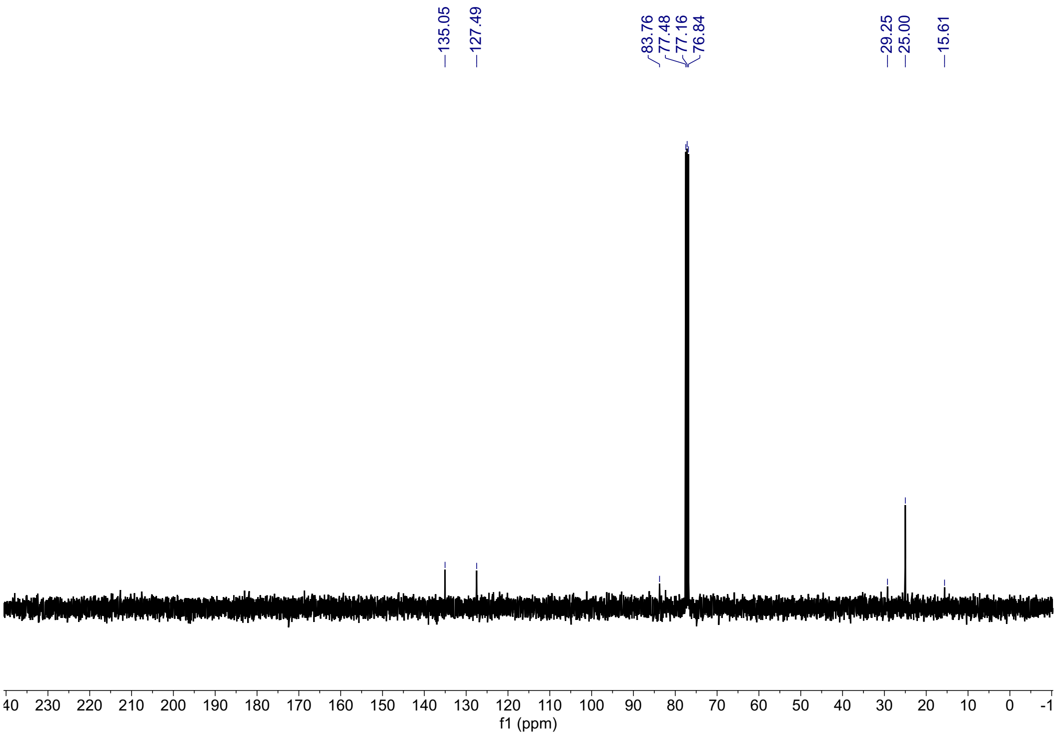

Supplementary Figure 8. ^{13}C NMR spectrum of **5** in $CDCl_3$.

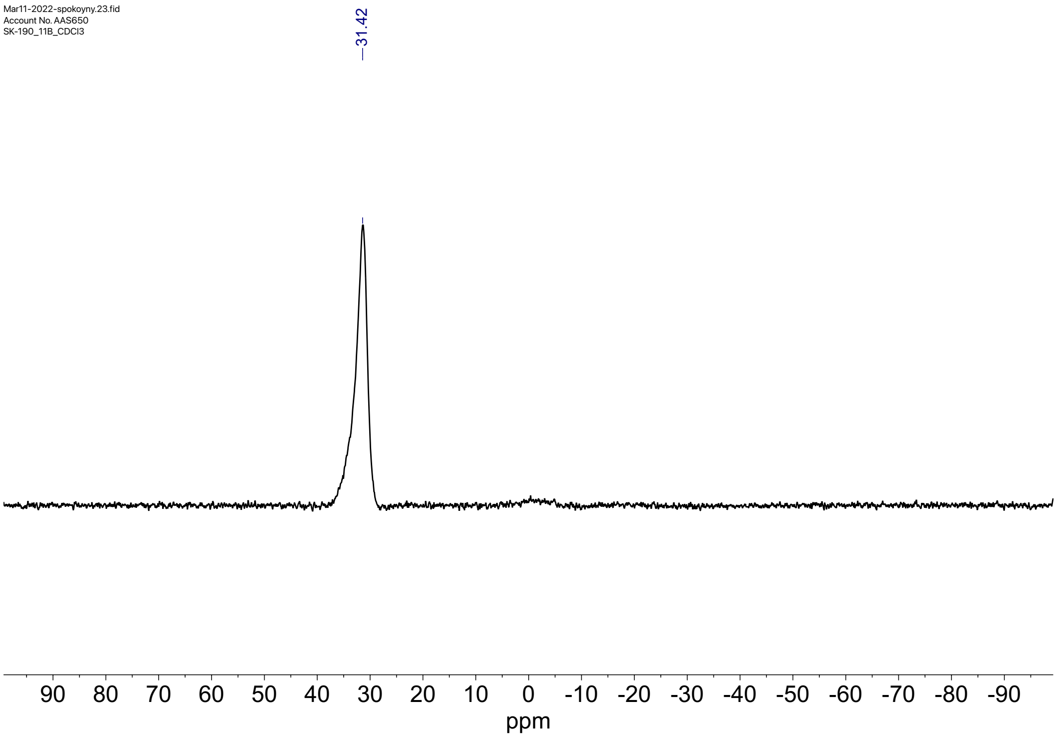

Supplementary Figure 9. ^{11}B NMR spectrum of **5** in $CDCl_3$.

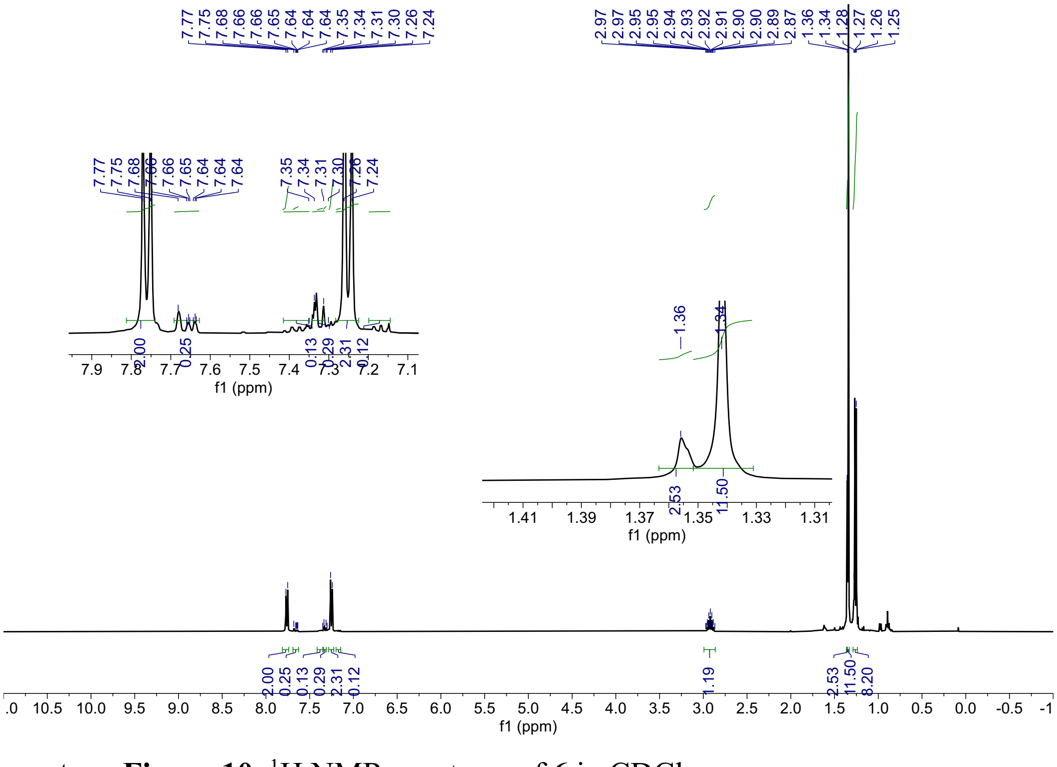

Supplementary Figure 10. ^{1}H NMR spectrum of **6** in $CDCl_3$.

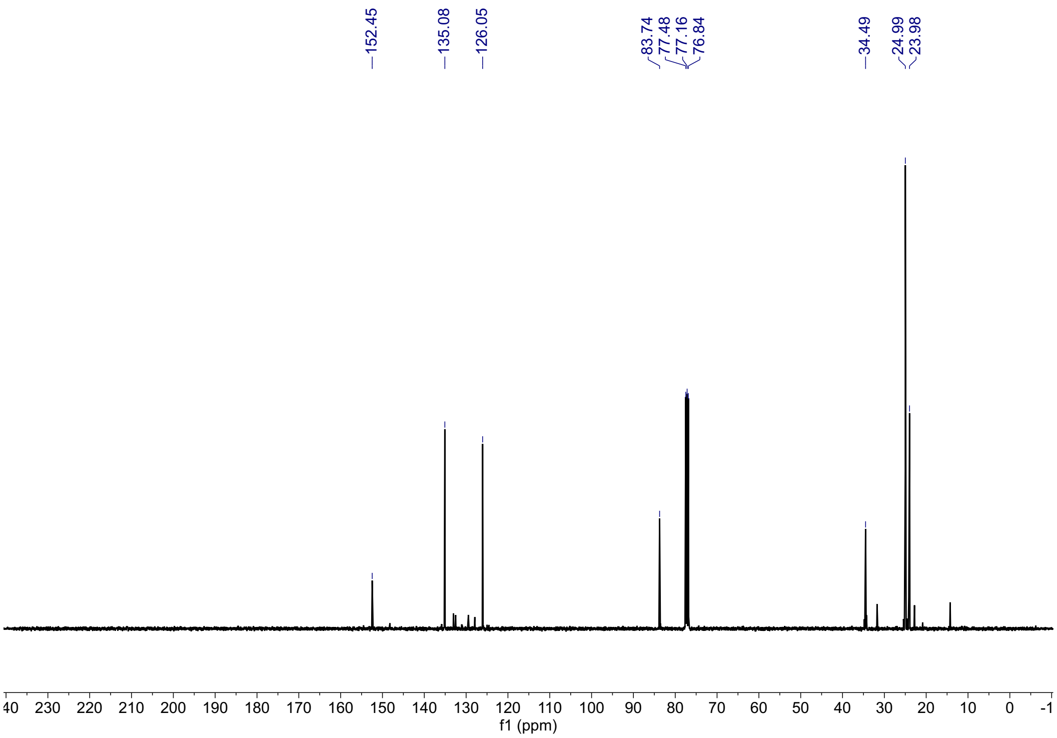

Supplementary Figure 11. ^{13}C NMR spectrum of **6** in $CDCl_3$.

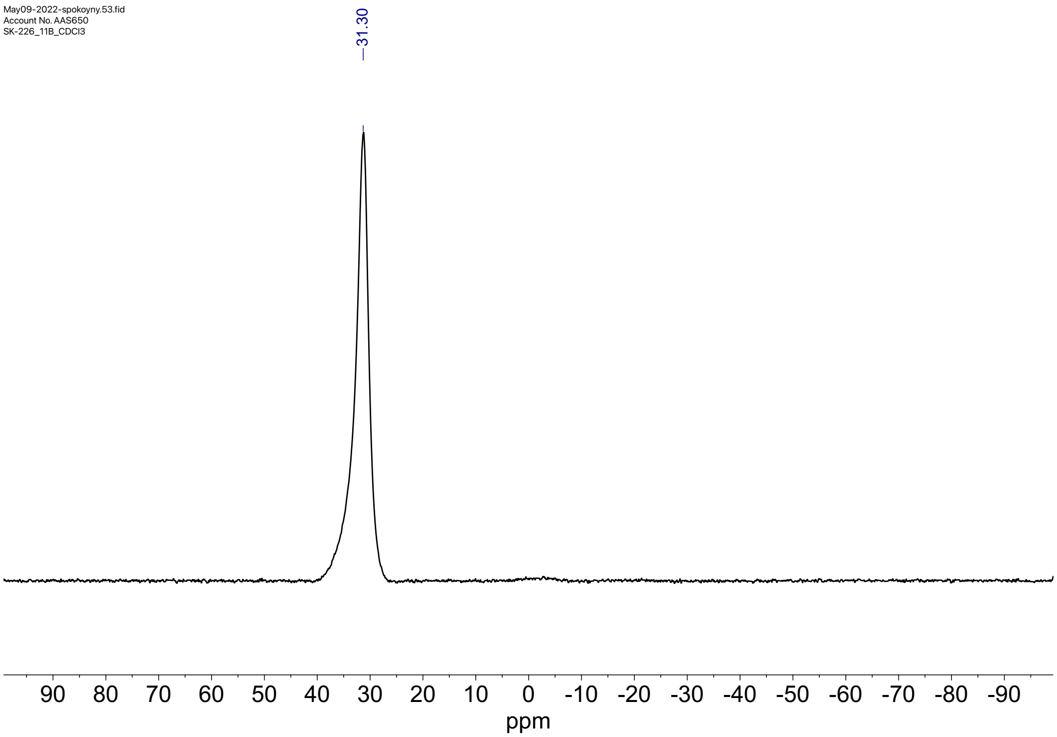

Supplementary Figure 12. ^{11}B NMR spectrum of **6** in $CDCl_3$.

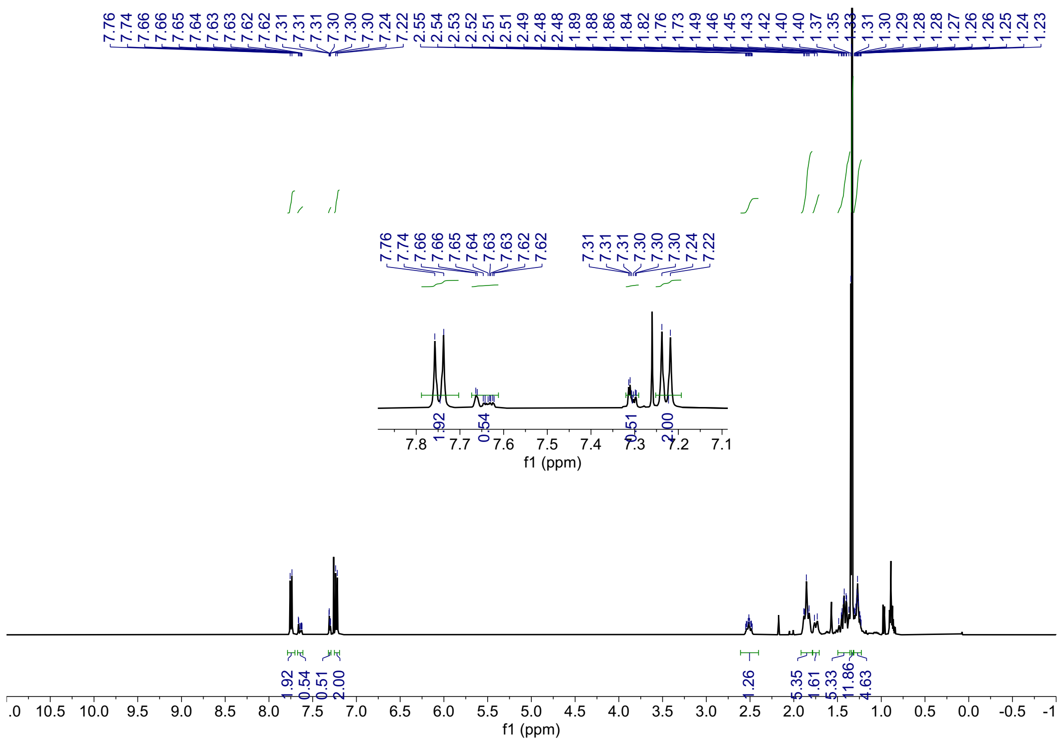

Supplementary Figure 13. ^{1}H NMR spectrum of **7** in $CDCl_3$.

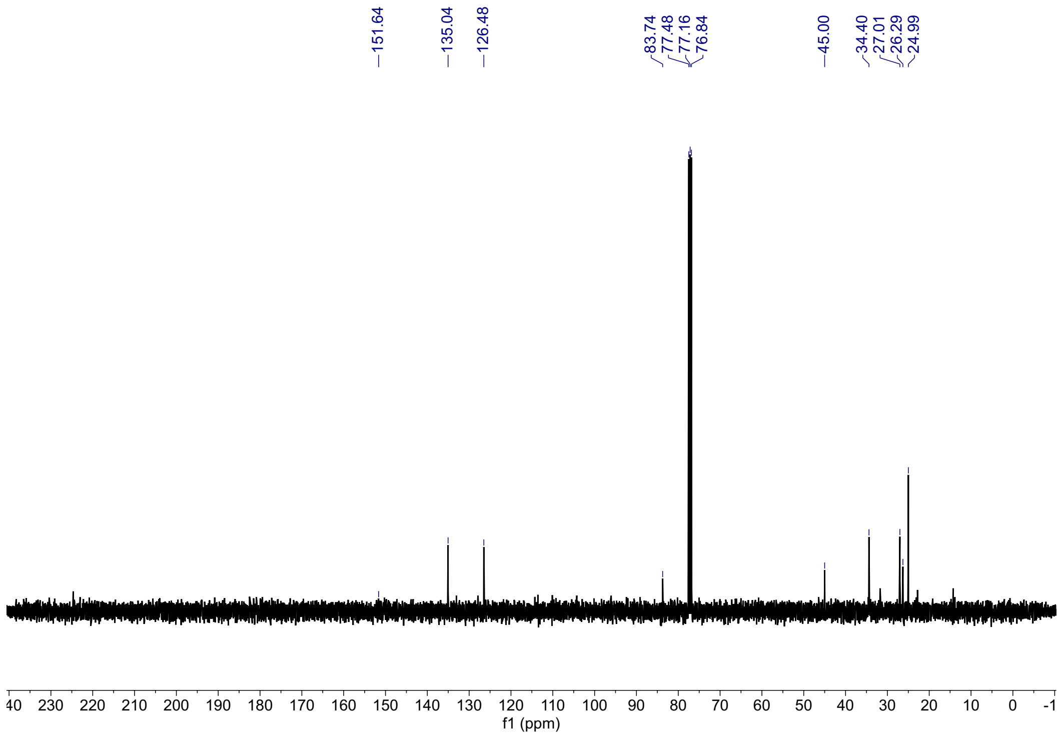

Supplementary Figure 14. ^{13}C NMR spectrum of **7** in $CDCl_3$.

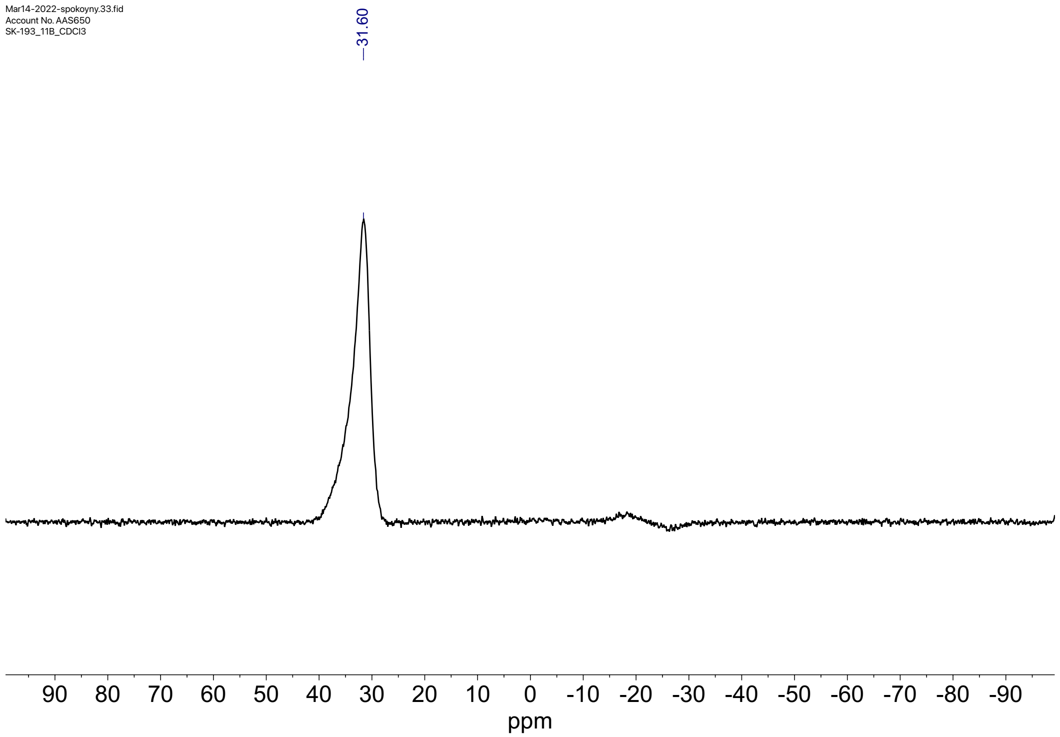

Supplementary Figure 15. ^{11}B NMR spectrum of **7** in $CDCl_3$.

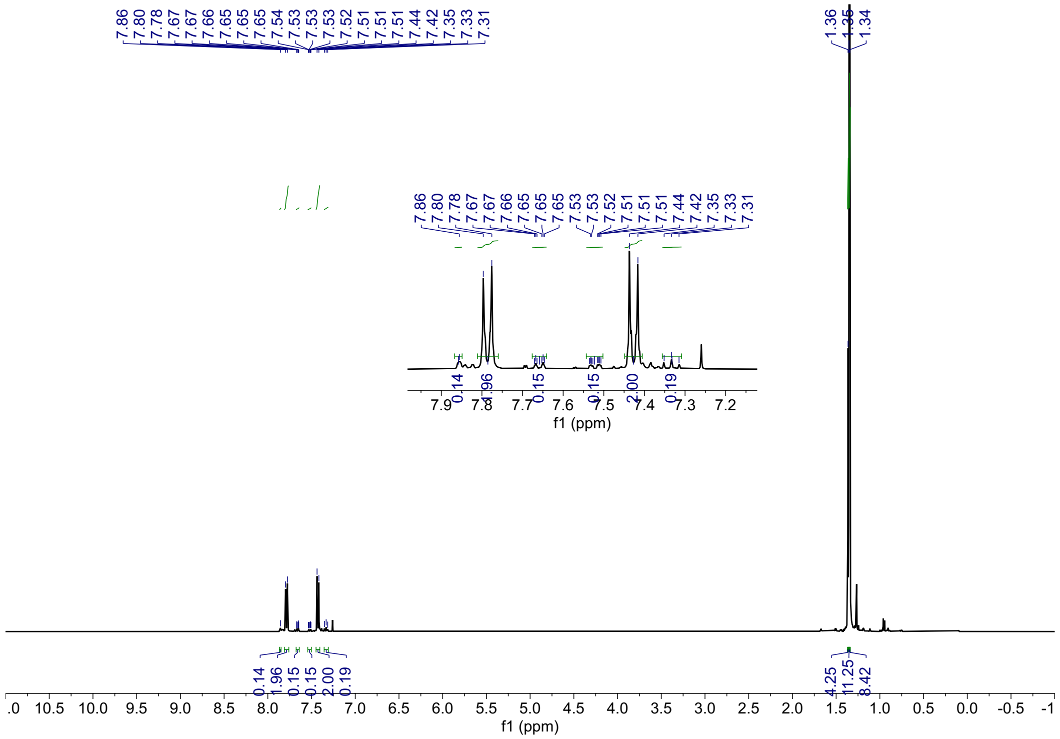

Supplementary Figure 16. ^{1}H NMR spectrum of **8** in $CDCl_3$.

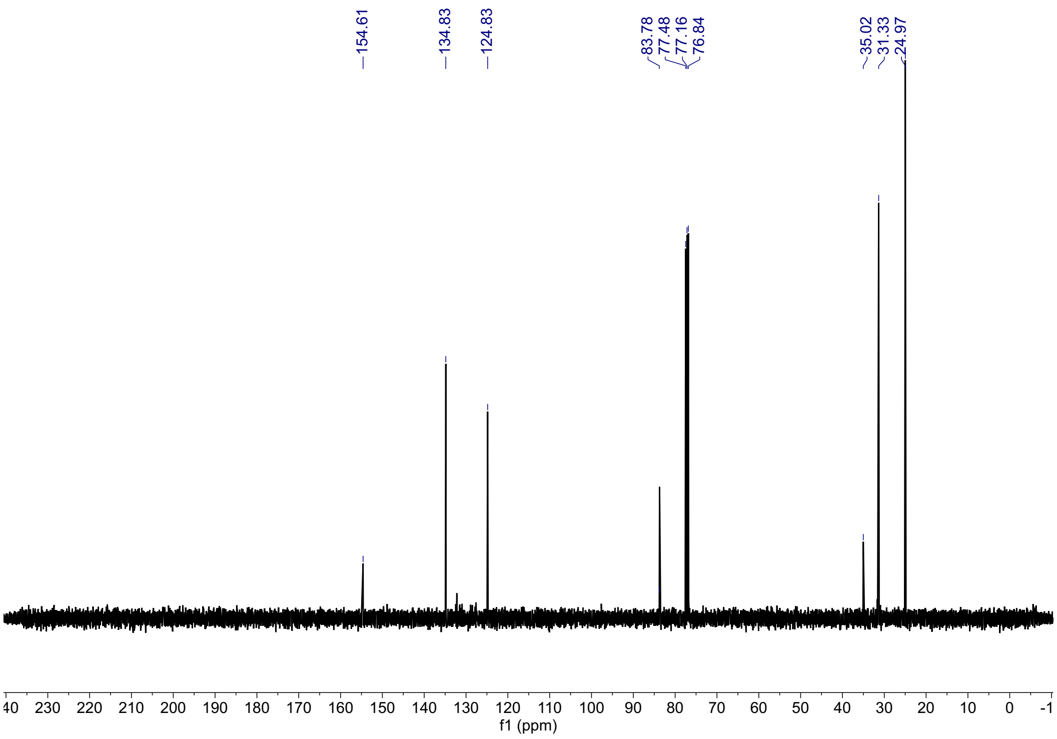

Supplementary Figure 17. ^{13}C NMR spectrum of **8** in $CDCl_3$.

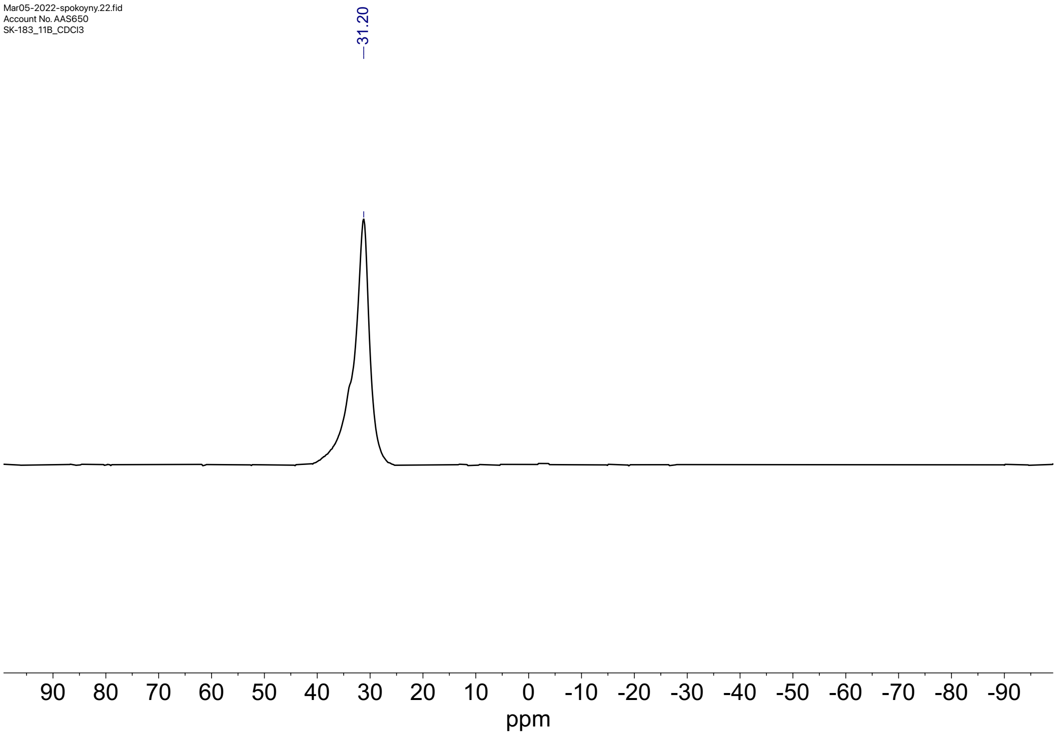

Supplementary Figure 18. ^{11}B NMR spectrum of **8** in $CDCl_3$.

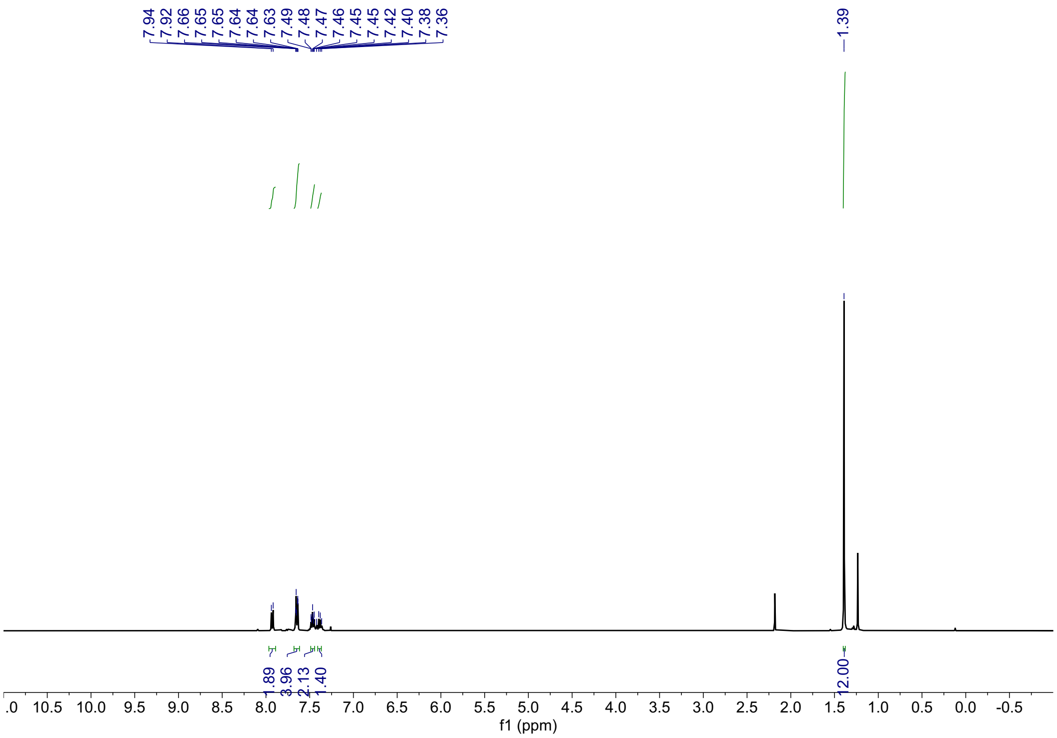

Supplementary Figure 19. ^{1}H NMR spectrum of **9** in $CDCl_3$.

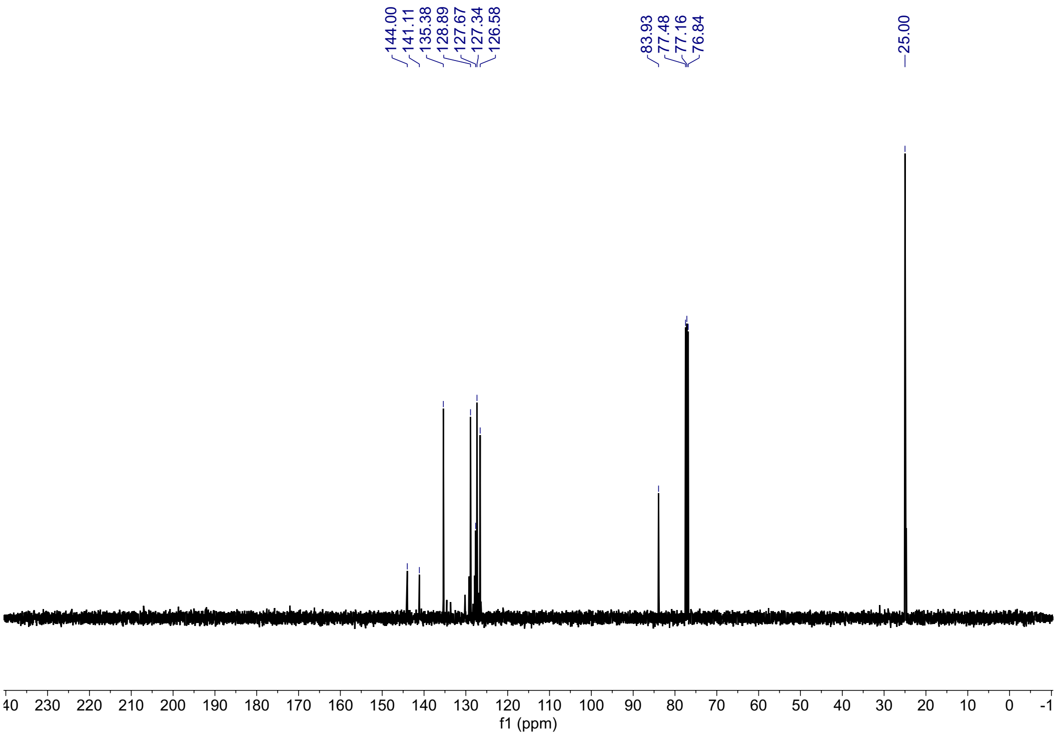

Supplementary Figure 20. ^{13}C NMR spectrum of **9** in $CDCl_3$.

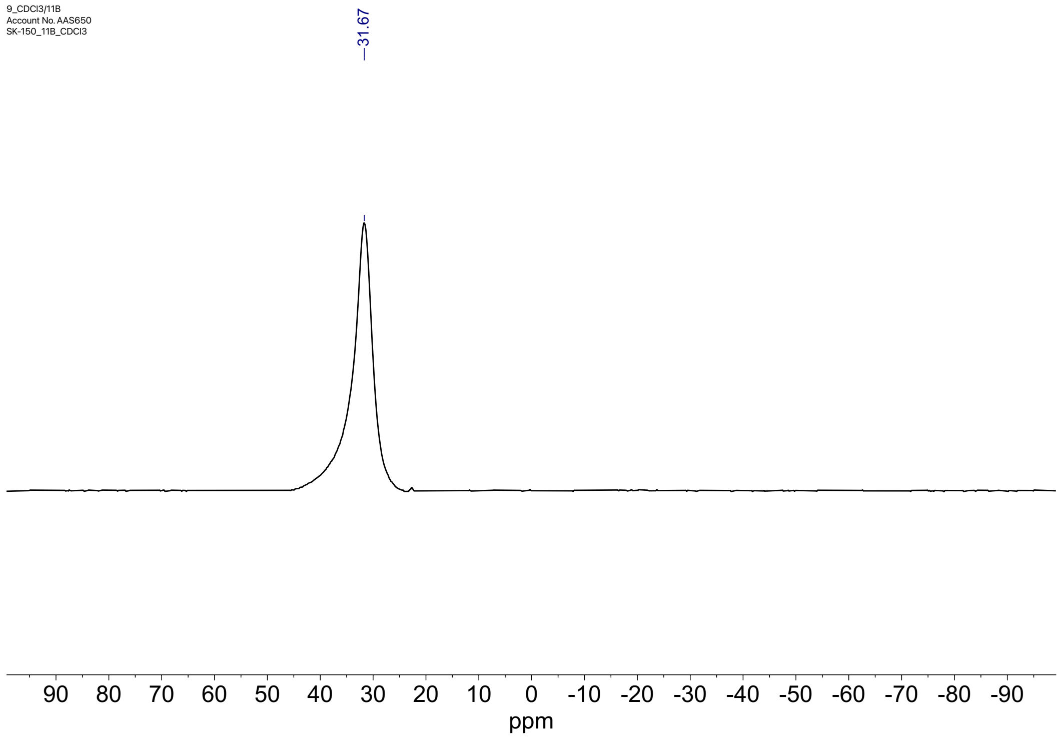

Supplementary Figure 21. ^{11}B NMR spectrum of **9** in $CDCl_3$.

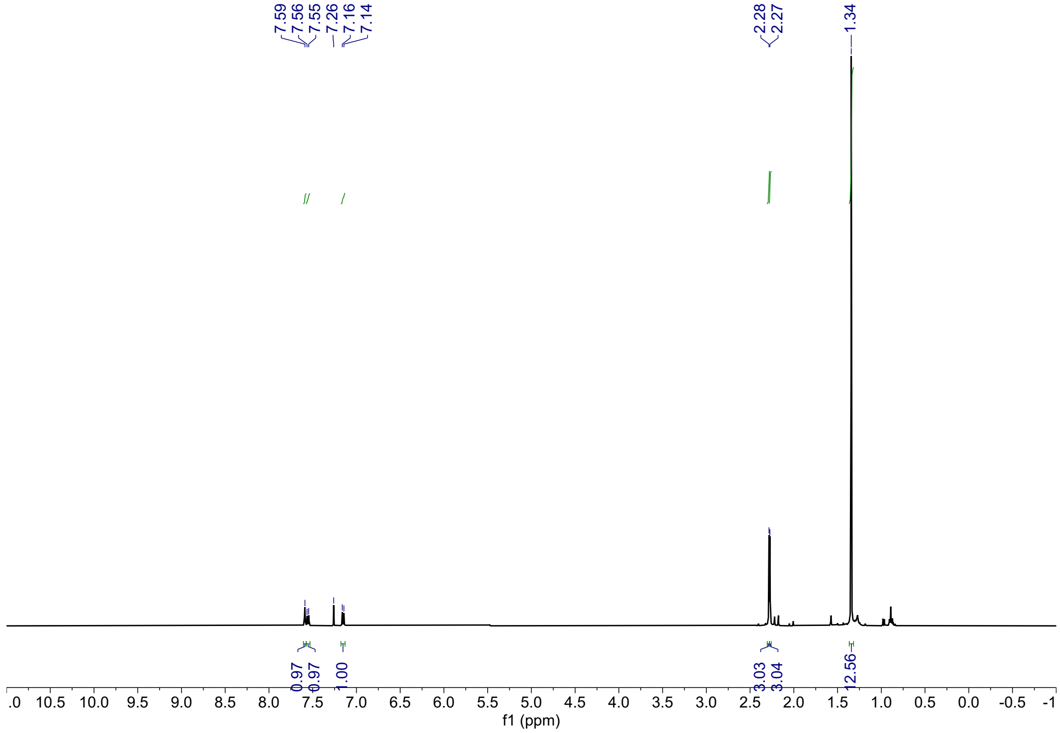

Supplementary Figure 22. ^{1}H NMR spectrum of **10** in $CDCl_3$.

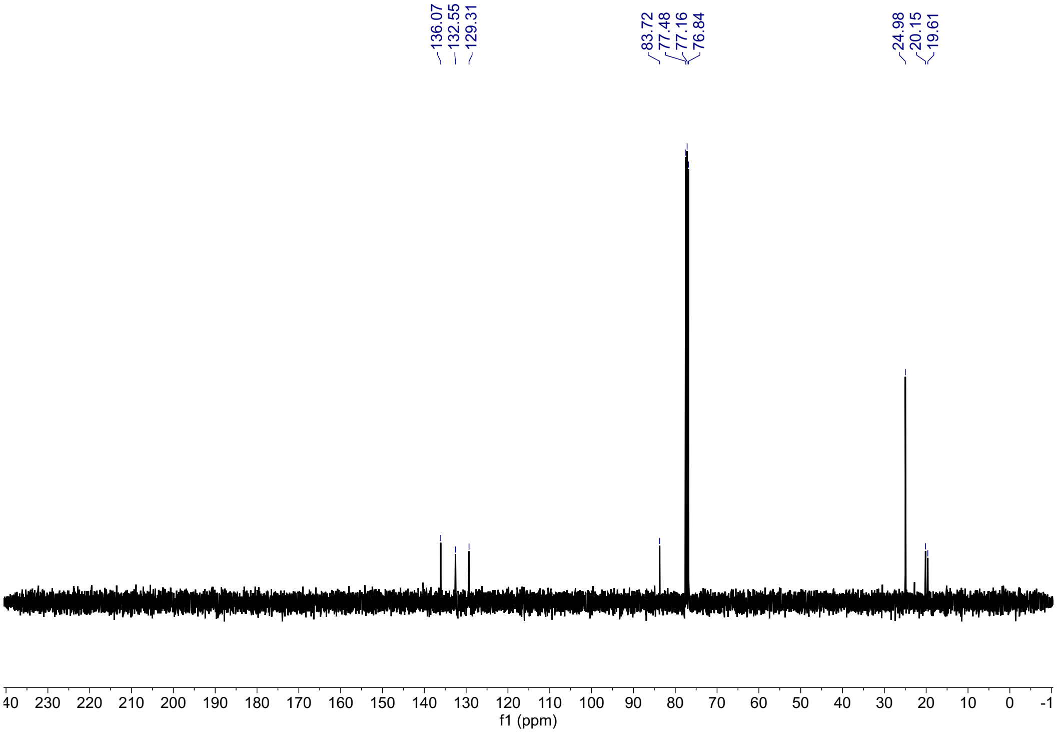

Supplementary Figure 23. ^{13}C NMR spectrum of **10** in $CDCl_3$.

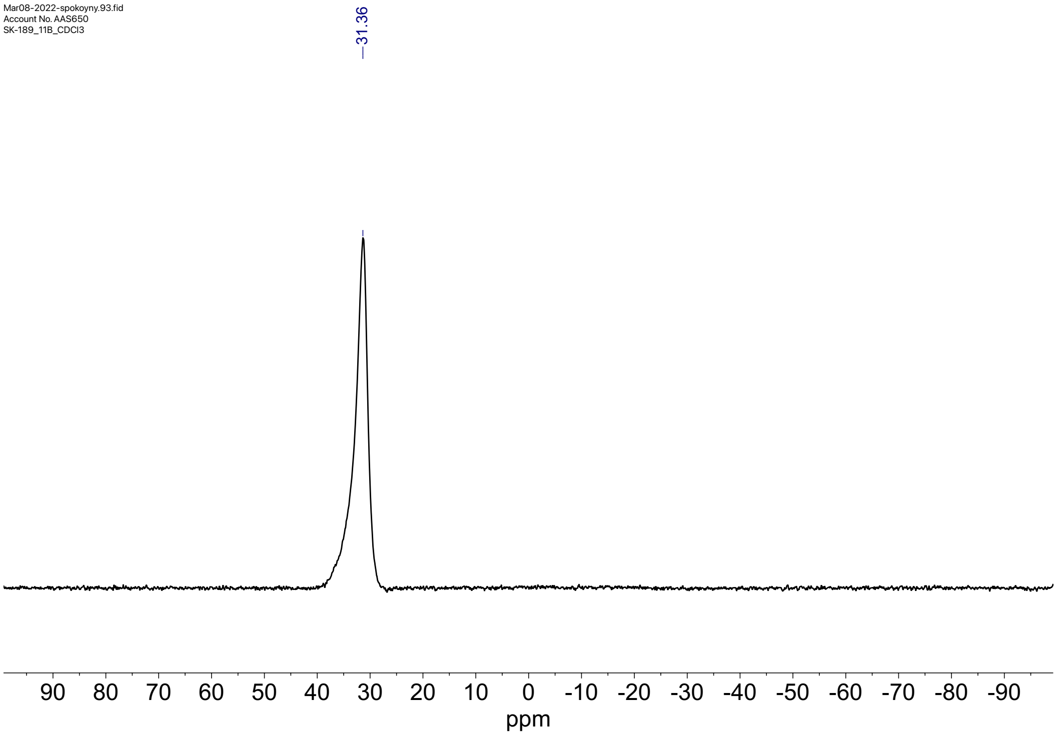

Supplementary Figure 24. ^{11}B NMR spectrum of **10** in $CDCl_3$.

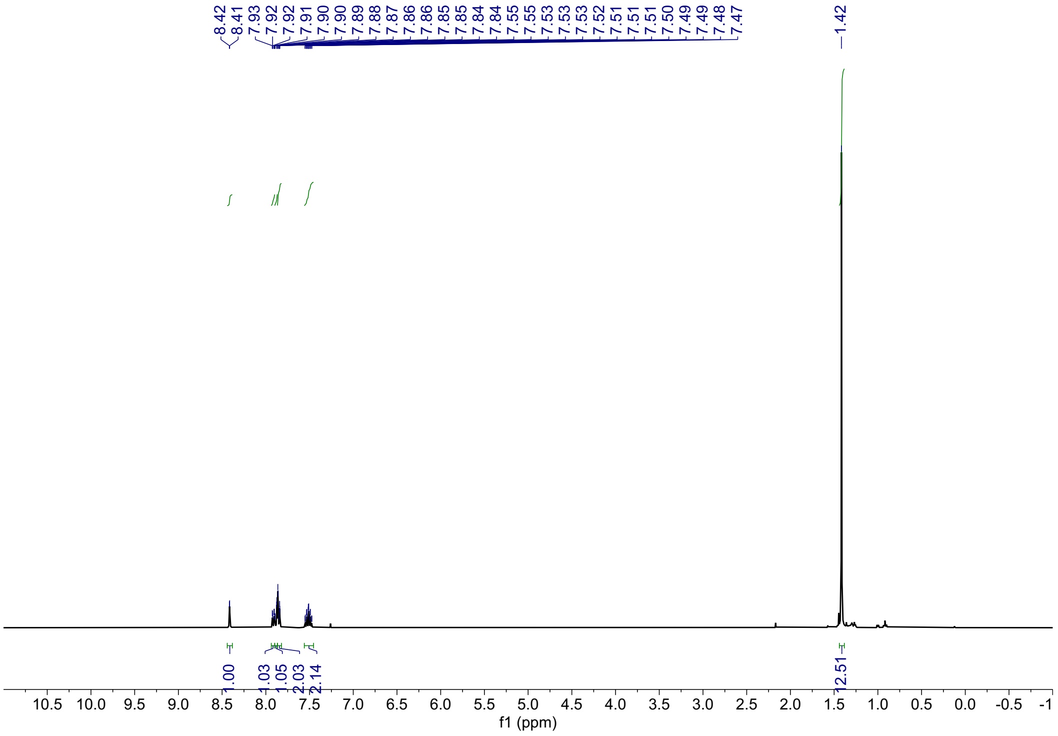

Supplementary Figure 25. ^{1}H NMR spectrum of **11** in $CDCl_3$.

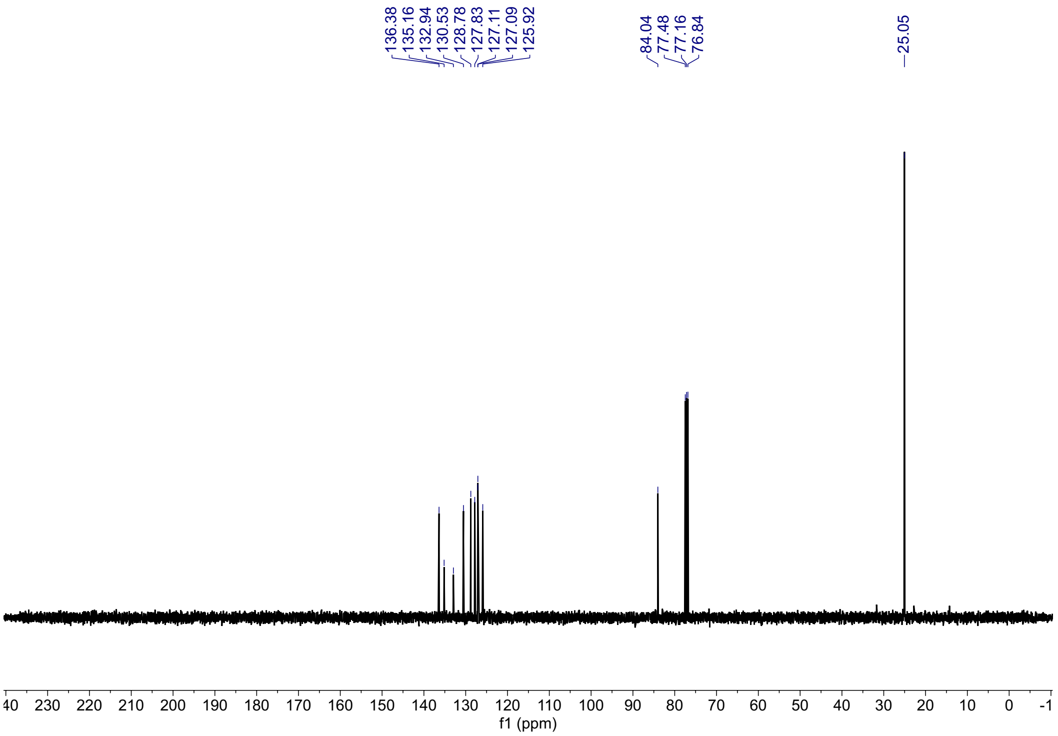

Supplementary Figure 26. ^{13}C NMR spectrum of **11** in $CDCl_3$.

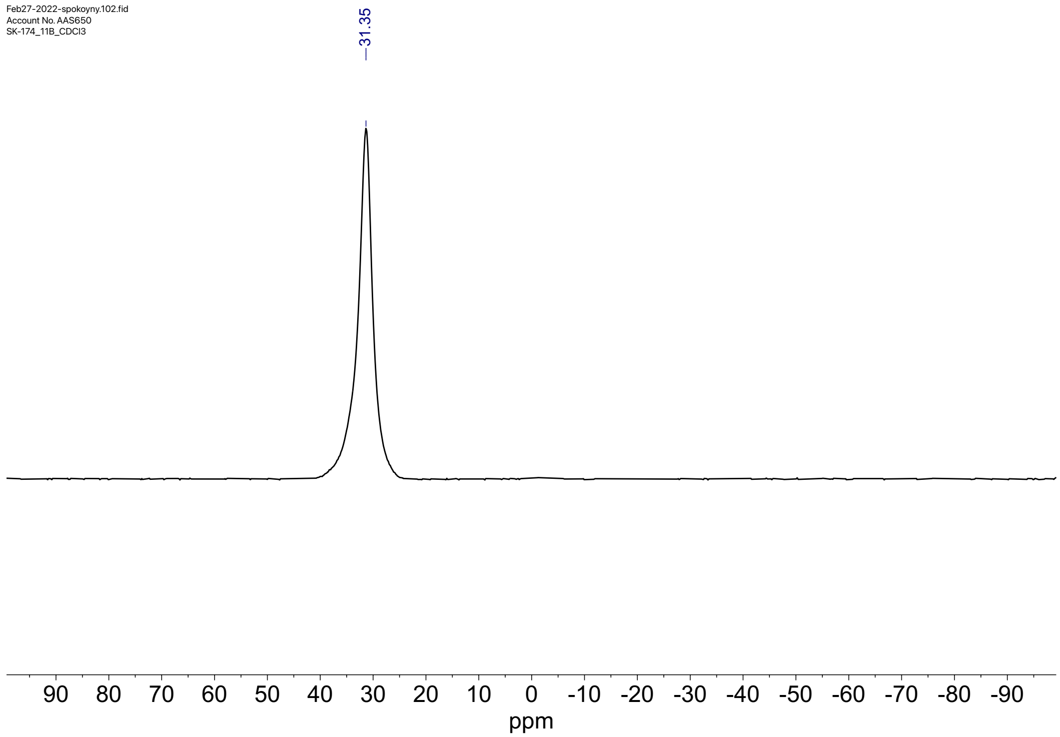

Supplementary Figure 27. ^{11}B NMR spectrum of **11** in $CDCl_3$.

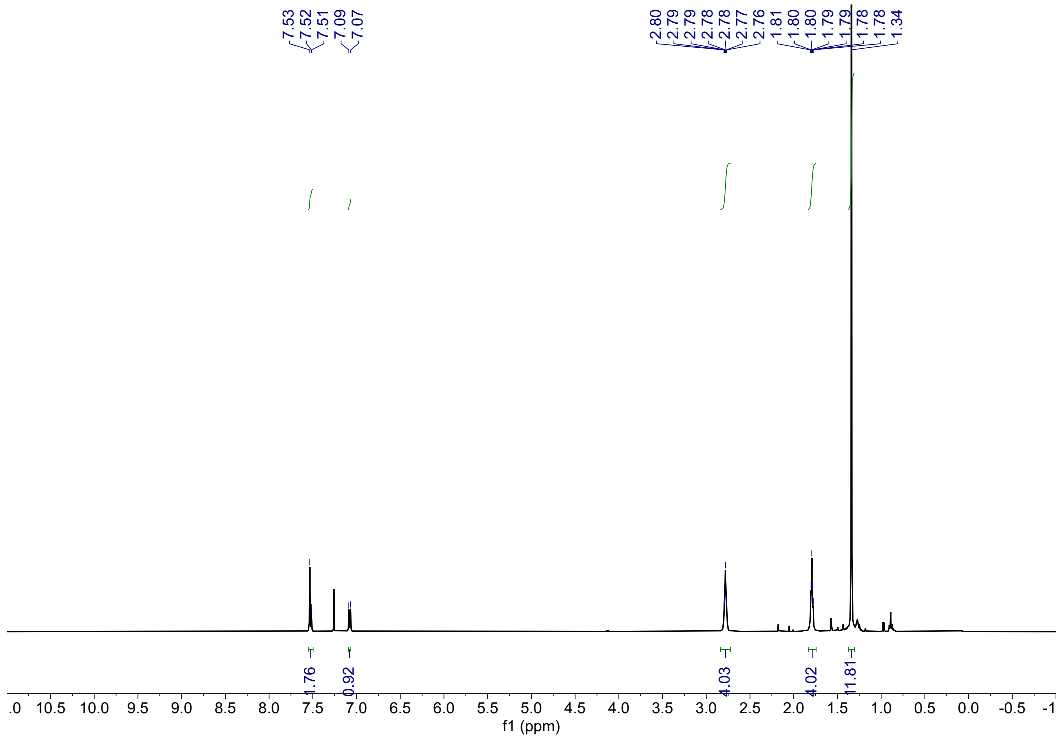

Supplementary Figure 28. ^{1}H NMR spectrum of **12** in $CDCl_3$.

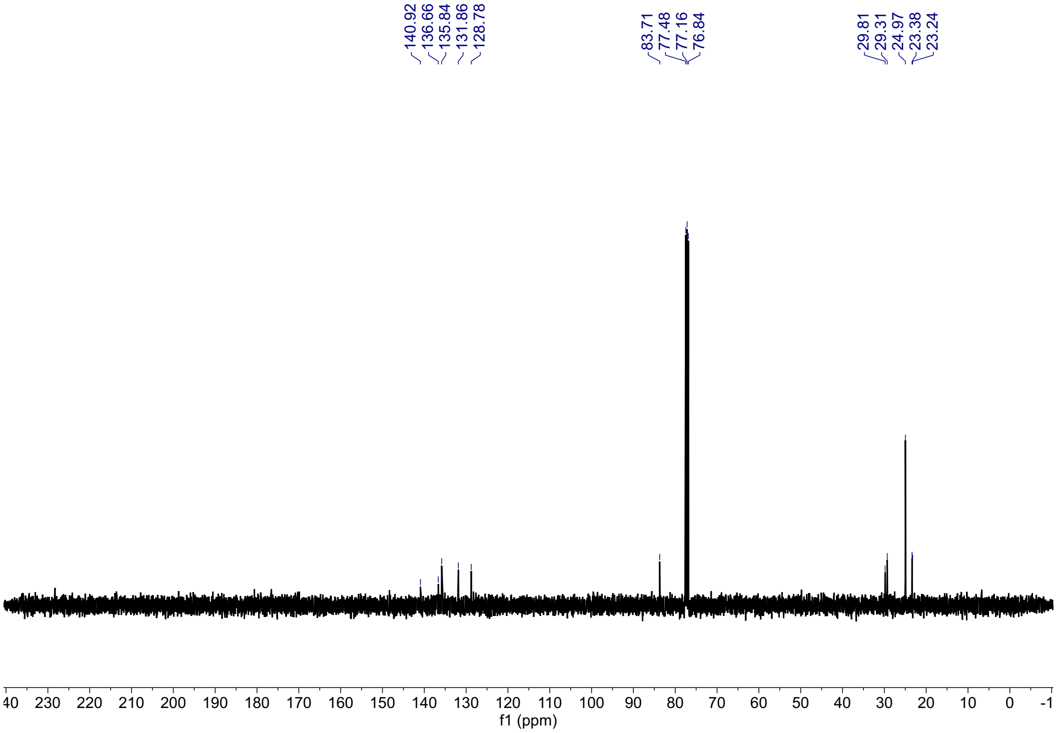

Supplementary Figure 29. ^{13}C NMR spectrum of **12** in $CDCl_3$.

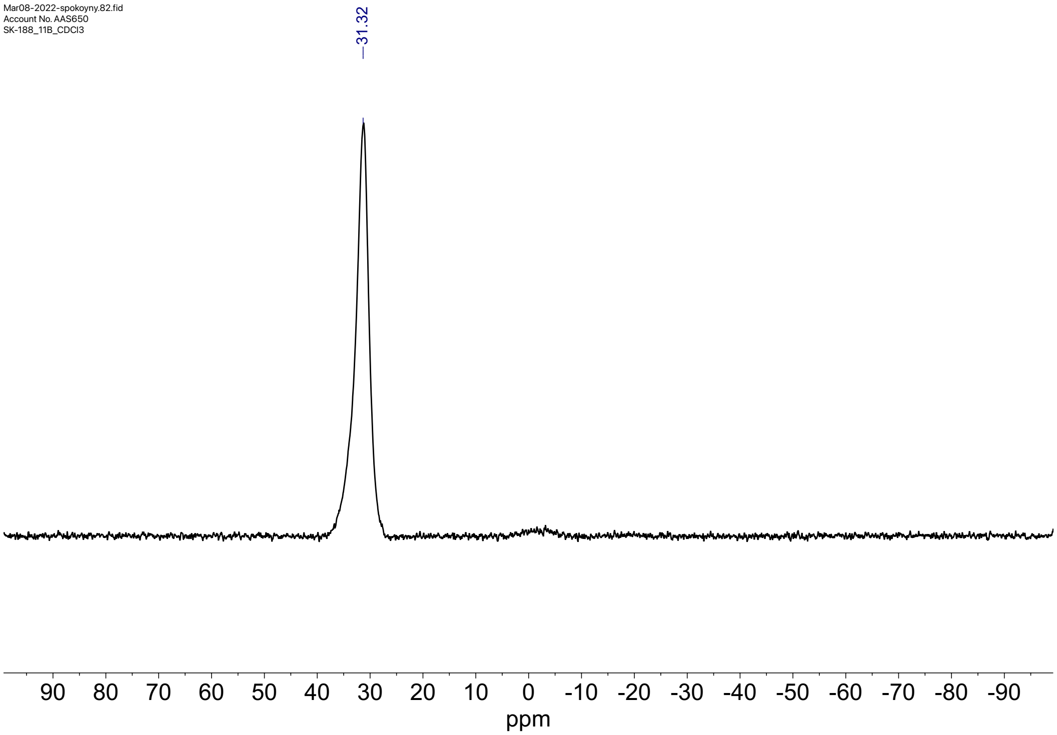

Supplementary Figure 30. ^{11}B NMR spectrum of **12** in $CDCl_3$.

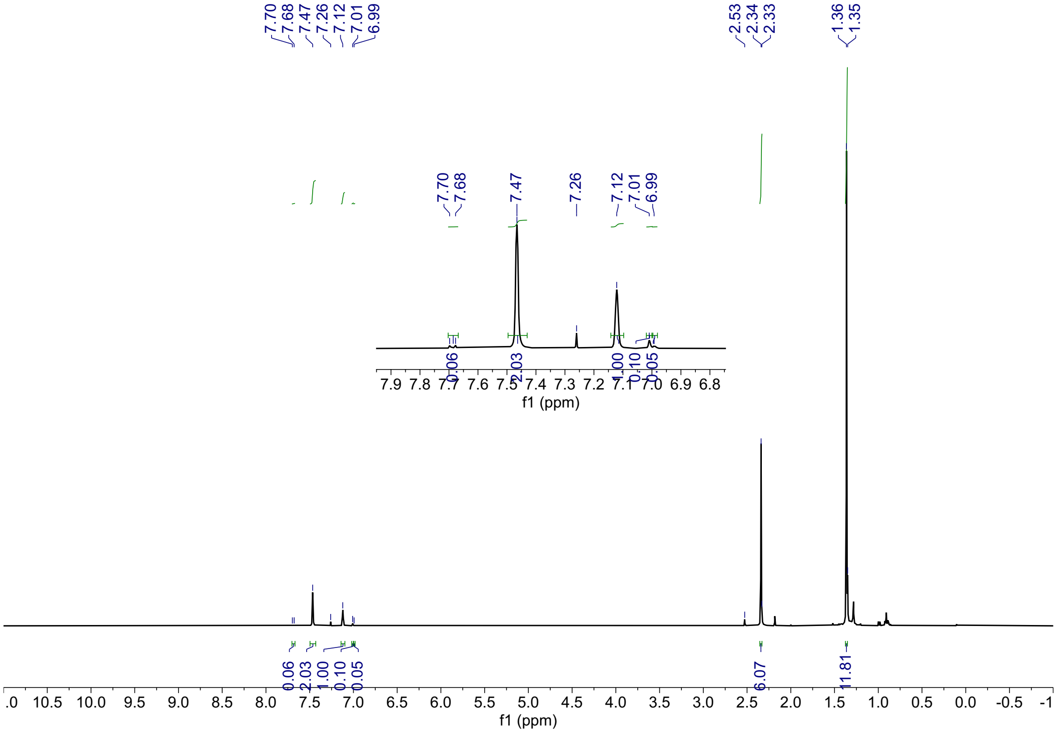

Supplementary Figure 31. ^{1}H NMR spectrum of **13** in $CDCl_3$.

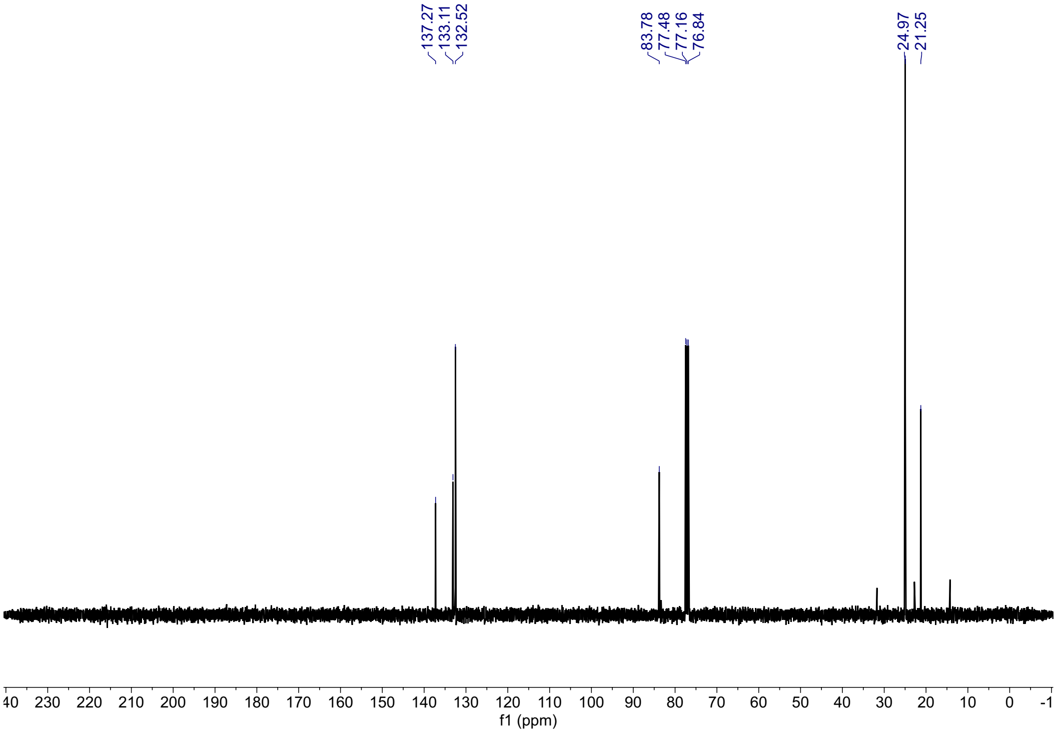

Supplementary Figure 32. ^{13}C NMR spectrum of **13** in $CDCl_3$.

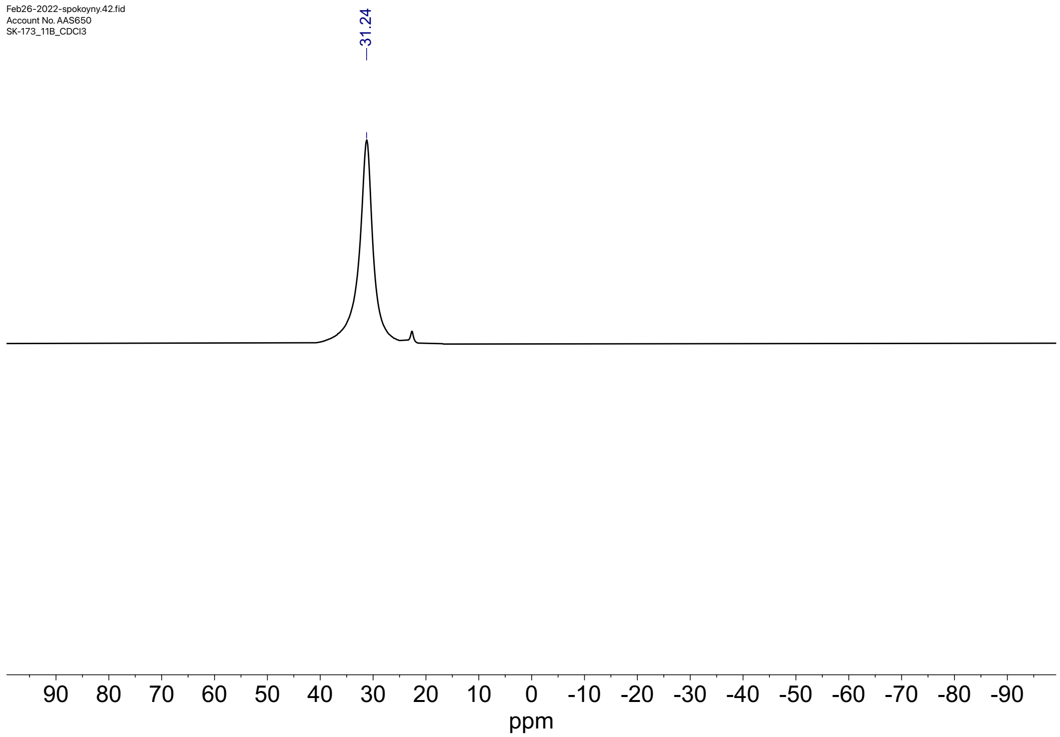

Supplementary Figure 33. ^{11}B NMR spectrum of **13** in $CDCl_3$.

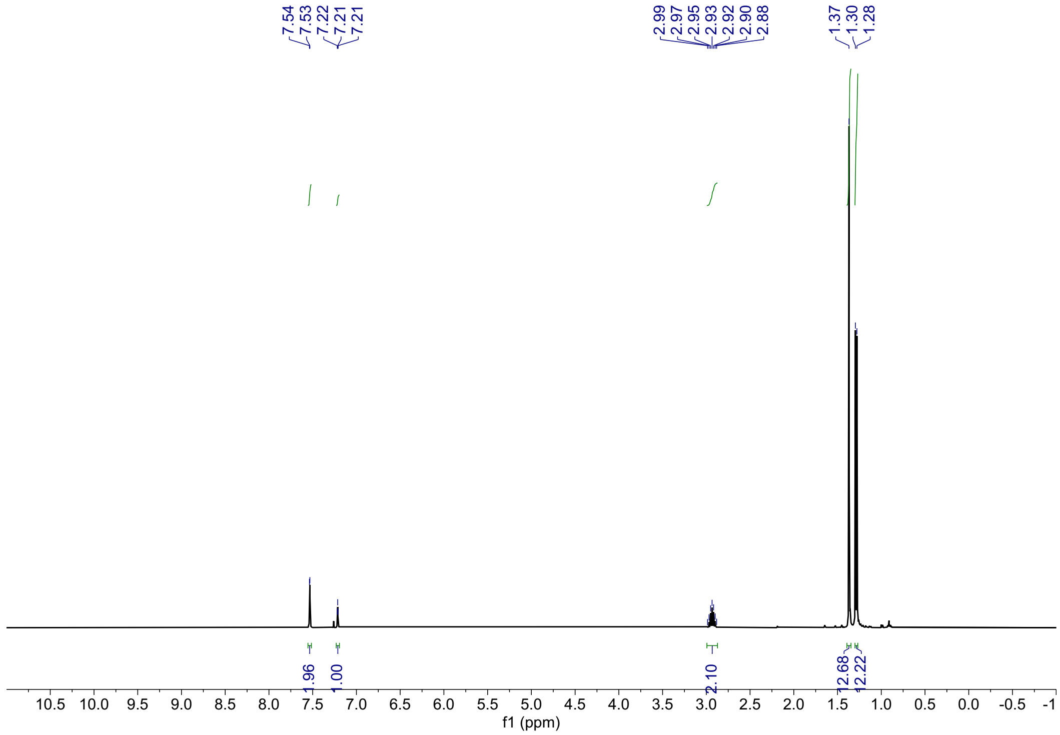

Supplementary Figure 34. ^{1}H NMR spectrum of **14** in $CDCl_3$.

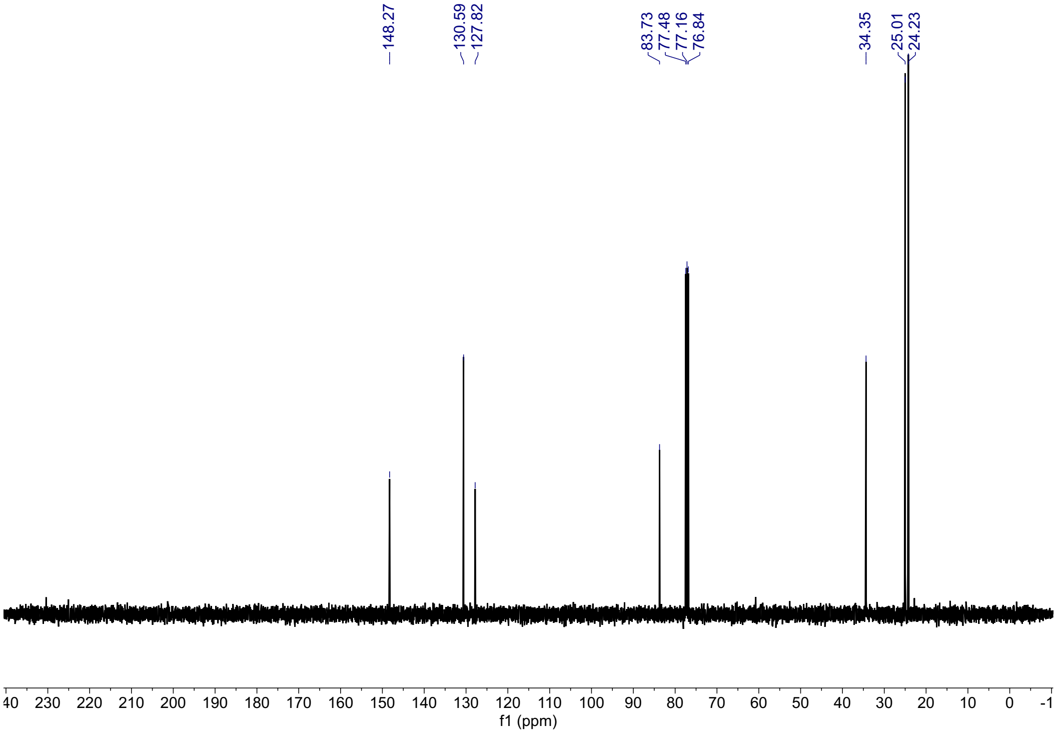

Supplementary Figure 35. ^{13}C NMR spectrum of **14** in $CDCl_3$.

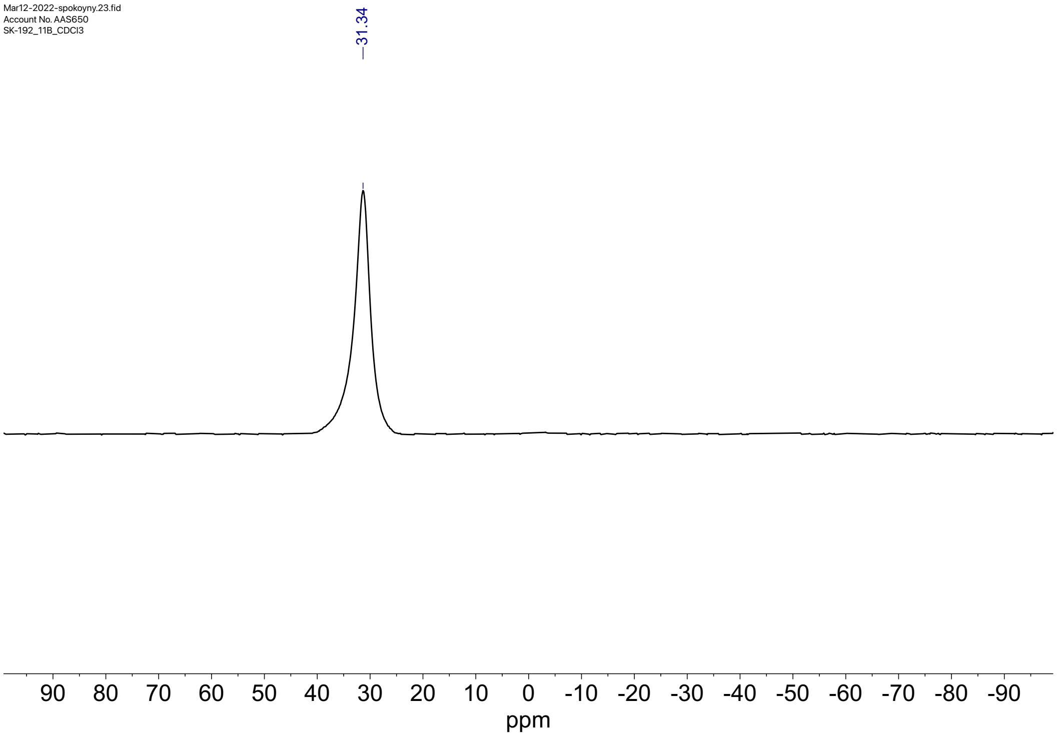

Supplementary Figure 36. ^{11}B NMR spectrum of **14** in $CDCl_3$.

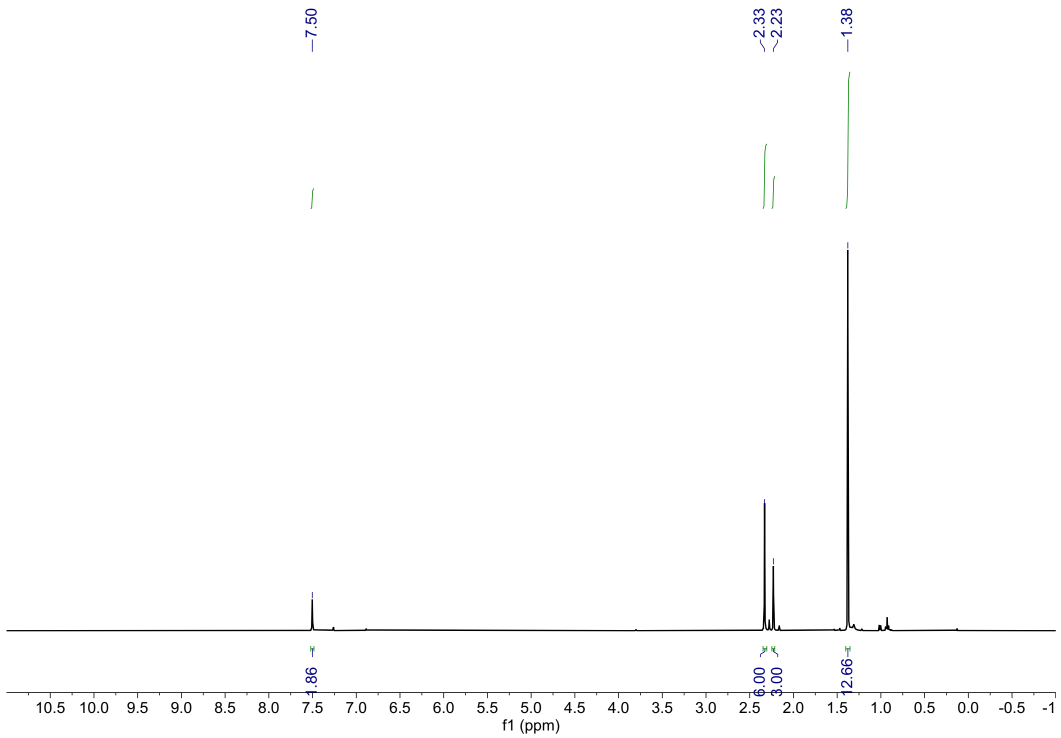

Supplementary Figure 37. ^{1}H NMR spectrum of **15** in $CDCl_3$.

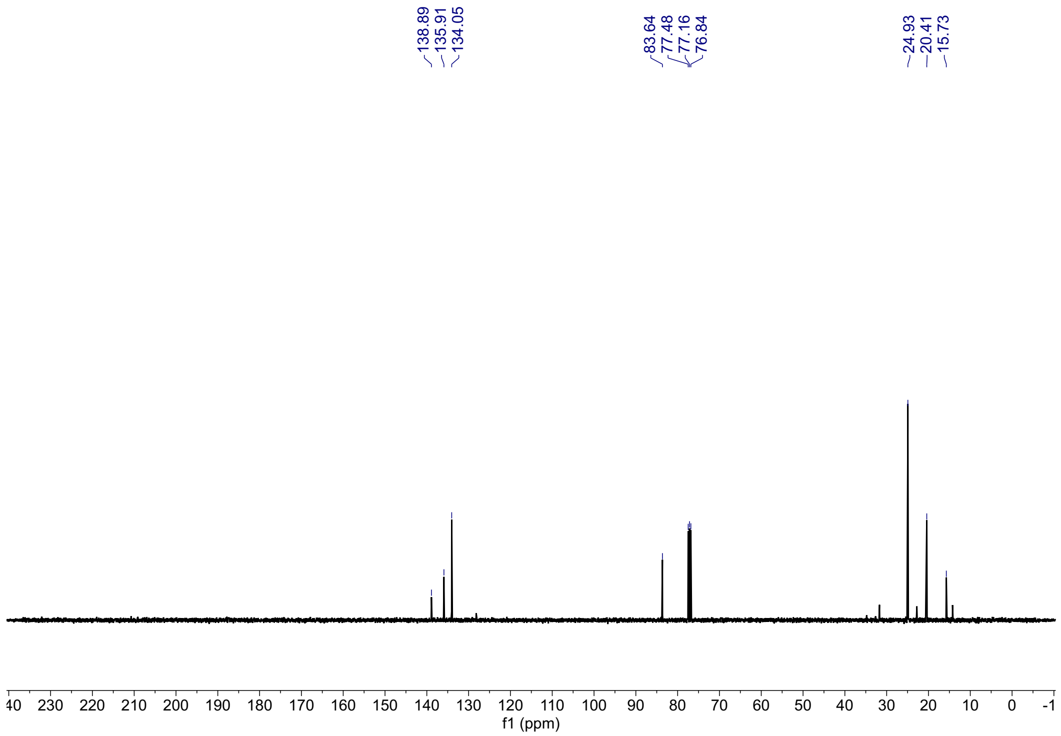

Supplementary Figure 38. ^{13}C NMR spectrum of **15** in $CDCl_3$.

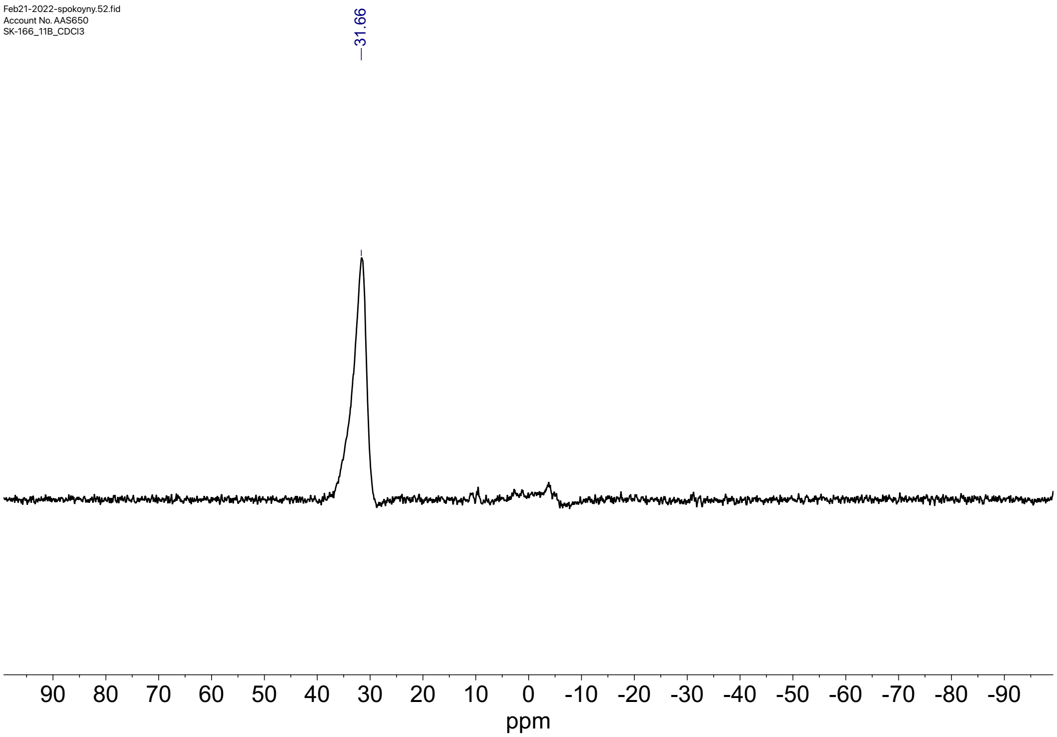

Supplementary Figure 39. ^{11}B NMR spectrum of **15** in $CDCl_3$.

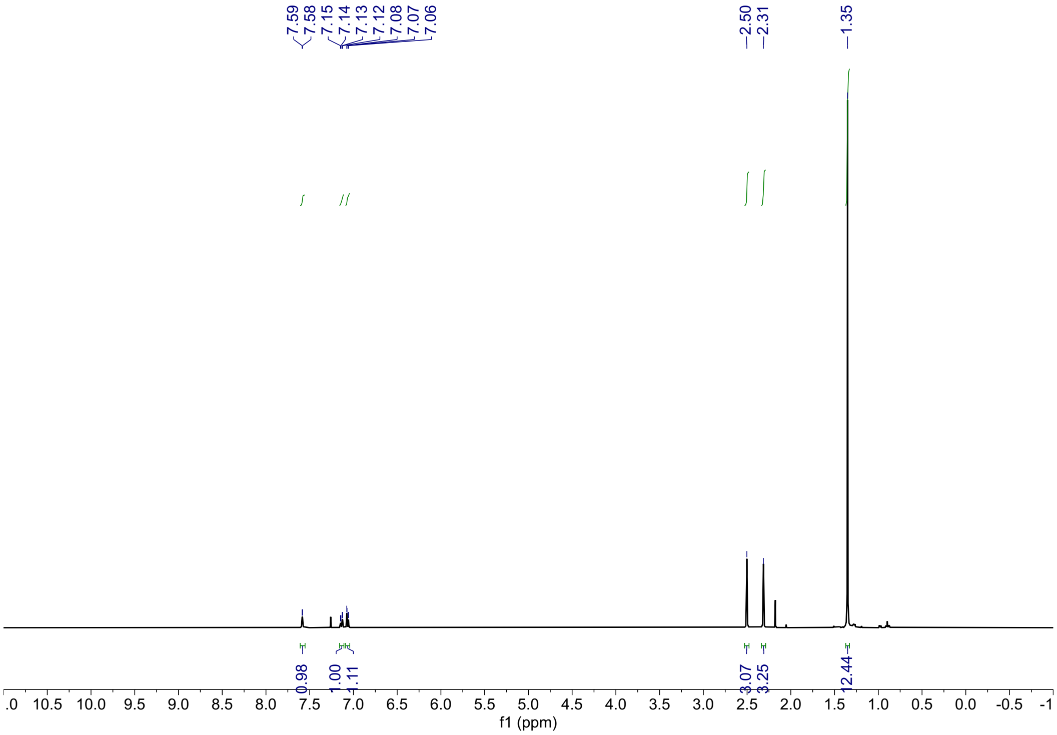

Supplementary Figure 40. ^{1}H NMR spectrum of **16** in $CDCl_3$.

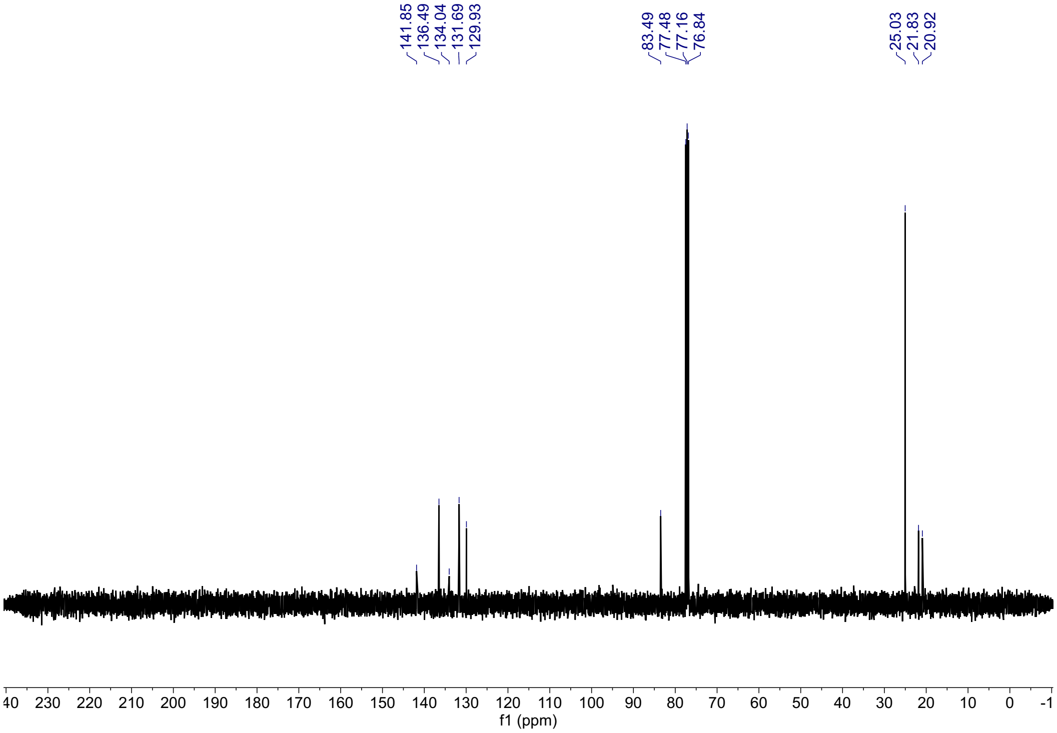

Supplementary Figure 41. ^{13}C NMR spectrum of **16** in $CDCl_3$.

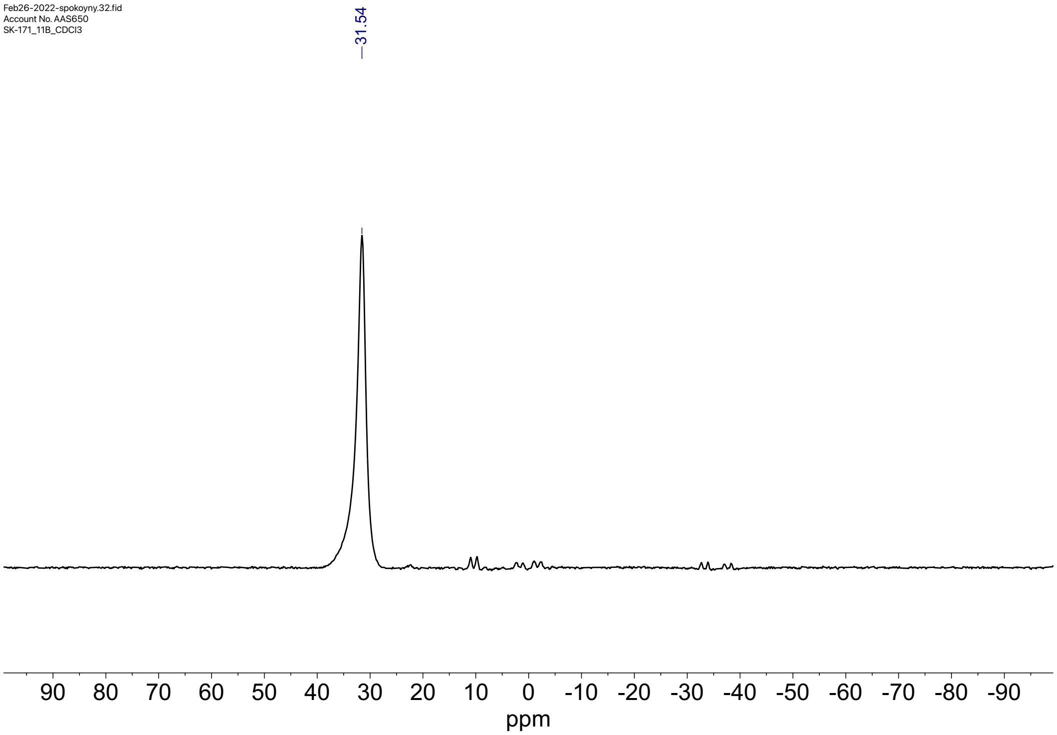

Supplementary Figure 42. ^{11}B NMR spectrum of **16** in $CDCl_3$.

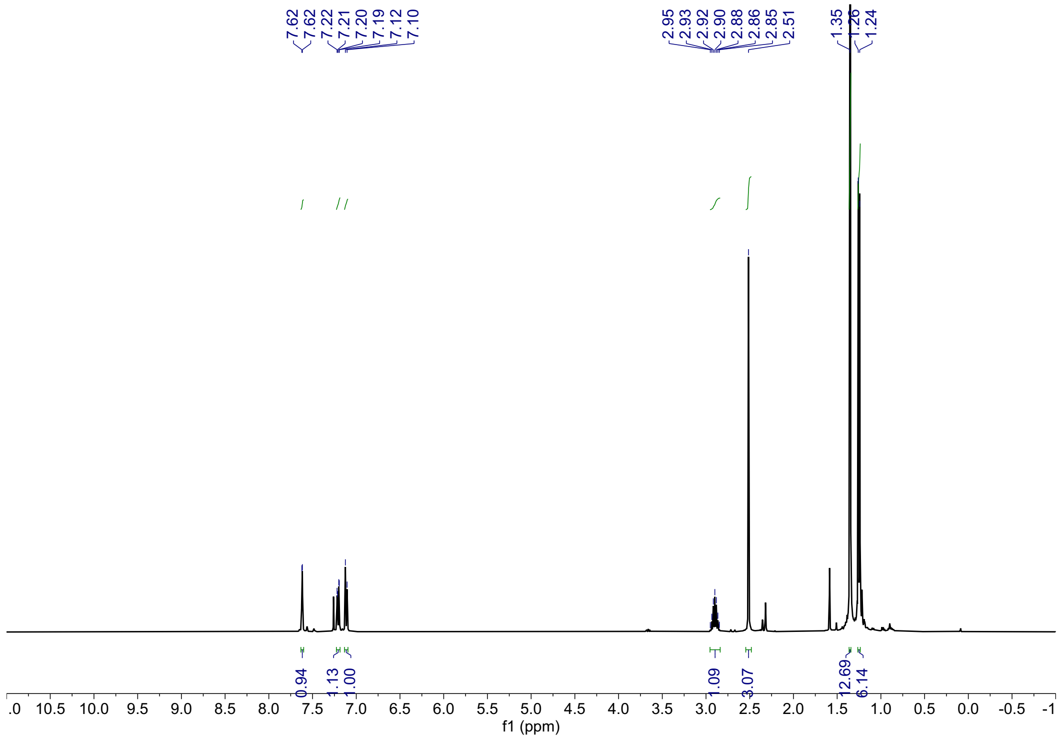

Supplementary Figure 43. ^{1}H NMR spectrum of **17** in $CDCl_3$.

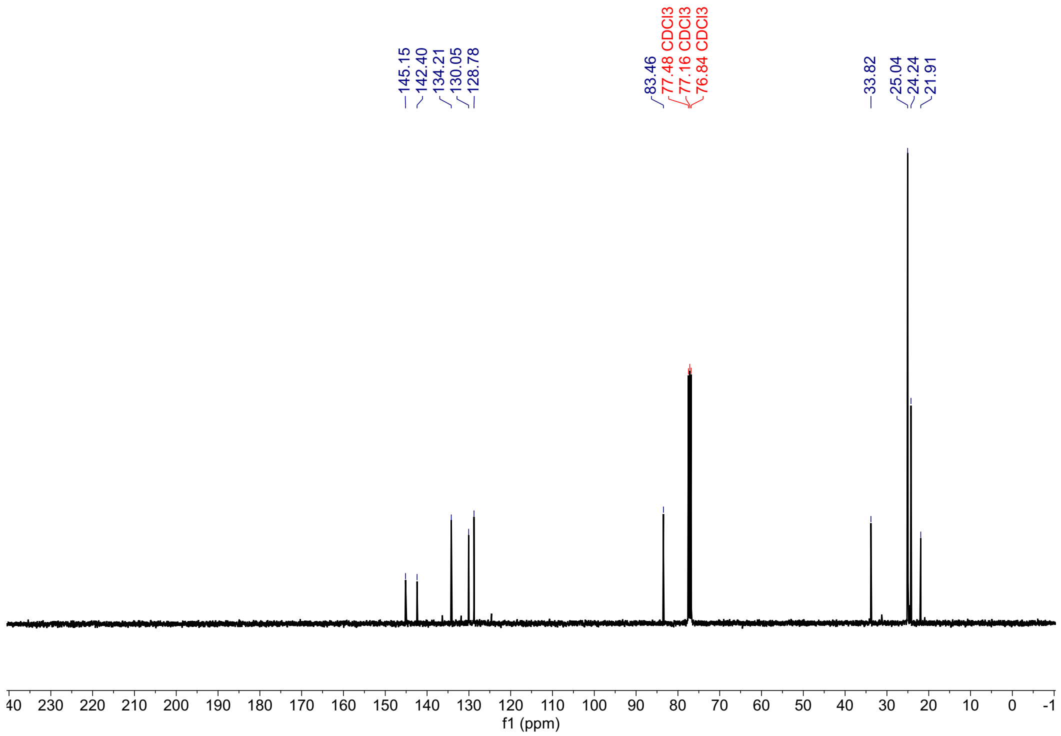

Supplementary Figure 44. ^{13}C NMR spectrum of **17** in $CDCl_3$.

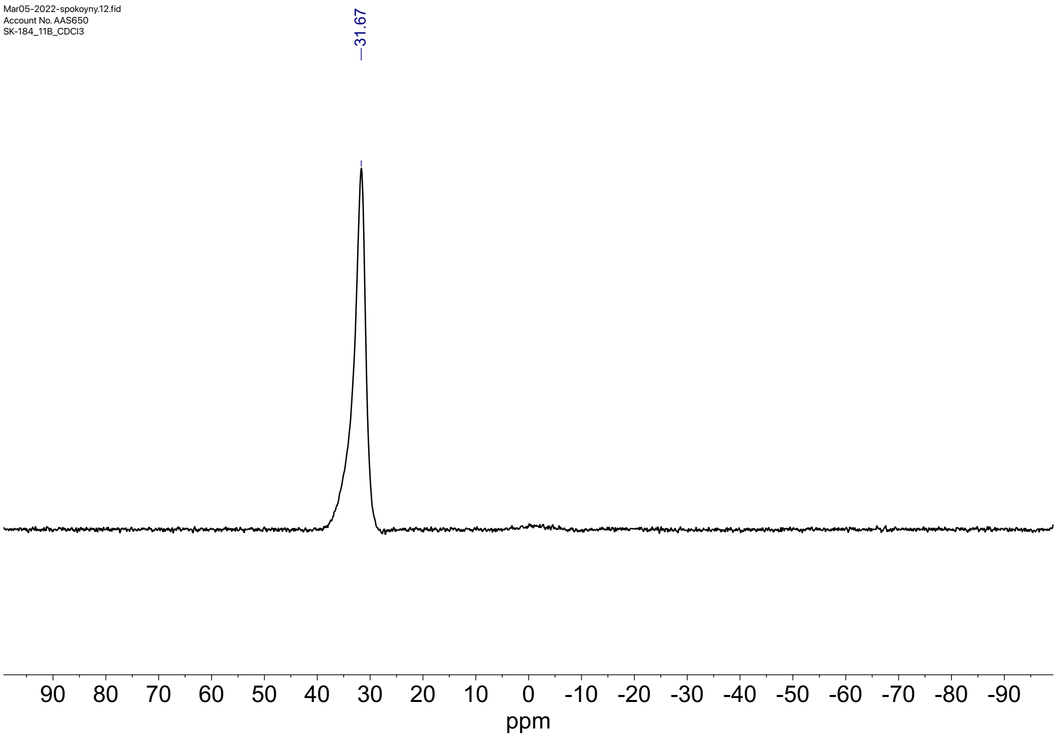

Supplementary Figure 45. ^{11}B NMR spectrum of **17** in $CDCl_3$.

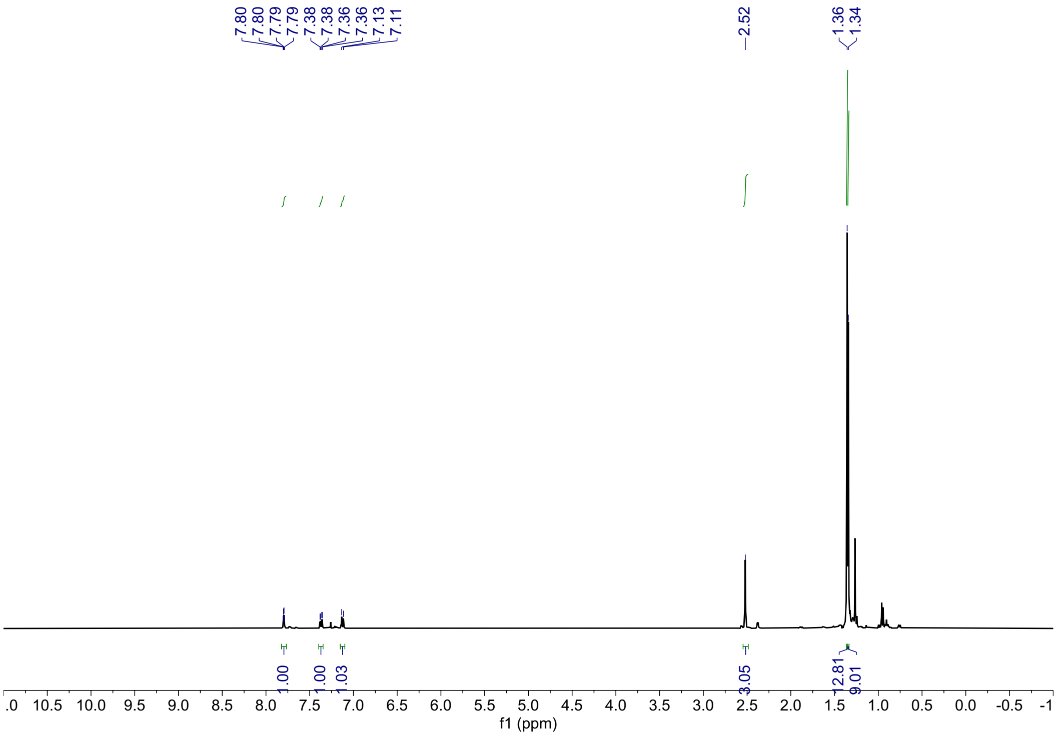

Supplementary Figure 46. ^{1}H NMR spectrum of **18** in $CDCl_3$.

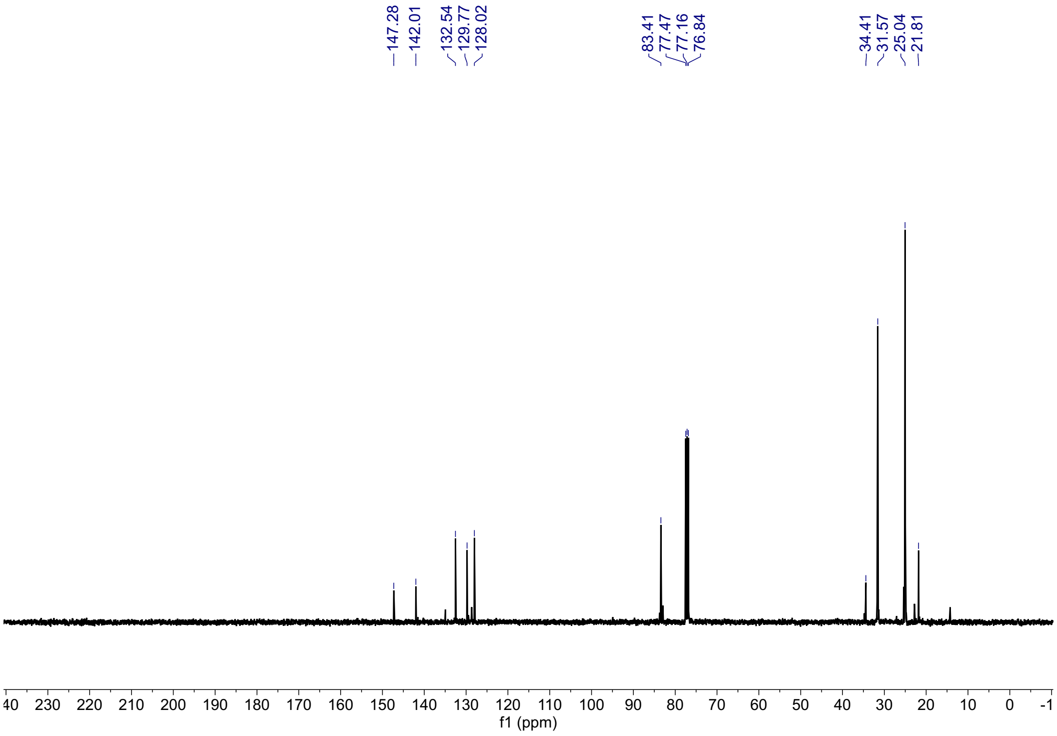

Supplementary Figure 47. ^{13}C NMR spectrum of **18** in $CDCl_3$.

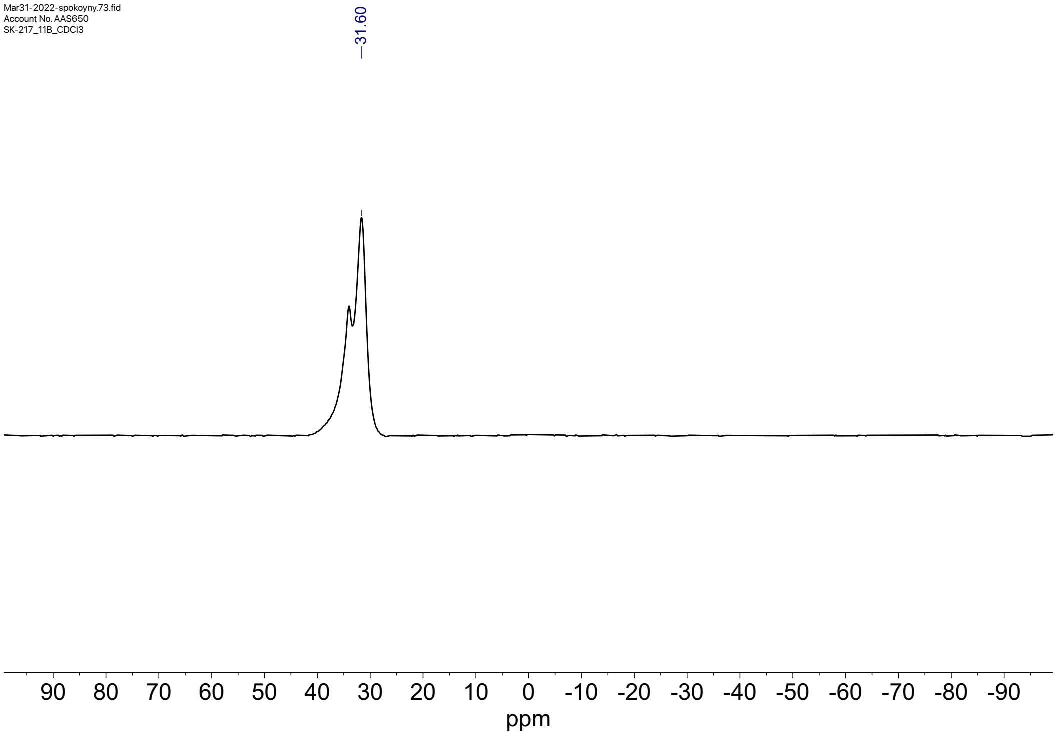

Supplementary Figure 48. ^{11}B NMR spectrum of **18** in $CDCl_3$.

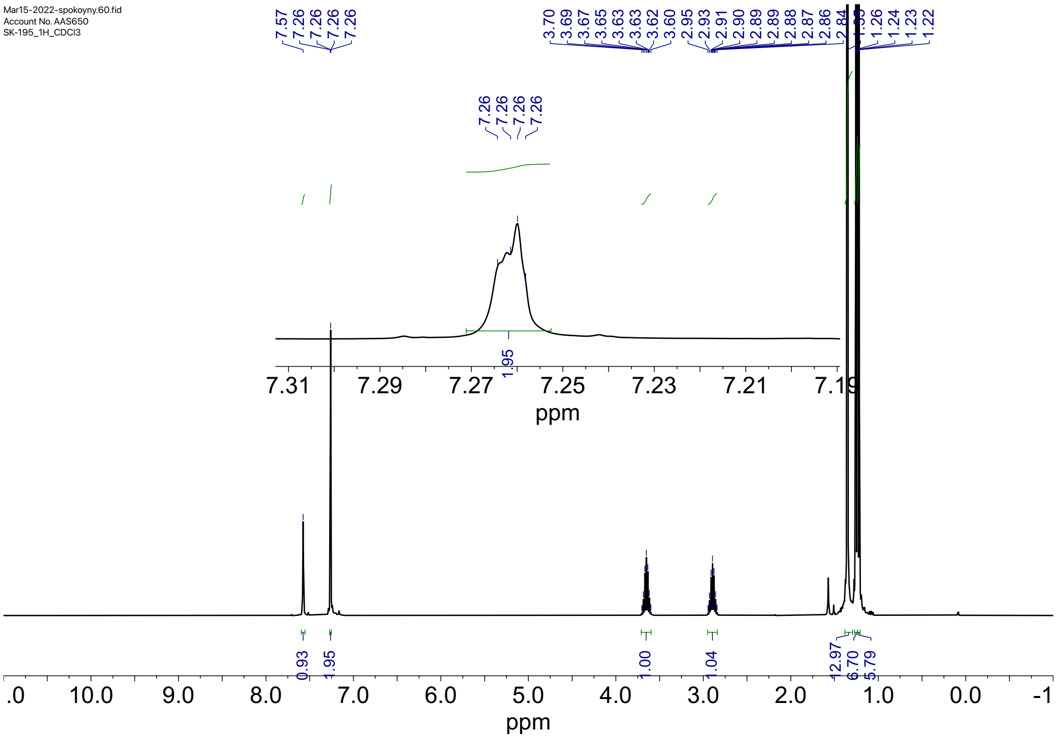

Supplementary Figure 49. ^{1}H NMR spectrum of **19** in $CDCl_3$.

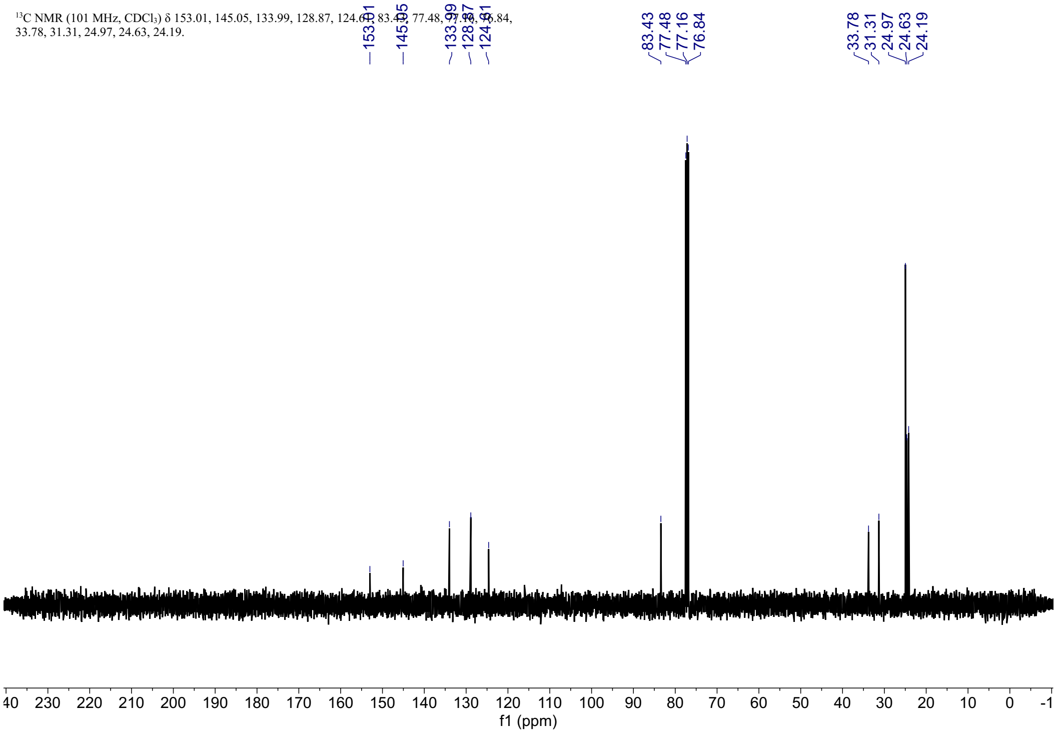

Supplementary Figure 50. ^{13}C NMR spectrum of **19** in $CDCl_3$.

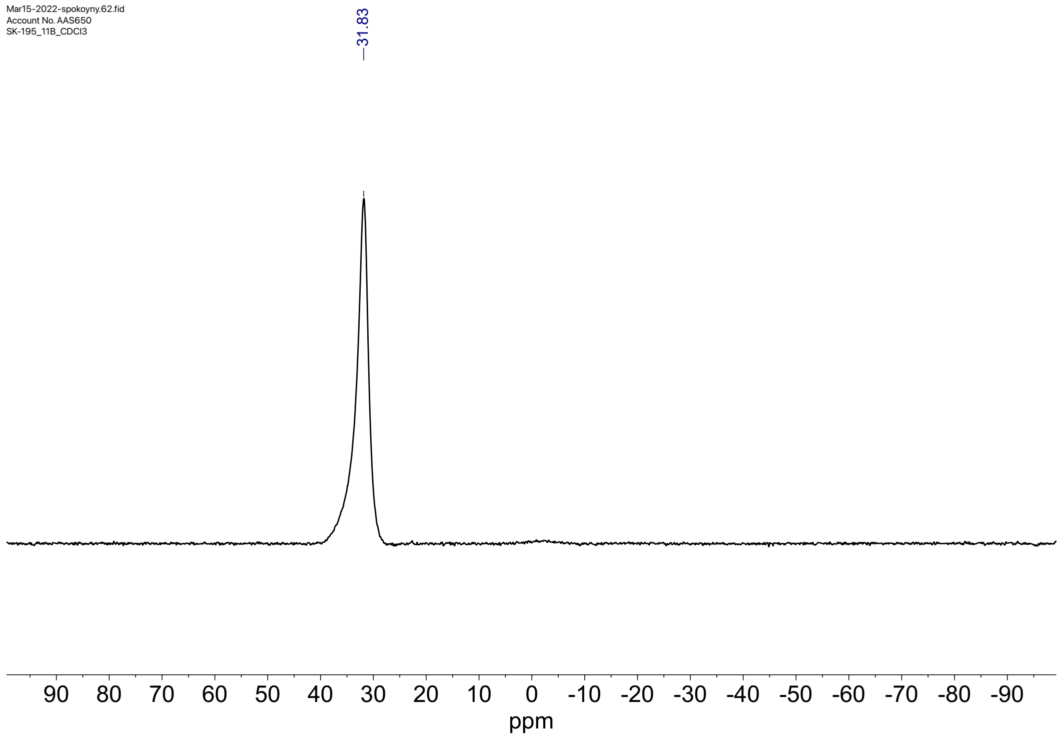

Supplementary Figure 51. ^{11}B NMR spectrum of **19** in $CDCl_3$.

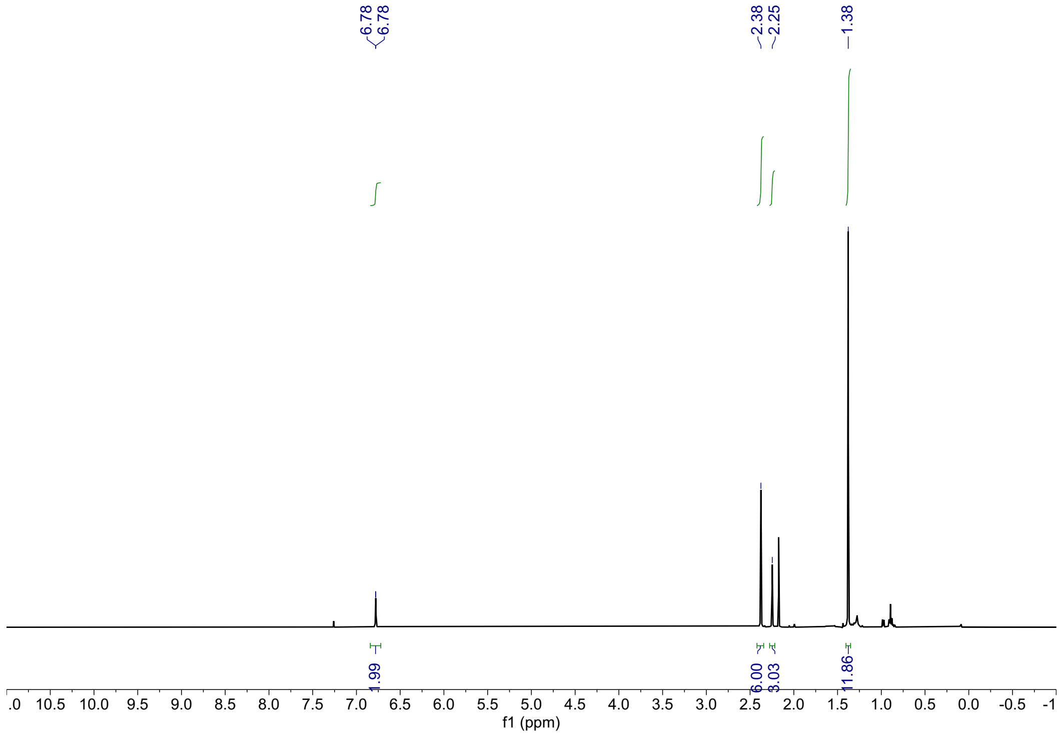

Supplementary Figure 52. ^{1}H NMR spectrum of **20** in $CDCl_3$.

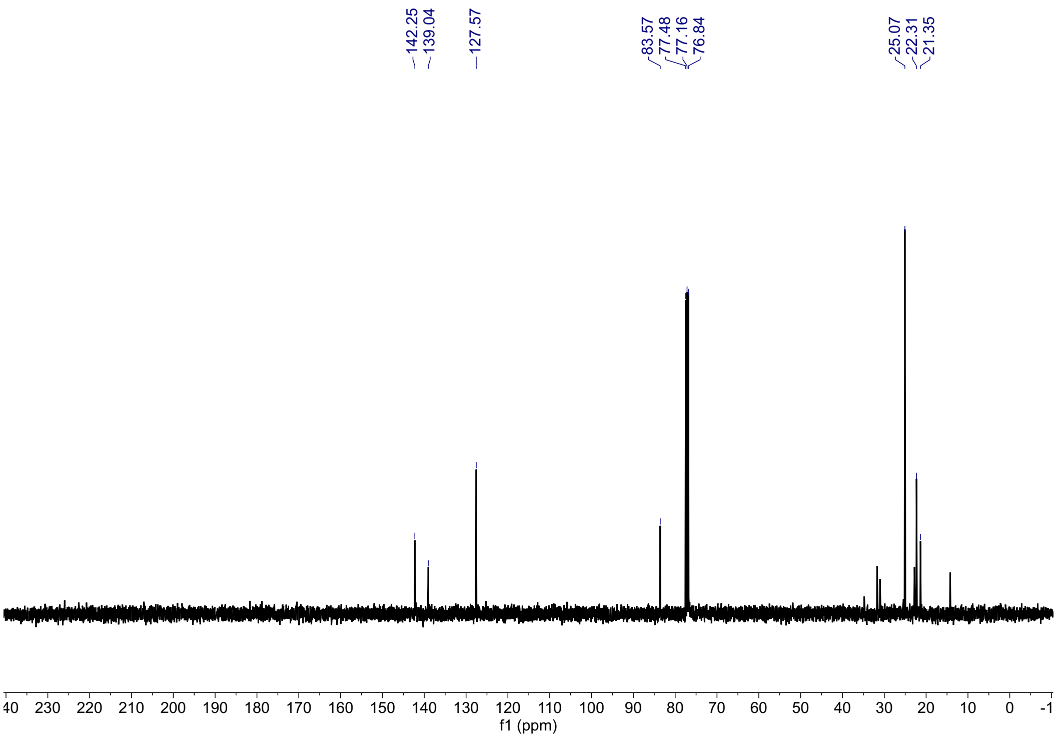

Supplementary Figure 53. ^{13}C NMR spectrum of **20** in $CDCl_3$.

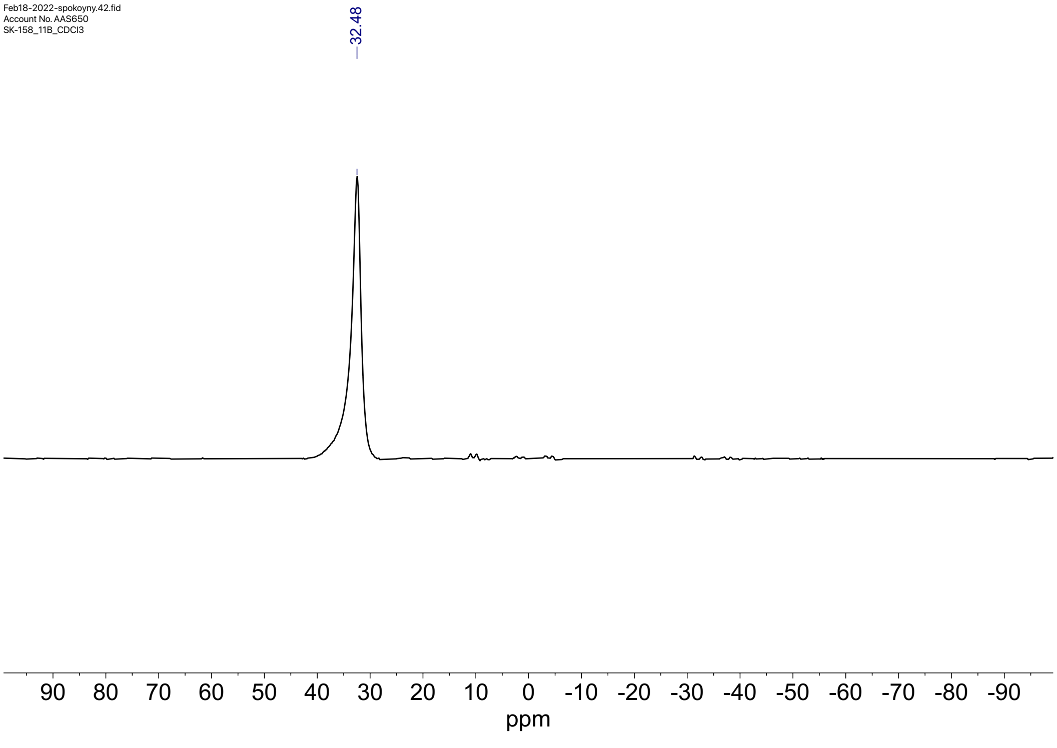

Supplementary Figure 54. ^{11}B NMR spectrum of **20** in $CDCl_3$.

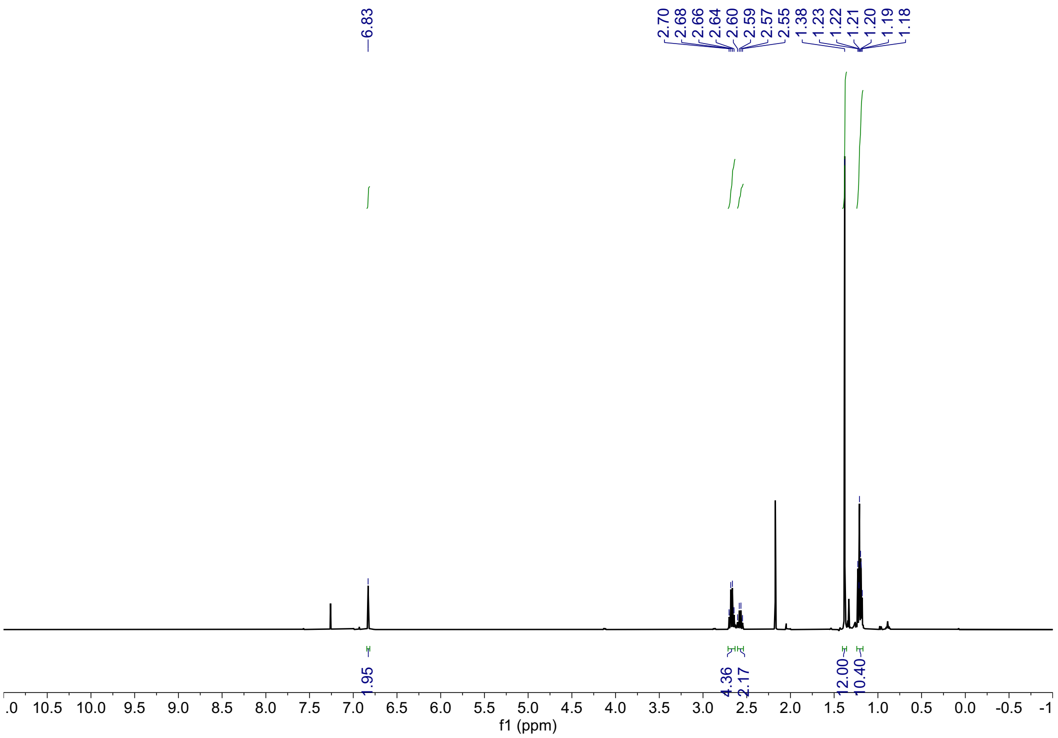

Supplementary Figure 55. ^{1}H NMR spectrum of **21** in $CDCl_3$.

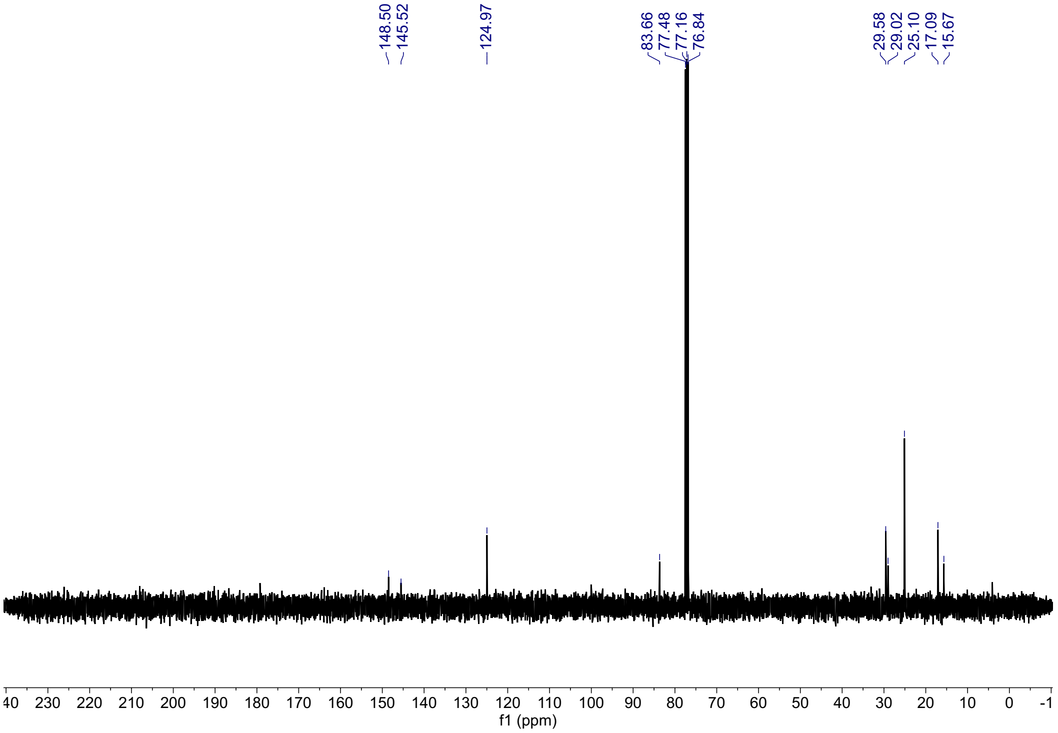

Supplementary Figure 56. ^{13}C NMR spectrum of **21** in $CDCl_3$.

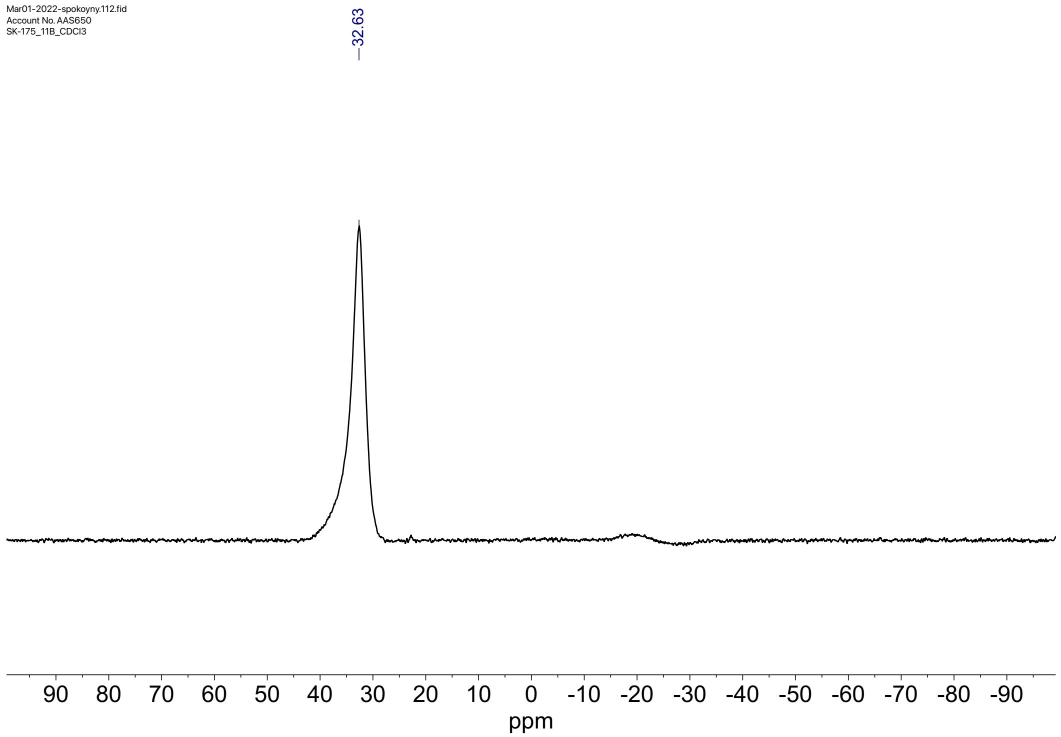

Supplementary Figure 57. ^{11}B NMR spectrum of **21** in $CDCl_3$.

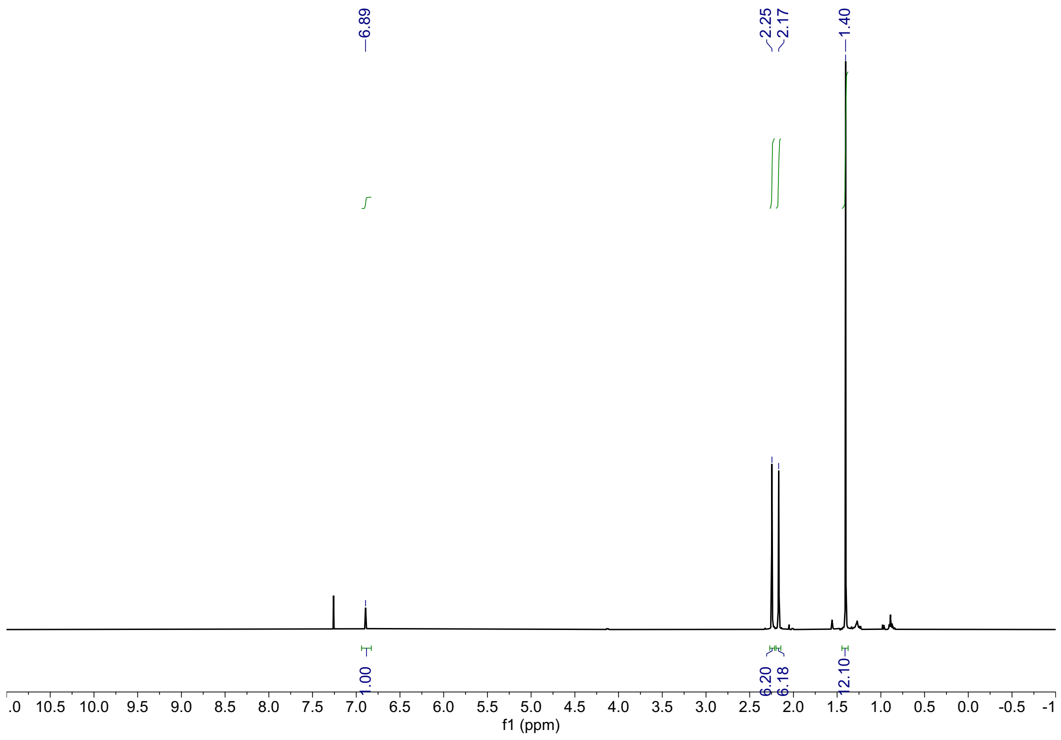

Supplementary Figure 58. ^{1}H NMR spectrum of **22** in $CDCl_3$.

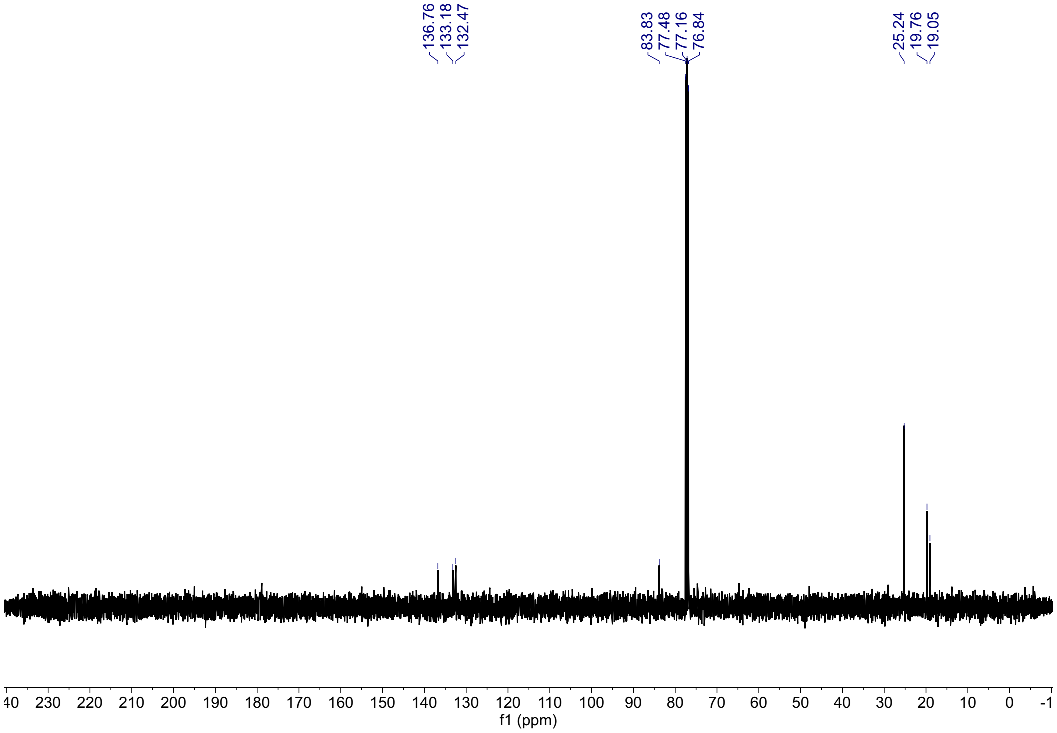

Supplementary Figure 59. ^{13}C NMR spectrum of **22** in $CDCl_3$.

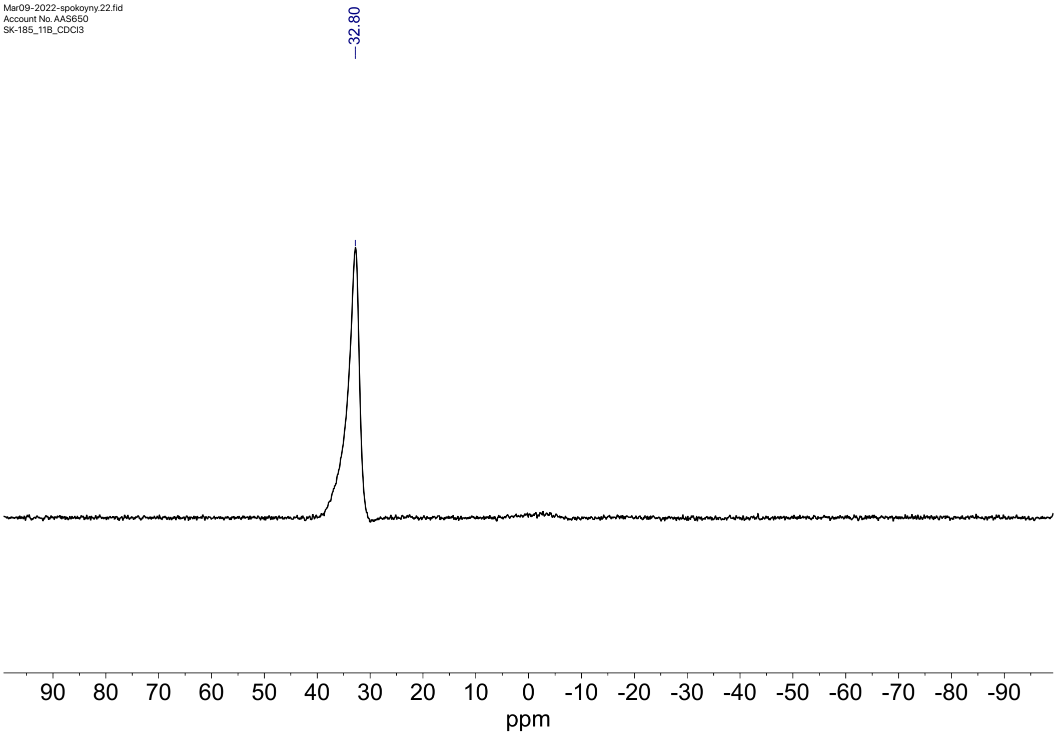

Supplementary Figure 60. ^{11}B NMR spectrum of **22** in $CDCl_3$.

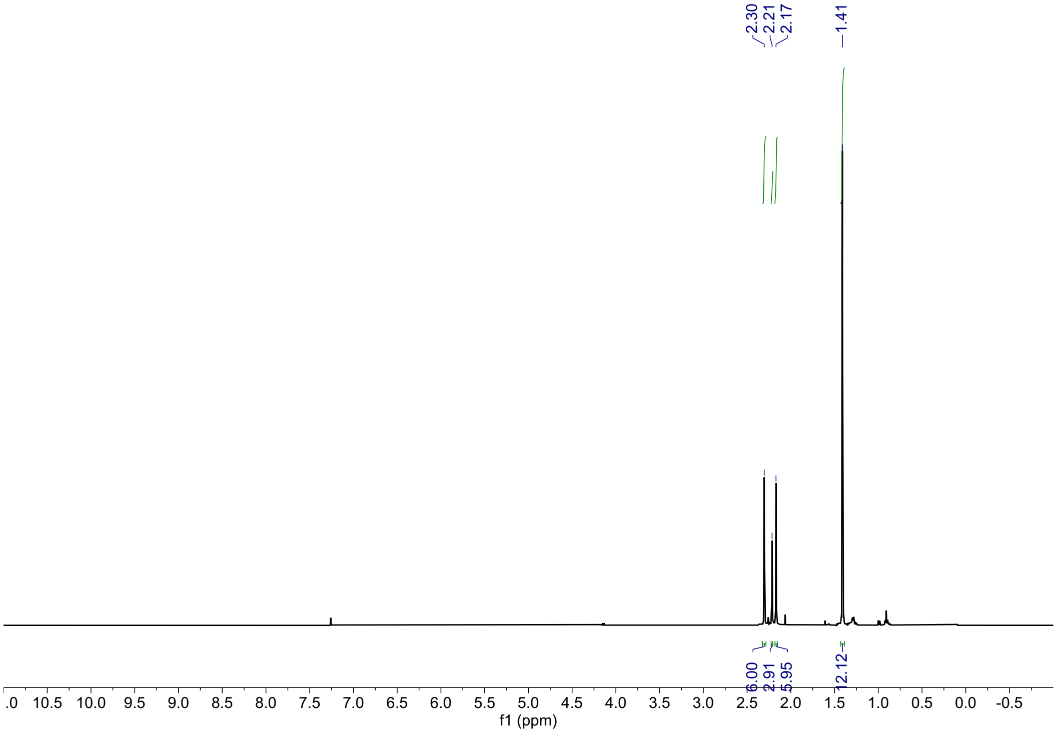

Supplementary Figure 61. ^{1}H NMR spectrum of **23** in $CDCl_3$.

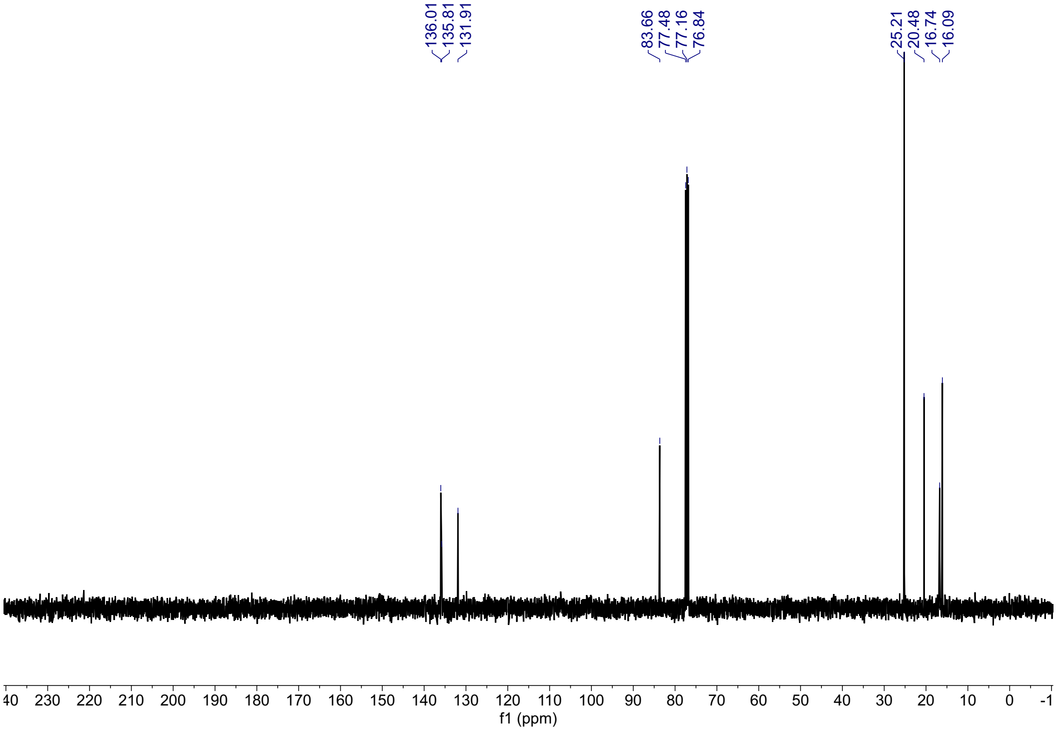

Supplementary Figure 62. ^{13}C NMR spectrum of **23** in $CDCl_3$.

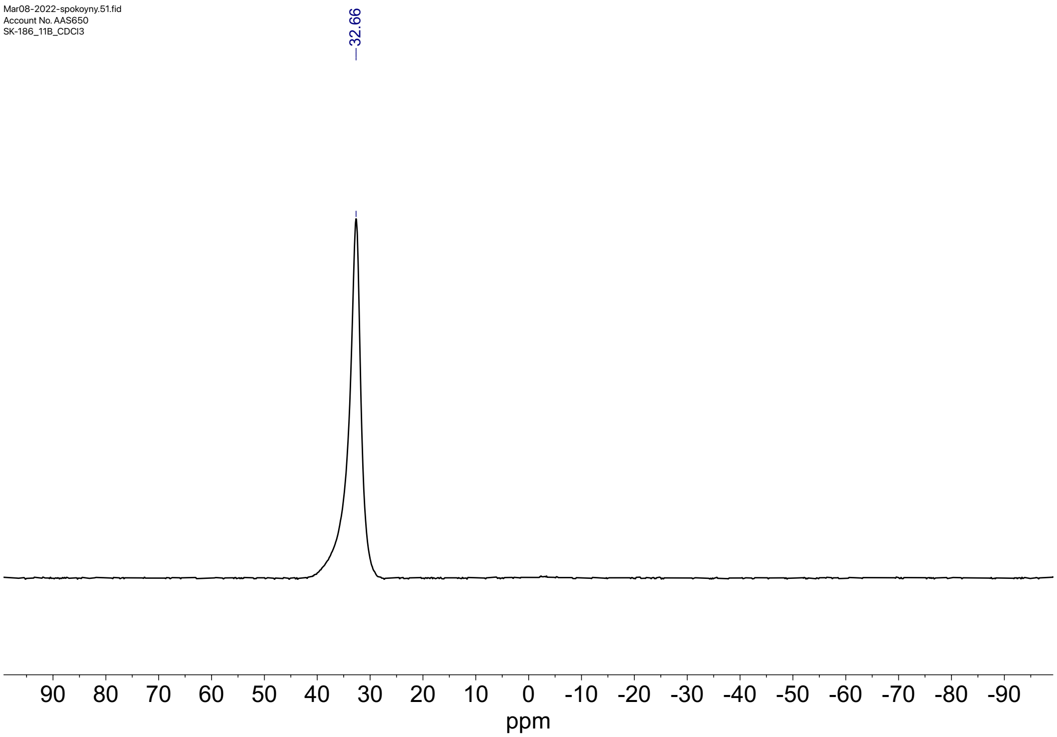

Supplementary Figure 63. ^{11}B NMR spectrum of **23** in $CDCl_3$.

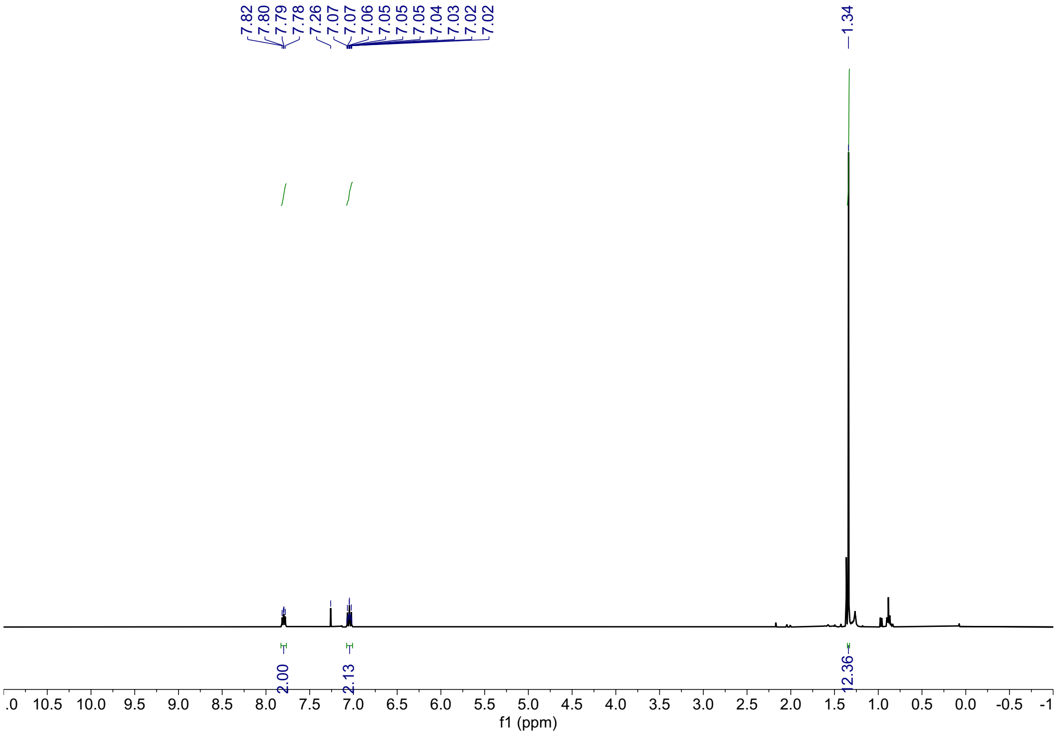

Supplementary Figure 64. ^{1}H NMR spectrum of **24** in $CDCl_3$.

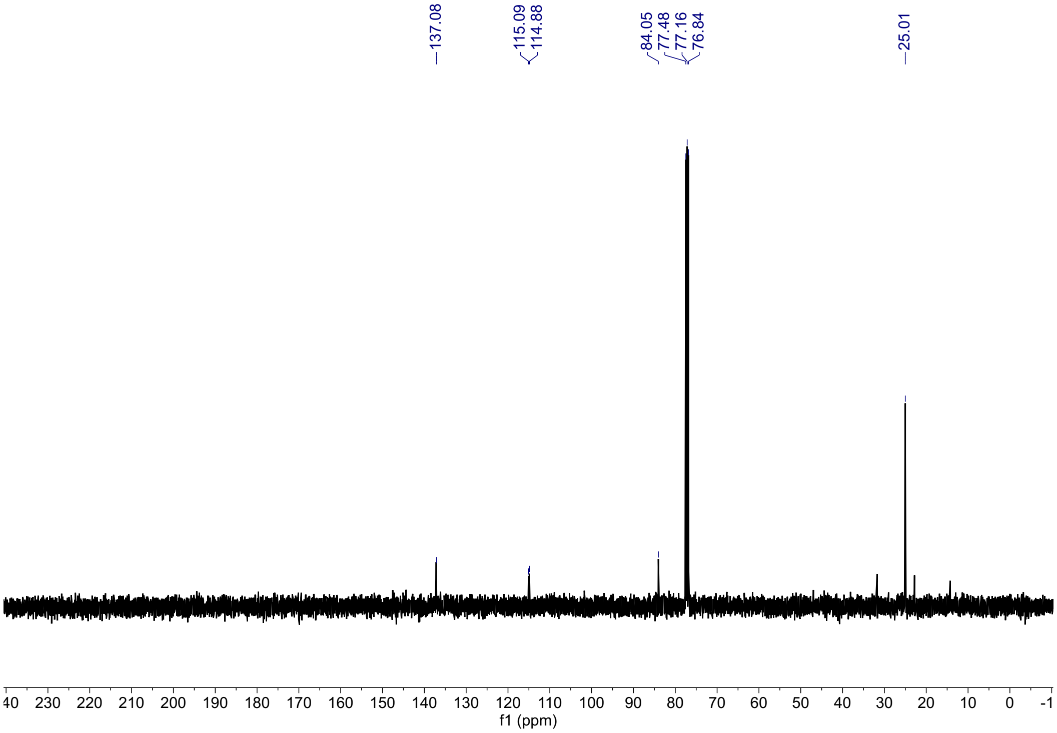

Supplementary Figure 65. ^{13}C NMR spectrum of **24** in $CDCl_3$.

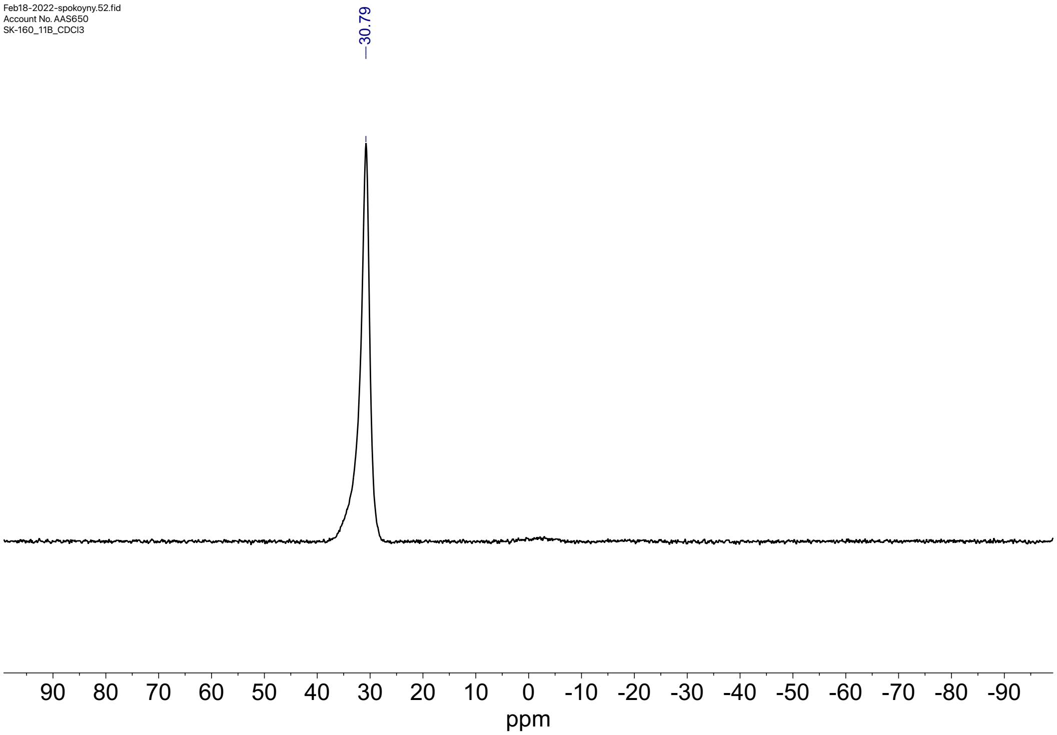

Supplementary Figure 66. ^{11}B NMR spectrum of **24** in $CDCl_3$.

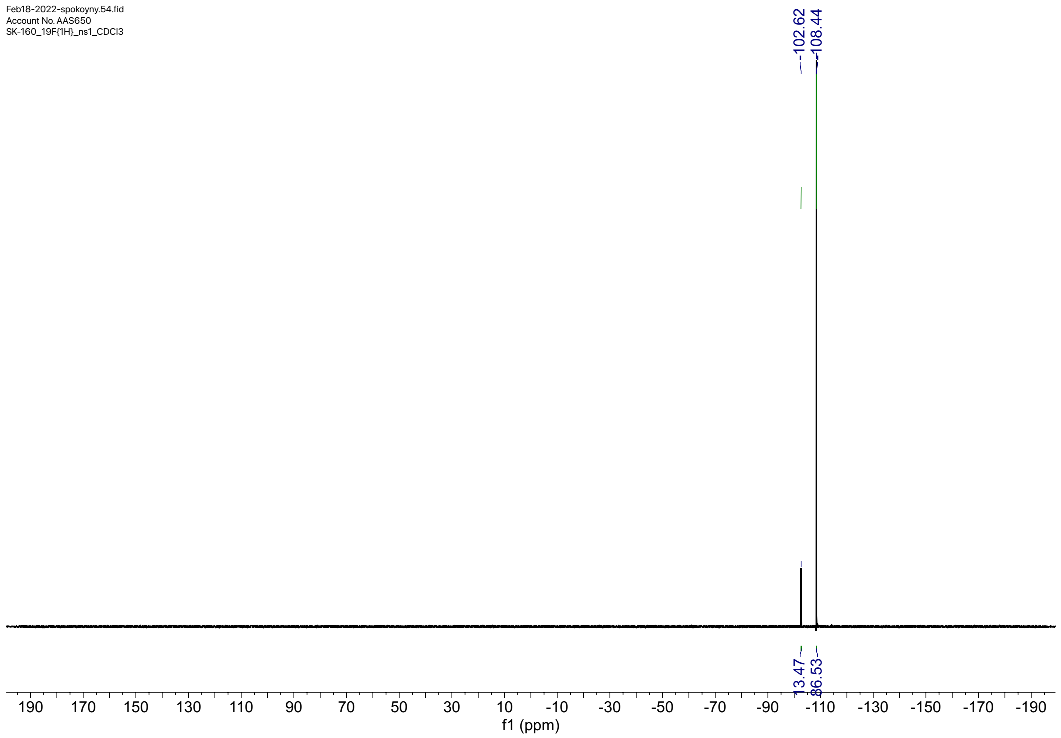

Supplementary Figure 67. ^{19}F NMR spectrum of **24** in $CDCl_3$. (ns = 1)

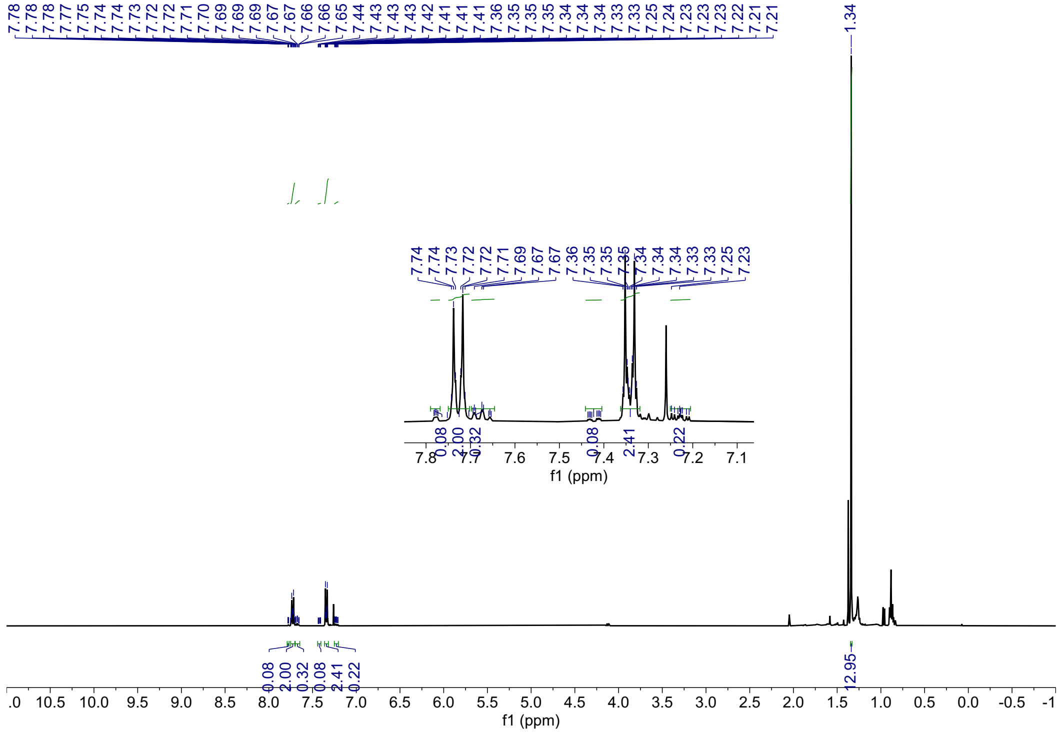

Supplementary Figure 68. ^{1}H NMR spectrum of **25** in $CDCl_3$.

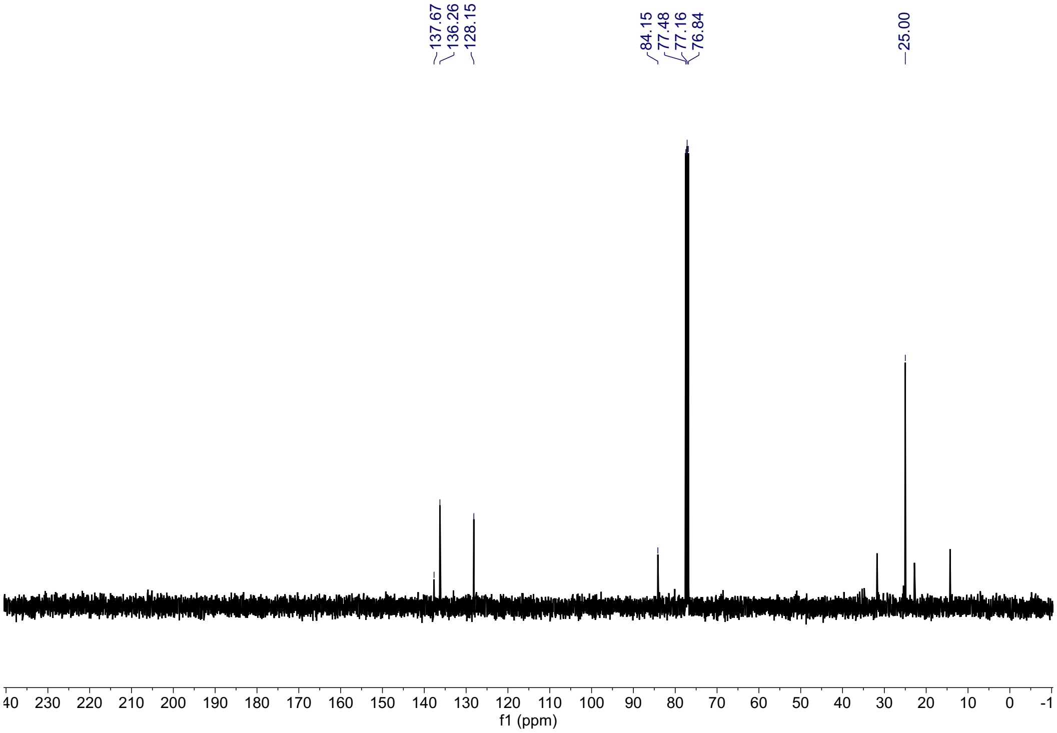

Supplementary Figure 69. ^{13}C NMR spectrum of **25** in $CDCl_3$.

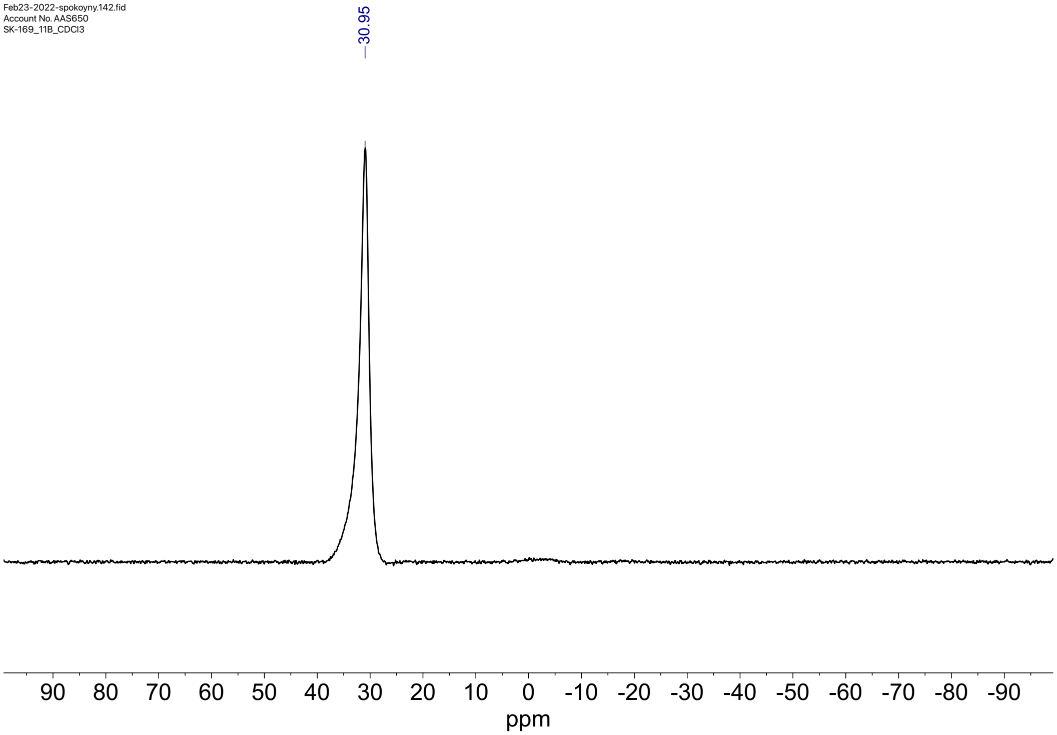

Supplementary Figure 70. ^{11}B NMR spectrum of **25** in $CDCl_3$.

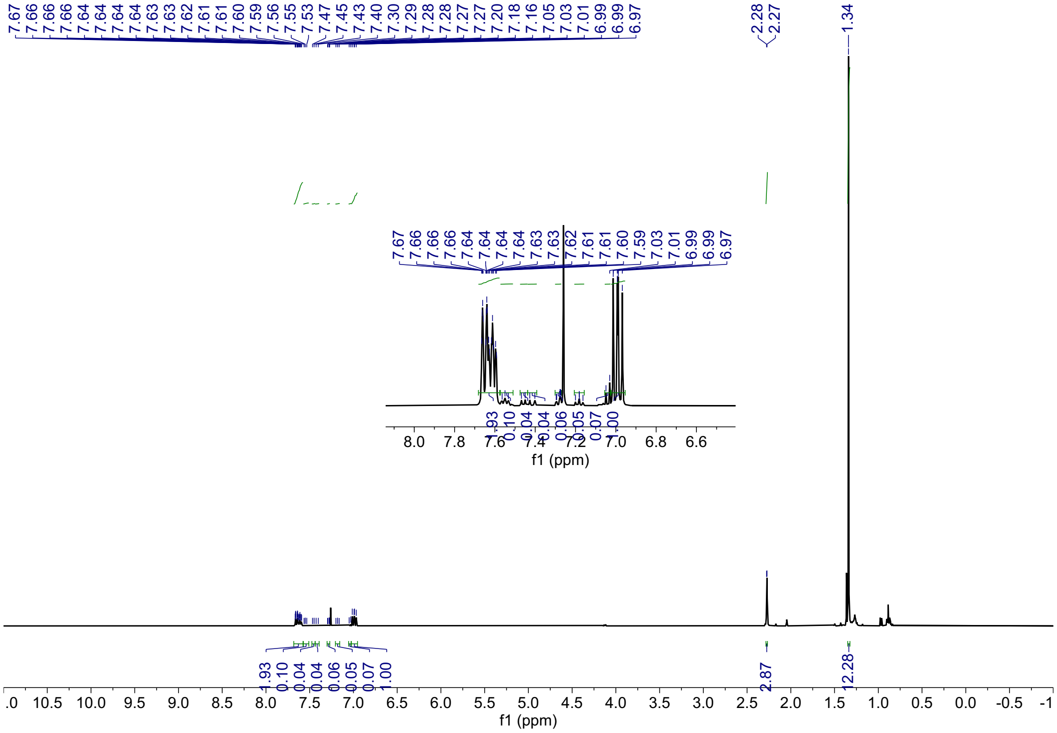

Supplementary Figure 71. ^{1}H NMR spectrum of **26** in $CDCl_3$.

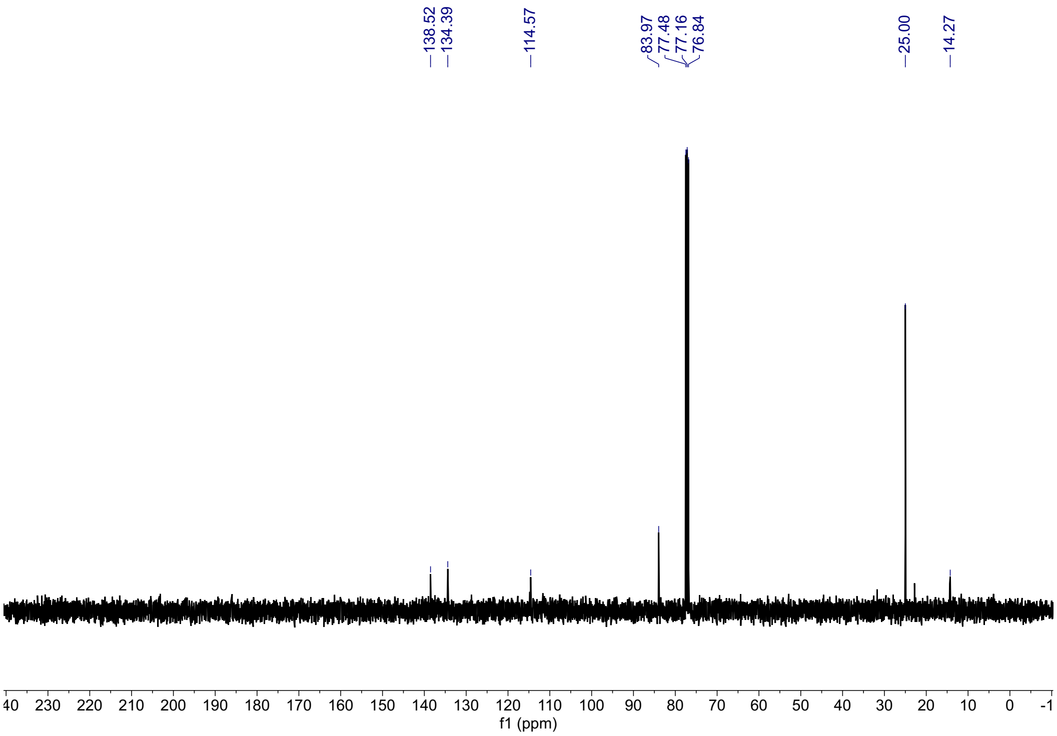

Supplementary Figure 72. ^{13}C NMR spectrum of **26** in $CDCl_3$.

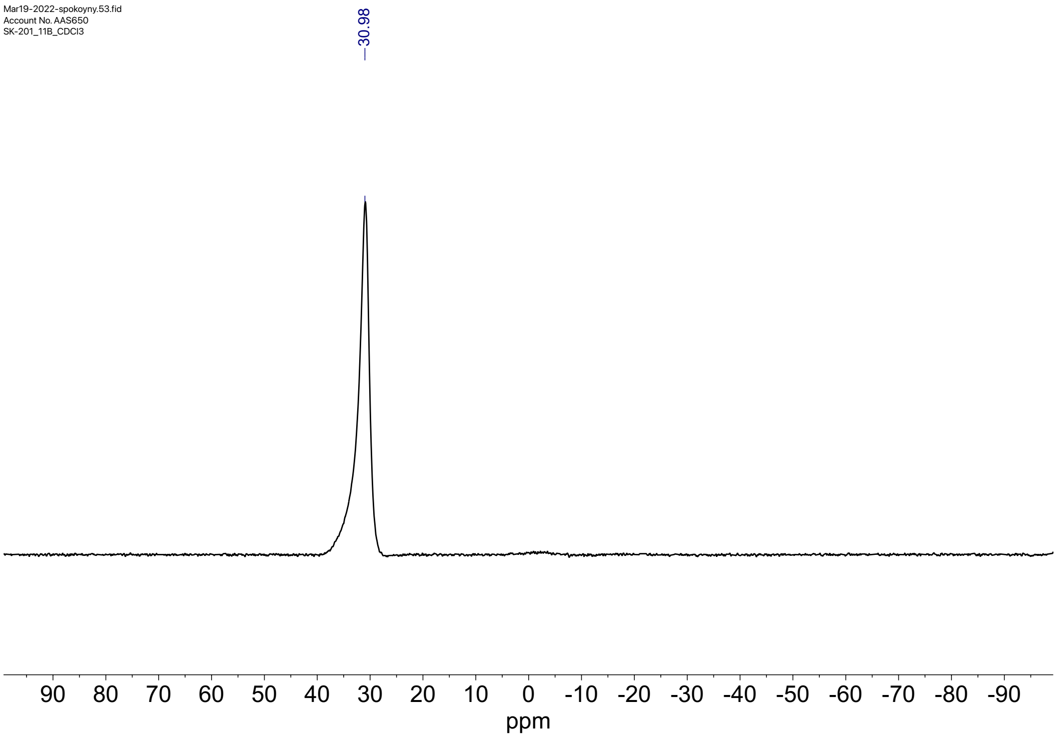

Supplementary Figure 73. ^{11}B NMR spectrum of **26** in $CDCl_3$.

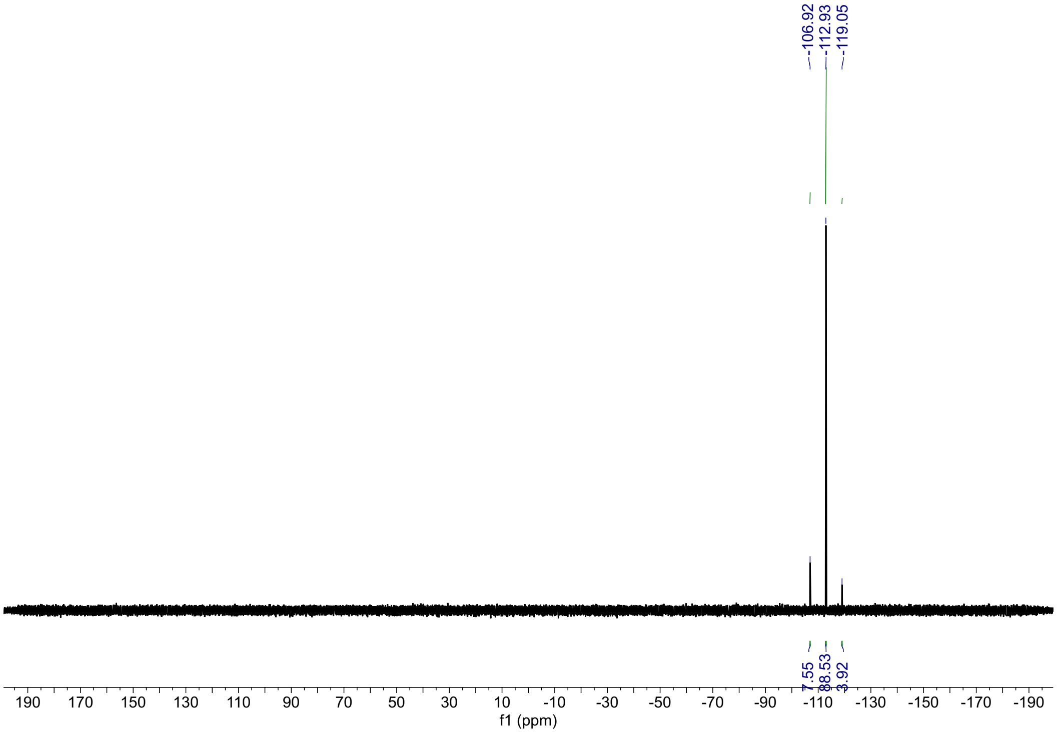

Supplementary Figure 74. ^{19}F NMR spectrum of **26** in $CDCl_3$. (ns = 1)

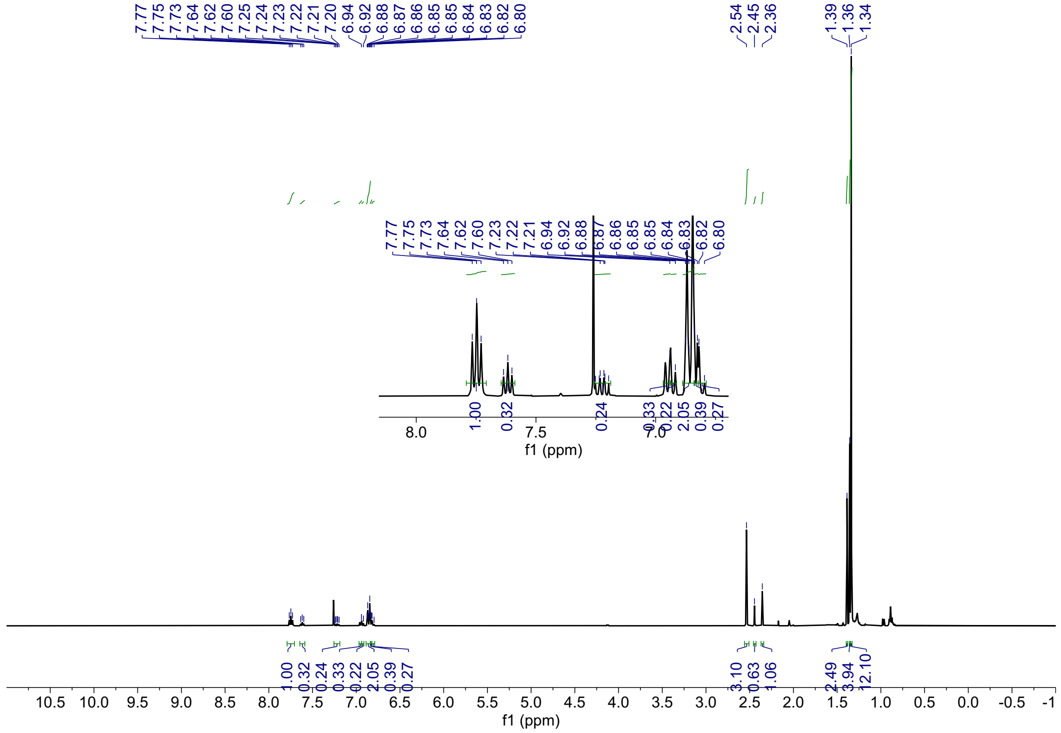

Supplementary Figure 75. ^{1}H NMR spectrum of **27** in $CDCl_3$.

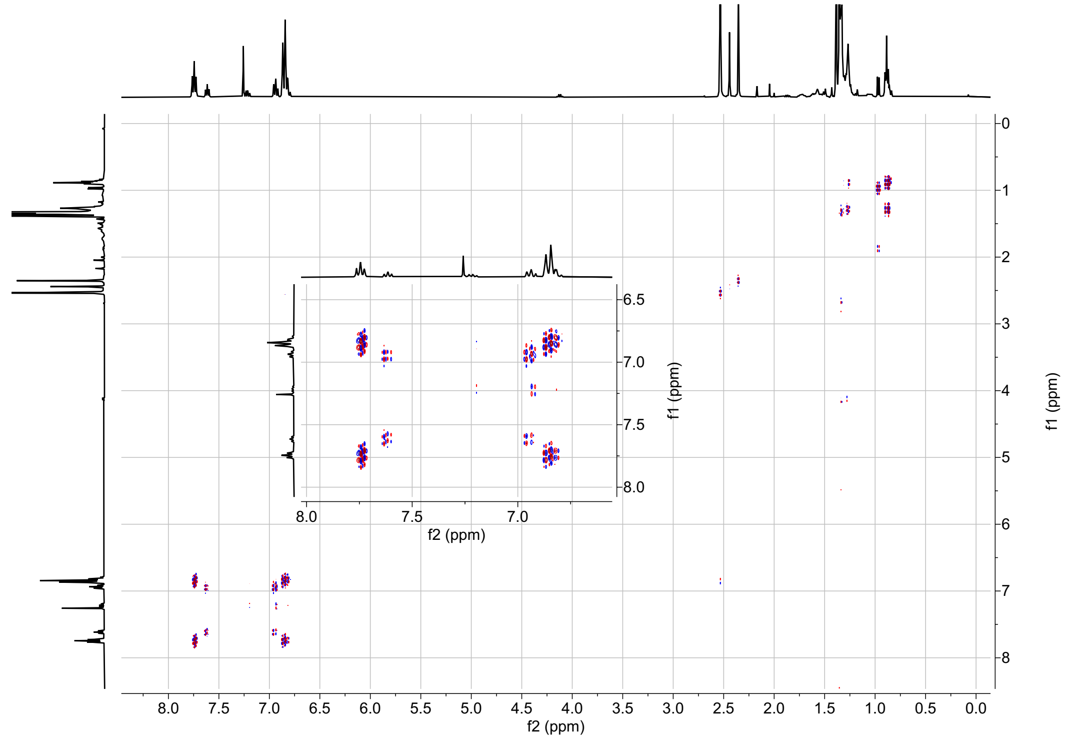

Supplementary Figure 76. ^{1}H-^{1}H COSY NMR spectrum of **27** in $CDCl_3$.

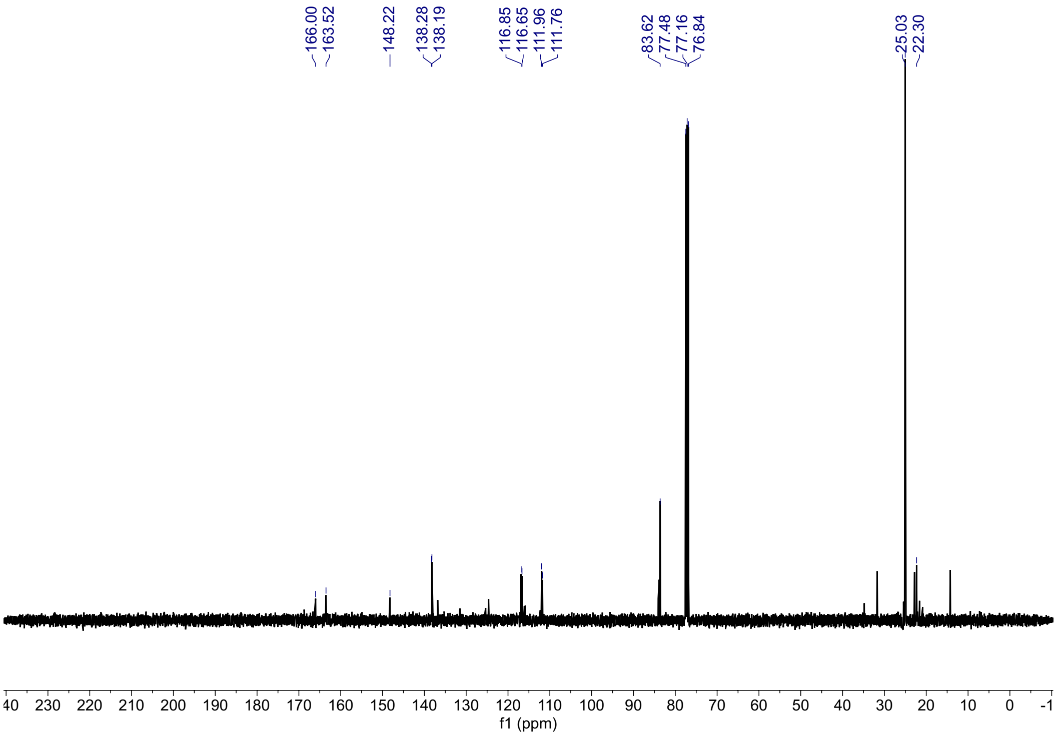

Supplementary Figure 77. ^{13}C NMR spectrum of **27** in $CDCl_3$.

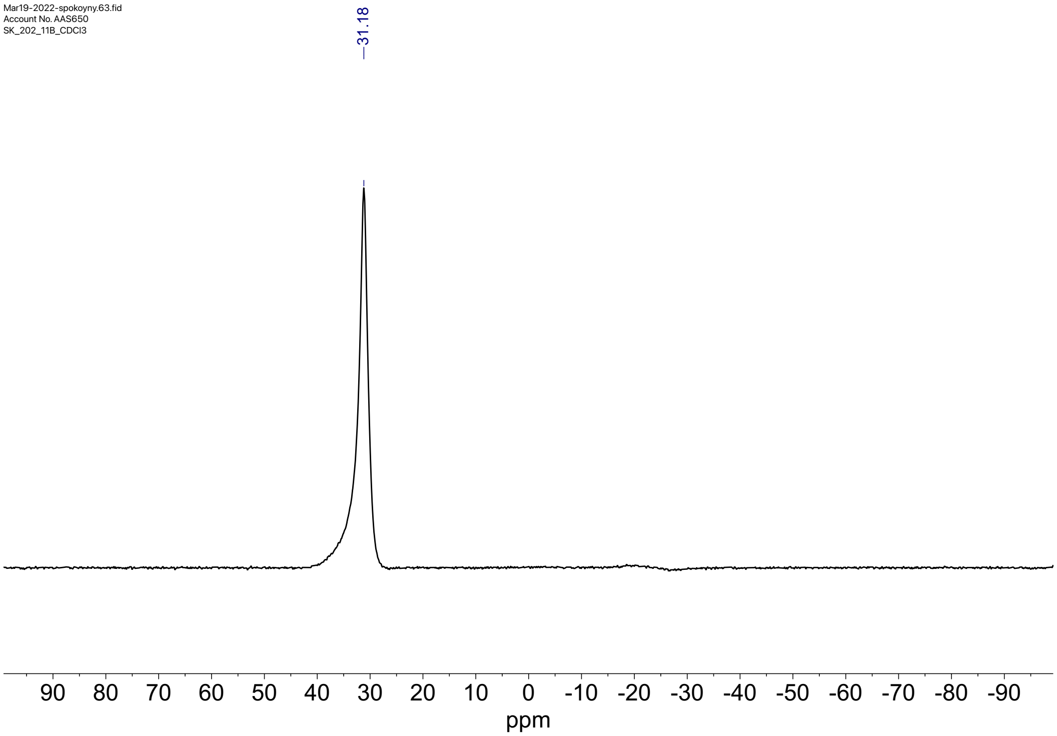

Supplementary Figure 78. ^{11}B NMR spectrum of **27** in $CDCl_3$.

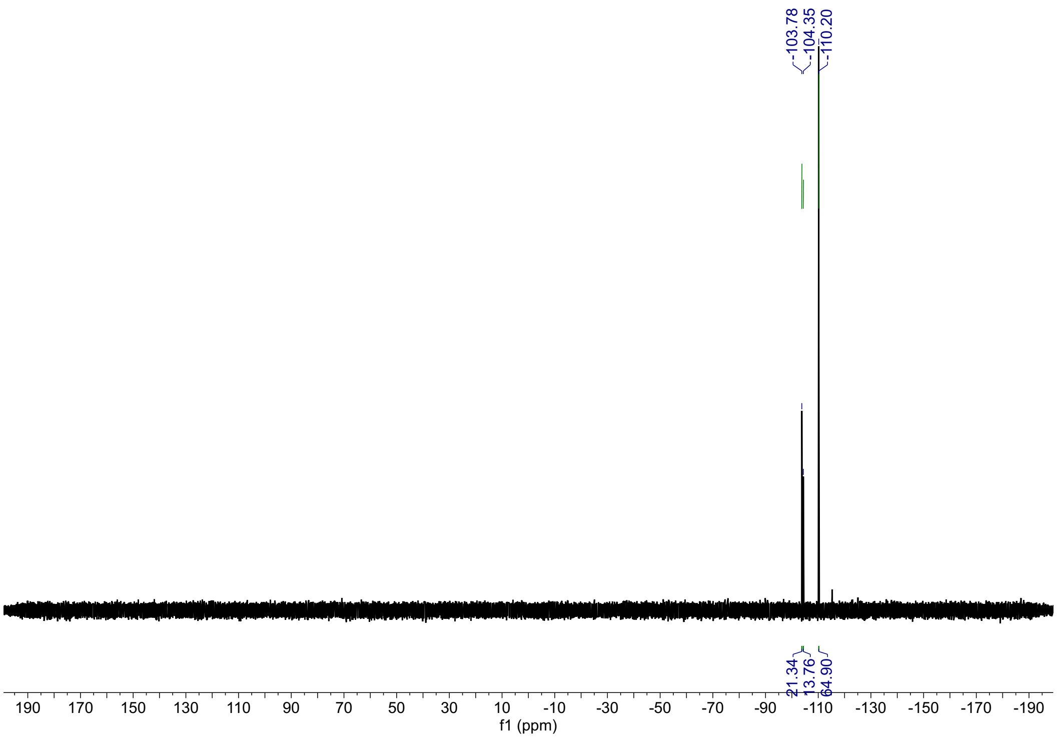

Supplementary Figure 79. ^{19}F NMR spectrum of **27** in $CDCl_3$. (ns = 1)

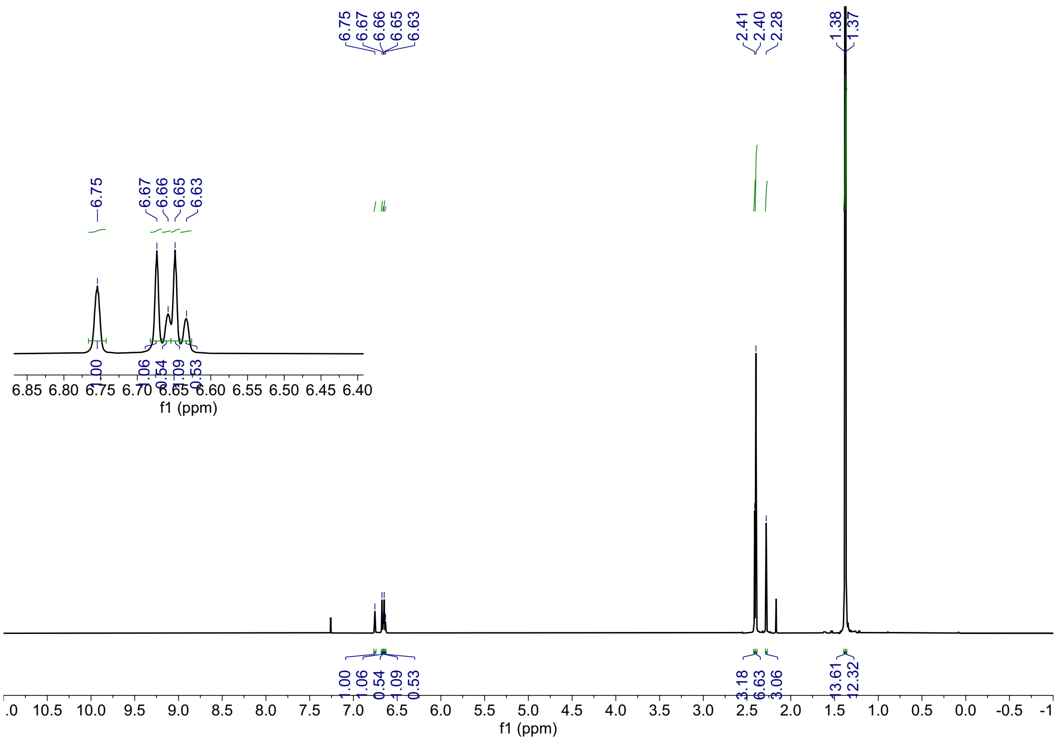

Supplementary Figure 80. ^{1}H NMR spectrum of **28** in $CDCl_3$.

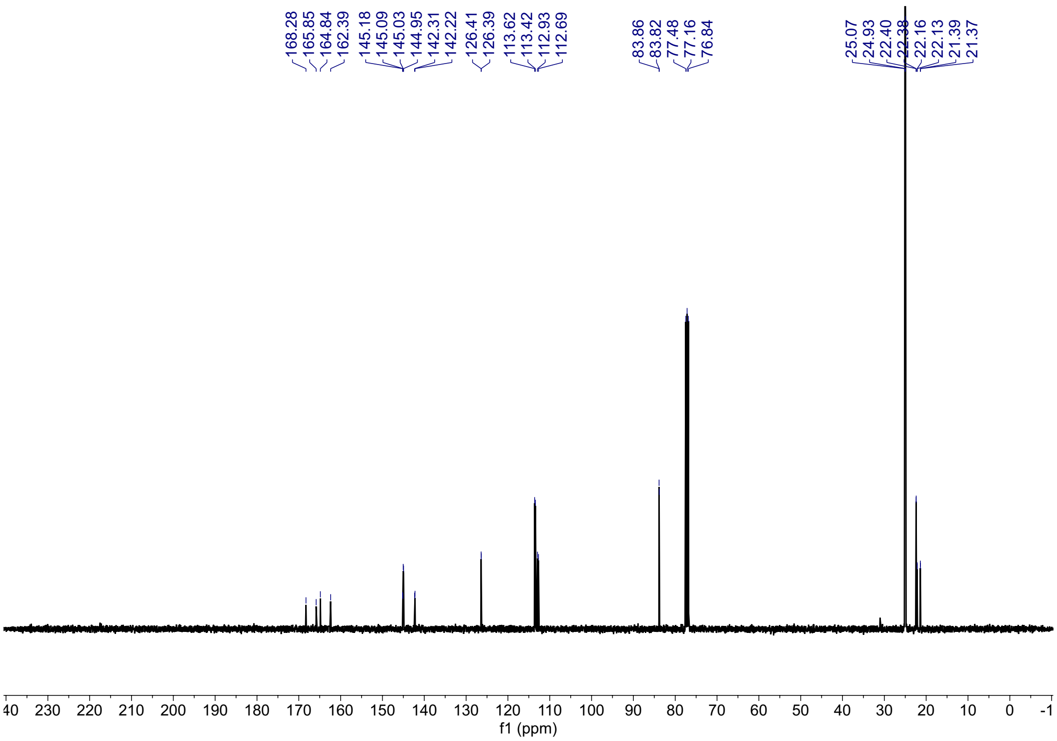

Supplementary Figure 81. ^{13}C NMR spectrum of **28** in $CDCl_3$.

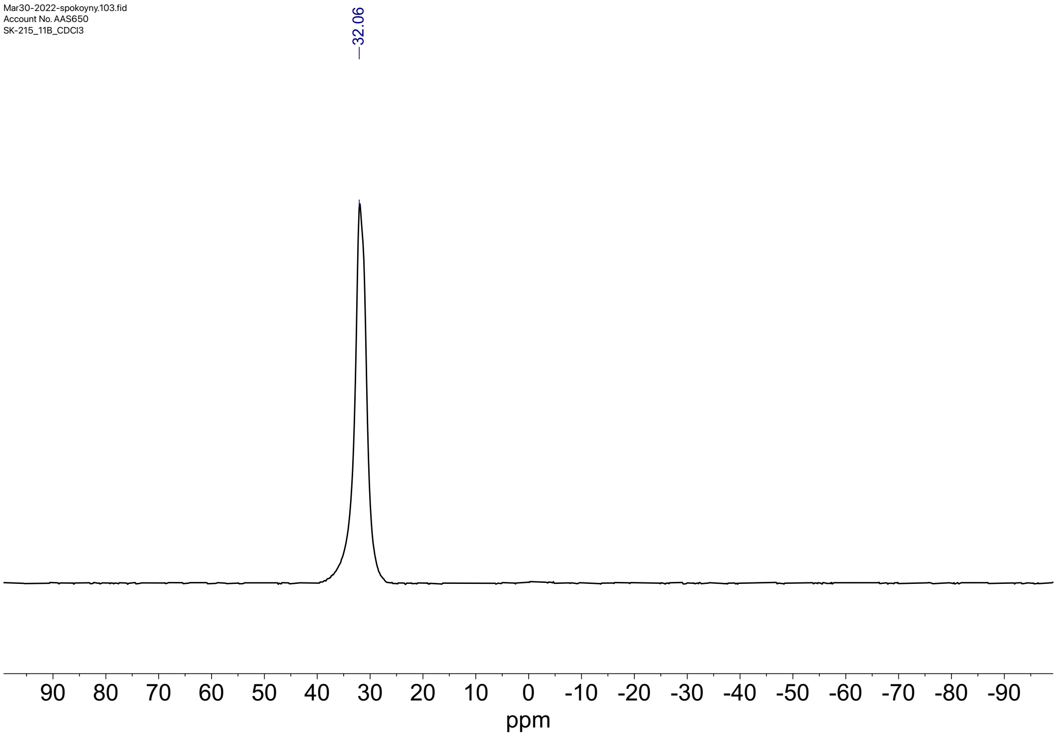

Supplementary Figure 82. ^{11}B NMR spectrum of **28** in $CDCl_3$.

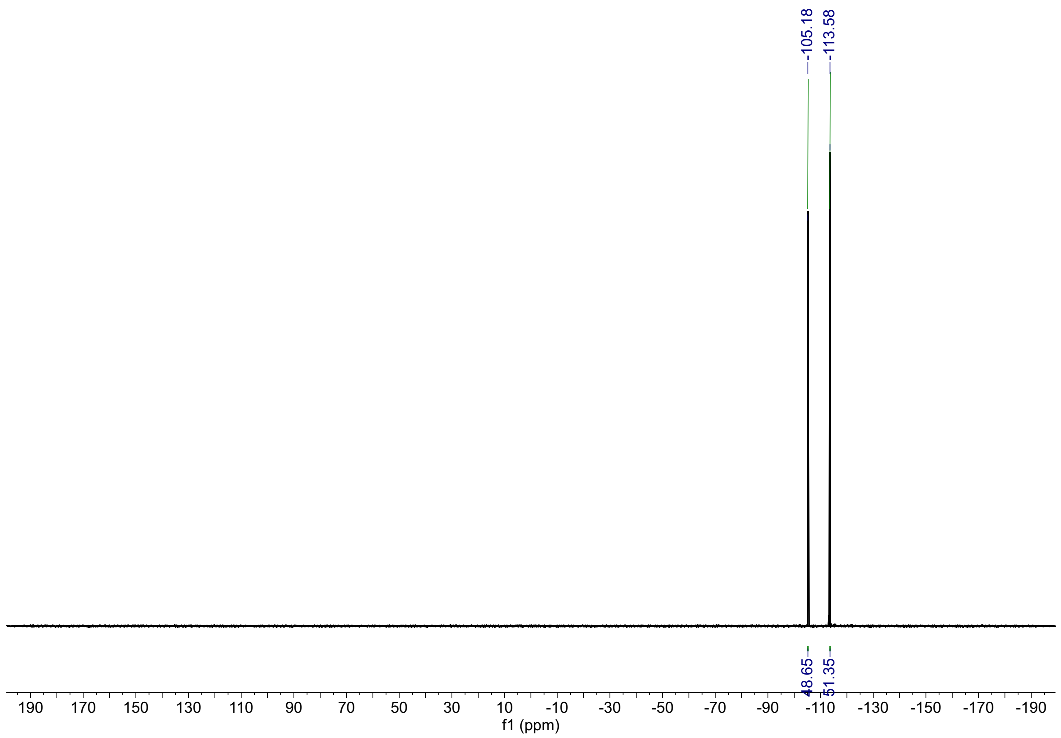

Supplementary Figure 83. ^{19}F NMR spectrum of **28** in $CDCl_3$. (ns = 1)

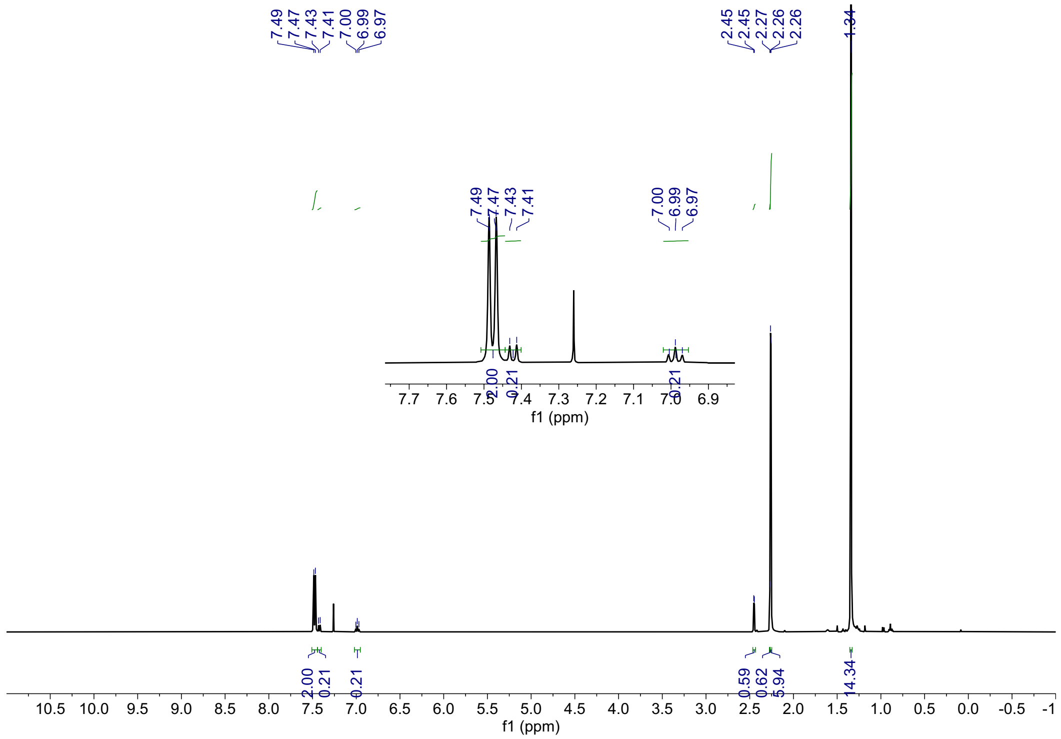

Supplementary Figure 84. ^{1}H NMR spectrum of **29** in $CDCl_3$.

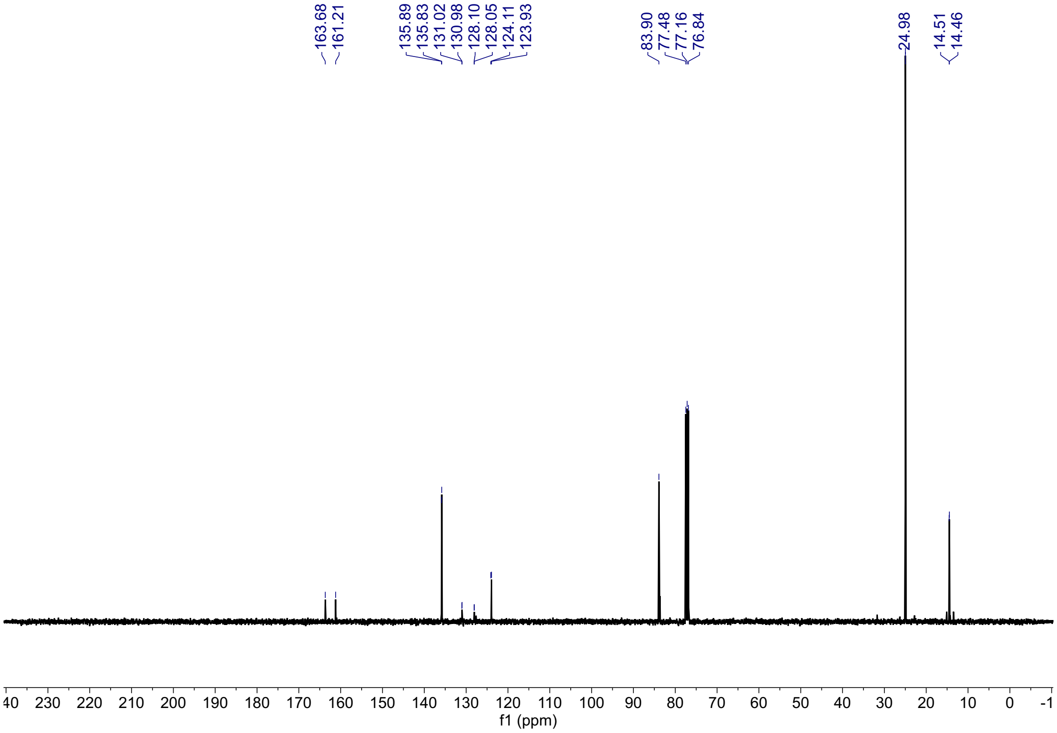

Supplementary Figure 85. ^{13}C NMR spectrum of **29** in $CDCl_3$.

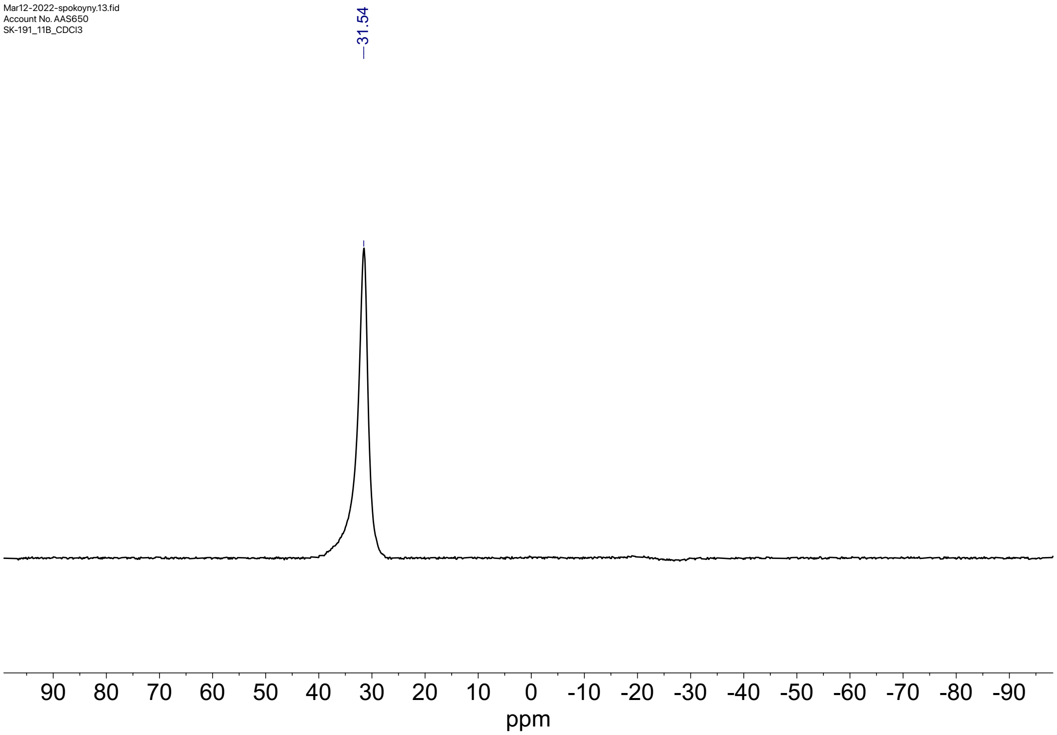

Supplementary Figure 86. ^{11}B NMR spectrum of **29** in $CDCl_3$.

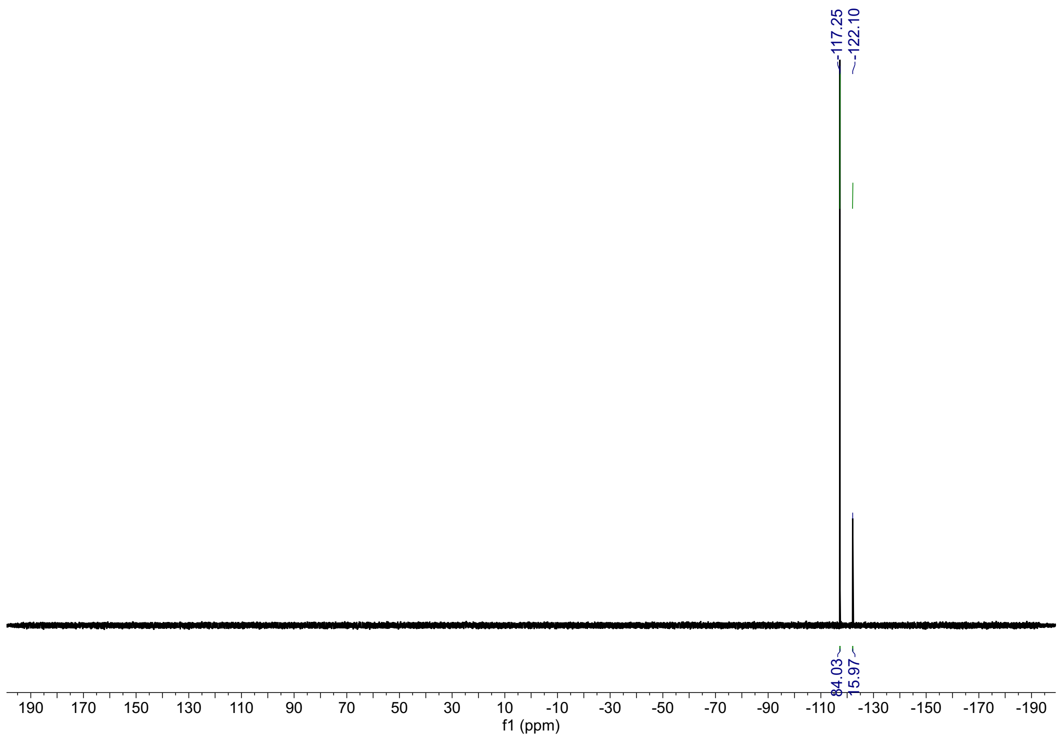

Supplementary Figure 87. ^{19}F NMR spectrum of **29** in $CDCl_3$. (ns = 1)

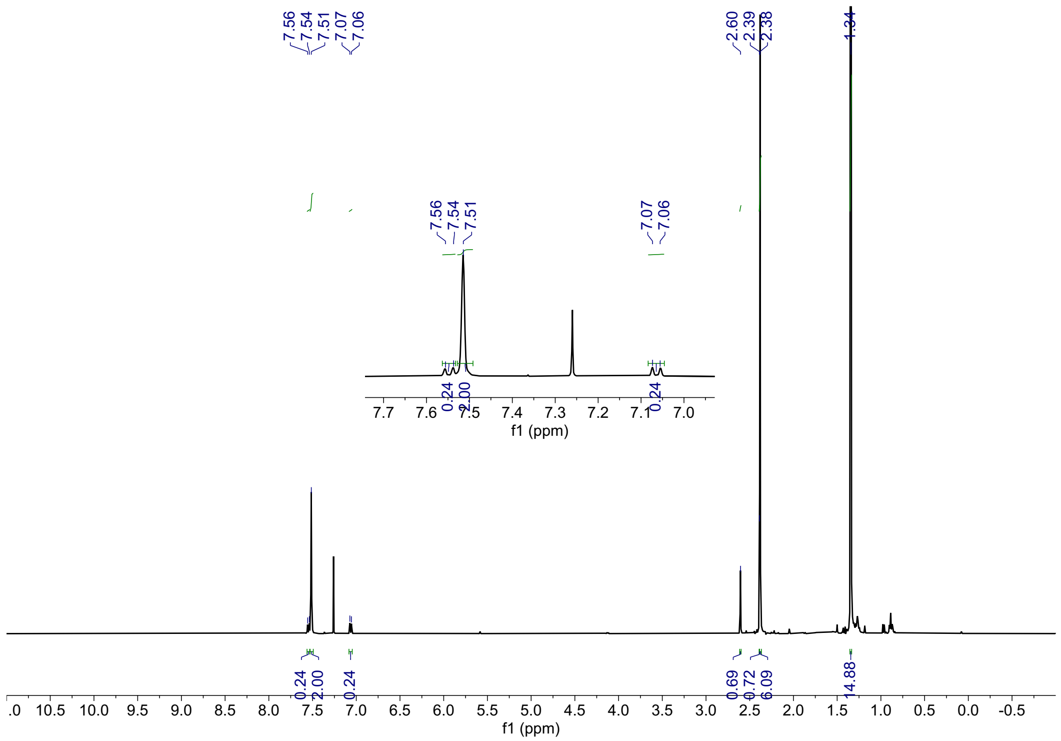

Supplementary Figure 88. ^{1}H NMR spectrum of **30** in $CDCl_3$.

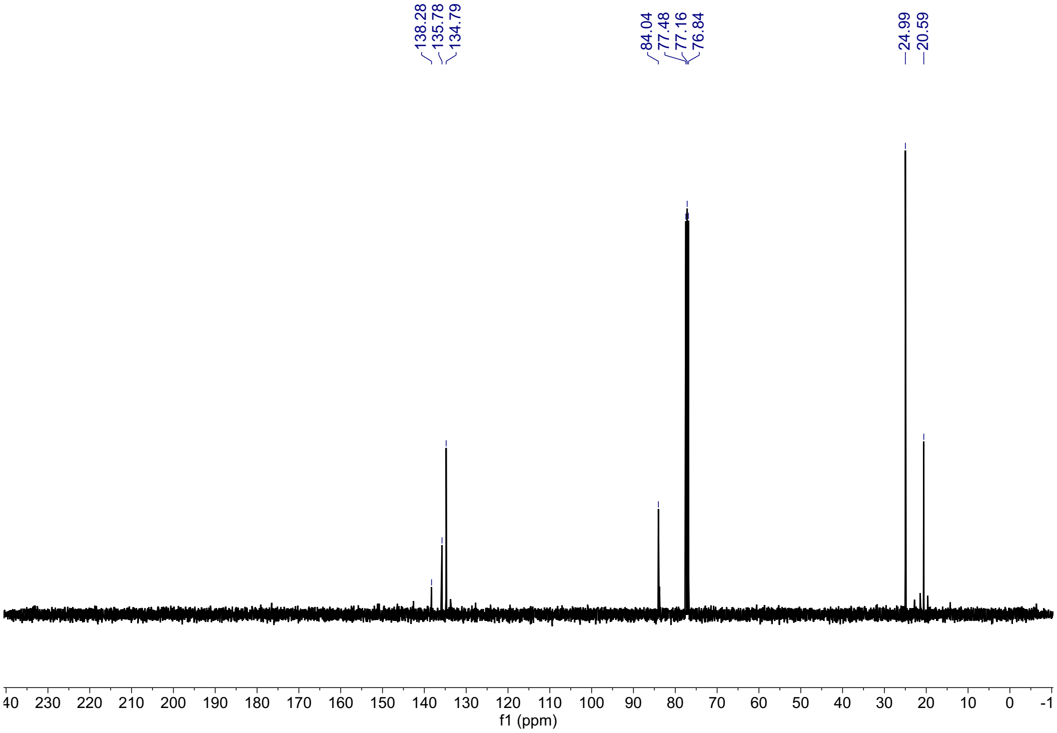

Supplementary Figure 89. ^{13}C NMR spectrum of **30** in $CDCl_3$.

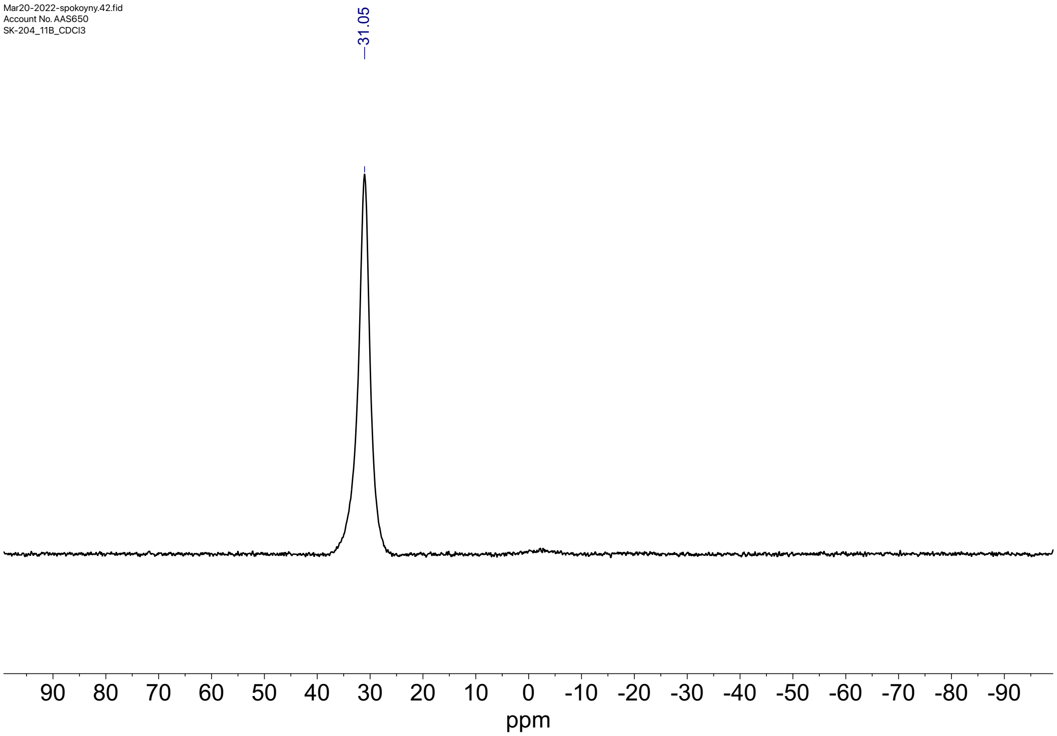

Supplementary Figure 90. ^{11}B NMR spectrum of **30** in $CDCl_3$.

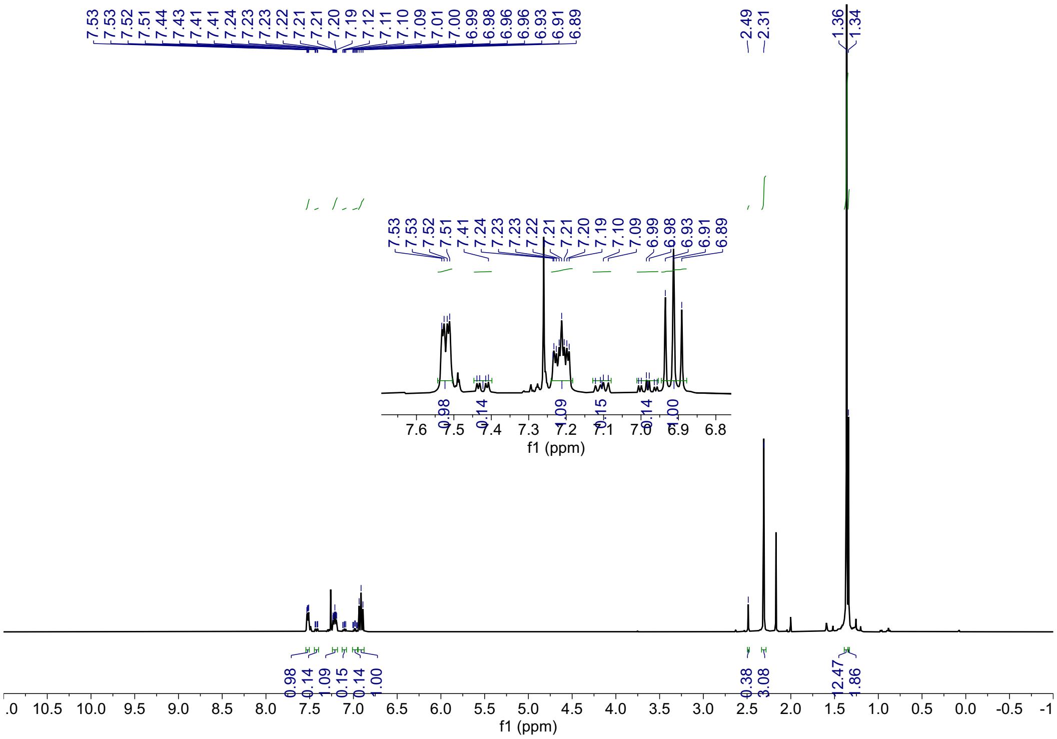

Supplementary Figure 91. ^{1}H NMR spectrum of **31** in $CDCl_3$.

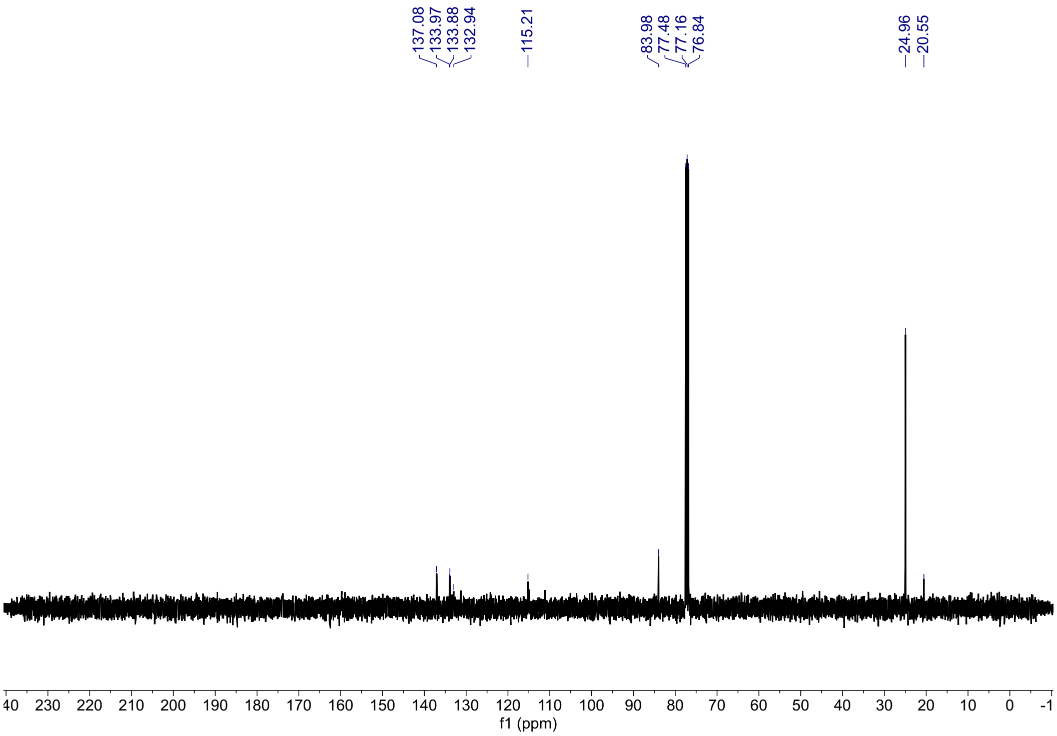

Supplementary Figure 92. ^{13}C NMR spectrum of **31** in $CDCl_3$.

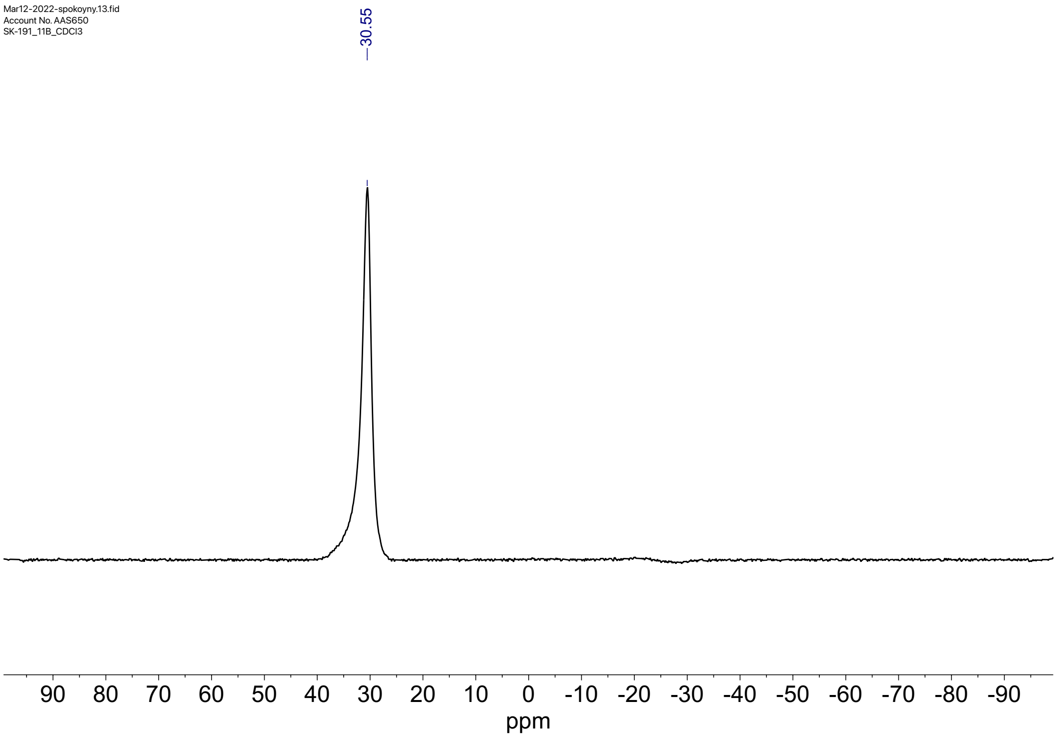

Supplementary Figure 93. ^{11}B NMR spectrum of **31** in $CDCl_3$.

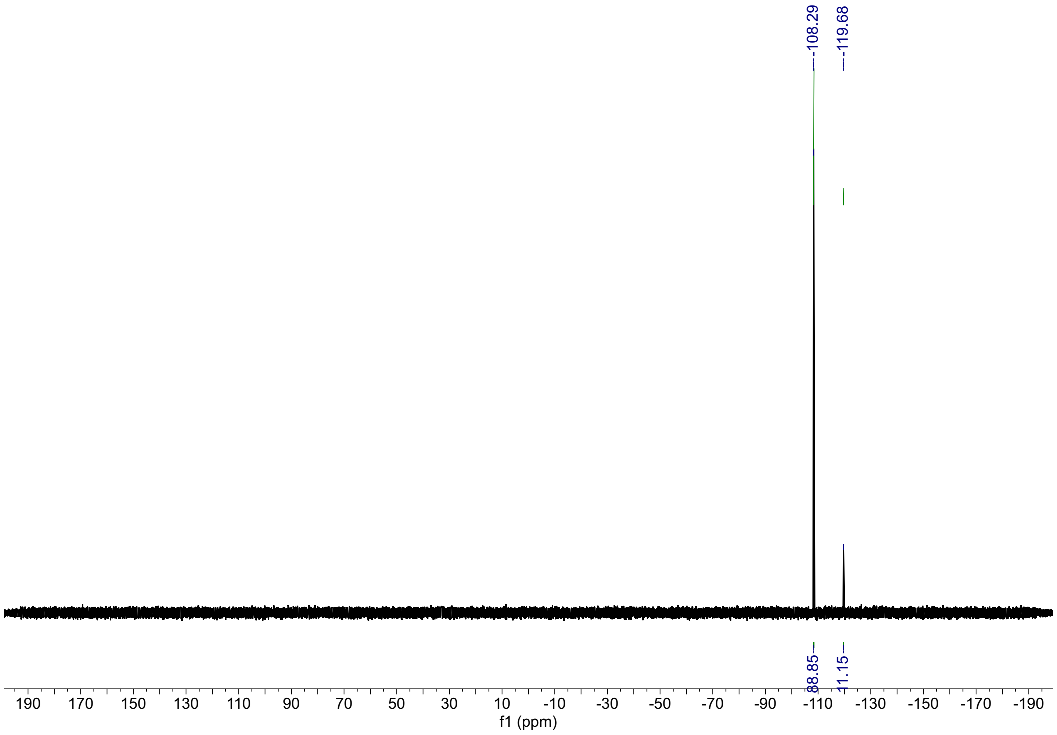

Supplementary Figure 94. ^{19}F NMR spectrum of **31** in $CDCl_3$. (ns = 1)

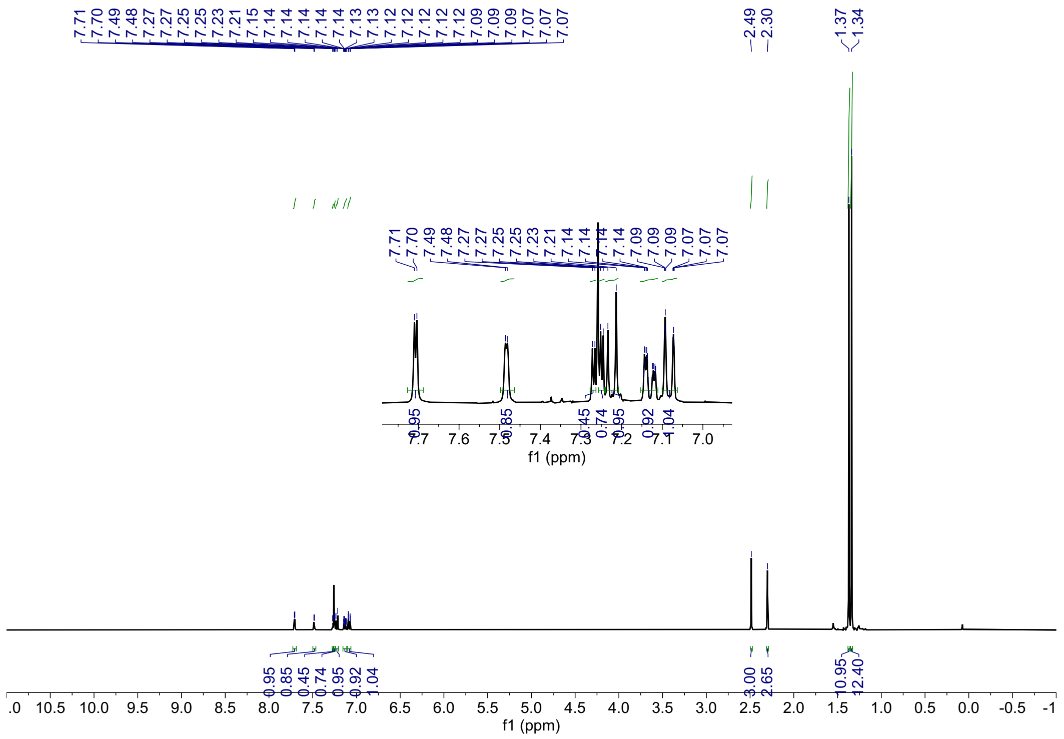

Supplementary Figure 95. ^{1}H NMR spectrum of **32** in $CDCl_3$.

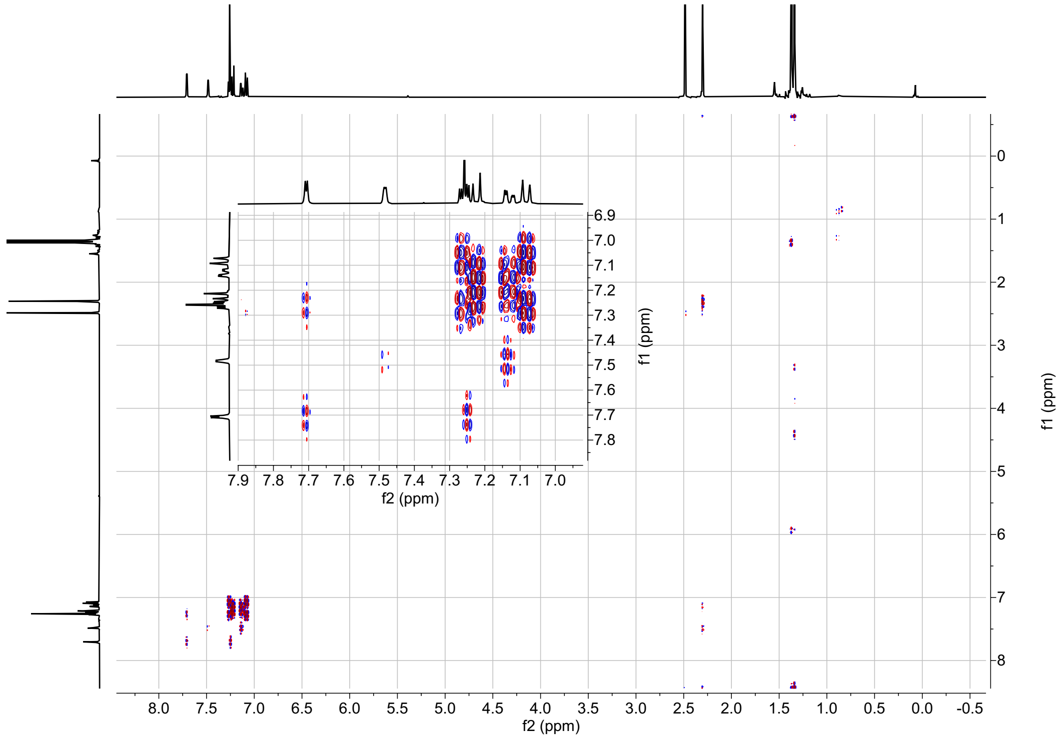

Supplementary Figure 96. ^{1}H-^{1}H COSY NMR spectrum of **32** in $CDCl_3$.

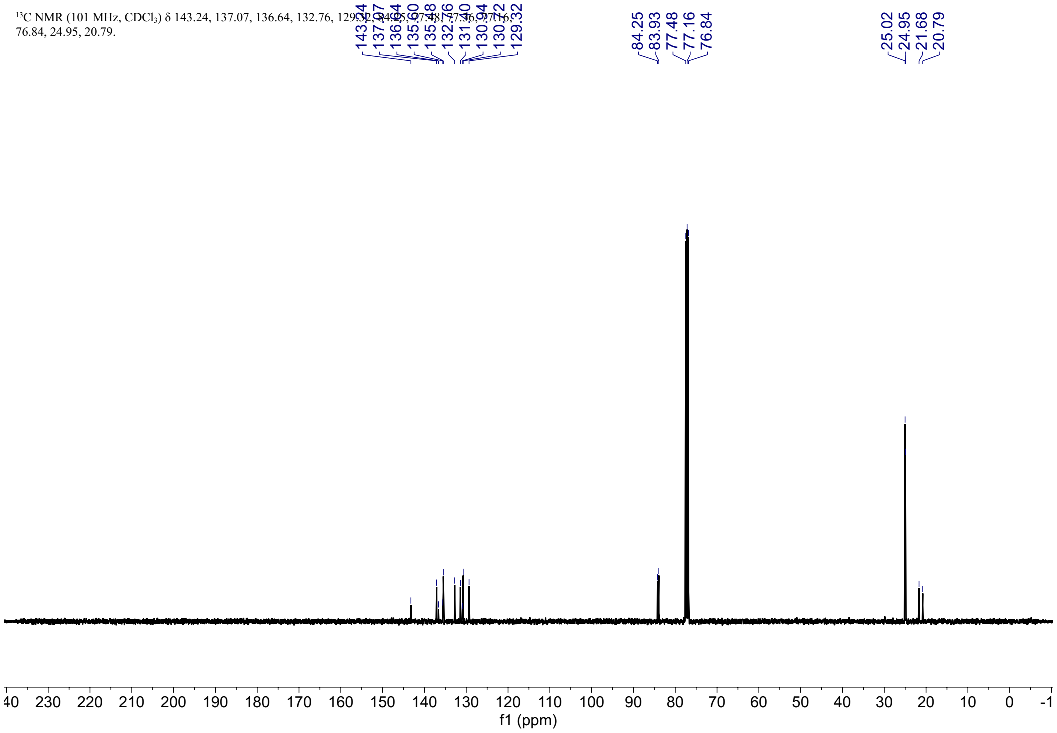

Supplementary Figure 97. ^{13}C NMR spectrum of **32** in $CDCl_3$.

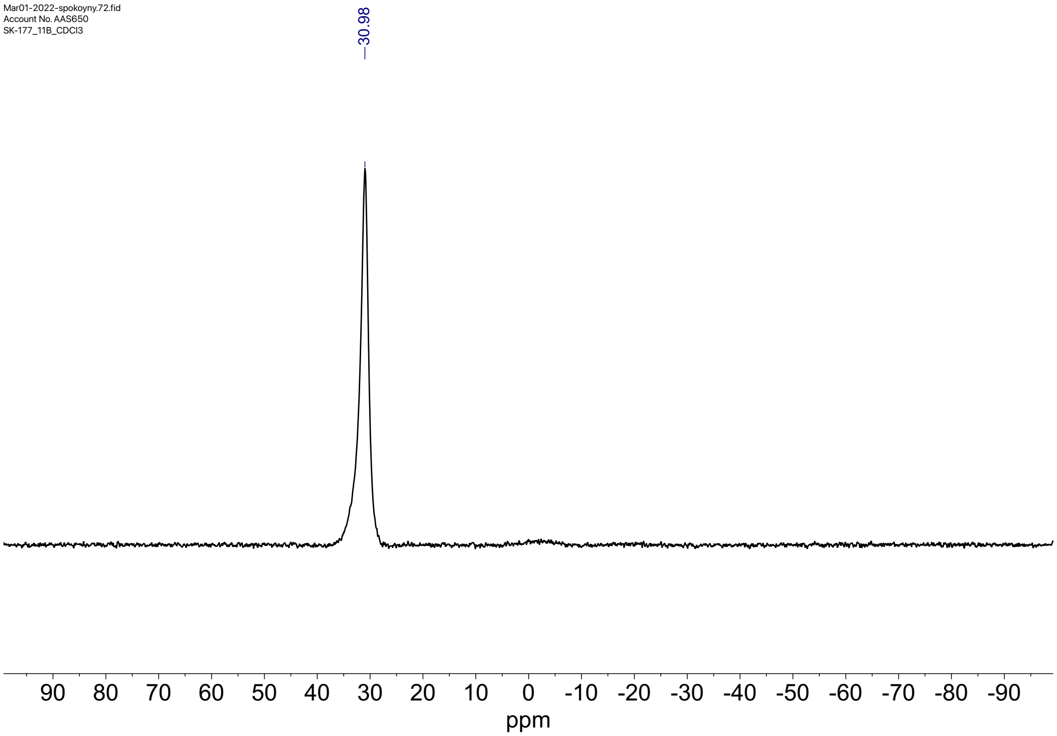

Supplementary Figure 98. ^{11}B NMR spectrum of **32** in $CDCl_3$.

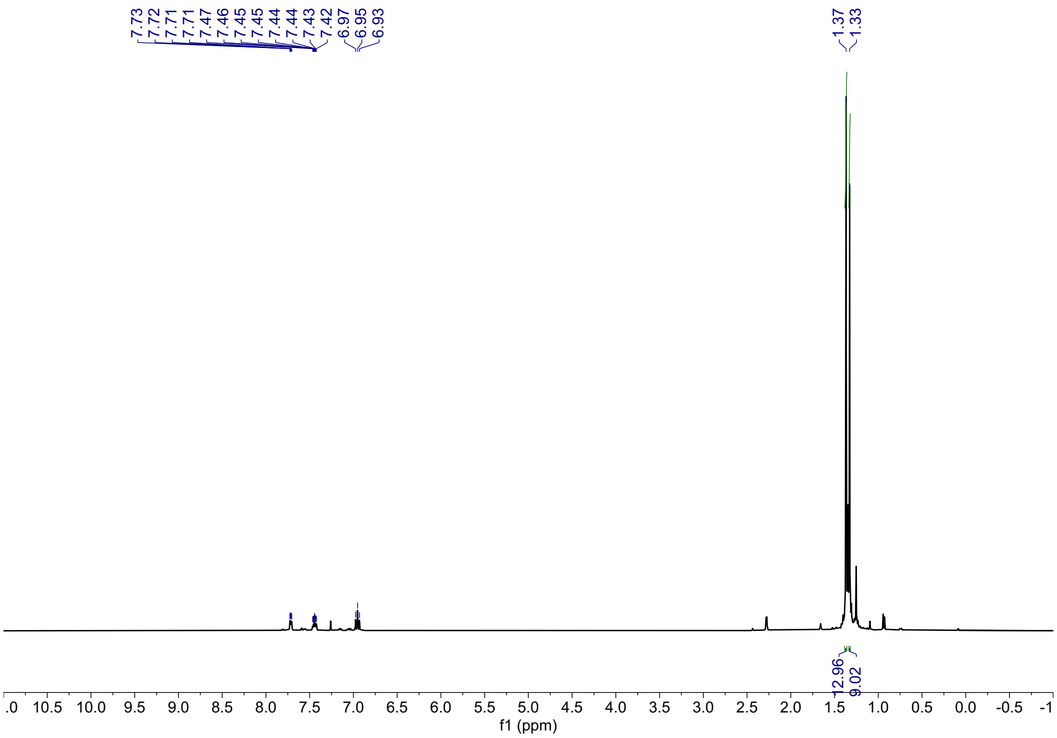

Supplementary Figure 99. ^{1}H NMR spectrum of **33** in $CDCl_3$.

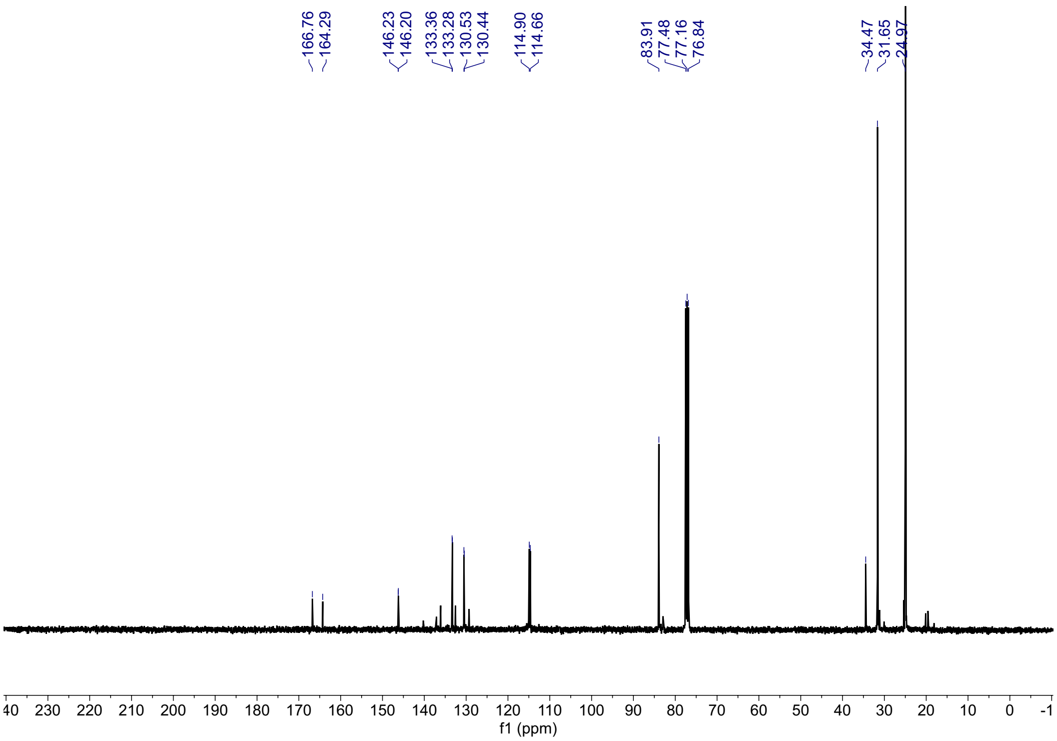

Supplementary Figure 100. ^{13}C NMR spectrum of **33** in $CDCl_3$.

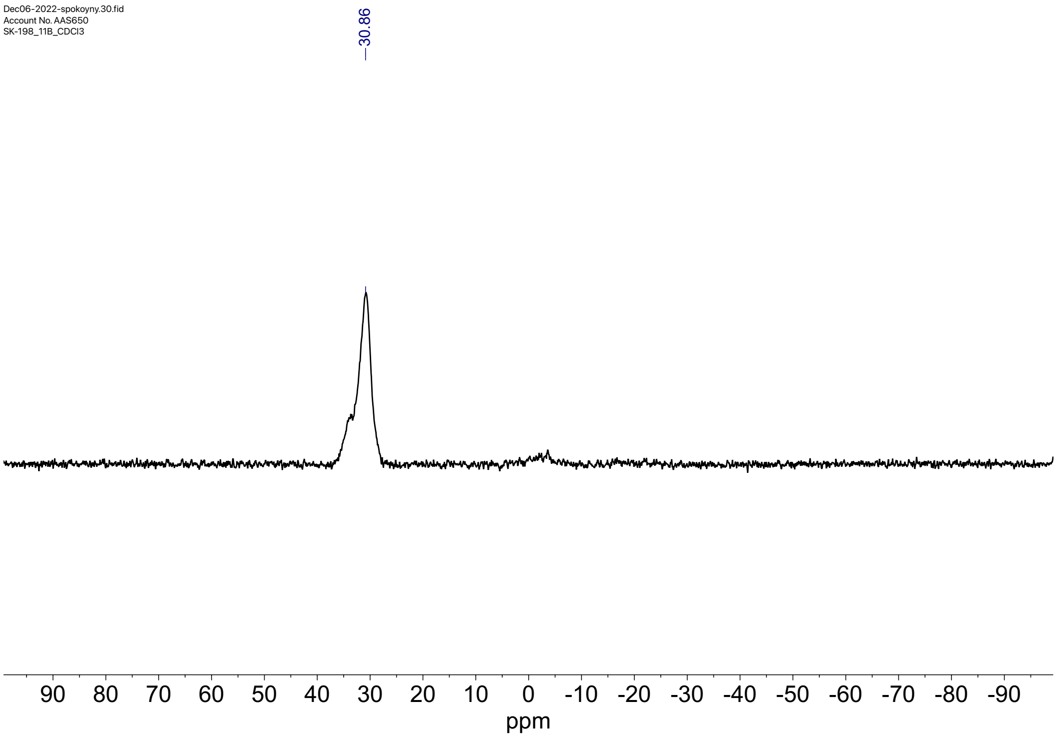

Supplementary Figure 101. ^{11}B NMR spectrum of **33** in $CDCl_3$.

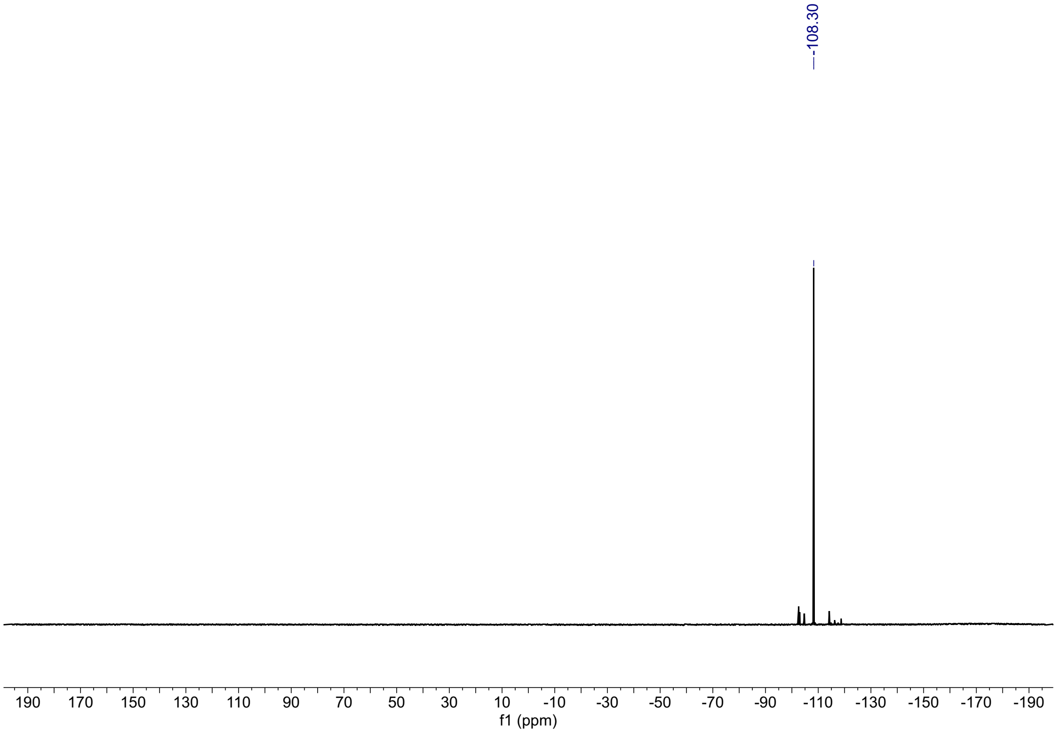

Supplementary Figure 102. ^{19}F{^{1}H} NMR spectrum of **33** in $CDCl_3$.

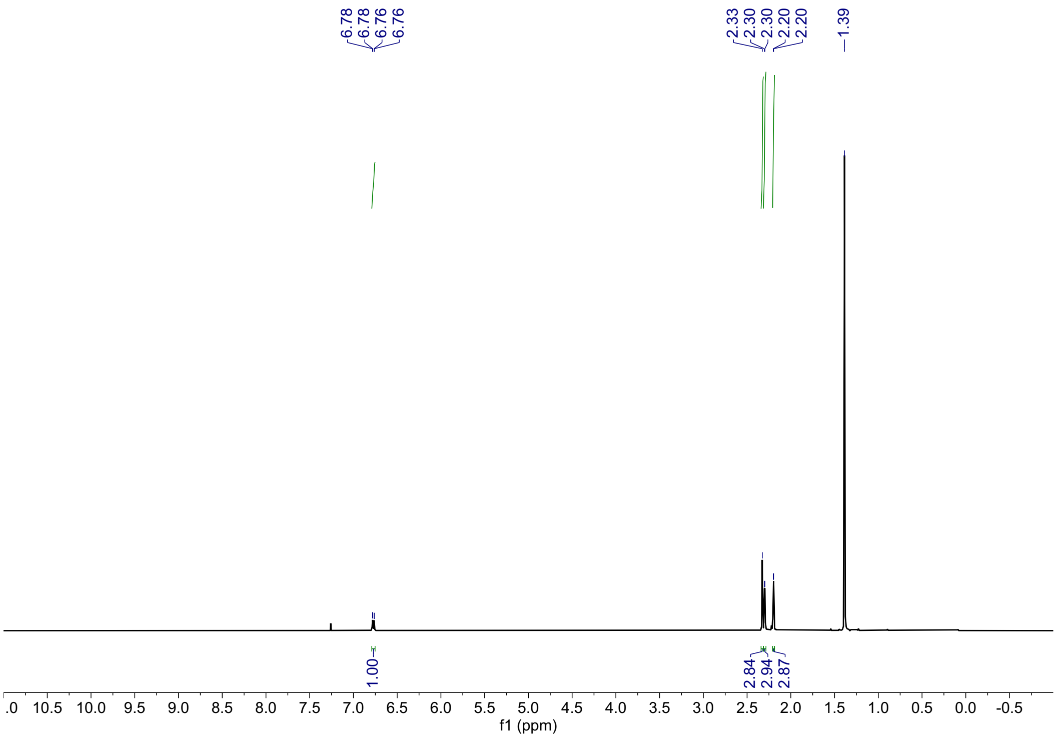

Supplementary Figure 103. ^{1}H NMR spectrum of **34** in $CDCl_3$.

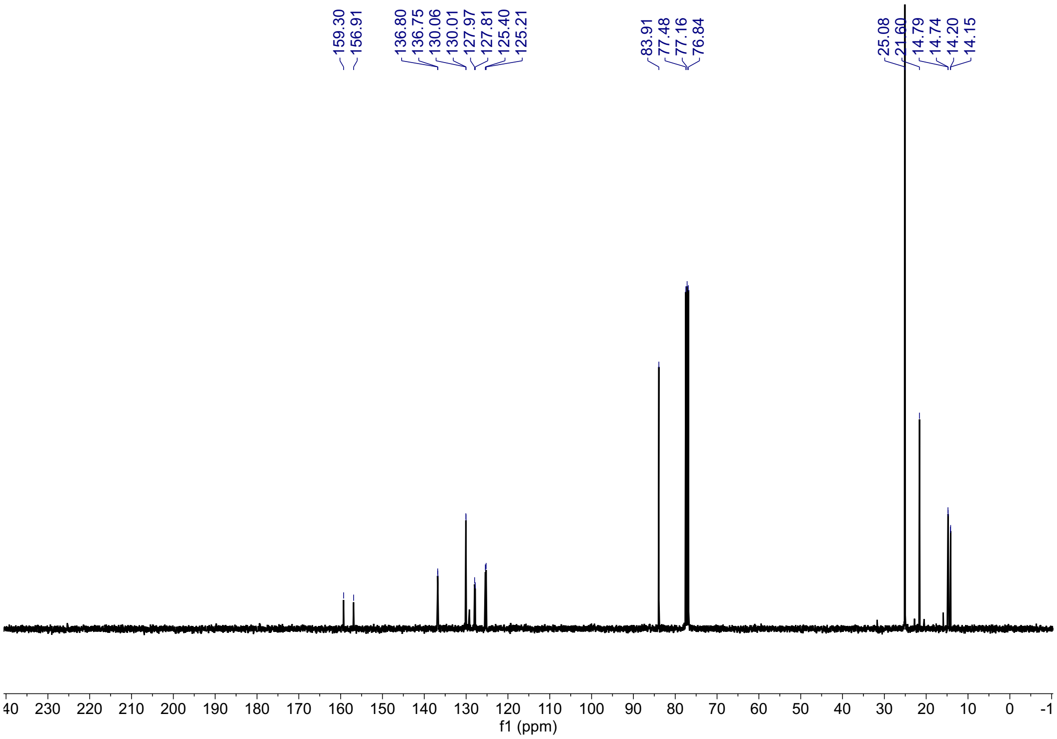

Supplementary Figure 104. ^{13}C NMR spectrum of **34** in $CDCl_3$.

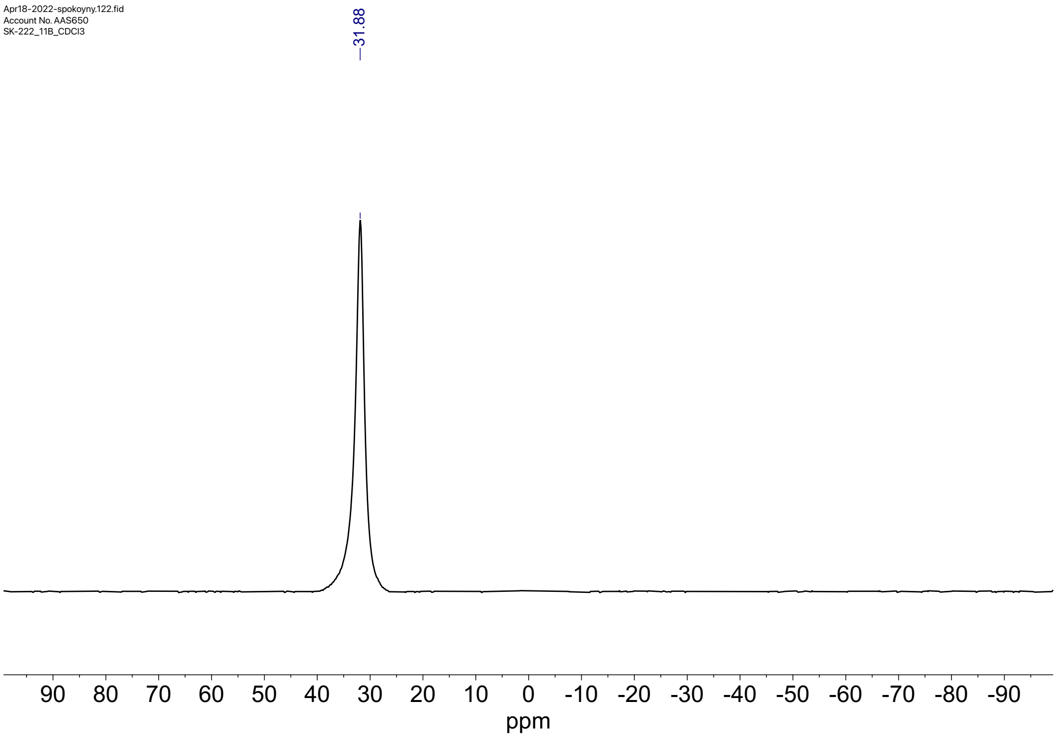

Supplementary Figure 105. ^{11}B NMR spectrum of **34** in $CDCl_3$.

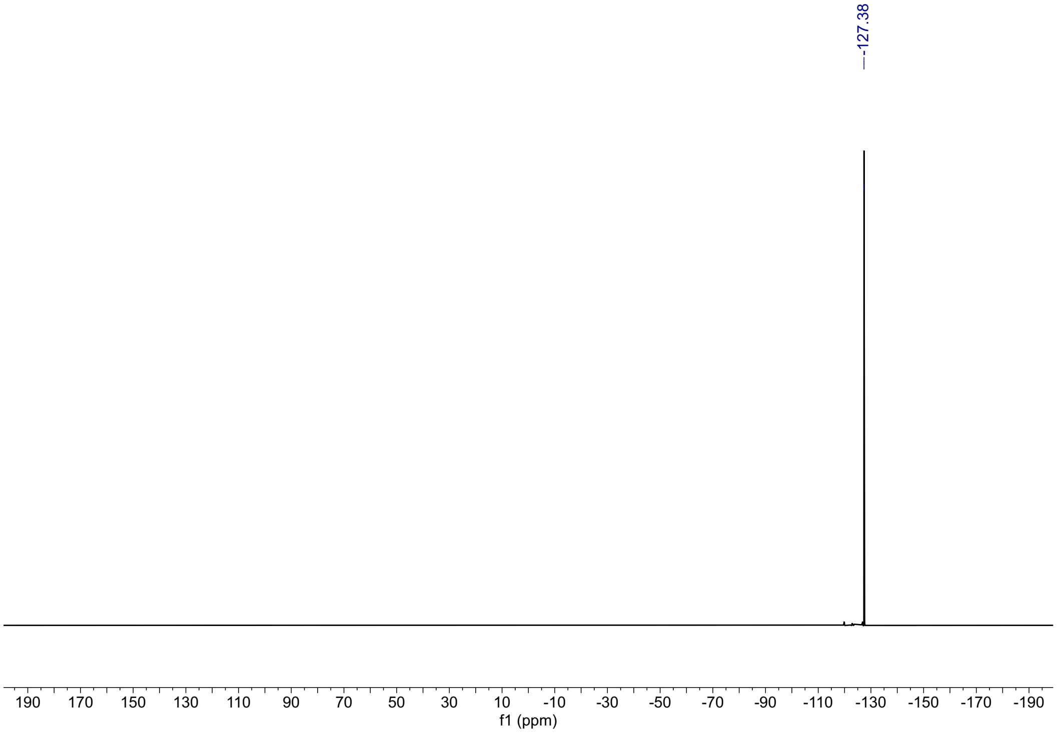

Supplementary Figure 106. $^{19}F\{^1H\}$ NMR spectrum of **34** in $CDCl_3$.

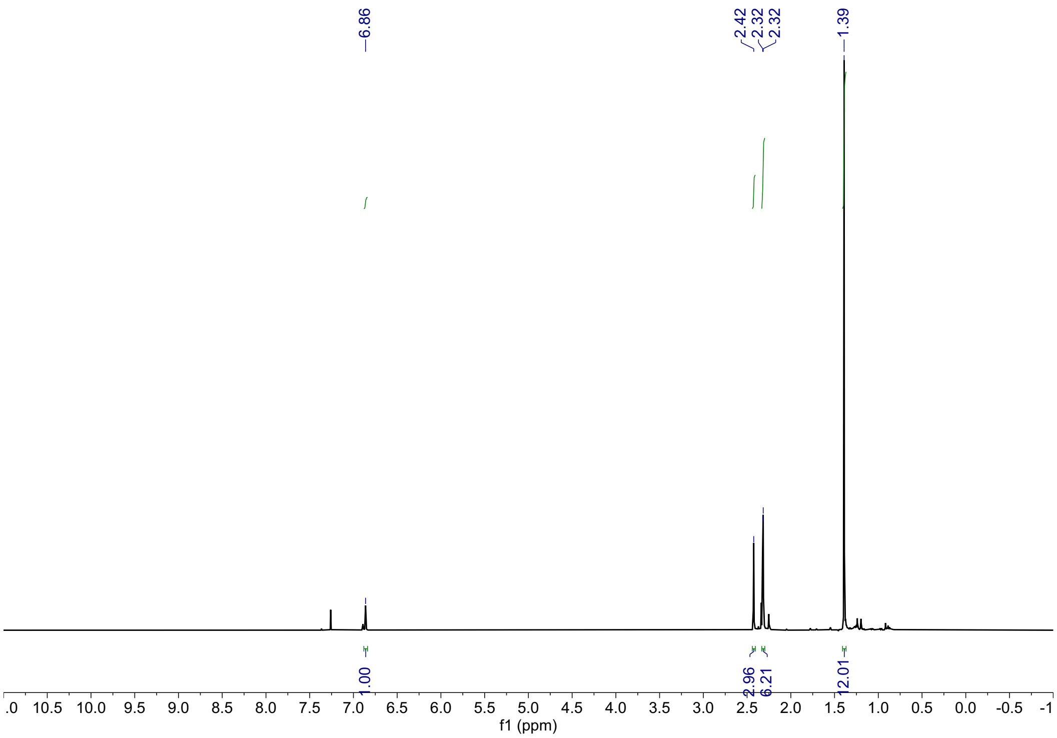

Supplementary Figure 107. ^{1}H NMR spectrum of **35** in $CDCl_3$.

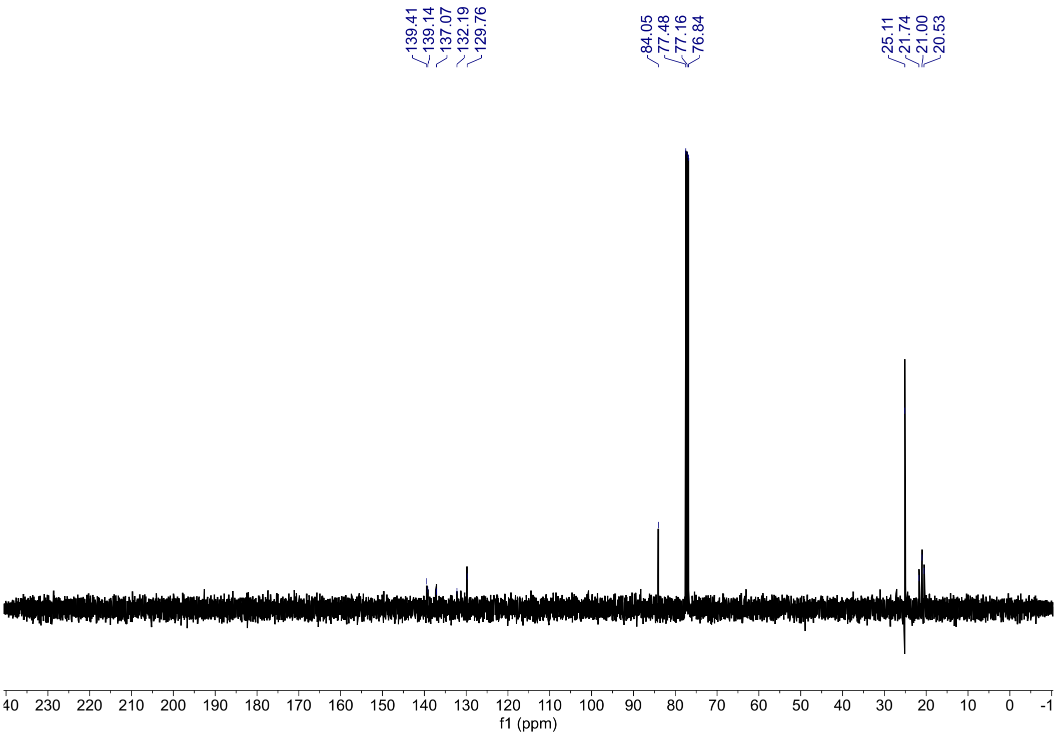

Supplementary Figure 108. ^{13}C NMR spectrum of **35** in $CDCl_3$.

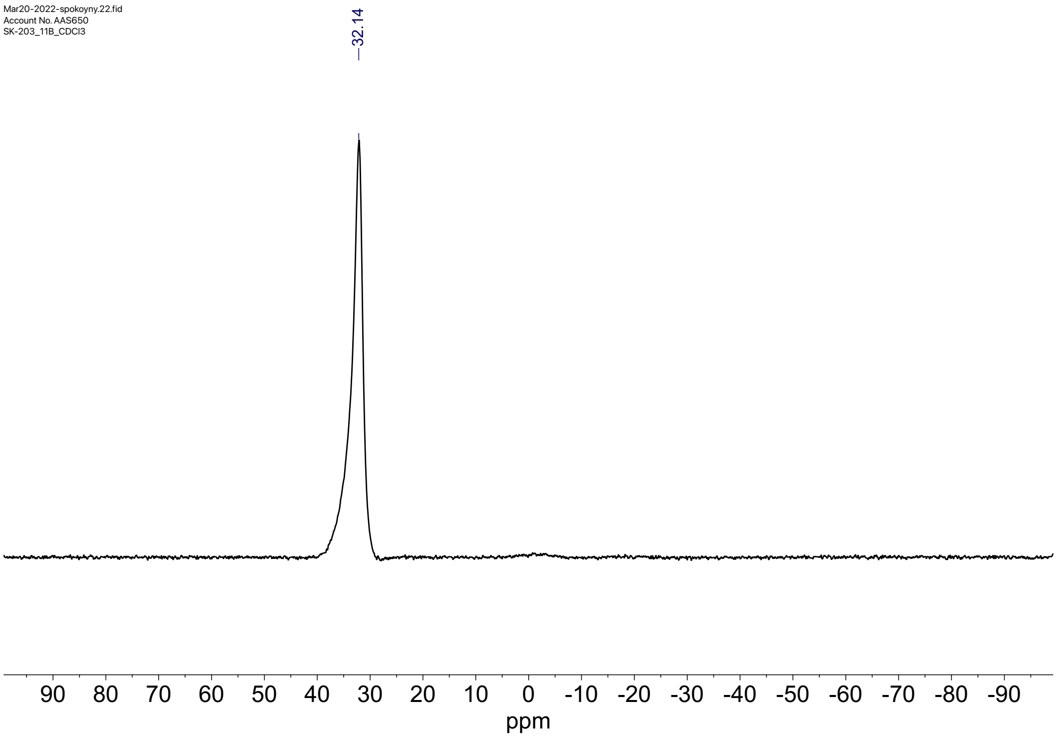

Supplementary Figure 109. ^{11}B NMR spectrum of **35** in $CDCl_3$.

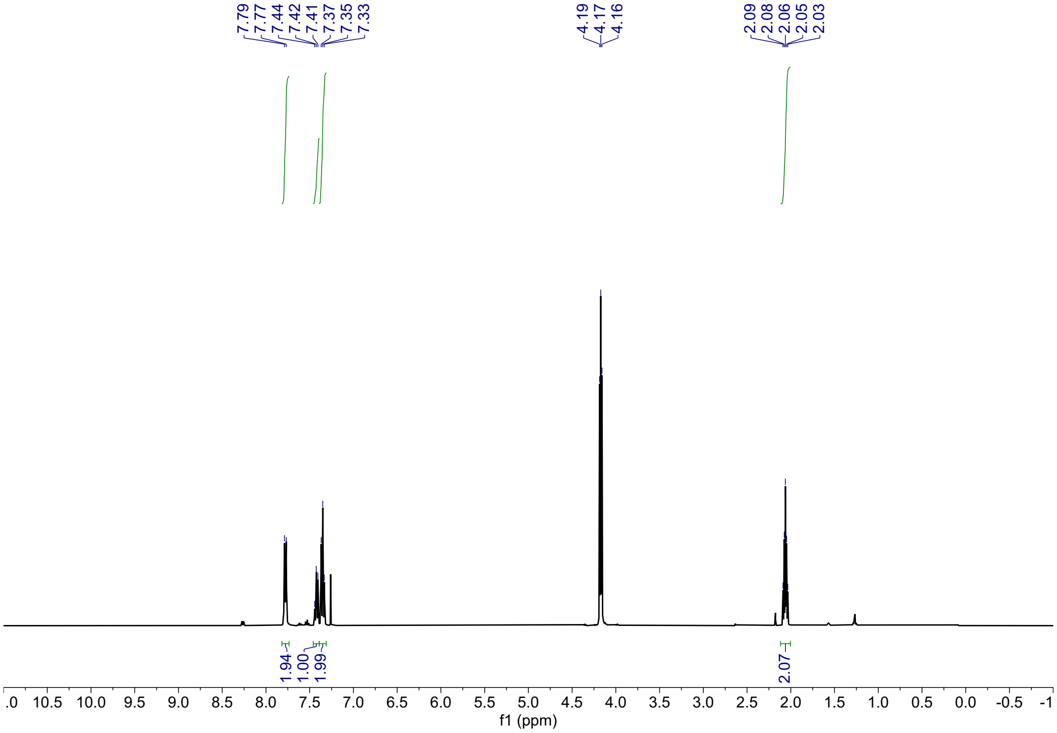

Supplementary Figure 110. ^{1}H NMR spectrum of **36** in $CDCl_3$.

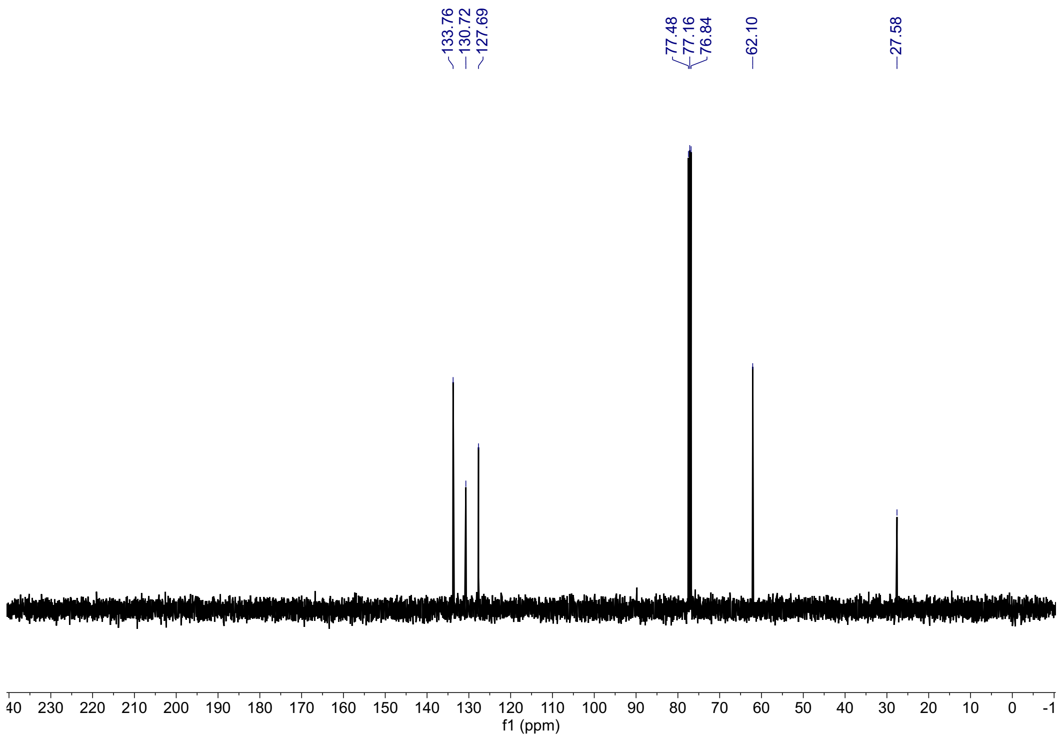

Supplementary Figure 111. ^{13}C NMR spectrum of **36** in $CDCl_3$.

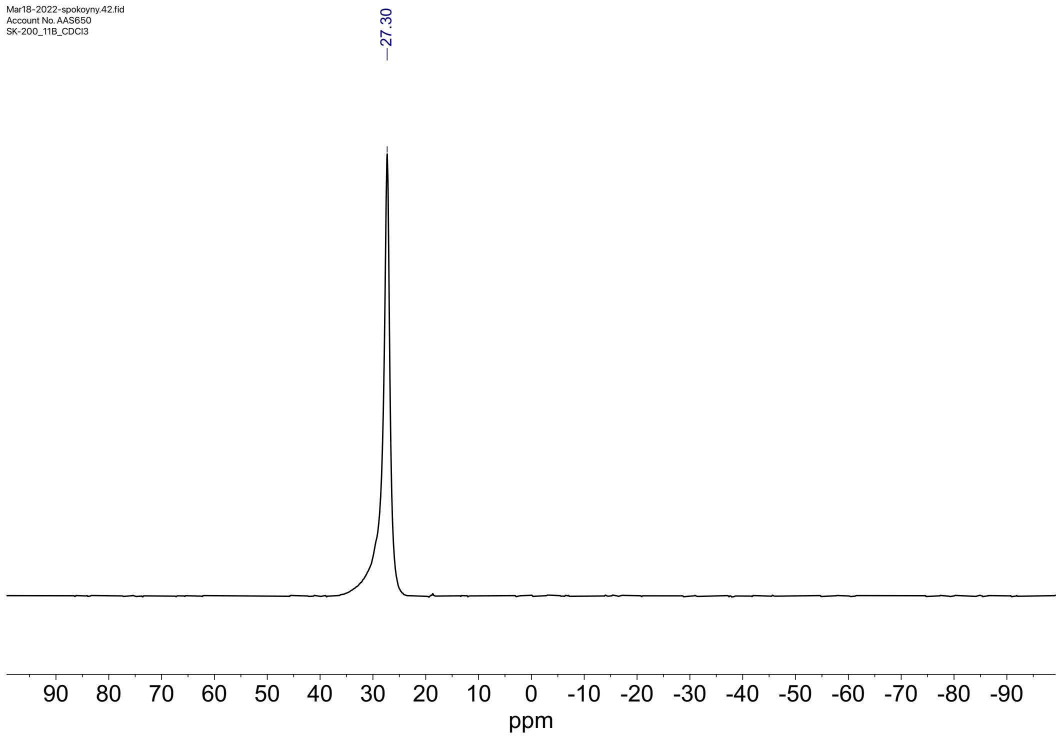

Supplementary Figure 112. ^{11}B NMR spectrum of **36** in $CDCl_3$.

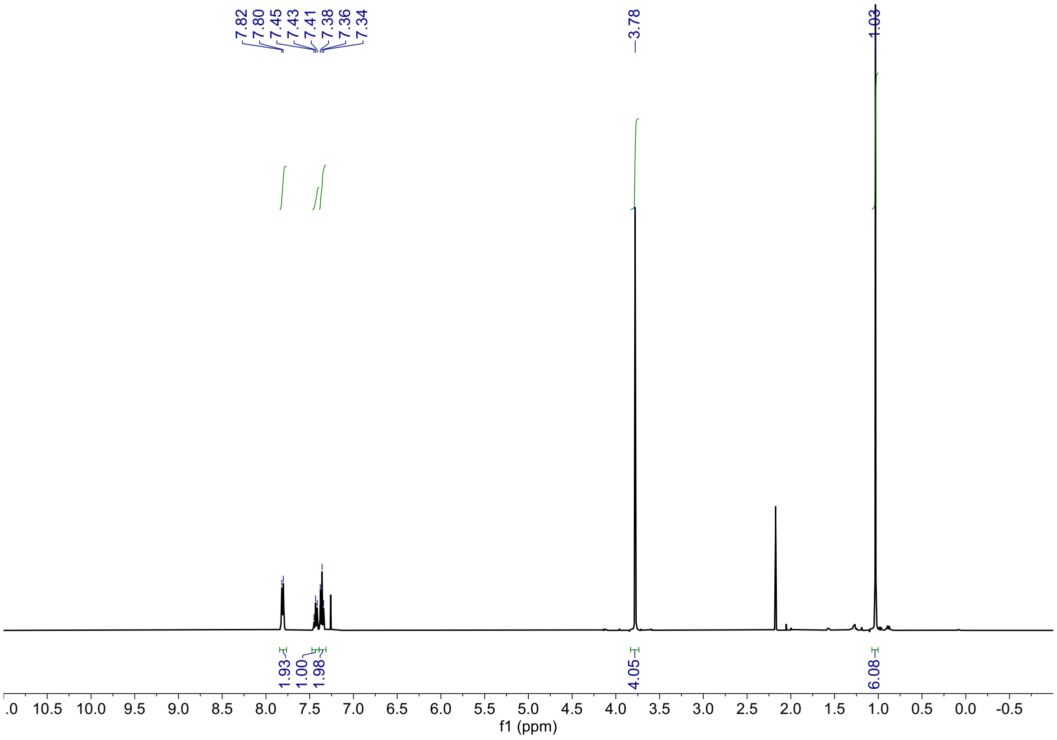

Supplementary Figure 113. ^{1}H NMR spectrum of **37** in $CDCl_3$.

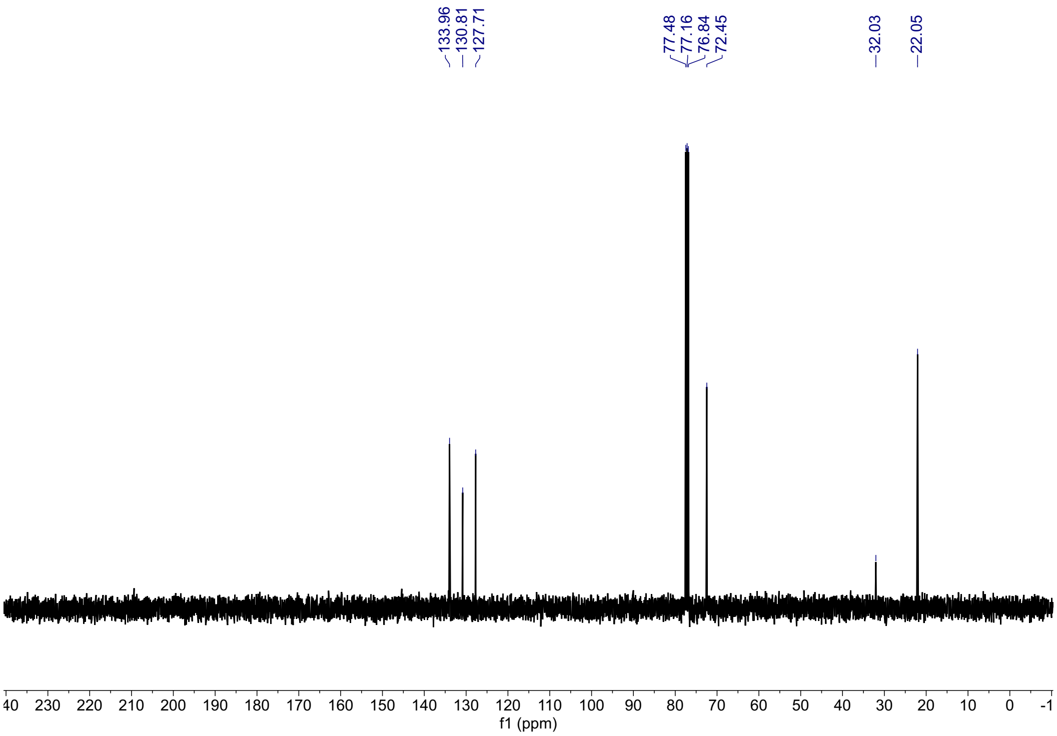

Supplementary Figure 114. ^{13}C NMR spectrum of **37** in $CDCl_3$.

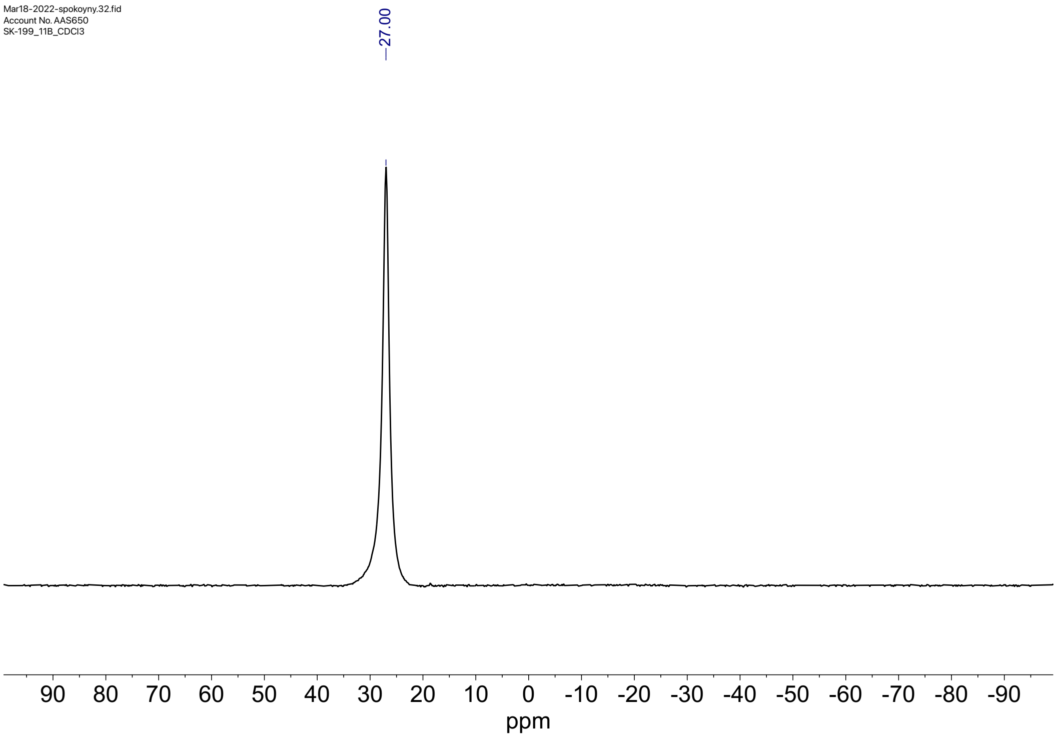

Supplementary Figure 115. ^{11}B NMR spectrum of **37** in $CDCl_3$.

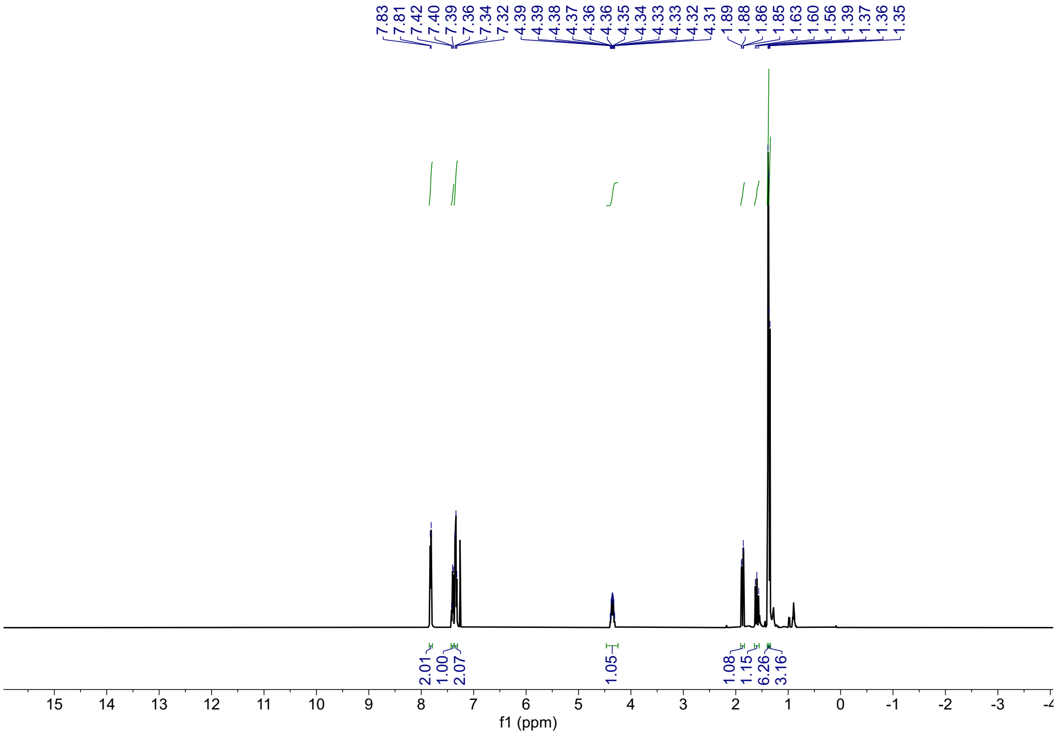

Supplementary Figure 116. ^{1}H NMR spectrum of **38** in $CDCl_3$.

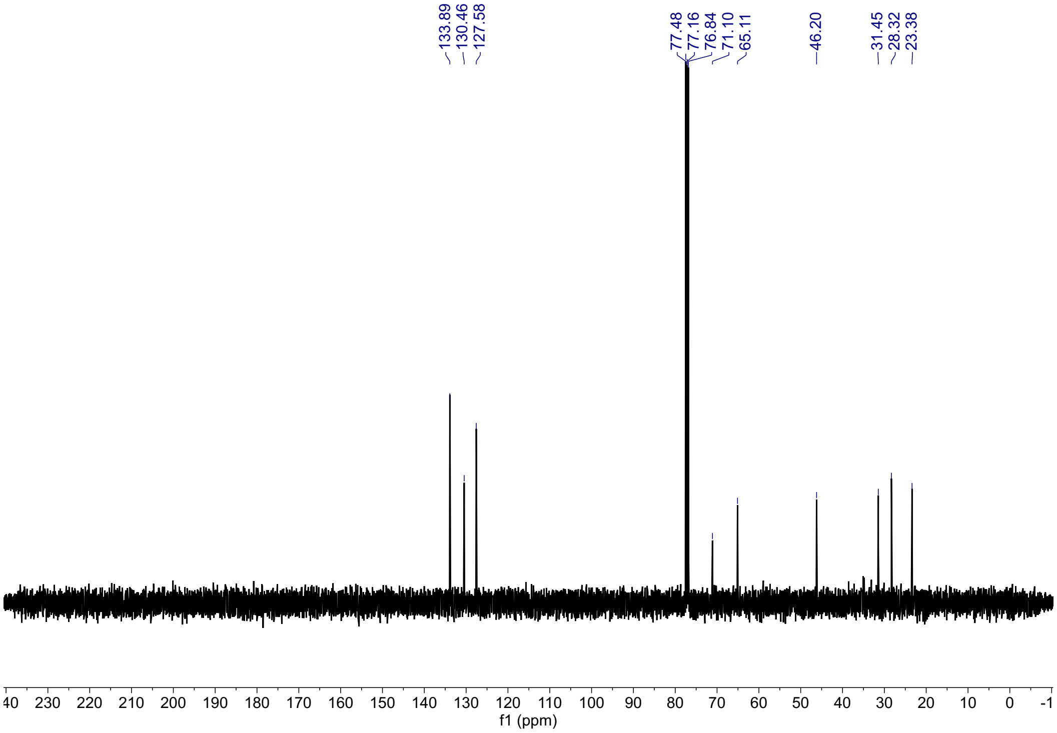

Supplementary Figure 117. ^{13}C NMR spectrum of **38** in $CDCl_3$.

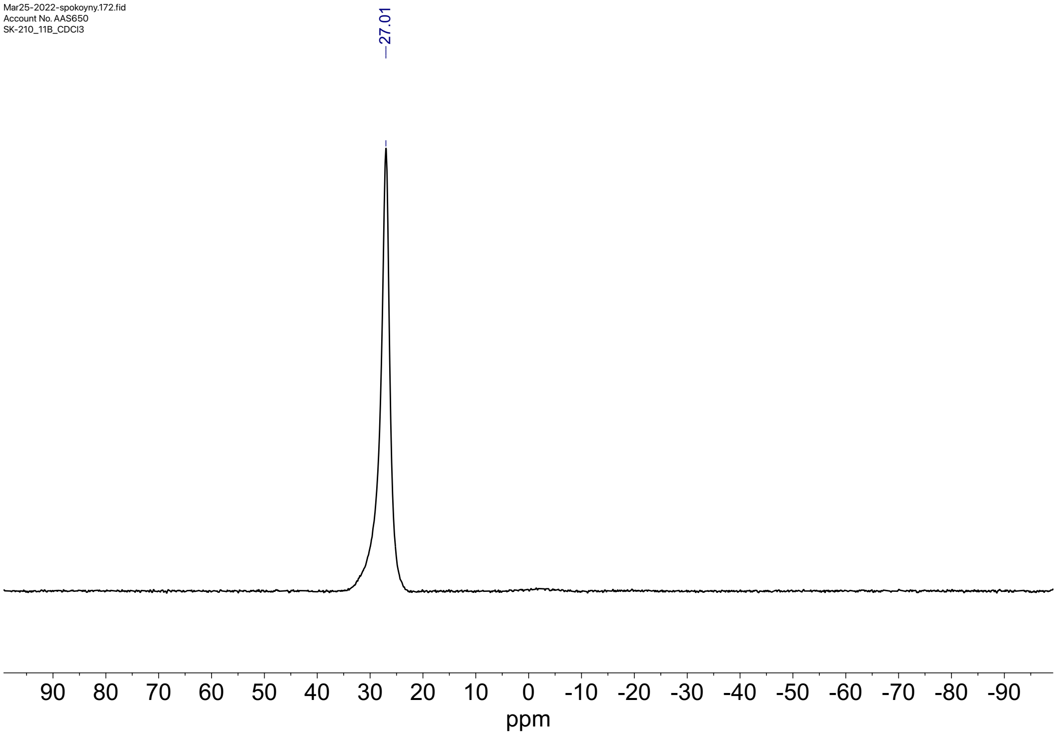

Supplementary Figure 118. ^{11}B NMR spectrum of **38** in $CDCl_3$.

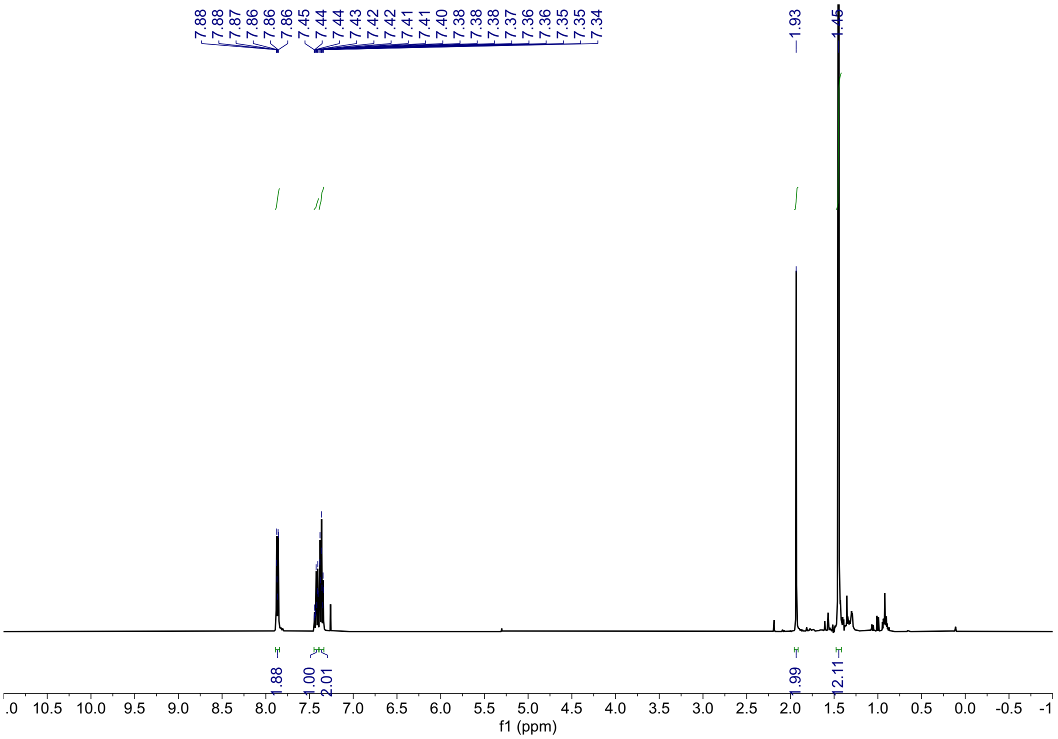

Supplementary Figure 119. ^{1}H NMR spectrum of **39** in $CDCl_3$.

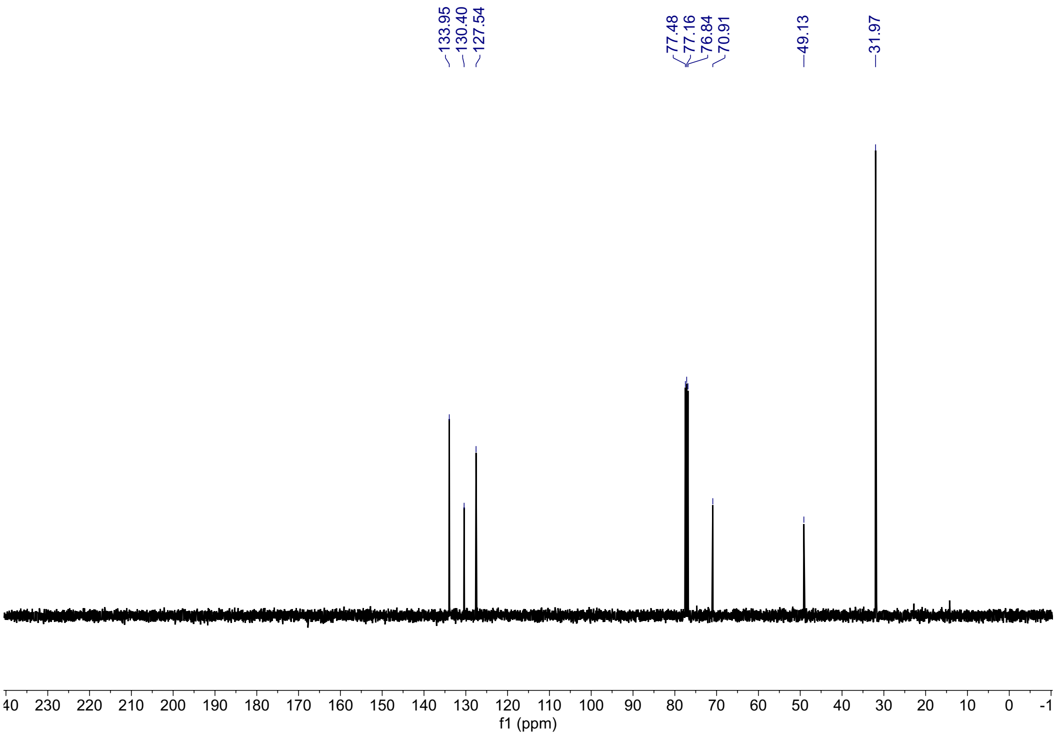

Supplementary Figure 120. ^{13}C NMR spectrum of **39** in $CDCl_3$.

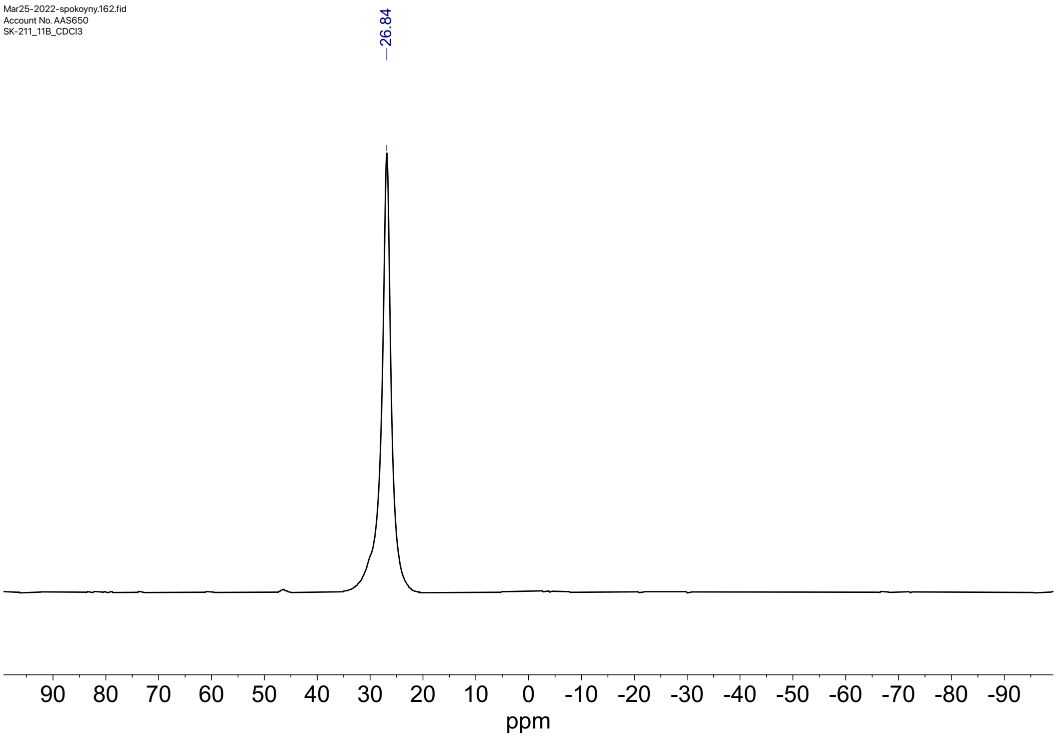

Supplementary Figure 121. ^{11}B NMR spectrum of **39** in $CDCl_3$.

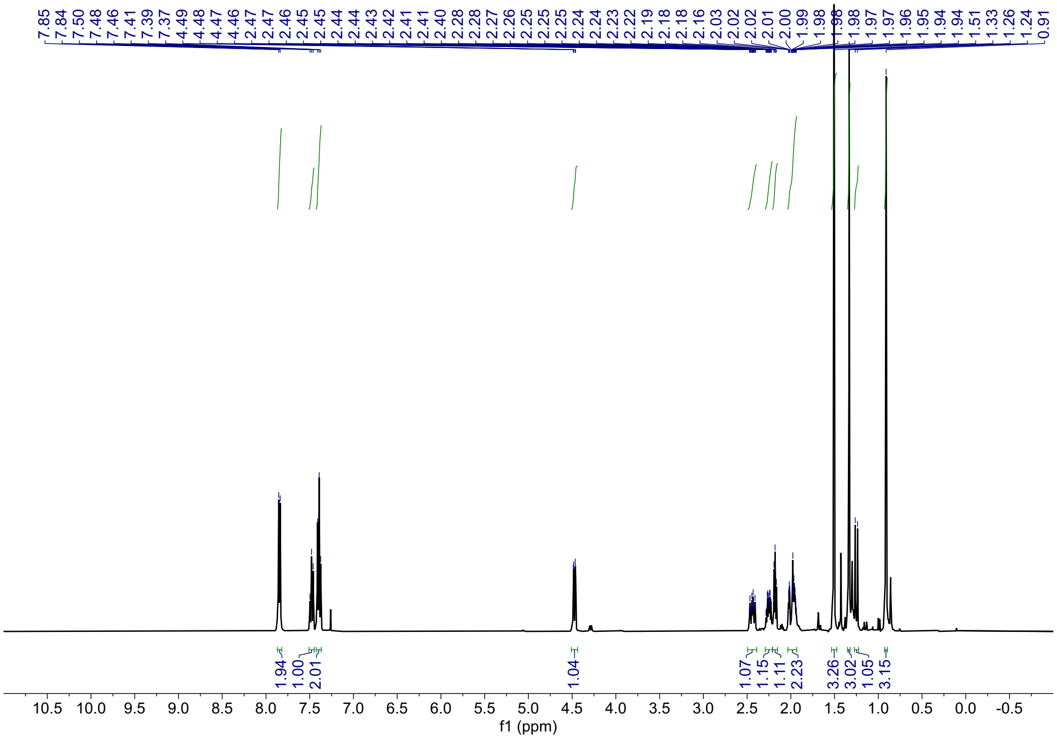

Supplementary Figure 122. ^{1}H NMR spectrum of **40** in $CDCl_3$.

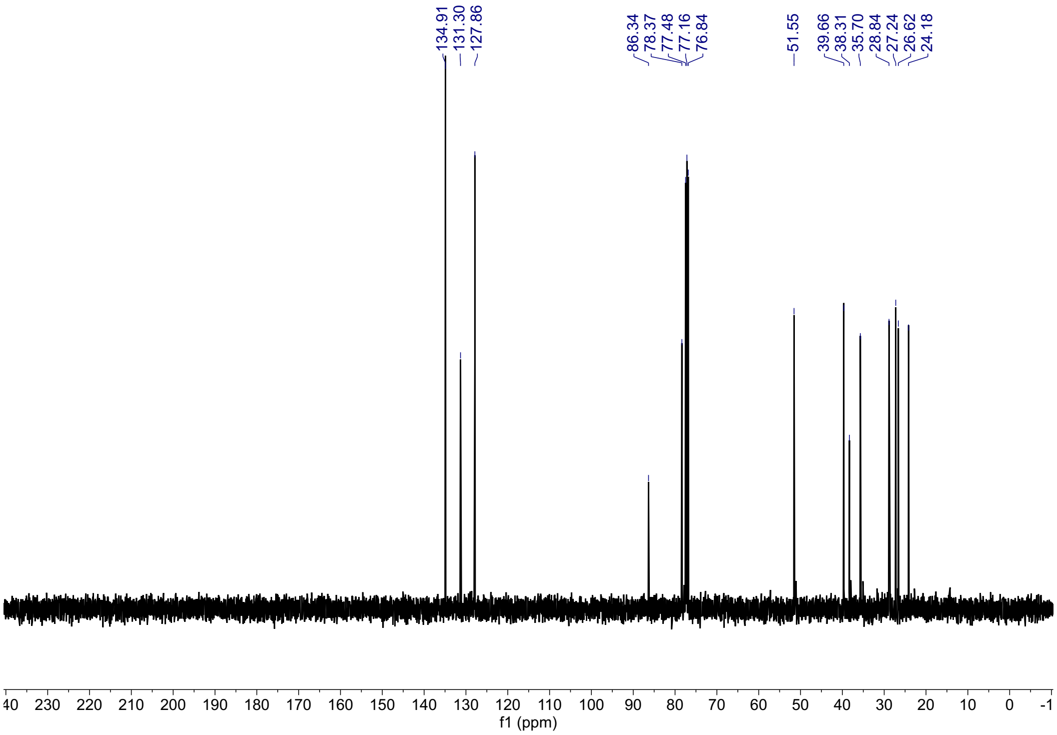

Supplementary Figure 123. ^{13}C NMR spectrum of **40** in $CDCl_3$.

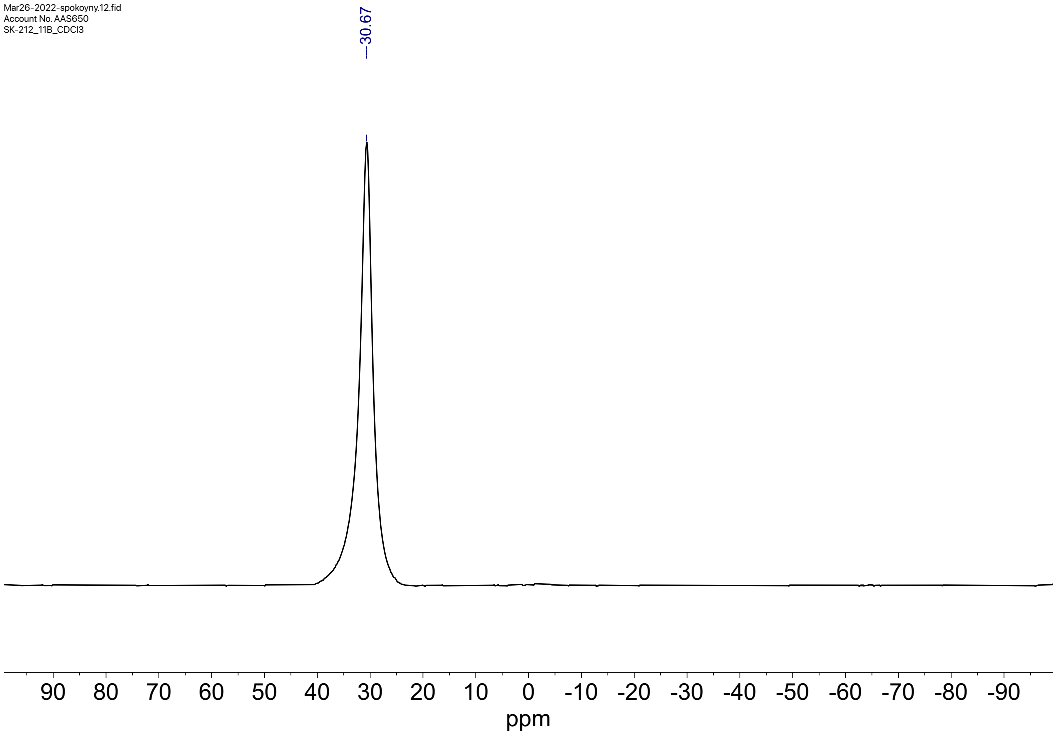

Supplementary Figure 124. ^{11}B NMR spectrum of **40** in $CDCl_3$.

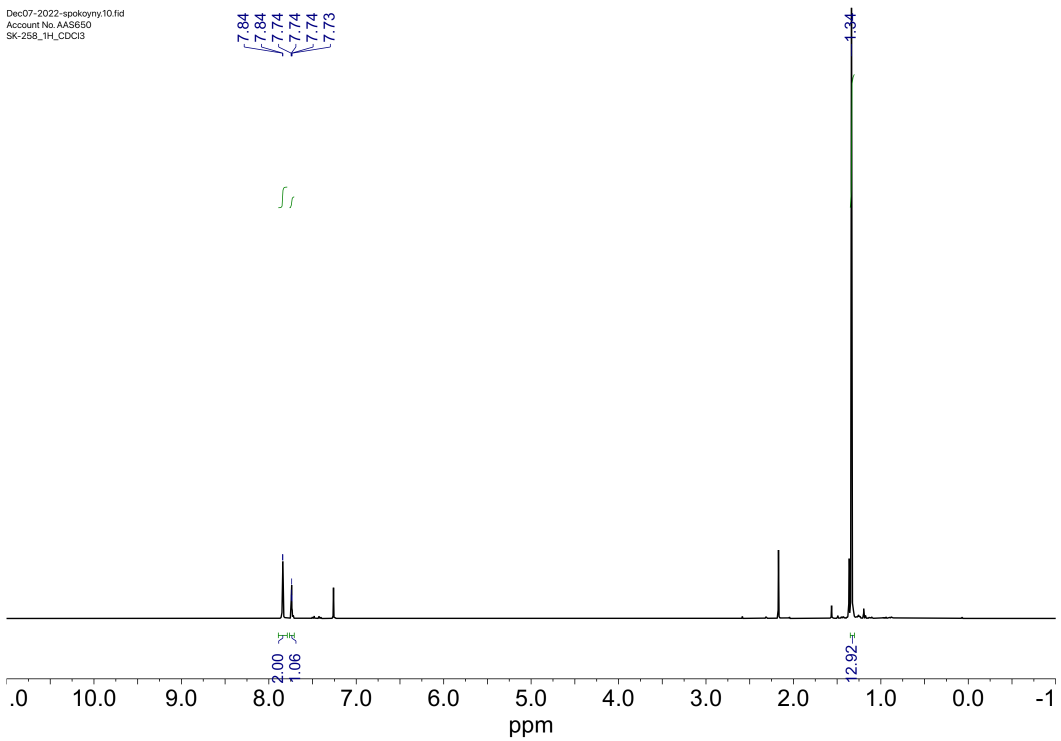

Supplementary Figure 125. ^{1}H NMR spectrum of **43** in $CDCl_3$.

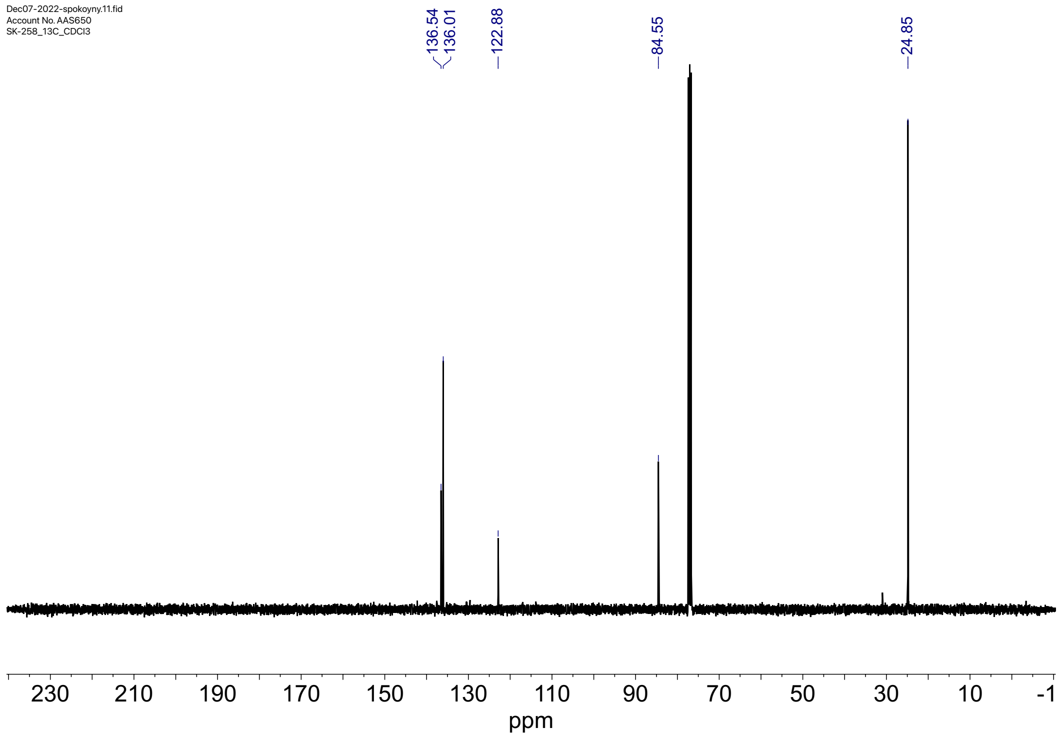

Supplementary Figure 126. ^{13}C NMR spectrum of **43** in $CDCl_3$.

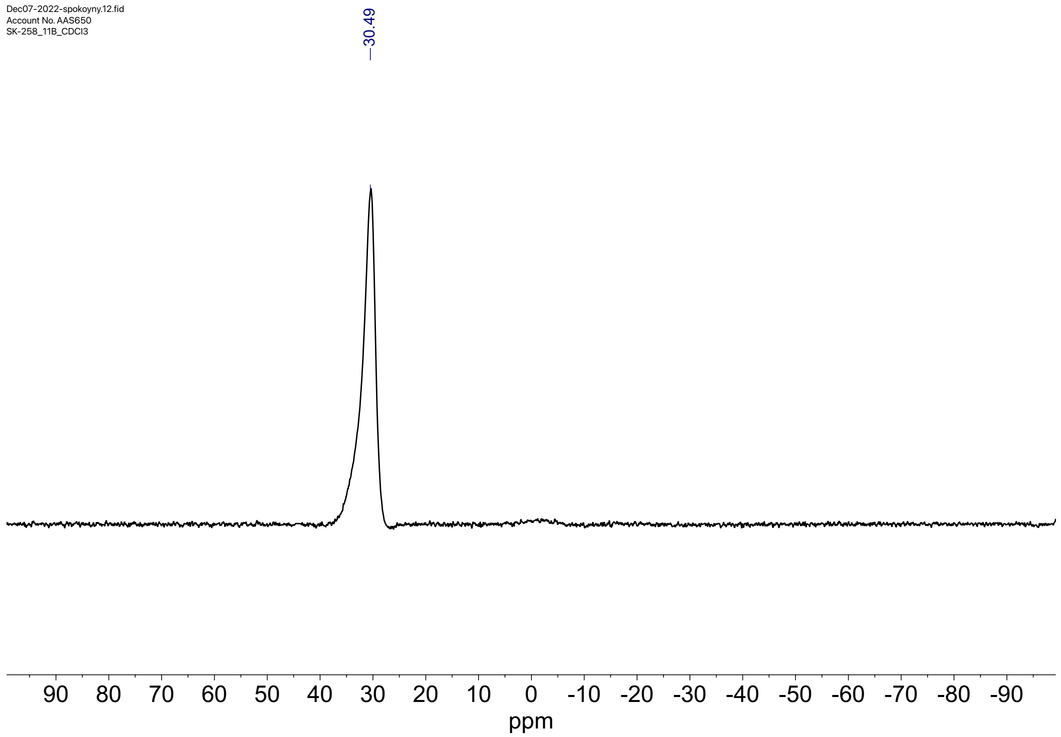

Supplementary Figure 127. ^{11}B NMR spectrum of **43** in $CDCl_3$.

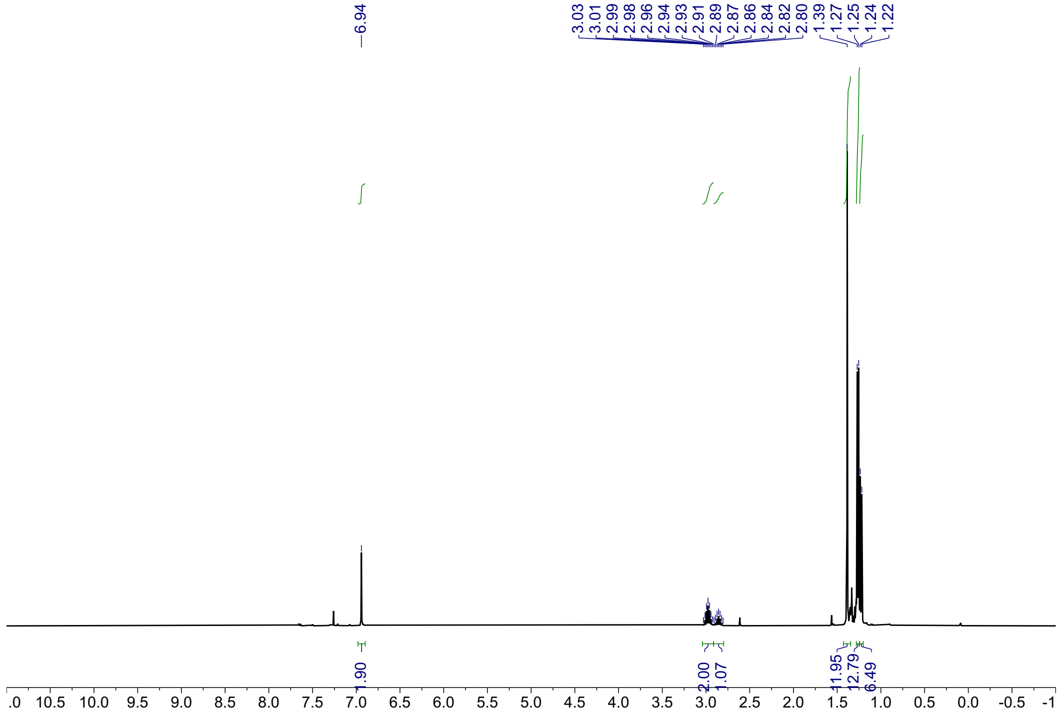

Supplementary Figure 128. ^{1}H NMR spectrum of **45** in $CDCl_3$.

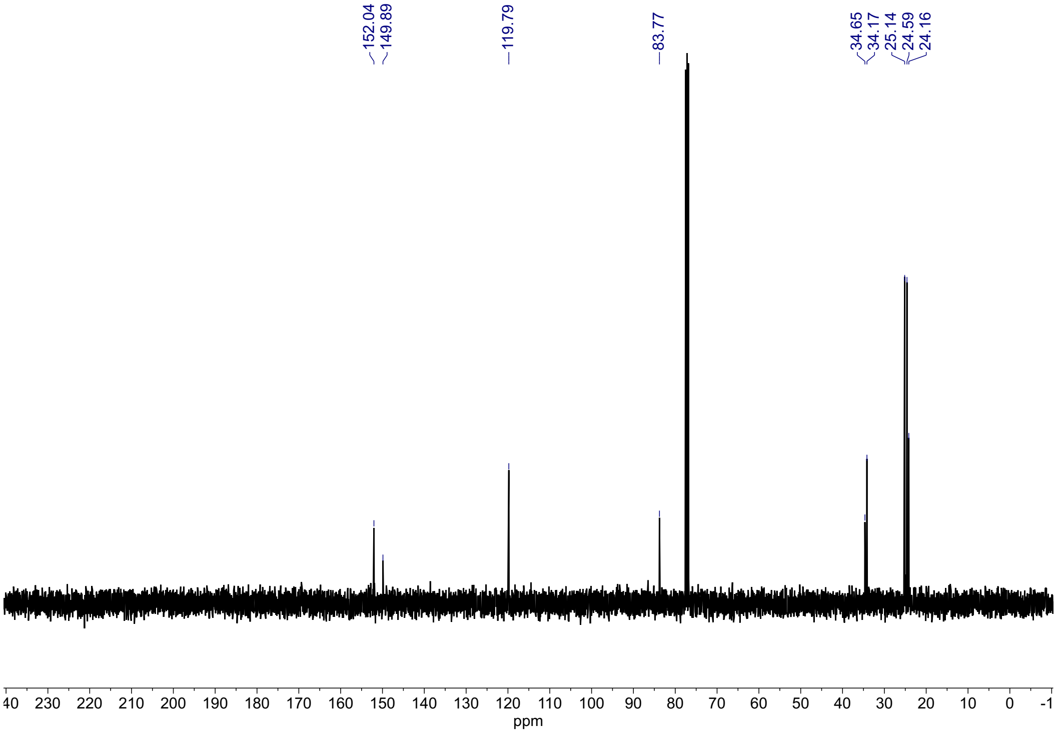

Supplementary Figure 129. ^{13}C NMR spectrum of **45** in $CDCl_3$.

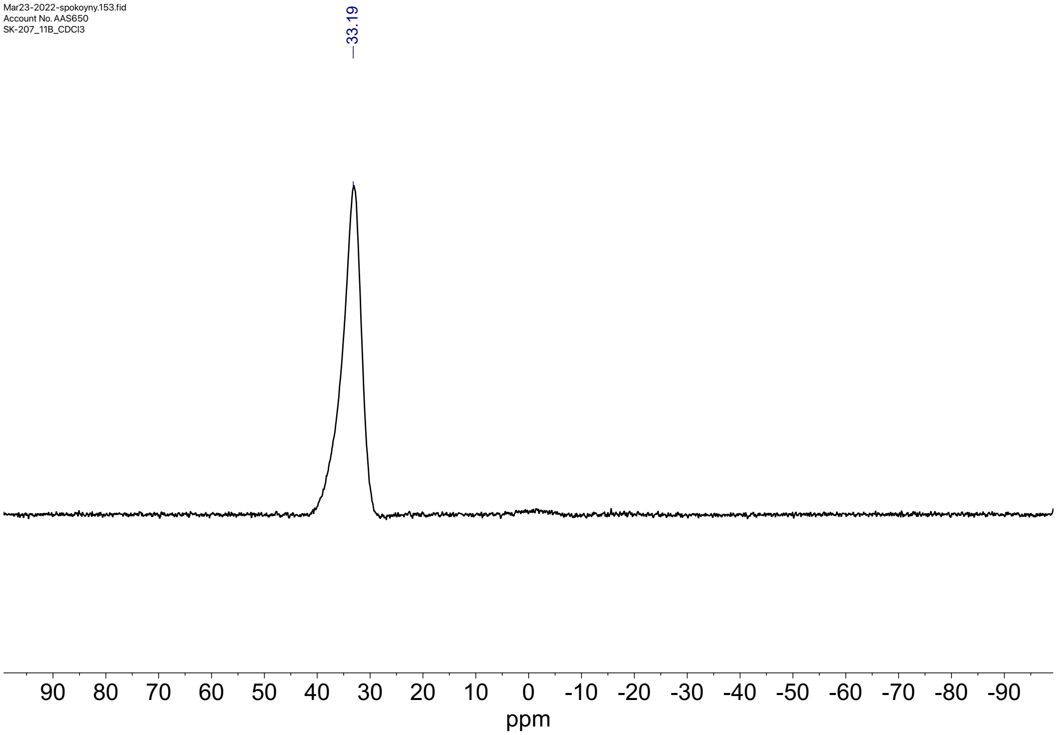

Supplementary Figure 130. ^{11}B NMR spectrum of **45** in $CDCl_3$.

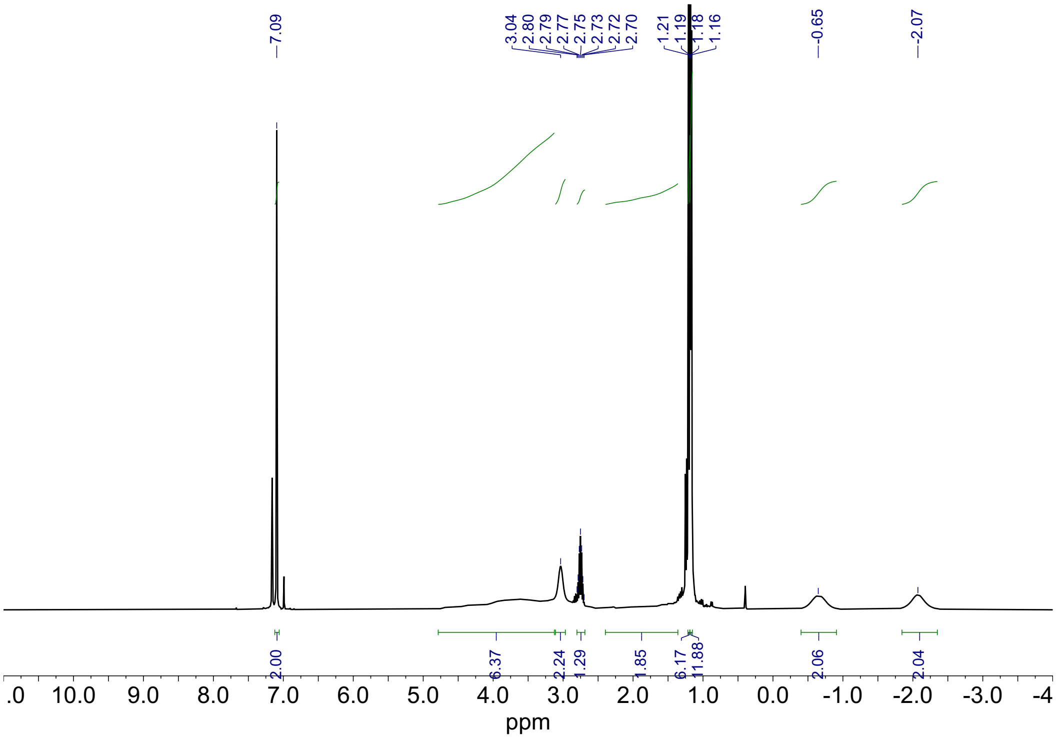

Supplementary Figure 131. ^{1}H NMR spectrum of **47** in C_6D_6.

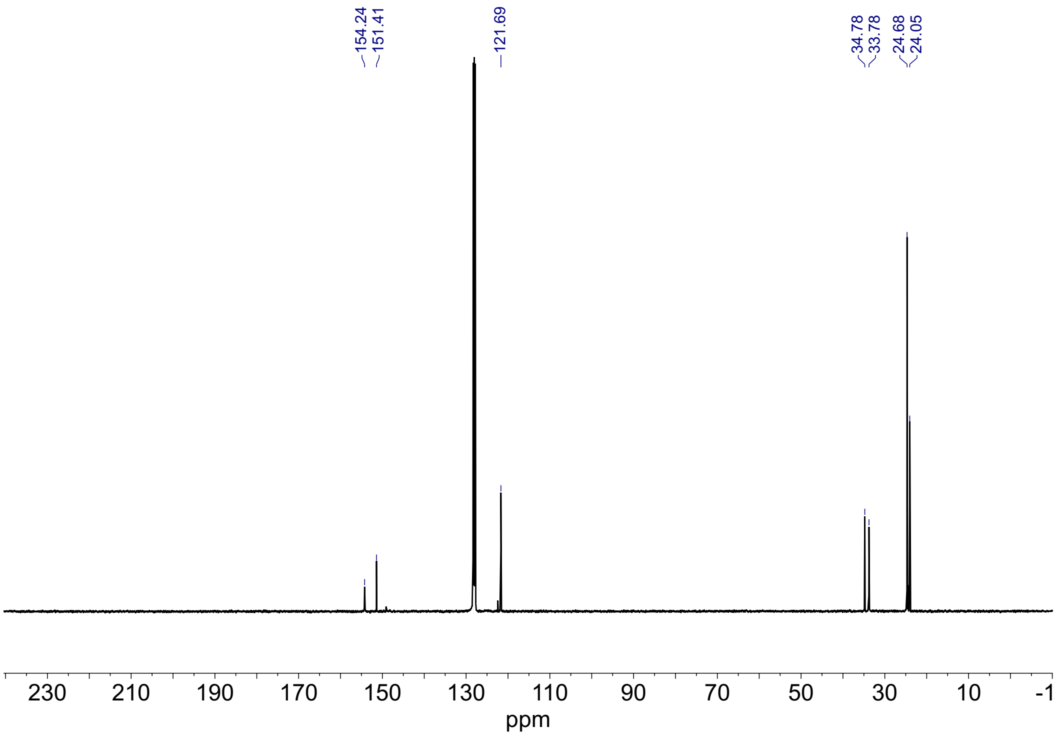

Supplementary Figure 132. ^{13}C NMR spectrum of **47** in C_6D_6.

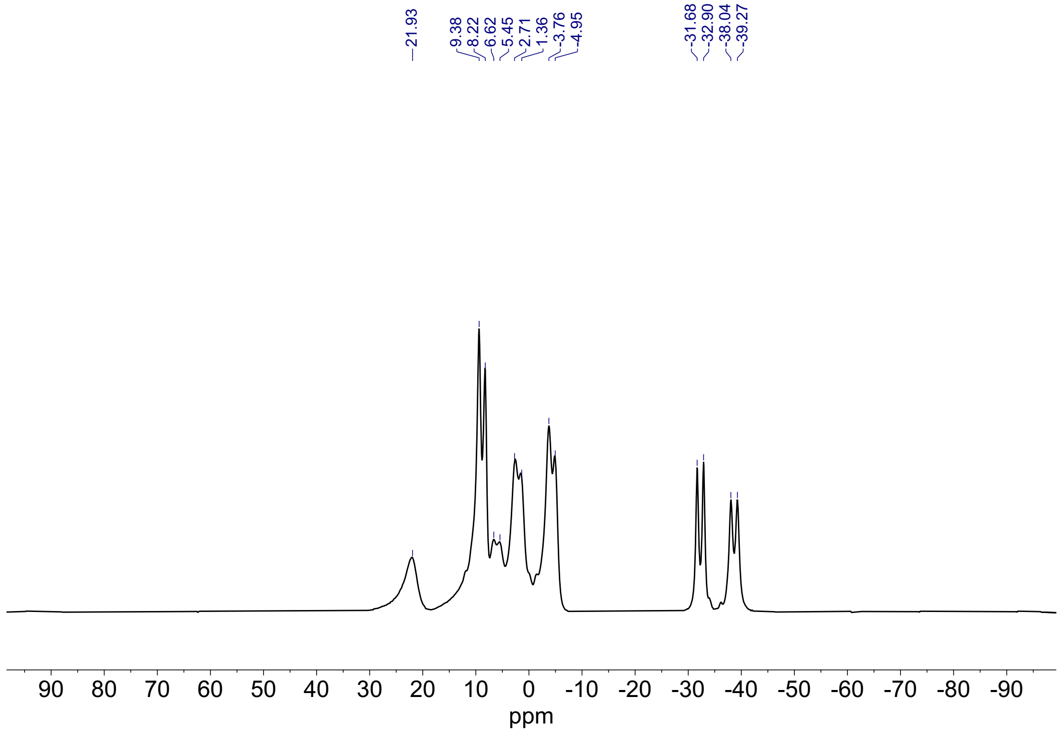

Supplementary Figure 133. ^{11}B NMR spectrum of **47** in C_6D_6.

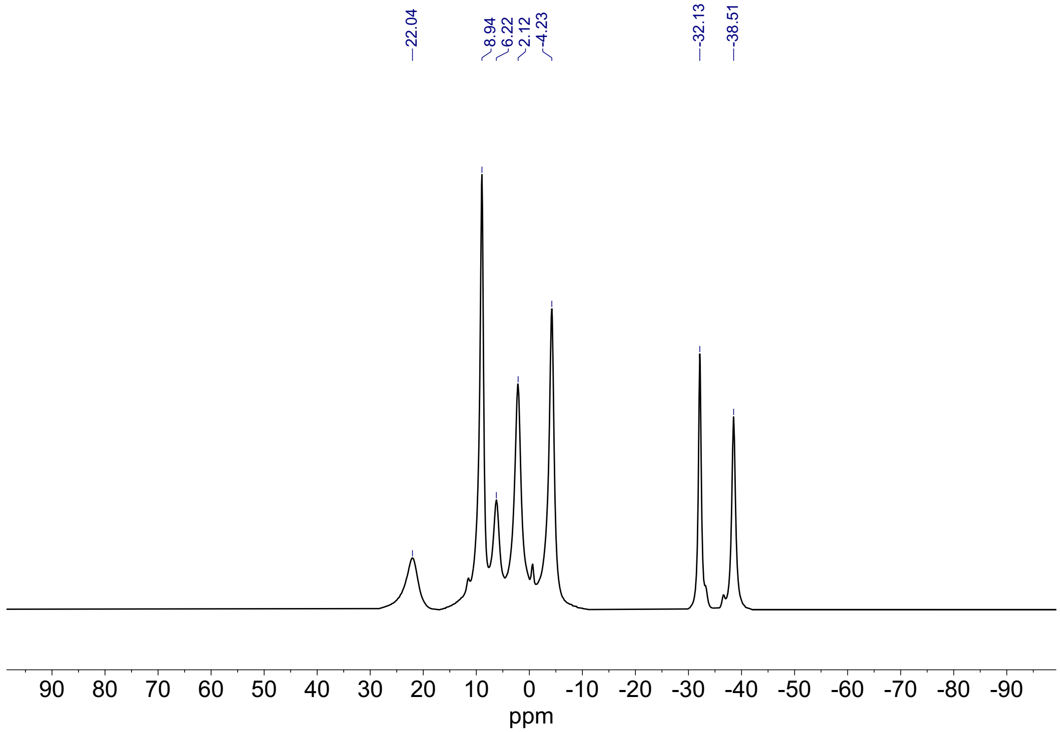

Supplementary Figure 134. $^{11}B\{^1H\}$ NMR spectrum of **47** in C_6D_6.

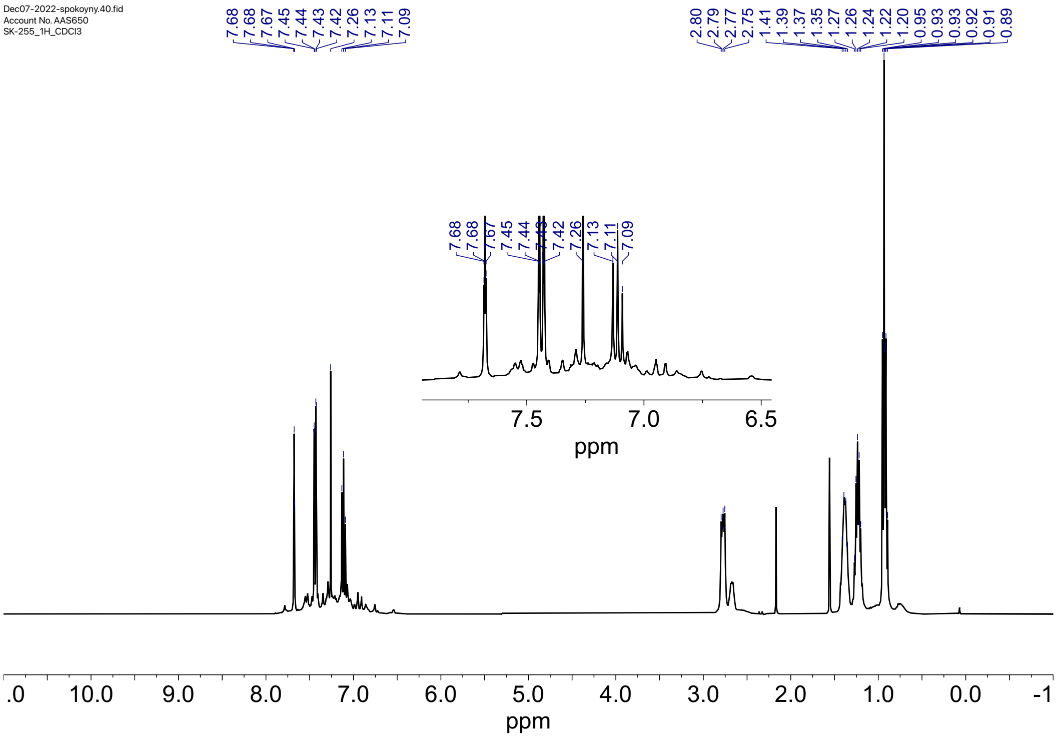

Supplementary Figure 135. ^{1}H NMR spectrum of $[TBA]_2[B_{10}(3,5\text{-}Br_2Ph)_nH_{10\text{-}n}]$ in $CDCl_3$. The sharp peaks at the aromatic region correspond to the remaining **42**.

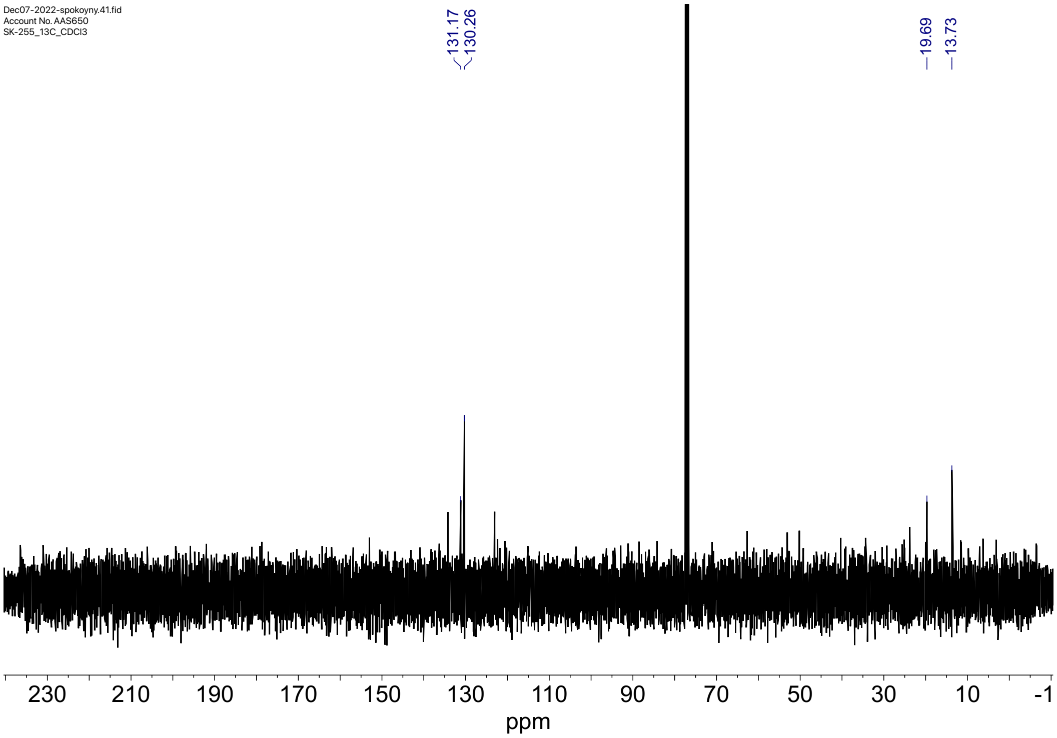

Supplementary Figure 136. ^{13}C NMR spectrum of $[TBA]_2[B_{10}(3,5\text{-}Br_2Ph)_nH_{10\text{-}n}]$ in $CDCl_3$.

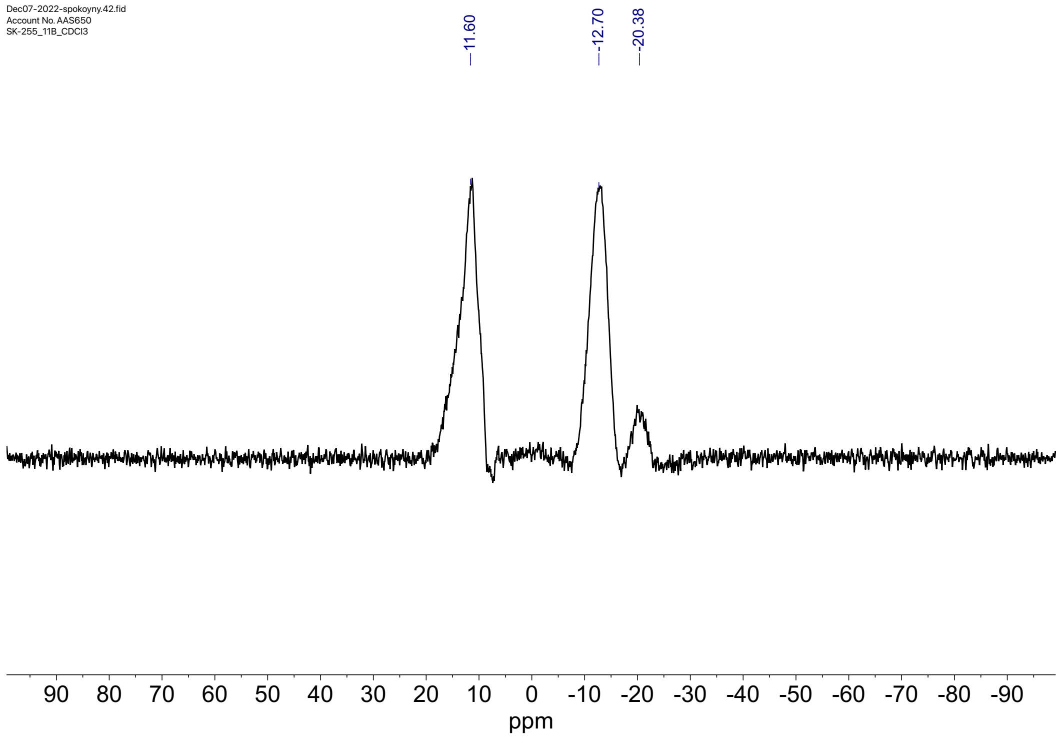

Supplementary Figure 137. ^{11}B NMR spectrum of $[TBA]_2[B_{10}(3,5\text{-}Br_2Ph)_nH_{10\text{-}n}]$ in $CDCl_3$.

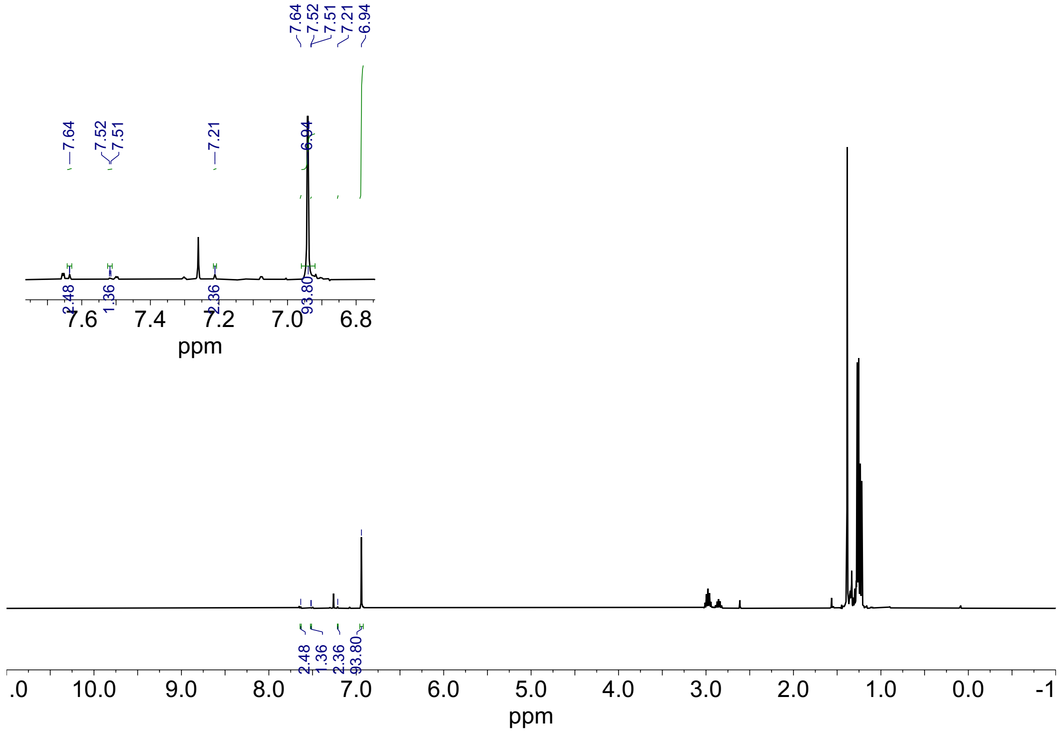

Supplementary Figure 138. Representative ^{1}H NMR spectrum of borylation of **44** conducted at 0 ºC (Fig. 5a) recorded in $CDCl_3$.

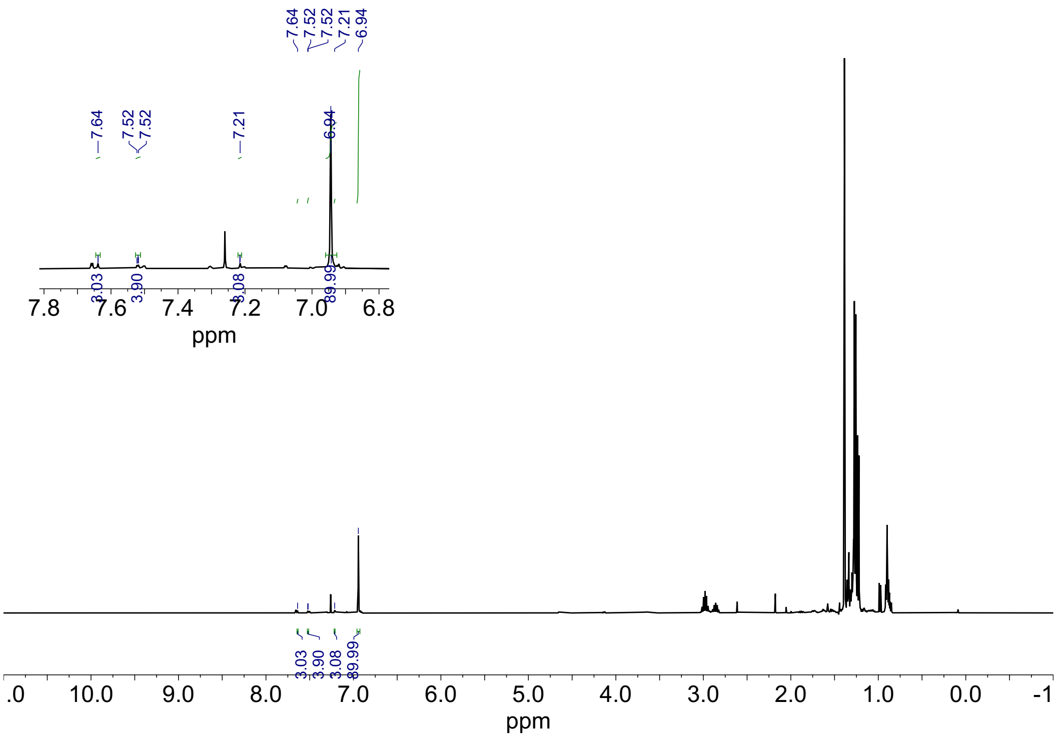

Supplementary Figure 139. Representative ^{1}H NMR spectrum of borylation of **44** conducted at 23 ºC (Fig. 5a) recorded in $CDCl_3$.

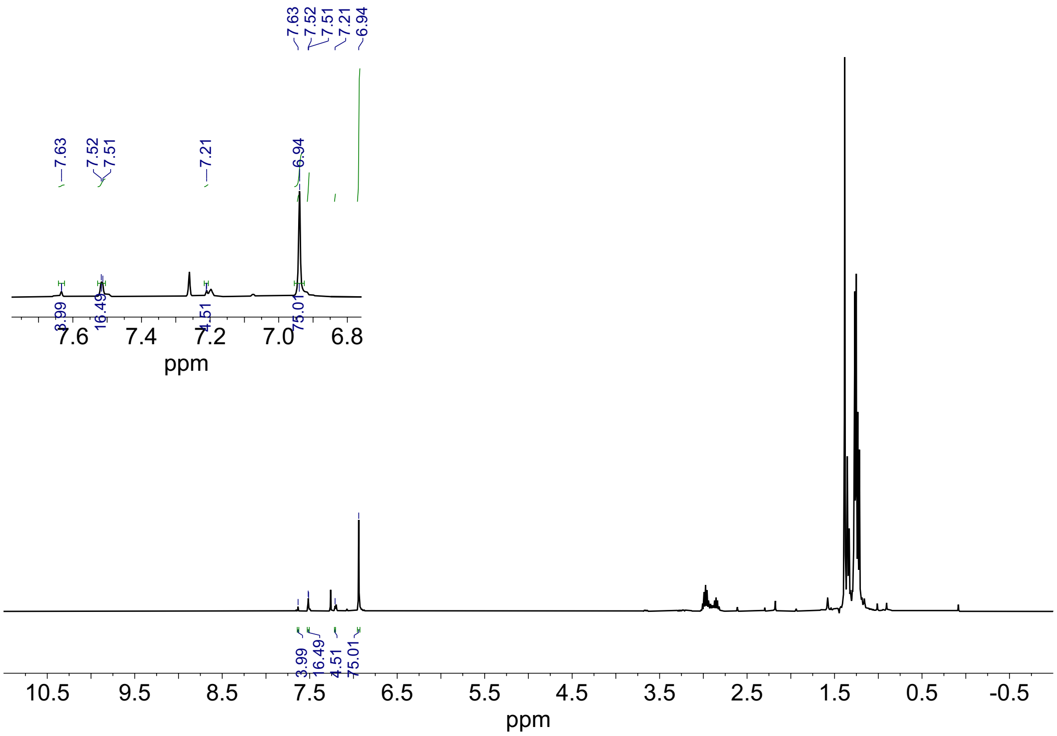

Supplementary Figure 140. Representative ^{1}H NMR spectrum of borylation of **44** conducted at 60 ºC (Figure 5a) recorded in $CDCl_3$.

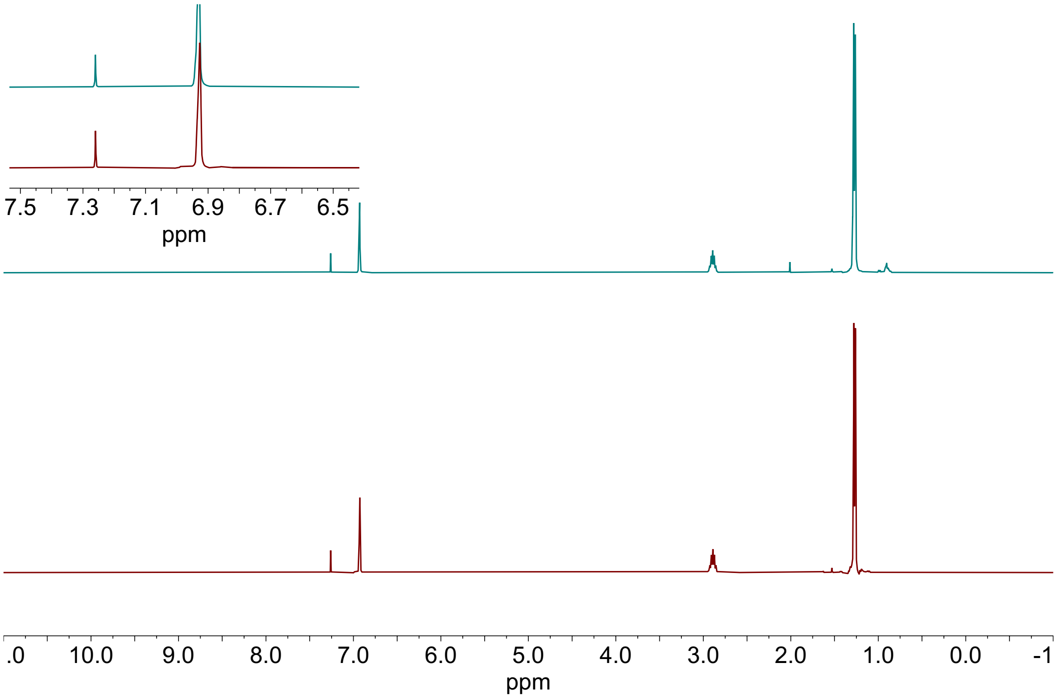

Supplementary Figure 141. Representative ^{1}H NMR spectra of the starting material **44** (bottom) and the crude reaction mixture of **44**and HOTf (top) in $CDCl_3$ (Figure 5b).

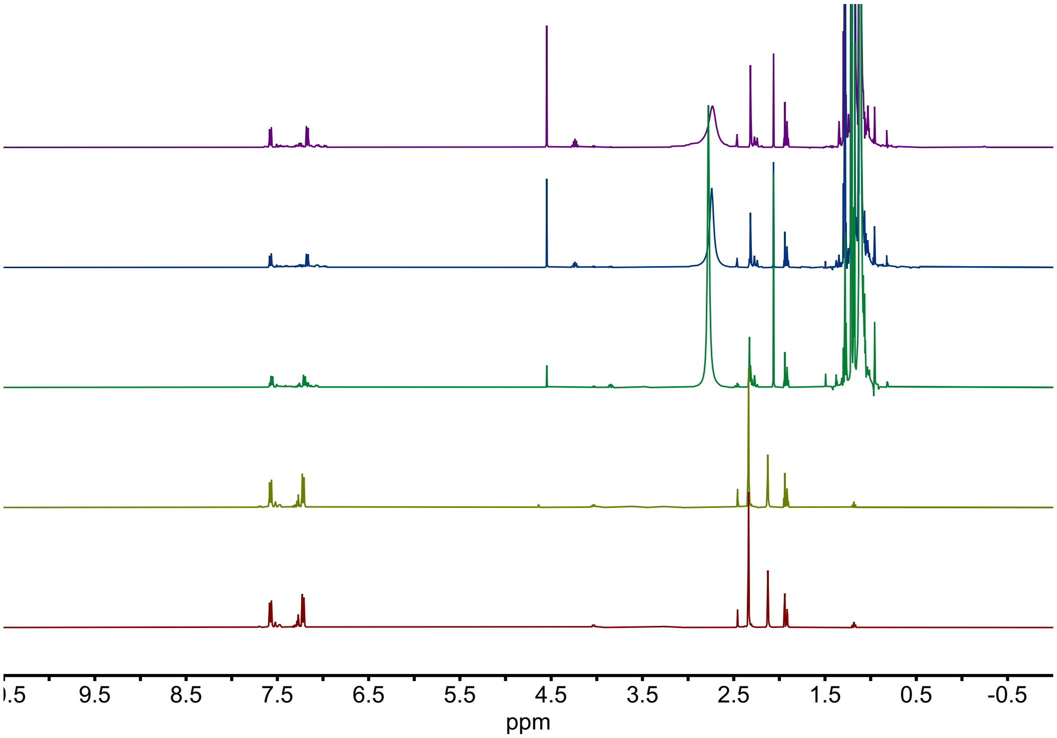

Supplementary Figure 142. Stacked ^{1}H NMR spectra in full range in Figure 2b.

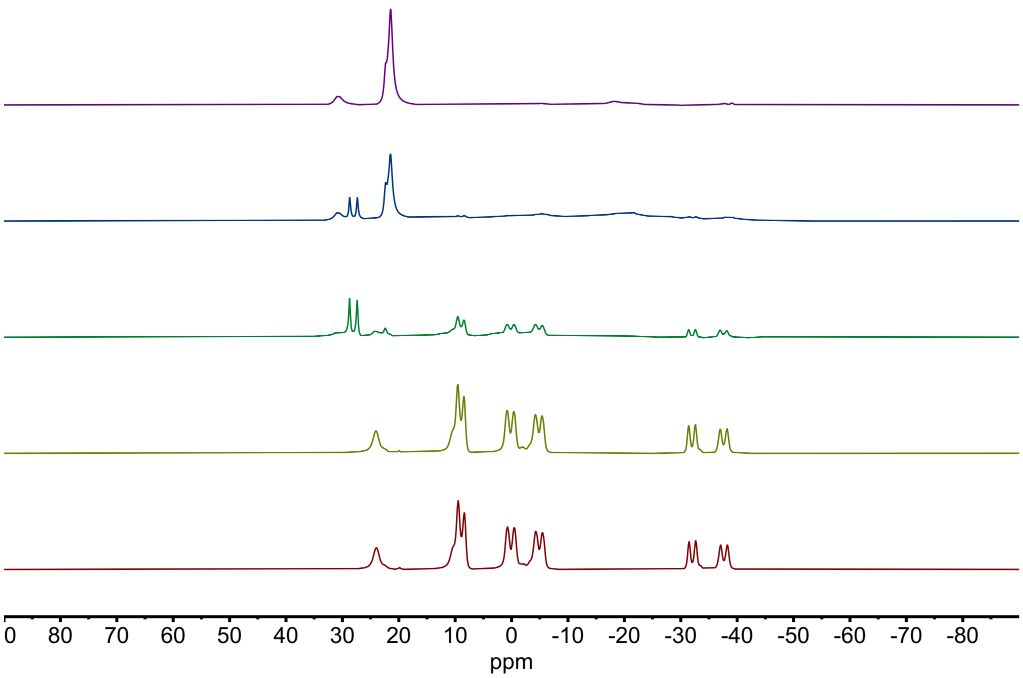

Supplementary Figure 143. Stacked ^{11}B NMR spectra in full range in Figure 2c.

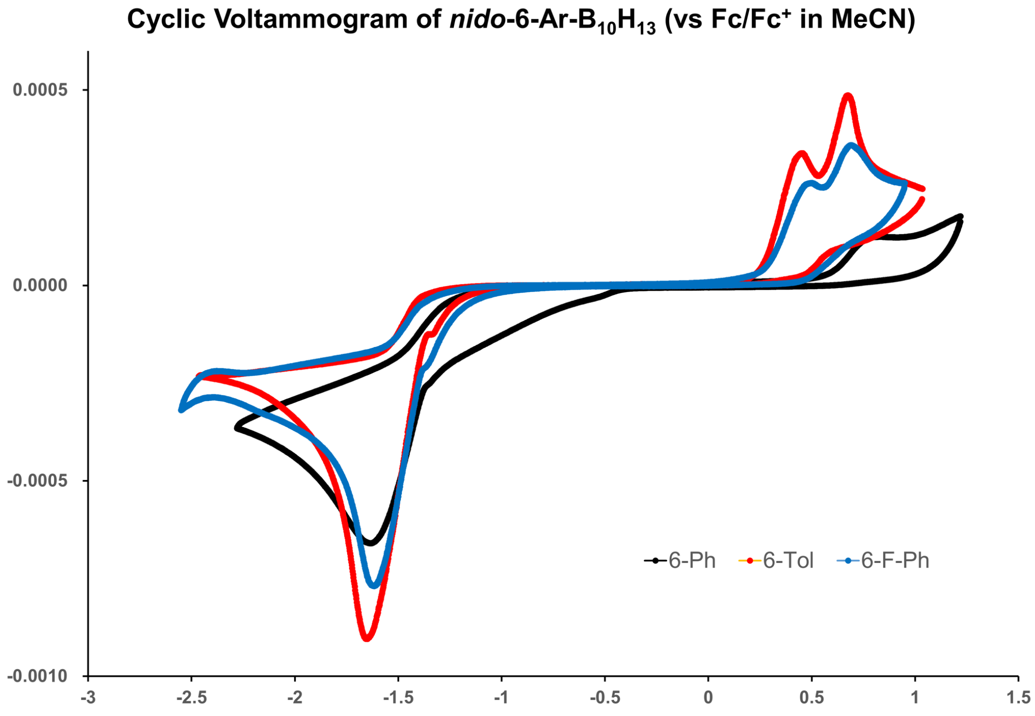

Supplementary Figure 144. Cyclic voltammograms of 0.025 M solutions of 6-Ar-*nido*-$B_{10}H_{13}$ (Ar = Ph, Tol, F-Ph) using a glassy-carbon working electrode, a platinum wire counter electrode, a silver wire reference electrode, 0.1 M [*n*-Bu_4N][PF_6] and a scan rate of 1000 mV/s in MeCN at 295 K versus Cp_2Fe/Cp_2Fe^+.

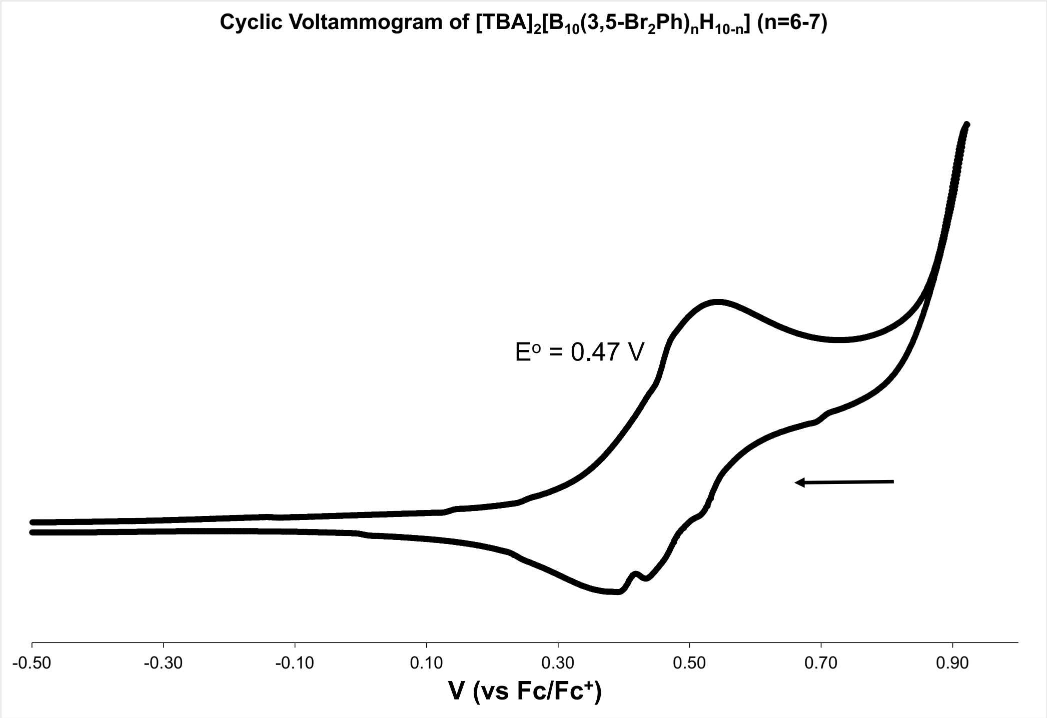

Supplementary Figure 145. Cyclic voltammograms of approximately 0.025 M solutions of $[TBA]_2[B10(3,5\text{-}Br_2Ph)_nH_{10\text{-}n}]$ (n = 6-7) using a glassy-carbon working electrode, a platinum wire counter electrode, a silver wire reference electrode, 0.1 M [*n*-Bu_4N][PF_6] and a scan rate of 100 mV/s in MeCN at 295 K versus Cp_2Fe/Cp_2Fe^+.

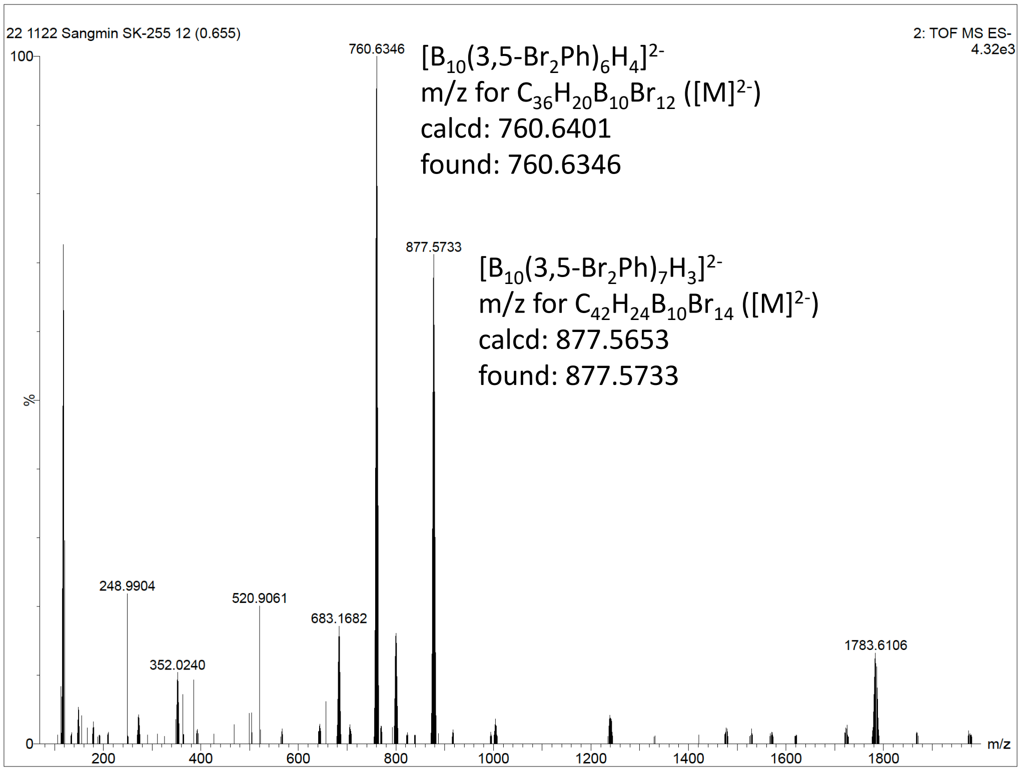

Supplementary Figure 146. ESI-MS(-) of $[TBA]_2[B10(3,5\text{-}Br_2Ph)_nH_{10\text{-}n}]$ (n = 6-7).

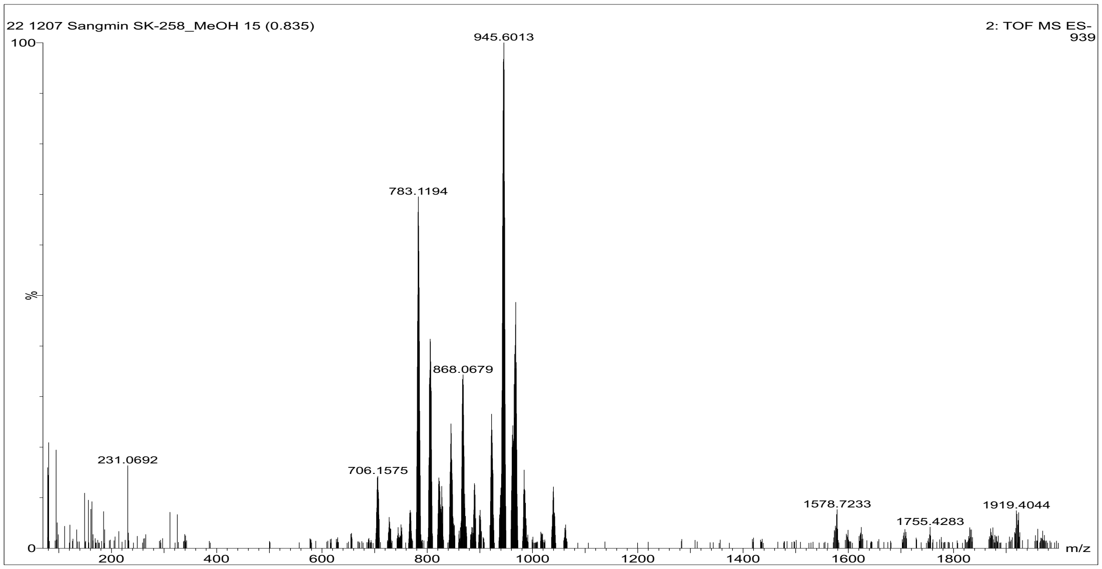

Supplementary Figure 147. ESI-MS(-) of the remaining residue after flash column chromatography of the deconstruction of $[TBA]_2[B10(3,5\text{-}Br_2Ph)_nH_{10-n}]$ (n = 6-7).

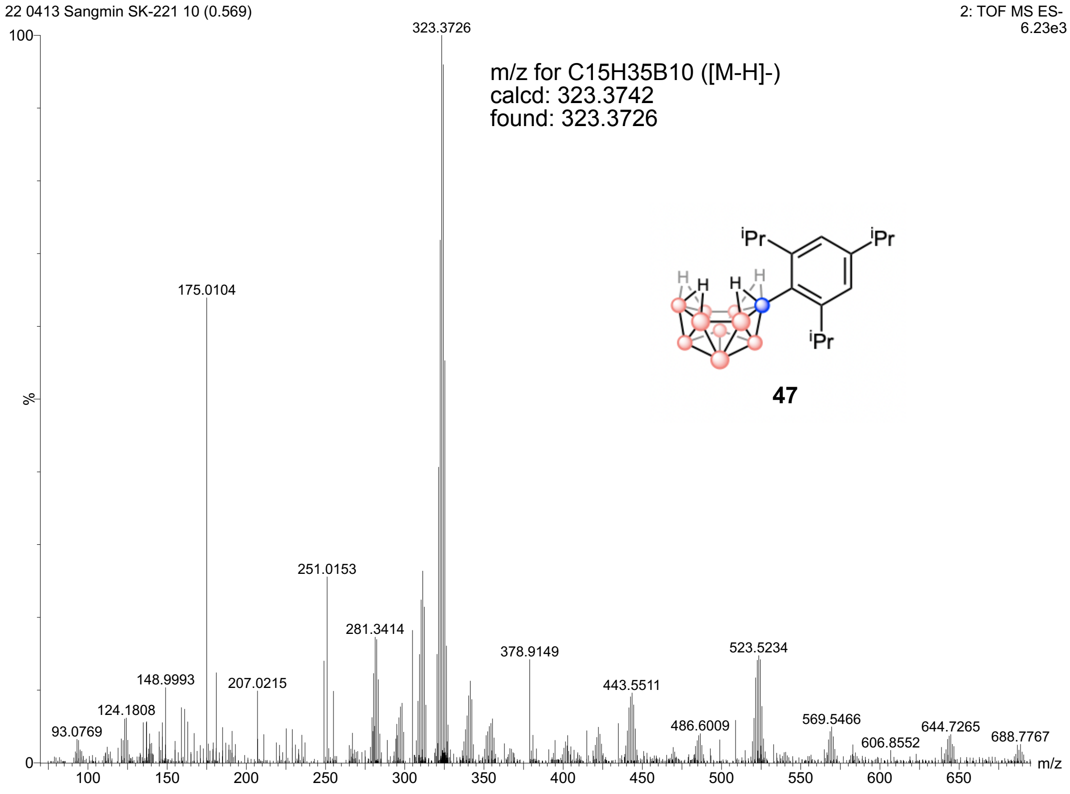

Supplementary Figure 148. ESI-MS(-) of **47**. *m/z* calculated for $C_{15}H_{35}B_{10}$ ($[M\text{-}H]^-$) 323.3742, found *m/z* 323.3726.

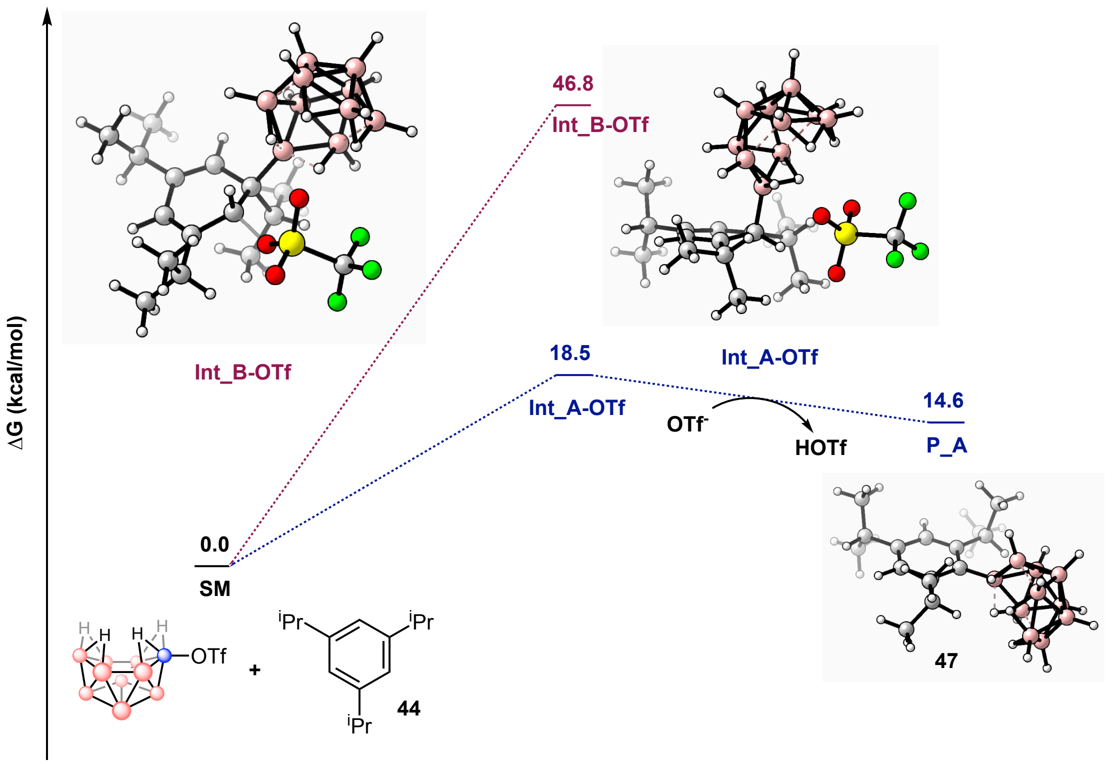

Supplementary Figure 149. DFT-calculated reaction profile for the formation of **47** from $B_{10}H_{13}(OTf)$ and **44**. DFT calculations were conducted at the ωB97X-D/6-311+G(d,p), CPCM(*n*-hexane) level of theory.

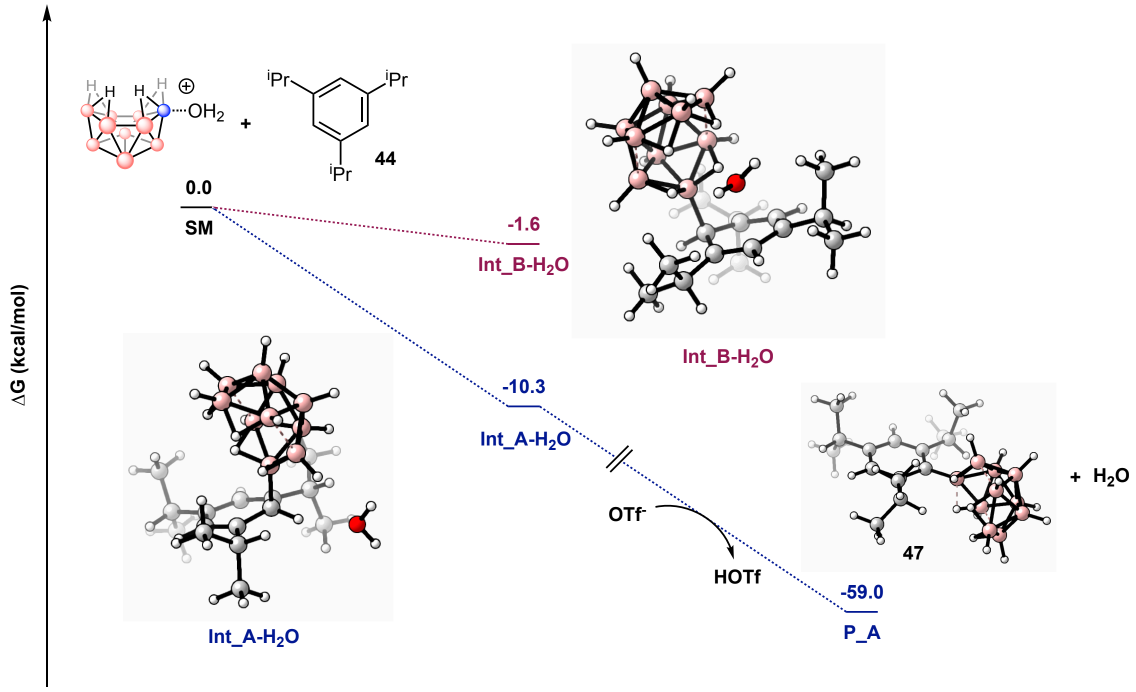

Supplementary Figure 150. DFT-calculated reaction profile for the formation of **47** from $B_{10}H_{13}(OH_2)^+$ and **44**. DFT calculations were conducted at the ωB97X-D/6-311+G(d,p), CPCM(*n*-hexane) level of theory.

IV. Supplementary References

1. Pangborn, A. B., Giardello, M. A., Grubbs, R. H., Rosen, R. K. & Timmers, F. J. Safe and Convenient Procedure for Solvent Purification. *Organometallics* **15**, 1518–1520 (1996).

2. Bondarev, O., Sevryugina, Y. V., Jalisatgi, S. S. & Hawthorne, M. F. Acid-Induced Opening of [*closo*-$B_{10}H_{10}$]$^{2-}$ as a New Route to 6-Substituted *nido*-$B_{10}H_{13}$ Decaboranes and Related Carboranes. *Inorg. Chem.* **51**, 9935-9942 (2012).

3. Fulmer, G. R., Miller, A. J. M., Sherden, N. H., Gottlieb, H. E., Nudelman, A., Stoltz, B. M., Bercaw, J. E. & Goldberg, K. I. NMR Chemical Shifts of Trace Impurities: Common Laboratory Solvents, Organics, and Gases in Deuterated Solvents Relevant to the Organometallic Chemist. *Organometallics* **29**, 2176-2179 (2010).

4. Gaussian 16, Revision A.03, M. J. Frisch, G. W. Trucks, H. B. Schlegel, G. E. Scuseria, M. A. Robb, J. R. Cheeseman, G. Scalmani, V. Barone, G. A. Petersson, H. Nakatsuji, X. Li, M. Caricato, A. V. Marenich, J. Bloino, B. G. Janesko, R. Gomperts, B. Mennucci, H. P. Hratchian, J. V. Ortiz, A. F. Izmaylov, J. L. Sonnenberg, D. Williams-Young, F. Ding, F. Lipparini, F. Egidi, J. Goings, B. Peng, A. Petrone, T. Henderson, D. Ranasinghe, V. G. Zakrzewski, J. Gao, N. Rega, G. Zheng, W. Liang, M. Hada, M. Ehara, K. Toyota, R. Fukuda, J. Hasegawa, M. Ishida, T. Nakajima, Y. Honda, O. Kitao, H. Nakai, T. Vreven, K. Throssell, J. A. Montgomery, Jr., J. E. Peralta, F. Ogliaro, M. J. Bearpark, J. J. Heyd, E. N. Brothers, K. N. Kudin, V. N. Staroverov, T. A. Keith, R. Kobayashi, J. Normand, K. Raghavachari, A. P. Rendell, J. C. Burant, S. S. Iyengar, J. Tomasi, M. Cossi, J. M. Millam, M. Klene, C. Adamo, R. Cammi, J. W. Ochterski, R. L. Martin, K. Morokuma, O. Farkas, J. B. Foresman, and D. J. Fox, Gaussian, Inc., Wallingford CT, 2016.

5. Barone, V. & Cossi, M. Quantum Calculation of Molecular Energies and Energy Gradients in Solution by a Conductor Solvent Model. *J. Phys. Chem. A* **102**, 1995–2001 (1998).

6. Chai, J.-D. & Head-Gordon, M. Long-range corrected hybrid density functionals with damped atom–atom dispersion corrections. *Phys. Chem. Chem. Phys.* **10**, 6615 (2008).

7. Grimme, S. Supramolecular Binding Thermodynamics by Dispersion-Corrected Density Functional Theory. *Chem. - Eur. J.* **18**, 9955–9964 (2012).

8. Luchini, G., Alegre-Requena, J. V., Funes-Ardoiz, I. & Paton, R. S. GoodVibes: automated thermochemistry for heterogeneous computational chemistry data. *F1000Research* **9**, 291 (2020).

9. Grimme, S., Bannwarth, C., Dohm, S., Hansen, A., Pisarek, J., Pracht, P., Seibert, J. & Neese, F. Fully Automated Quantum-Chemistry-Based Computation of Spin-Spin-Coupled Nuclear Magnetic Resonance Spectra. *Angew. Chem. Int. Ed.* **56**, 14763–14769 (2017).

10. Grimme, S. Exploration of Chemical Compound, Conformer, and Reaction Space with Meta-Dynamics Simulations Based on Tight-Binding Quantum Chemical Calculations. *J. Chem. Theory Comput.* **15**, 2847–2862 (2019).

11. Grimme, S., Bannwarth, C. & Shushkov, P. A Robust and Accurate Tight-Binding Quantum Chemical Method for Structures, Vibrational Frequencies, and Noncovalent

Interactions of Large Molecular Systems Parametrized for All spd-Block Elements (*Z* = 1–86). *J. Chem. Theory Comput.* **13**, 1989–2009 (2017).

12. Bannwarth, C., Ehlert, S. & Grimme, S. GFN2-xTB—An Accurate and Broadly Parametrized Self-Consistent Tight-Binding Quantum Chemical Method with Multipole Electrostatics and Density-Dependent Dispersion Contributions. *J. Chem. Theory Comput.* **15**, 1652–1671 (2019).

13. Pracht, P., Caldeweyher, E., Ehlert, S. & Grimme, S. *A Robust Non-Self-Consistent Tight-Binding Quantum Chemistry Method for large Molecules.* https://chemrxiv.org/articles/A_Robust_Non-Self-Consistent_Tight-Binding_Quantum_Chemistry_Method_for_large_Molecules/8326202/1 (2019) doi:10.26434/chemrxiv.8326202.v1.

14. CYLview20; Legault, C. Y., Université de Sherbrooke, 2020 (http://www.cylview.org).

15. The PyMOL Molecular Graphics System, Version 2.4.1, Schrödinger, LLC.

16. Kato, T., Kuriyama, S., Nakajima, K. & Nishibayashi, Y. Catalytic C−H Borylation Using Iron Complexes Bearing 4,5,6,7-Tetrahydroisoindol-2-ide-Based PNP-Type Pincer Ligand. *Chem. Asian J.* **14**, 2097-2101 (2019).

17. (a) (*para*- and *meta*-isomers) Cho, J.-Y., Iverson, C. N. & Smith, M. R. Steric and Chelate Directing Effects in Aromatic Borylation. *J. Am. Chem. Soc.* **122**, 12868-12869 (2000). (b) (*ortho*-isomer) Kalvet, I., Sperger, T., Scattolin, T., Magnin, G. & Schoenebeck, F. Palladium(I) Dimer Enabled Extremely Rapid and Chemoselective Alkylation of Aryl Bromides over Triflates and Chlorides in Air. *Angew. Chem. Int. Ed.* **56**, 7078-7082 (2017).

18. Mazzarella, D., Pulcinella, A., Bovy, L., Broersma, R. & Noël, T. Rapid and Direct Photocatalytic C(sp^3)−H Acylation and Arylation in Flow. *Angew. Chem. Int. Ed.* **60**, 21277-21282 (2021).

19. Jin, S., Dang, H. T., Haug, G. C., He, R., Nguyen, V. D., Nguyen, V. T., Arman, H. D., Schanze, K. S. & Larionov, O. V. Visible Light-Induced Borylation of C−O, C−N, and C−X Bonds. *J. Am. Chem. Soc.* **142**, 1603-1613 (2020).

20. Niwa, T., Ochiai, H., Watanabe, Y. & Hosoya, T. Ni/Cu-Catalyzed Defluoroborylation of Fluoroarenes for Diverse C–F Bond Functionalizations. *J. Am. Chem. Soc.* **137**, 14313-14318 (2015).

21. Saito, Y., Segawa, Y. & Itami, K. *para*-C–H Borylation of Benzene Derivatives by a Bulky Iridium Catalyst. *J. Am. Chem. Soc.* **137**, 5193-5198 (2015).

22. Uetake, Y., Niwa, T. & Hosoya, T. Rhodium-Catalyzed ipso-Borylation of Alkylthioarenes via C–S Bond Cleavage. *Org. Lett.* **18**, 2758-2761 (2016).

23. Pein, W. L., Wiensch, E. M. & Montgomery, J. Nickel-Catalyzed Ipso-Borylation of Silyloxyarenes via C–O Bond Activation. *Org. Lett.* **23**, 4588-4592 (2021).

24. Furukawa, T., Tobisu, M. & Chatani, N. C−H Functionalization at Sterically Congested Positions by the Platinum-Catalyzed Borylation of Arenes. *J. Am. Chem. Soc.* **137**, 12211-12214 (2015).

25. Preshlock, S. M., Ghaffari, B., Maligres, P. E., Krska, S. W., Maleczka, Jr., R. E. & Smith, III, M. R. High-Throughput Optimization of Ir-Catalyzed C–H Borylation: A Tutorial for Practical Applications. *J. Am. Chem. Soc.* **135**, 7572-7582 (2013).

26. Walsh, T. F., Toupence, R. B., Ujjainwalla, F., Young, J. R. & Goulet, M. T. A convergent synthesis of (*S*)-β-methyl-2-aryltryptamine based gonadotropin releasing hormone antagonists. *Tetrahedron* **57**, 5233-5241 (2001).

27. Kuwano, R., Lee, E. & Won, S. Economical and Readily Accessible Preparation of *o,o*-Disubstituted Arylboronates through Palladium-Catalyzed Borylation of Haloarenes. *Org. Lett.* **23**, 9649-9653 (2021).

28. Del Grosso, A., Carrillo, J. A. & Ingleson, M. J. Regioselective electrophilic borylation of haloarenes. *Chem. Commun.* **51**, 2878-2881 (2015).

29. (a) Wager, T. T., Welch, Jr., W. M. & O'neill, B. T. Nk1 antagonist. (2004). WO2004110996 (A1). (b) Sime, M., Bower, J., Mcarthur, D. & Pugliese, A. Isoquinoline derivatives as SIK2 inhibitors. (2021). WO2021084264 (A1).

30. Takaya, J., Ito, S., Nomoto, H., Saito, N., Kirai, N. & Iwasawa, N. Fluorine-controlled C–H borylation of arenes catalyzed by a PSiN-pincer platinum complex. *Chem. Commun.* **51**, 17662-17665 (2015).

31. Chen, S., Pan, Z. & Wang, Y. PPh_3-Mediated Borylation of Arenediazonium Salts with Bis(pinacolato)diborane. *Z. Naturforsch.* **69b**, 982-986 (2014).

32. Dastbaravardeh, N., Schnürch, M. & Mihovilovic, M. D. Ruthenium(0)-Catalyzed sp^3 C–H Bond Arylation of Benzylic Amines Using Arylboronates. *Org. Lett.* **14**, 1930-1933 (2012).

33. Miralles, N., Romero, R. M., Fernández, E. & Muñiz, K. A mild carbon–boron bond formation from diaryliodonium salts. *Chem. Commun.* **51**, 14068-14071 (2015).

34. Mfuh, A. M., Doyle, J. D., Chhetri, B., Arman, H. D. & Larionov, O. V. Scalable, Metal- and Additive-Free, Photoinduced Borylation of Haloarenes and Quaternary Arylammonium Salts. *J. Am. Chem. Soc.* **138**, 2985-2988 (2016).

35. Wood, J. L., Marciasini, L. D., Vaultier, M. & Pucheault, M. Iron Catalysis and Water: A Synergy for Refunctionalization of Boron. *Synlett* **25**, 551-555 (2014).

36. Wang, G., Xu, L., Li, P. Double N,B-Type Bidentate Boryl Ligands Enabling a Highly Active iridium Catalyst for C–H Borylation. *J. Am. Chem. Soc.* **137**, 8058-8061 (2015).
