# Supplementary material for: Arene C–H borylation strategy enabled by a non-classical boron cluster-based electrophile
Source: Nat Commun. 2023 Mar 25;14:1671. doi: 10.1038/s41467-023-37258-6 (PMC10039867; doi:10.1038/s41467-023-37258-6)
Supplement: Supplementary file 3 — Description of Additional Supplementary Files [file 41467_2023_37258_MOESM3_ESM.pdf]

Description of Additional Supplementary Files

Supplementary Data 1: Cartesian Coordinates of the Structures

Supplementary Data 2: Crystallographic Data
