# Supplementary material for: Arene C–H borylation strategy enabled by a non-classical boron cluster-based electrophile
Source: Nat Commun. 2023 Mar 25;14:1671. doi: 10.1038/s41467-023-37258-6 (PMC10039867; doi:10.1038/s41467-023-37258-6)
Supplement: Supplementary file 4 — Supplementary Data 1 [file 41467_2023_37258_MOESM4_ESM.pdf]

Supplementary Data File 1: Cartesian Coordinates of the Structures

Computed Energies

Supplementary Table 3. Computed energies of the structures calculated at the ωB97X-D/6-311+G(d,p), CPCM(*n*-Hexane) level of theory. All units are in Hartrees.

Structure	E	ZPE	H	T.S	T. qh-S	G(T)	qh-G(T)
$B_{10}H_{13}^{+}$	-256.05275	0.165697	-255.87726	0.041167	0.041177	-255.91842	-255.91843
$B_{10}H_{13}$-OTf	-1217.86276	0.201833	-1217.643963	0.061673	0.058514	-1217.705636	-1217.702477
$B_{10}H_{13}^{+}$-H_2O	-332.594325	0.198424	-332.38493	0.044343	0.044209	-332.429273	-332.429139
**44**	-586.04974	0.35465	-585.67682	0.063739	0.060914	-585.74056	-585.73774
Int_A	-842.23428	0.529844	-841.67744	0.081042	0.077011	-841.75849	-841.75446
Int_A-OTf	-1803.911447	0.559673	-1803.316234	0.10163	0.094574	-1803.417864	-1803.410808
Int_A-H2O	-918.68226	0.553542	-918.097578	0.091212	0.085774	-918.18879	-918.183352
Int_B	-842.21617	0.528979	-841.66017	0.081036	0.076784	-841.74121	-841.73695
Int_B-OTf	-1803.870268	0.561434	-1803.27455	0.096539	0.09112	-1803.371089	-1803.36567
Int_B-H_2O	-918.670889	0.554498	-918.086073	0.087483	0.08332	-918.173555	-918.169393
**47**	-841.85439	0.517969	-841.30982	0.080104	0.076299	-841.38993	-841.38612
Triflate	-961.58421	0.027513	-961.54869	0.040313	0.039883	-961.589	-961.58857
Triflic Acid	-962.03755	0.039161	-961.98975	0.041574	0.041006	-962.03133	-962.03076
Water	-76.435929	0.021646	-76.410502	0.022066	0.022066	-76.432568	-76.432568

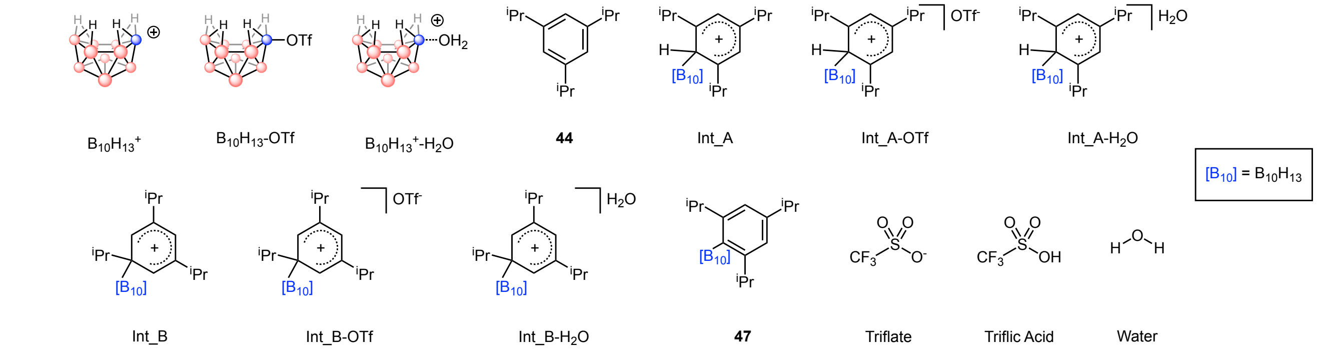

Coordinates of Computed Structures

$B_{10}H_{13}^{+}$

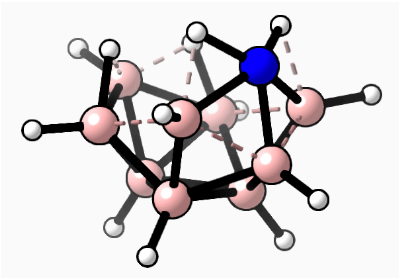

Charge: 1 Multiplicity: 1
Negative Frequency: None

B	-0.19195200	0.89307800	1.05636800
B	-1.64329100	-0.00071900	0.47057400
B	-0.19139900	-0.89322500	1.05654200
B	1.32142800	0.00050900	0.85742200
B	-1.02708200	1.49440900	-0.36213900
B	-1.38682900	-0.00049800	-1.15708300
B	-1.02543700	-1.49487200	-0.36269700
B	0.96903300	-1.42816900	-0.15865700
B	1.83932600	0.00065800	-0.79405800

B	0.96808000	1.42874400	-0.15880500
H	1.13966900	0.99249800	-1.39001600
H	-2.70236200	-0.00104600	0.99956000
H	2.91292600	0.00124700	-1.28332200
H	-0.34569300	-1.65508600	1.94965100
H	1.49949400	2.48237200	-0.11854700
H	1.50131400	-2.48134600	-0.11797100
H	-0.34711000	1.65530800	1.94906700
H	-1.60091800	2.50877700	-0.53143000
H	2.09973500	0.00085400	1.74660400
H	-0.93029200	1.00505300	-1.76054600
H	1.14101100	-0.99212700	-1.38987100
H	-1.59891600	-2.50959900	-0.53123900
H	-0.92824100	-1.00648000	-1.75927400

$B_{10}H_{13}$-OTf

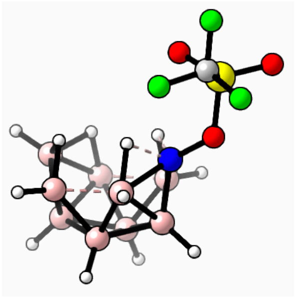

Charge: 0 Multiplicity: 1
Negative Frequency: None

B	3.44025300	-0.74992000	-0.82734800
B	1.94155300	-0.22286300	-1.62297900
B	3.10368300	0.98352400	-1.03127300
B	4.10797300	0.42621600	0.32404000
B	1.93990300	-1.54164300	-0.40503100
B	0.62844500	-0.35144400	-0.53224600
B	1.40073700	1.23821500	-0.73134000
B	2.83860000	1.66138000	0.56169700
B	3.20062900	0.42344900	1.79436200
B	3.37275000	-1.10279800	0.88675800
H	2.49129500	-0.70940200	1.79329600
H	1.75920400	-0.39262500	-2.78027600
H	3.54816700	0.62133200	2.90748200
H	3.57167200	1.73814900	-1.81747500
H	3.93618500	-2.01672000	1.38603000
H	3.00327300	2.80580900	0.81738800
H	4.18759900	-1.43382000	-1.44395600
H	1.69405200	-2.68096500	-0.60985700
H	5.27013200	0.65294700	0.33725800
H	1.05340800	-1.13275600	0.48295200
H	2.12651600	1.18630400	1.57178700
H	0.76034600	2.12907500	-1.17440400
H	0.69841100	0.73972100	0.26560600
S	-1.86037900	-0.86085400	0.21583900
O	-1.26525000	-0.85951800	1.52951000
O	-2.73846100	-1.89684900	-0.23084400

O	-0.72468100	-0.64759100	-0.87273300
C	-2.75307200	0.76983300	0.01146400
F	-3.77941800	0.79223400	0.84177000
F	-3.17403800	0.90001400	-1.23081600
F	-1.92001800	1.75872000	0.30585800

$B_{10}H_{13}$-H_2O

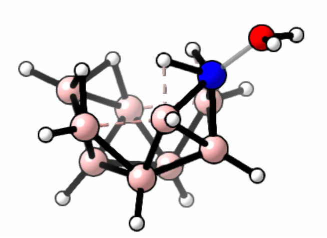

Charge: 1 Multiplicity: 1
Negative Frequency: None

B	-0.76435600	-0.89034800	1.12536800
B	0.77528200	-0.00008700	1.21486900
B	-0.76438300	0.89016300	1.12550800
B	-2.06524700	-0.00002700	0.31612700
B	0.58464600	-1.43217300	0.15118800
B	1.48646100	0.00004700	-0.32632800
B	0.58464000	1.43219200	0.15148500
B	-1.29829600	1.42113000	-0.46191800
B	-1.83976700	0.00010300	-1.40143500
B	-1.29830700	-1.42106500	-0.46213700
H	-0.96360700	-0.98063800	-1.66249000
H	1.46882200	-0.00018600	2.17508100
H	-2.62611500	0.00020700	-2.28277200
H	-1.02675900	1.63529000	2.00751100
H	-1.78894500	-2.47948100	-0.65295600
H	-1.78894100	2.47956600	-0.65260700
H	-1.02673400	-1.63558800	2.00727300
H	1.15839600	-2.46313700	0.24837300
H	-3.15402300	-0.00005700	0.77563200
H	0.95020500	-0.95048100	-1.06610500
H	-0.96351900	0.98084400	-1.66229400
H	1.15835800	2.46316300	0.24875700
H	0.95037000	0.95065600	-1.06595700
O	2.96643100	-0.00009300	-0.60977500
H	3.45896800	-0.79513100	-0.34997900
H	3.45872300	0.79604600	-0.35290500

44

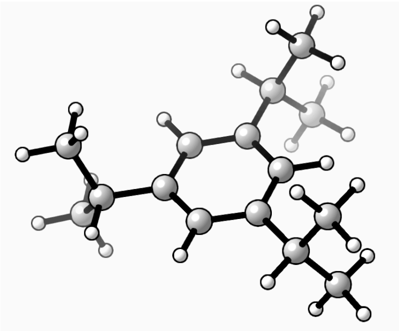

Charge: 0 Multiplicity: 1
Negative Frequency: None

C	-0.20053500	1.21413300	-0.00076800
C	1.13391000	0.81816800	-0.00099900
C	1.49475700	-0.52958300	-0.00066000
C	0.48255300	-1.48542200	-0.00007600
C	-0.86469600	-1.12421800	0.00017800
C	-1.18869200	0.22966700	-0.00016800
C	2.95313800	-0.94796700	-0.00063100
H	2.97339100	-2.04348400	-0.00138300
C	3.67764700	-0.46867500	-1.26370100
H	3.70826400	0.62440900	-1.30770500
H	4.70920600	-0.83263700	-1.27698600
H	3.17508300	-0.82724700	-2.16566100
C	3.67670000	-0.47036600	1.26365000
H	4.70837300	-0.83400600	1.27710900
H	3.70684700	0.62267100	1.30924000
H	3.17362500	-0.83042400	2.16473300
C	-1.94823200	-2.18651400	0.00067300
H	-1.44501600	-3.15981300	0.00134600
C	-2.81292700	-2.10938500	1.26413400
H	-3.36036100	-1.16270300	1.30787200
H	-3.54670800	-2.92062700	1.27838500
H	-2.19982700	-2.18459100	2.16585900
C	-2.81242200	-2.11084100	-1.26323100
H	-2.19896700	-2.18723500	-2.16461500
H	-3.54631800	-2.92199200	-1.27678800
H	-3.35966500	-1.16411100	-1.30832900
C	-0.56891900	2.68616200	-0.00075400
H	0.36890500	3.25275000	-0.00282400
C	-1.34310200	3.07326200	1.26445900
H	-0.77844900	2.81798300	2.16482600
H	-1.54498800	4.14833800	1.27823900
H	-2.30435600	2.55209400	1.31141000
C	-1.34817200	3.07256800	-1.26303000
H	-1.54952600	4.14774000	-1.27680000
H	-0.78740700	2.81630100	-2.16554200
H	-2.30994600	2.55199500	-1.30563100
H	0.74843000	-2.53962700	0.00016500
H	1.90720300	1.58187900	-0.00143000
H	-2.23384000	0.52640700	0.00004500

Int_A

Charge: 1 Multiplicity: 1
Negative Frequency: None

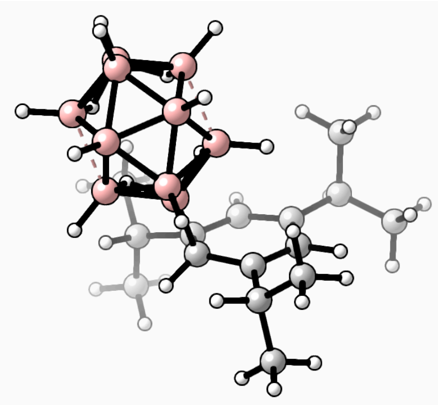

B	-3.77930600	-1.12652600	-0.34699500
B	-2.07382700	-1.62685500	-0.45949300
B	-2.93439100	-1.72982100	1.09577300
B	-4.26873300	-0.57339500	1.26877400
B	-2.65790500	-0.12702900	-1.23496500
B	-1.05311200	-0.24365900	-0.47510400
B	-1.30659700	-1.09966300	1.07023300
B	-2.75492400	-0.38451100	2.20464000
B	-3.66390300	1.01811400	1.57847000
B	-4.11226000	0.57602000	-0.09187400
H	-3.32824500	1.52787500	0.38909400
H	-1.77499000	-2.60829700	-1.04913600
H	-4.11698200	1.90168800	2.22017000
H	-3.02332600	-2.79195800	1.61407200
H	-4.98416600	1.15579100	-0.64292600
H	-2.62027600	-0.51592500	3.37316000
H	-4.57057500	-1.68756300	-1.02773200
H	-2.70289100	0.10435200	-2.39581500
H	-5.30041300	-0.92138100	1.73114400
H	-1.85912600	0.79364700	-0.72139900
H	-2.38955700	0.86215500	1.95805800
H	-0.38956100	-1.61458900	1.61157800
H	-0.91070800	0.13553000	0.80123000
C	2.29586500	0.49217400	0.72202000
C	2.14802200	-0.78850800	0.16687000
C	1.26073900	-1.01915500	-0.85452100
C	0.37484900	0.06549600	-1.27247600
C	0.69791100	1.42900300	-0.83703100
C	1.59115300	1.59178200	0.18500000
C	1.16477200	-2.33993800	-1.56558900
H	0.22341300	-2.34764200	-2.12191000
C	2.31170300	-2.40342000	-2.59326700
H	3.28255100	-2.41600800	-2.09120900
H	2.21732000	-3.31846400	-3.18135800
H	2.28884000	-1.55336600	-3.27955900
C	1.18122200	-3.54789200	-0.62765800
H	0.38874000	-3.48255900	0.12036400
H	1.02436800	-4.45847800	-1.20909100
H	2.13994600	-3.64973500	-0.11282600
C	0.01104500	2.58371600	-1.51851900
H	-0.82369600	2.17979700	-2.10189000

C	-0.53873100	3.63024700	-0.54608800
H	-1.21204800	3.18910100	0.19473900
H	0.26283000	4.14318300	-0.00931300
H	-1.09841400	4.38597700	-1.10025400
C	0.99909100	3.20879700	-2.52051300
H	0.49550300	3.99606100	-3.08503200
H	1.85130800	3.65082300	-1.99826900
H	1.37534900	2.46763700	-3.22941400
C	3.17959200	0.72643000	1.91497800
H	3.54232800	1.75719700	1.84915600
C	4.38358900	-0.20955600	2.01342300
H	4.08509500	-1.23498600	2.24669500
H	4.96780000	-0.21679400	1.09030900
H	5.03510900	0.12806600	2.82147100
C	2.28804000	0.62370400	3.17112700
H	1.46093000	1.33680400	3.14019000
H	1.87413200	-0.38388100	3.26792700
H	2.88865300	0.83397500	4.05820200
H	0.07262700	0.00015000	-2.32214600
H	2.76271000	-1.59829400	0.53635200
H	1.79704100	2.57945100	0.58153300

Int_A-OTf

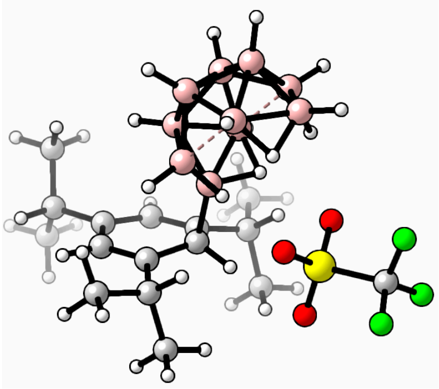

Charge: 0   Multiplicity: 1
Negative Frequency: None

B	-0.48149800	3.64393500	-0.86428400
B	-1.33478000	2.39809800	0.08201600
B	-0.39371500	3.66713400	0.90660000
B	0.94830100	4.31672800	-0.05611000
B	-0.39149000	1.97920100	-1.36935900
B	-0.43153800	0.93109900	0.05690500
B	-0.25322400	2.01623400	1.44455700
B	1.25662800	3.26477400	1.35270200
B	2.25810300	3.18211300	-0.10647400
B	1.11729800	3.22889300	-1.46129500
H	1.94927800	2.28576600	-1.05492000
H	-2.52057900	2.37129000	0.14053300
H	3.42503200	3.36387900	-0.16630700
H	-0.95900300	4.38552500	1.66392300
H	1.49192700	3.52670300	-2.54509800
H	1.73707200	3.58917800	2.38593000
H	-1.12035100	4.34287300	-1.58021500
H	-0.82548900	1.59005000	-2.39788400
H	1.15221700	5.48403100	-0.08118600

H	0.44690100	1.04254900	-0.94770700
H	2.04504700	2.31030200	0.88991500
H	-0.58332000	1.65721200	2.52152300
H	0.53946700	1.06562500	0.96977400
C	-3.66921000	-0.55728800	0.21503500
C	-3.03930200	-0.70525300	-1.03559100
C	-1.68007400	-0.84914200	-1.12143800
C	-0.88578300	-0.67713400	0.09668000
C	-1.57083500	-0.81376900	1.38191900
C	-2.93505100	-0.66704300	1.40822500
C	-0.94849500	-1.21070200	-2.38375300
H	0.00108000	-0.66567700	-2.38807400
C	-0.58719700	-2.70938200	-2.29935500
H	-0.04875600	-2.99454900	-3.20565800
H	0.06189400	-2.92188400	-1.44773700
H	-1.48952200	-3.32367700	-2.22788600
C	-1.71394700	-0.90132200	-3.66672000
H	-2.59693900	-1.53928000	-3.77037700
H	-2.03297300	0.14276200	-3.70860600
H	-1.07103300	-1.09265500	-4.52797800
C	-0.73449600	-1.14453300	2.58527500
H	0.21499700	-0.60768700	2.49046500
C	-1.38286600	-0.79477200	3.92122000
H	-1.69278100	0.25205100	3.96132300
H	-2.25582400	-1.42365500	4.12030500
H	-0.66709000	-0.96603700	4.72746200
C	-0.38939800	-2.64802500	2.51000300
H	0.22365600	-2.91230300	3.37421400
H	-1.29724600	-3.25777900	2.53306400
H	0.18291600	-2.88721600	1.61167100
C	-5.15737500	-0.33230600	0.27870200
H	-5.44210700	-0.31479700	1.33443000
C	-5.93404100	-1.46601800	-0.40418000
H	-5.74403900	-1.49353200	-1.48018600
H	-5.67072200	-2.43937000	0.01622600
H	-7.00547200	-1.30995000	-0.26003400
C	-5.50332100	1.03541100	-0.33047600
H	-4.96189600	1.84163600	0.16890000
H	-5.25560900	1.06733500	-1.39494500
H	-6.57462500	1.22155700	-0.22522500
H	0.05907500	-1.22469300	0.06426000
H	-3.64594100	-0.73447000	-1.93126400
H	-3.46996500	-0.66729000	2.34968900
S	2.65549300	-0.82794700	-0.05462900
O	2.41005900	-0.05889700	1.17547800
O	2.16614100	-2.20532500	-0.01893900

O	2.30016500	-0.08355900	-1.27316100
C	4.50768800	-0.97812000	-0.13600700
F	4.87309700	-1.64502200	-1.22814700
F	5.06587200	0.23090300	-0.17164000
F	4.96921900	-1.62577900	0.93103100

Int_A-H_2O

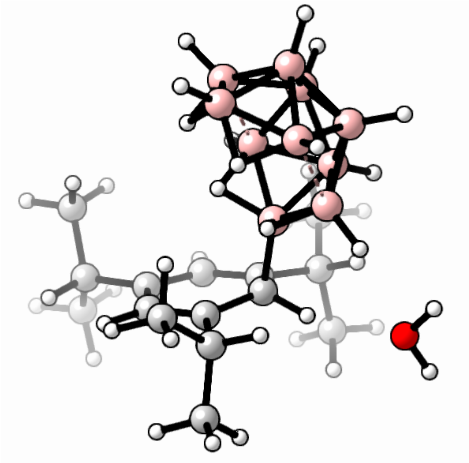

Charge: 1 Multiplicity: 1
Negative Frequency: None

B	-3.81552800	0.69076000	-0.59938600
B	-2.14794900	1.29631600	-0.74744500
B	-2.92136500	0.46151100	-2.11699800
B	-4.18416900	-0.69426100	-1.64837800
B	-2.69809700	0.42985200	0.71481100
B	-1.05609700	0.23452300	0.05212600
B	-1.26810800	0.07035300	-1.71312300
B	-2.61268800	-1.25394400	-2.29481600
B	-3.48190200	-2.14351500	-1.01600300
B	-4.04439600	-0.89000600	0.12353200
H	-3.18639700	-1.88737400	0.26282200
H	-1.93458400	2.45960300	-0.79400000
H	-3.85268500	-3.26486900	-1.06978600
H	-3.03386200	1.05311900	-3.13793400
H	-4.91561100	-1.13433400	0.88622500
H	-2.41801300	-1.77642900	-3.33884000
H	-4.67303500	1.47275300	-0.35780200
H	-2.80658900	0.86915600	1.80894100
H	-5.20485200	-0.73568100	-2.24505600
H	-1.82388300	-0.55262000	0.81590800
H	-2.19784100	-2.12826700	-1.39357900
H	-0.34959900	0.26371600	-2.43248800
H	-0.82579500	-0.77733200	-0.79750400
C	2.39017300	-0.89282300	-0.36720200
C	2.17136700	0.45361900	-0.70252700
C	1.21567600	1.19748800	-0.05801300
C	0.33767900	0.53397100	0.90252700
C	0.71708200	-0.79726700	1.37747700
C	1.68047600	-1.49069400	0.69282700
C	1.04364100	2.67480900	-0.27843000
H	0.07265400	2.95688800	0.13389000
C	2.11558500	3.39406500	0.56286400
H	3.11991800	3.16405600	0.19712800
H	1.96363000	4.47329200	0.49342000
H	2.05569900	3.10800600	1.61565500

C	1.10084300	3.09680800	-1.74648700
H	2.08399700	2.91244900	-2.18753800
H	0.34924800	2.57376400	-2.34101500
H	0.90575700	4.16847100	-1.82223100
C	0.01268200	-1.35881200	2.58550400
H	-0.83087100	-0.69975900	2.81599700
C	-0.52119200	-2.77780900	2.37087900
H	-1.18452600	-2.83826500	1.50275900
H	0.28845500	-3.49660800	2.22427400
H	-1.08889600	-3.09338300	3.24821500
C	0.97585200	-1.29302600	3.78403500
H	1.33927900	-0.27615900	3.94841000
H	0.45616500	-1.62025700	4.68686900
H	1.83726400	-1.94716100	3.62707800
C	3.38747300	-1.71022800	-1.14294300
H	3.50589700	-2.66522500	-0.62357700
C	4.76297600	-1.03816900	-1.22754500
H	4.73276300	-0.12216900	-1.82267100
H	5.15073600	-0.79403300	-0.23603600
H	5.46699500	-1.71948700	-1.70944100
C	2.81155300	-1.99695900	-2.54115400
H	1.84183700	-2.49704000	-2.48084900
H	2.68669800	-1.07204300	-3.11082200
H	3.49677300	-2.64537000	-3.09094000
H	-0.03513700	1.20914500	1.68498400
H	2.78490200	0.91207900	-1.46772300
H	1.93392800	-2.50492900	0.97896600
O	-0.91748900	2.88154200	2.59768700
H	-1.84557800	3.09519800	2.47971600
H	-0.58462900	3.52698400	3.22460400

Int_B

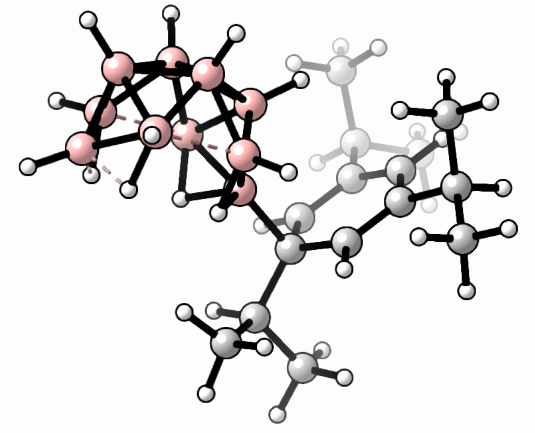

Charge: 1 Multiplicity: 1
Negative Frequency: None

B	2.74908400	0.42130300	-1.79504100
B	1.24045300	0.86815600	-0.96197200
B	2.68636600	1.89739500	-0.80969700
B	4.22126900	1.02863500	-1.00971300
B	1.84407600	-0.81242300	-0.96280400
B	1.02113100	-0.08118100	0.45099200
B	1.74153800	1.55063700	0.61356000
B	3.71105400	1.64411800	0.58954500
B	4.57992300	0.09919900	0.40634300
B	3.81206700	-0.71135000	-0.98287300

H	3.86478000	-1.03139300	0.29528700
H	0.28749400	1.22994900	-1.56771500
H	5.61210800	-0.18825400	0.90523800
H	2.60868500	3.02502600	-1.16659800
H	4.37938200	-1.58573600	-1.54279500
H	4.20405900	2.52730800	1.20334700
H	2.72476800	0.31747000	-2.97551900
H	1.34808800	-1.75043500	-1.48680100
H	5.09342500	1.49784900	-1.65627800
H	1.84695400	-1.11651700	0.33462800
H	3.79173500	0.58911900	1.37662700
H	1.16890600	2.36803200	1.24969500
H	1.76891000	0.46825200	1.40411200
C	-2.31234800	0.30744500	-0.57855900
C	-1.80469500	-0.98497700	-0.78514000
C	-0.90111100	-1.46222800	0.13389500
C	-0.38821800	-0.64716800	1.21797800
C	-1.04143000	0.63599600	1.39026700
C	-1.95855300	1.13357200	0.49697000
H	-0.50309400	-2.46418200	0.02664500
C	-2.51817000	2.53289300	0.63482600
H	-2.21590000	2.89967000	1.62093000
H	-0.74606200	1.23617300	2.24573400
H	-3.02900000	0.68615600	-1.30332500
C	-0.03665900	-1.36986400	2.55433900
H	0.38353100	-0.60116700	3.21262400
C	-4.04726800	2.56075800	0.56919200
H	-4.49184100	1.89589300	1.31359400
H	-4.40706200	3.57387100	0.76017900
H	-4.41641200	2.26721500	-0.41796700
C	-1.88519300	3.44527200	-0.42438500
H	-0.79463100	3.44285700	-0.35258100
H	-2.15826100	3.12153900	-1.43388200
H	-2.23631100	4.47136100	-0.29560100
C	-2.22805600	-1.75895200	-2.01715000
H	-3.30869300	-1.61357800	-2.12513000
C	-1.95902700	-3.25874700	-1.92115300
H	-2.38743100	-3.76358000	-2.78872900
H	-0.88616400	-3.47653800	-1.92080900
H	-2.40514500	-3.69730300	-1.02448000
C	-1.53952100	-1.15332500	-3.25079400
H	-1.77160400	-0.09159800	-3.36698200
H	-0.45304000	-1.25375400	-3.17300700
H	-1.86639800	-1.67101800	-4.15497900
C	-1.33598200	-1.88302700	3.18359600
H	-2.07446500	-1.08423400	3.28836800

H	-1.77526100	-2.67729900	2.57266700
H	-1.13857500	-2.29380300	4.17561400
C	0.98790800	-2.49588800	2.43113900
H	1.98320400	-2.13926700	2.15372300
H	1.09212900	-2.98930600	3.39915300
H	0.68232600	-3.26178800	1.71317800

Int_B-OTf

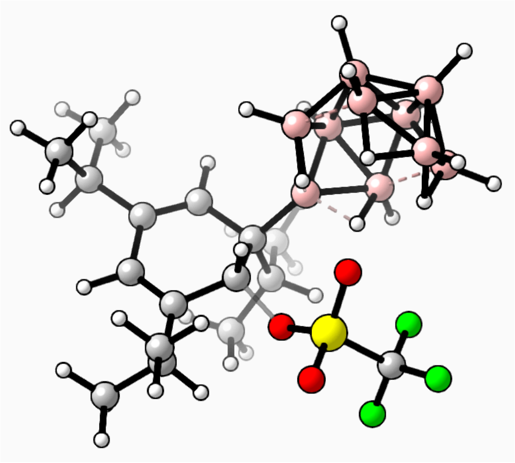

Charge: 0 Multiplicity: 1
Negative Frequency: None

B	-2.45421700	3.62234800	0.45274000
B	-0.72029900	3.24386500	0.44287200
B	-1.47791900	3.97478000	-0.98672200
B	-3.21098900	3.61910300	-1.15350300
B	-1.91481200	2.08768900	1.09203700
B	-0.45109200	1.54787600	0.21552200
B	-0.36486000	2.64495000	-1.20043500
B	-2.00729400	2.89302300	-2.25912900
B	-3.44445000	2.00106200	-1.71053200
B	-3.55730200	2.32754600	0.03237500
H	-3.31694200	1.21639100	-0.63419800
H	0.04002300	3.87369800	1.10085200
H	-4.29282400	1.53771900	-2.39247100
H	-1.07367100	5.02615000	-1.36100100
H	-4.58451500	2.14174200	0.59377300
H	-1.87272600	3.13615300	-3.41132100
H	-2.86354600	4.37774500	1.27183600
H	-2.05684400	1.77501900	2.22507100
H	-3.97319100	4.47468300	-1.45663400
H	-1.64933000	0.97959100	0.44544000
H	-2.26972600	1.60149800	-2.20741600
H	0.67084300	2.73832000	-1.76921300
H	-0.63800400	1.37572000	-1.11771600
C	3.18325300	-0.68012000	-0.24723000
C	3.17071600	0.75165000	0.08163900
C	2.04166600	1.31416000	0.51900700
C	0.73580200	0.56115700	0.68933900
C	0.76186200	-0.66538700	-0.25695800
C	2.07423700	-1.41293400	-0.35599400
H	2.03102000	2.35254200	0.82598000
C	2.05314000	-2.87177200	-0.75073600
H	1.23014000	-3.34987700	-0.21141200
H	0.48876800	-0.35620700	-1.27208400
H	4.15224600	-1.14662500	-0.39391700

C	0.54712900	0.15806400	2.19931100
H	-0.49512900	-0.15524500	2.31452500
C	3.33501000	-3.61607400	-0.38104000
H	3.57176200	-3.49863900	0.67940900
H	3.21553400	-4.68207000	-0.58996300
H	4.19056000	-3.26410100	-0.96513100
C	1.76907100	-3.00414600	-2.25632500
H	0.85233100	-2.49086900	-2.55575100
H	2.59765400	-2.57899600	-2.83067000
H	1.65734600	-4.05552000	-2.53288600
C	4.47709300	1.49664500	-0.09202200
H	5.25058300	0.88865100	0.39697300
C	4.49779000	2.88204200	0.54702800
H	4.25549900	2.83866900	1.61213000
H	5.49052200	3.32707300	0.44279500
H	3.78354800	3.55423500	0.06101700
C	4.83153300	1.58378400	-1.58419600
H	4.85318900	0.59731200	-2.05488300
H	4.08980200	2.19196200	-2.11139500
H	5.81312600	2.04540000	-1.72164800
C	1.43074100	-1.00592800	2.66000300
H	2.48961100	-0.77239800	2.52156900
H	1.26322000	-1.18045000	3.72667600
H	1.21246400	-1.93671800	2.13435300
C	0.78525800	1.34270800	3.14386000
H	0.46874500	1.06991600	4.15370700
H	1.84877400	1.59434300	3.18604800
H	0.23862900	2.24054600	2.85607200
S	-1.56093800	-1.94364500	-0.67212000
O	-0.29323500	-1.60477000	0.19704900
O	-1.44301600	-3.22234800	-1.31232400
O	-1.97854000	-0.77925400	-1.42316900
C	-2.74188900	-2.15445700	0.76209000
F	-3.90638000	-2.54568200	0.27593600
F	-2.27637500	-3.05920100	1.60106800
F	-2.89218400	-0.99796600	1.39079300

Int_B-H_2O

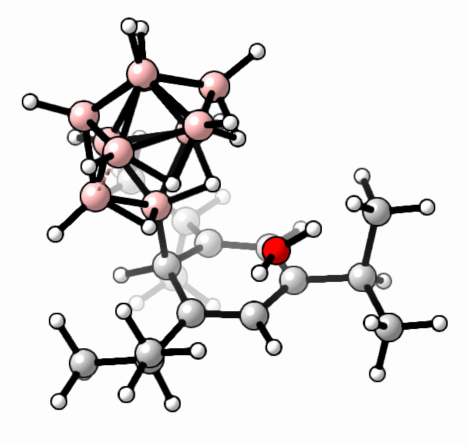

Charge: 1 Multiplicity: 1
Negative Frequency: None

B	3.06960400	0.87325600	-1.65753400
B	2.12671600	1.72945800	-0.41310800
B	3.67138400	0.95886000	0.01034500
B	4.11320800	-0.41094200	-1.02435600
B	1.34309800	0.62298000	-1.58559800
B	0.79957000	0.76179700	0.10908600
B	2.30261100	0.76705200	1.07331100
B	3.60491000	-0.66370900	0.67484200
B	3.17790700	-1.80675100	-0.61845700
B	2.65084200	-0.79701600	-1.98358000
H	1.99309800	-1.65133400	-1.22761900
H	2.04461600	2.90704100	-0.42536100
H	3.45640500	-2.95466500	-0.66150500
H	4.53191800	1.64343300	0.45254200
H	2.61896100	-1.25298500	-3.07591400
H	4.28734400	-1.01948700	1.57482600
H	3.42157200	1.48855500	-2.60784800
H	0.59061600	0.92238100	-2.44808000
H	5.22384400	-0.52604000	-1.41610800
H	0.67184800	-0.21523900	-0.79569000
H	2.64723200	-1.56295700	0.58737600
H	2.27141900	1.16787100	2.18492300
H	1.33269200	-0.12172000	0.96660900
C	-2.13413500	0.32294000	-1.18127000
C	-2.16463100	-0.92989900	-0.53637600
C	-1.72073800	-1.03890400	0.79048000
C	-1.11171400	0.01628200	1.42470100
C	-0.74402500	1.19325700	0.61843100
C	-1.52498800	1.40649400	-0.61059100
H	-1.89979200	-1.95531100	1.33629600
C	-1.75150200	2.78418500	-1.17383300
H	-2.10747600	2.65471600	-2.19995200
H	-0.59008200	2.10176700	1.19942700
H	-2.64519000	0.43285700	-2.13184500
C	-0.83438700	-0.06235500	2.91243700
H	-1.80351300	-0.38808900	3.31307200
C	-0.55004800	3.72495300	-1.19758500
H	0.24412200	3.35144100	-1.84288100
H	-0.87117600	4.69459200	-1.58307900
H	-0.13518000	3.89091900	-0.20012700
C	-2.90380800	3.39928500	-0.34956100

H	-3.78943200	2.76045300	-0.35141700
H	-2.59795000	3.56638800	0.68748000
H	-3.17580900	4.36541500	-0.77960300
C	-2.70743300	-2.10668100	-1.29917700
H	-3.59736100	-1.75333800	-1.83265000
C	-3.08938200	-3.31101700	-0.44424900
H	-3.81406000	-3.04772900	0.33000500
H	-3.54260100	-4.07593900	-1.07742900
H	-2.21052600	-3.75325200	0.03337700
C	-1.65562100	-2.49009900	-2.36228500
H	-1.43817400	-1.66124100	-3.03903800
H	-0.72253200	-2.79358600	-1.88104900
H	-2.02581100	-3.32882500	-2.95481700
C	0.17152100	-1.16148200	3.29652700
H	1.19262100	-0.89237200	3.01959300
H	0.15410300	-1.29909500	4.37902800
H	-0.08338800	-2.11477300	2.83156300
C	-0.49846900	1.27245900	3.57580500
H	-0.43467900	1.12930800	4.65592700
H	0.46228900	1.67053600	3.24347200
H	-1.27090000	2.02266500	3.38950000
O	0.61873800	-3.16115100	0.44711300
H	0.95902600	-3.53841900	1.26221700
H	0.71891300	-3.86617200	-0.19759400

47

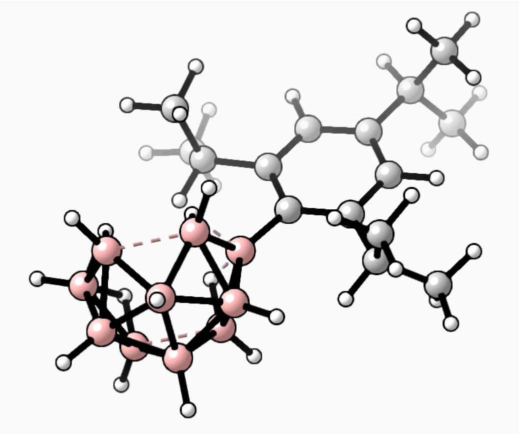

Charge: 0 Multiplicity: 1
Negative Frequency: None

B	3.91754900	1.35115900	-0.75411100
H	4.40684800	2.26960600	-1.32502500
B	4.08409600	-0.19284100	-1.56025400
H	4.53287300	-0.32248800	-2.64939100
B	4.99413800	0.11727000	-0.05664800
H	6.16380200	0.30265600	-0.00996000
B	4.37223500	-1.46810200	-0.34428800
H	4.94792200	-2.48767200	-0.51853800
B	4.04999100	-0.68532200	1.22773200
H	4.47219800	-1.17975900	2.21854600
B	3.89597700	1.04191900	0.99377100
H	4.36436600	1.70819800	1.85720600
B	2.35368600	0.32292600	1.38026900
H	1.84206600	0.36590300	2.44913600
H	1.79203600	-0.63691100	0.73900800
B	1.28638200	0.33732500	-0.06950000

C	-0.27387100	0.07950100	-0.09664000
C	-1.22053500	1.12632700	-0.11679900
C	-2.57705600	0.82213900	-0.07208000
C	-3.04108000	-0.48813200	-0.01916600
C	-2.10375400	-1.51111000	-0.03327400
C	-0.73494500	-1.25394400	-0.08056100
C	0.21823800	-2.44615500	-0.07508800
C	0.41919100	-2.97499700	1.35145800
H	-0.52691100	-3.35484400	1.74779200
H	0.76680200	-2.19127000	2.03043600
H	1.14714000	-3.79140800	1.36505500
C	-0.20990300	-3.57320100	-1.02090100
H	-1.11572100	-4.07488200	-0.67140000
H	-0.39843500	-3.19343300	-2.02787400
H	0.57815400	-4.32885100	-1.07960300
H	1.19563000	-2.11876100	-0.44816900
H	-2.45590300	-2.53735000	-0.00749200
C	-4.52517600	-0.79249500	0.03735600
C	-5.24569200	-0.30593600	-1.22535500
H	-5.19138800	0.78354700	-1.31291700
H	-4.79960600	-0.73809100	-2.12473800
H	-6.30238800	-0.58689900	-1.19656300
C	-5.17063700	-0.21041300	1.30005000
H	-5.11810900	0.88263600	1.30024500
H	-4.66887100	-0.57126900	2.20151100
H	-6.22549200	-0.49390400	1.35686200
H	-4.62948600	-1.88233300	0.08168900
H	-3.29760200	1.63249200	-0.07968000
C	-0.79116600	2.58483400	-0.13536100
H	0.17923700	2.63685800	-0.63032000
C	-0.61749200	3.09910100	1.29941500
H	0.08314600	2.47881200	1.86327400
H	-0.23824300	4.12526500	1.29918000
H	-1.57813200	3.08800500	1.82459200
C	-1.72943300	3.49970400	-0.92750400
H	-2.69050800	3.64151100	-0.42473200
H	-1.27348700	4.48771500	-1.03387900
H	-1.92257800	3.10434900	-1.92815600
B	2.43381800	1.63398900	0.17932700
H	2.08987000	2.74715700	0.38149700
B	2.38653200	0.81911300	-1.40739000
H	1.91319600	1.24487200	-2.40772600
H	1.79051000	-0.29906600	-1.17272800
H	3.44669500	-1.68346000	0.58801900
H	3.46820900	-1.34546100	-1.31300200

Triflate

Charge: -1 Multiplicity: 1
Negative Frequency: None

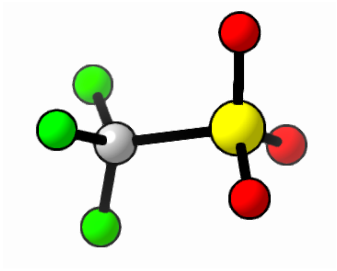

C	-0.94962100	0.00022500	0.00000300
S	0.92019100	-0.00023400	-0.00000200
O	1.24388500	-0.70938300	-1.23864100
O	1.24389300	-0.71790700	1.23371800
O	1.24375700	1.42734700	0.00492000
F	-1.43966100	0.62737600	-1.07764200
F	-1.44041300	-1.24716000	-0.00425700
F	-1.43966000	0.61999900	1.08190200

Triflic Acid

Charge: 0 Multiplicity: 1
Negative Frequency: None

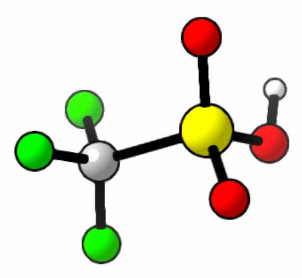

C	-1.00523600	0.00480900	-0.00313200
F	-1.42093300	-0.08512900	-1.25231800
F	-1.36905600	1.17568200	0.50181300
F	-1.53151300	-0.97030700	0.71574100
S	0.85829300	-0.14218700	0.06532200
O	1.24470400	0.04374300	1.43644300
O	1.22587700	-1.28624900	-0.70903700
O	1.25607000	1.14799400	-0.78918500
H	1.37904100	1.92001900	-0.21924400

Water

Charge: 0 Multiplicity: 1
Negative Frequency: None

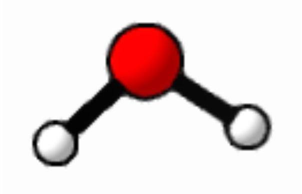

O	0.00000000	0.11665100	0.00000000
H	0.76029600	-0.46660200	0.00000000
H	-0.76029600	-0.46660200	0.00000000
