# Supplementary material for: Arene C–H borylation strategy enabled by a non-classical boron cluster-based electrophile
Source: Nat Commun. 2023 Mar 25;14:1671. doi: 10.1038/s41467-023-37258-6 (PMC10039867; doi:10.1038/s41467-023-37258-6)
Supplement: Supplementary file 5 — Supplementary Data 2 [file 41467_2023_37258_MOESM5_ESM.pdf]

Supplementary Data File 2: Crystallographic Data

Crystallographic Data

Compound 47 (spok226): CCDC 2189086

Crystal data

$C_{15}H_{36}B_{10}$	$F(000) = 704$
$M_r = 324.54$	$D_x = 1.013$ Mg m^{-3}
Monoclinic, $P2_1/n$	Mo *K*a radiation, l = 0.71073 Å
a = 11.7286 (6) Å	Cell parameters from 9505 reflections
b = 15.7765 (7) Å	q = 3.1–26.8°
c = 12.6392 (6) Å	m = 0.05 mm^{-1}
b = 114.5337 (15)°	*T* = 100 K
V = 2127.56 (18) Å^3	Block, colourless
Z = 4	0.18 × 0.16 × 0.13 mm

Data collection

Bruker SMART APEX2 area detector diffractometer	4357 independent reflections
Radiation source: microfocus sealed X-ray tube, Incoatec Ims	3875 reflections with *I* > 2s(*I*)
Mirror optics monochromator	$R_{int} = 0.048$
Detector resolution: 7.9 pixels mm^{-1}	$q_{max} = 26.4°$, $q_{min} = 2.6°$
w and f scans	*h* = -14Æ14
Absorption correction: multi-scan *SADABS2016*/2 (Bruker,2016/2) was used for absorption correction. wR2(int) was 0.1465 before and 0.0598 after correction. The Ratio of minimum to maximum transmission is 0.9258. The l/2 correction factor is Not present.	*k* = -19Æ19
$T_{min} = 0.690$, $T_{max} = 0.745$	*l* = -15Æ15
60134 measured reflections	

Refinement

Refinement on F^2	Primary atom site location: dual
Least-squares matrix: full	Hydrogen site location: mixed
$R[F^2 > 2s(F^2)] = 0.038$	H atoms treated by a mixture of independent and constrained refinement
$wR(F^2) = 0.097$	$w = 1/[s^2(F_o^2) + (0.0411P)^2 + 0.7258P]$ where $P = (F_o^2 + 2F_c^2)/3$
S = 1.04	$(D/s)_{max} < 0.001$
4357 reflections	$D\rho_{max} = 0.32$ e Å^{-3}

271 parameters	$\Delta\rho_{min}$ = -0.17 e Å^{-3}
0 restraints	

Special details

Geometry. All esds (except the esd in the dihedral angle between two l.s. planes) are estimated using the full covariance matrix. The cell esds are taken into account individually in the estimation of esds in distances, angles and torsion angles; correlations between esds in cell parameters are only used when they are defined by crystal symmetry. An approximate (isotropic) treatment of cell esds is used for estimating esds involving l.s. planes.

Fractional atomic coordinates and isotropic or equivalent isotropic displacement parameters (Å^2) for (spok226)

	x	*y*	*z*	U_{iso}*/U_{eq}
C1	0.74286 (9)	0.48543 (6)	0.48631 (9)	0.0161 (2)
C2	0.83814 (9)	0.52462 (6)	0.58461 (9)	0.0167 (2)
C3	0.85098 (9)	0.61235 (6)	0.58763 (9)	0.0177 (2)
H3A	0.915839	0.637735	0.653217	0.021*
C4	0.77215 (9)	0.66440 (6)	0.49794 (9)	0.0173 (2)
C5	0.67619 (9)	0.62580 (6)	0.40492 (9)	0.0186 (2)
H5	0.620141	0.660275	0.343836	0.022*
C6	0.65889 (9)	0.53791 (6)	0.39773 (9)	0.0172 (2)
C7	0.92956 (9)	0.47362 (6)	0.68800 (9)	0.0181 (2)
H7C	0.892191	0.416112	0.684187	0.022*
C8	0.95067 (10)	0.51224 (7)	0.80592 (9)	0.0226 (2)
H8C	0.997492	0.565384	0.816954	0.034*
H8D	0.998501	0.472467	0.868131	0.034*
H8E	0.869517	0.523570	0.808097	0.034*
C9	1.05524 (10)	0.46150 (7)	0.67925 (10)	0.0249 (2)
H9B	1.040753	0.434254	0.605073	0.037*
H9C	1.110247	0.425686	0.743545	0.037*
H9D	1.094938	0.516817	0.683561	0.037*
C10	0.79002 (10)	0.76008 (6)	0.50805 (9)	0.0188 (2)
H10C	0.882205	0.771498	0.544335	0.023*
C11	0.73523 (13)	0.79609 (7)	0.58885 (11)	0.0329 (3)
H11A	0.645318	0.783618	0.557209	0.049*
H11B	0.747928	0.857595	0.595340	0.049*
H11C	0.777208	0.770197	0.665949	0.049*
C12	0.73482 (13)	0.80625 (7)	0.39187 (10)	0.0298 (3)
H12A	0.766674	0.780699	0.338785	0.045*

H12B	0.758988	0.866142	0.403758	0.045*
H12C	0.643242	0.801633	0.358246	0.045*
C13	0.54513 (9)	0.50378 (7)	0.29434 (9)	0.0190 (2)
H13	0.548098	0.440527	0.299942	0.023*
C14	0.42378 (10)	0.53262 (7)	0.30087 (11)	0.0273 (3)
H14A	0.422178	0.512254	0.373489	0.041*
H14B	0.351831	0.509421	0.234613	0.041*
H14C	0.419532	0.594669	0.298684	0.041*
C15	0.54642 (11)	0.52754 (8)	0.17757 (10)	0.0298 (3)
H15A	0.539297	0.589196	0.167572	0.045*
H15B	0.475686	0.500233	0.114476	0.045*
H15C	0.625119	0.508417	0.175817	0.045*
B1	0.61984 (11)	0.21757 (7)	0.42412 (10)	0.0181 (2)
H1	0.5309 (12)	0.1891 (8)	0.3678 (11)	0.027*
B2	0.67215 (11)	0.30742 (7)	0.37173 (10)	0.0165 (2)
H2	0.6234 (11)	0.3240 (8)	0.2814 (11)	0.025*
B3	0.76356 (11)	0.21333 (7)	0.40853 (10)	0.0181 (2)
H3	0.7825 (12)	0.1820 (8)	0.3399 (11)	0.027*
B4	0.74251 (11)	0.14906 (8)	0.51537 (10)	0.0199 (2)
H4	0.7325 (12)	0.0811 (9)	0.5034 (11)	0.030*
B5	0.60752 (10)	0.31818 (7)	0.47733 (10)	0.0168 (2)
H5A	0.6905 (11)	0.3579 (8)	0.5557 (11)	0.025*
H5B	0.5187 (12)	0.3469 (8)	0.4659 (11)	0.025*
B6	0.73348 (10)	0.38541 (7)	0.47904 (10)	0.0163 (2)
B7	0.83783 (11)	0.31139 (7)	0.45264 (10)	0.0181 (2)
H7A	0.9046 (12)	0.3360 (8)	0.4233 (11)	0.027*
H7B	0.8465 (12)	0.3529 (8)	0.5387 (11)	0.027*
B8	0.88556 (11)	0.20576 (8)	0.54759 (10)	0.0198 (2)
H8A	0.9001 (12)	0.2449 (8)	0.6426 (11)	0.030*
H8B	0.9761 (12)	0.1787 (8)	0.5627 (11)	0.030*
B9	0.82027 (12)	0.19252 (8)	0.65205 (11)	0.0214 (2)
H9A	0.8618 (12)	0.1617 (8)	0.7350 (12)	0.032*
B10	0.65606 (11)	0.21367 (8)	0.57314 (10)	0.0199 (2)
H10A	0.7451 (12)	0.2512 (8)	0.6555 (11)	0.030*
H10B	0.5925 (12)	0.1930 (8)	0.6092 (11)	0.030*

Atomic displacement parameters (Å^2) for (spok226)

	U^{11}	U^{22}	U^{33}	U^{12}	U^{13}	U^{23}
C1	0.0146 (5)	0.0161 (5)	0.0190 (5)	-0.0003 (4)	0.0084 (4)	-0.0002 (4)
C2	0.0149 (5)	0.0183 (5)	0.0182 (5)	0.0003 (4)	0.0080 (4)	0.0003 (4)
C3	0.0151 (5)	0.0186 (5)	0.0188 (5)	-0.0023 (4)	0.0066 (4)	-0.0018 (4)
C4	0.0165 (5)	0.0169 (5)	0.0209 (5)	-0.0006 (4)	0.0103 (4)	0.0001 (4)
C5	0.0159 (5)	0.0183 (5)	0.0202 (5)	0.0019 (4)	0.0061 (4)	0.0024 (4)
C6	0.0151 (5)	0.0178 (5)	0.0193 (5)	-0.0005 (4)	0.0077 (4)	-0.0005 (4)
C7	0.0180 (5)	0.0154 (5)	0.0189 (5)	0.0002 (4)	0.0058 (4)	0.0003 (4)
C8	0.0236 (5)	0.0207 (5)	0.0197 (5)	0.0009 (4)	0.0053 (4)	-0.0003 (4)
C9	0.0196 (5)	0.0281 (6)	0.0260 (6)	0.0060 (4)	0.0084 (5)	0.0054 (4)
C10	0.0177 (5)	0.0158 (5)	0.0225 (5)	-0.0018 (4)	0.0079 (4)	0.0006 (4)
C11	0.0526 (8)	0.0213 (6)	0.0339 (7)	-0.0050 (5)	0.0270 (6)	-0.0065 (5)
C12	0.0466 (7)	0.0171 (5)	0.0266 (6)	-0.0012 (5)	0.0162 (5)	0.0019 (4)
C13	0.0164 (5)	0.0168 (5)	0.0202 (5)	0.0003 (4)	0.0041 (4)	0.0005 (4)
C14	0.0175 (5)	0.0283 (6)	0.0318 (6)	-0.0006 (4)	0.0058 (5)	-0.0043 (5)
C15	0.0297 (6)	0.0352 (7)	0.0213 (6)	-0.0091 (5)	0.0073 (5)	-0.0038 (5)
B1	0.0180 (5)	0.0159 (5)	0.0193 (6)	-0.0011 (4)	0.0066 (5)	-0.0013 (4)
B2	0.0169 (5)	0.0164 (5)	0.0158 (5)	0.0007 (4)	0.0064 (4)	-0.0003 (4)
B3	0.0181 (5)	0.0173 (5)	0.0177 (5)	0.0016 (4)	0.0062 (5)	-0.0014 (4)
B4	0.0203 (6)	0.0161 (6)	0.0212 (6)	0.0000 (4)	0.0065 (5)	0.0002 (4)
B5	0.0164 (5)	0.0166 (5)	0.0169 (5)	-0.0014 (4)	0.0064 (4)	-0.0006 (4)
B6	0.0143 (5)	0.0184 (6)	0.0162 (5)	0.0002 (4)	0.0062 (4)	0.0004 (4)
B7	0.0170 (5)	0.0182 (5)	0.0195 (6)	0.0014 (4)	0.0082 (5)	-0.0004 (4)
B8	0.0184 (6)	0.0186 (6)	0.0197 (6)	0.0014 (4)	0.0053 (5)	-0.0005 (4)
B9	0.0232 (6)	0.0191 (6)	0.0199 (6)	0.0014 (5)	0.0067 (5)	0.0028 (5)
B10	0.0207 (6)	0.0184 (6)	0.0208 (6)	-0.0016 (4)	0.0087 (5)	0.0015 (4)

Geometric parameters (Å, º) for (spok226)

C1—C2	1.4218 (14)	B1—H1	1.086 (13)
C1—C6	1.4119 (14)	B1—B2	1.7779 (16)
C1—B6	1.5818 (15)	B1—B3	1.7777 (17)
C2—C3	1.3912 (14)	B1—B4	1.7861 (17)
C2—C7	1.5301 (14)	B1—B5	1.7531 (16)
C3—H3A	0.9500	B1—B10	1.7514 (17)
C3—C4	1.3942 (14)	B2—H2	1.076 (12)
C4—C5	1.3860 (14)	B2—B3	1.7761 (16)
C4—C10	1.5221 (14)	B2—B5	1.7959 (16)

C5—H5	0.9500	B2—B6	1.7495 (16)
C5—C6	1.3988 (14)	B2—B7	1.7820 (16)
C6—C13	1.5264 (14)	B3—H3	1.097 (13)
C7—H7C	1.0000	B3—B4	1.7859 (17)
C7—C8	1.5321 (14)	B3—B7	1.7492 (16)
C7—C9	1.5352 (14)	B3—B8	1.7500 (16)
C8—H8C	0.9800	B4—H4	1.082 (14)
C8—H8D	0.9800	B4—B8	1.7927 (17)
C8—H8E	0.9800	B4—B9	1.7256 (17)
C9—H9B	0.9800	B4—B10	1.7930 (17)
C9—H9C	0.9800	B5—H5A	1.232 (12)
C9—H9D	0.9800	B5—H5B	1.089 (12)
C10—H10C	1.0000	B5—B6	1.8115 (16)
C10—C11	1.5243 (15)	B5—B10	1.9840 (17)
C10—C12	1.5220 (15)	B6—H5A	1.337 (12)
C11—H11A	0.9800	B6—B7	1.8199 (16)
C11—H11B	0.9800	B6—H7B	1.327 (12)
C11—H11C	0.9800	B7—H7A	1.069 (13)
C12—H12A	0.9800	B7—H7B	1.237 (12)
C12—H12B	0.9800	B7—B8	1.9932 (17)
C12—H12C	0.9800	B8—H8A	1.297 (13)
C13—H13	1.0000	B8—H8B	1.085 (13)
C13—C14	1.5287 (15)	B8—B9	1.7925 (17)
C13—C15	1.5289 (15)	B9—H8A	1.291 (13)
C14—H14A	0.9800	B9—H9A	1.072 (13)
C14—H14B	0.9800	B9—B10	1.7944 (17)
C14—H14C	0.9800	B9—H10A	1.291 (13)
C15—H15A	0.9800	B10—H10A	1.273 (13)
C15—H15B	0.9800	B10—H10B	1.073 (13)
C15—H15C	0.9800		
C2—C1—B6	119.67 (9)	B7—B3—B4	117.91 (8)
C6—C1—C2	118.31 (9)	B7—B3—B8	69.45 (7)
C6—C1—B6	122.02 (9)	B8—B3—B1	107.99 (8)
C1—C2—C7	122.38 (9)	B8—B3—B2	118.23 (8)
C3—C2—C1	119.57 (9)	B8—B3—H3	114.4 (7)
C3—C2—C7	118.05 (9)	B8—B3—B4	60.92 (7)

C2—C3—H3A	118.8	B1—B4—H4	119.8 (7)
C2—C3—C4	122.41 (9)	B1—B4—B8	105.76 (8)
C4—C3—H3A	118.8	B1—B4—B10	58.59 (6)
C3—C4—C10	119.61 (9)	B3—B4—B1	59.69 (7)
C5—C4—C3	117.50 (9)	B3—B4—H4	119.8 (7)
C5—C4—C10	122.82 (9)	B3—B4—B8	58.55 (7)
C4—C5—H5	118.8	B3—B4—B10	105.32 (8)
C4—C5—C6	122.37 (9)	B8—B4—H4	124.3 (7)
C6—C5—H5	118.8	B8—B4—B10	104.95 (8)
C1—C6—C13	123.09 (9)	B9—B4—B1	110.81 (8)
C5—C6—C1	119.68 (9)	B9—B4—B3	110.48 (8)
C5—C6—C13	117.19 (9)	B9—B4—H4	121.0 (7)
C2—C7—H7C	107.4	B9—B4—B8	61.22 (7)
C2—C7—C8	113.24 (8)	B9—B4—B10	61.29 (7)
C2—C7—C9	110.93 (8)	B10—B4—H4	125.0 (7)
C8—C7—H7C	107.4	B1—B5—B2	60.11 (6)
C8—C7—C9	110.33 (9)	B1—B5—H5A	127.2 (6)
C9—C7—H7C	107.4	B1—B5—H5B	123.6 (7)
C7—C8—H8C	109.5	B1—B5—B6	109.75 (8)
C7—C8—H8D	109.5	B1—B5—B10	55.48 (6)
C7—C8—H8E	109.5	B2—B5—H5A	102.1 (6)
H8C—C8—H8D	109.5	B2—B5—H5B	128.1 (6)
H8C—C8—H8E	109.5	B2—B5—B6	58.02 (6)
H8D—C8—H8E	109.5	B2—B5—B10	105.80 (7)
C7—C9—H9B	109.5	H5A—B5—H5B	106.7 (9)
C7—C9—H9C	109.5	B6—B5—H5A	47.6 (6)
C7—C9—H9D	109.5	B6—B5—H5B	119.2 (7)
H9B—C9—H9C	109.5	B6—B5—B10	116.96 (8)
H9B—C9—H9D	109.5	B10—B5—H5A	90.1 (6)
H9C—C9—H9D	109.5	B10—B5—H5B	116.3 (7)
C4—C10—H10C	107.6	C1—B6—B2	137.98 (9)
C4—C10—C11	110.13 (9)	C1—B6—B5	128.43 (8)
C4—C10—C12	113.89 (9)	C1—B6—H5A	108.7 (5)
C11—C10—H10C	107.6	C1—B6—B7	127.94 (8)
C12—C10—H10C	107.6	C1—B6—H7B	108.9 (6)
C12—C10—C11	109.83 (9)	B2—B6—B5	60.54 (6)
C10—C11—H11A	109.5	B2—B6—H5A	100.1 (5)

C10—C11—H11B	109.5	B2—B6—B7	59.86 (6)
C10—C11—H11C	109.5	B2—B6—H7B	99.3 (5)
H11A—C11—H11B	109.5	B5—B6—H5A	42.9 (5)
H11A—C11—H11C	109.5	B5—B6—B7	103.26 (8)
H11B—C11—H11C	109.5	B5—B6—H7B	113.4 (5)
C10—C12—H12A	109.5	H5A—B6—H7B	92.9 (8)
C10—C12—H12B	109.5	B7—B6—H5A	114.2 (5)
C10—C12—H12C	109.5	B7—B6—H7B	42.8 (5)
H12A—C12—H12B	109.5	B2—B7—B6	58.11 (6)
H12A—C12—H12C	109.5	B2—B7—H7A	126.5 (7)
H12B—C12—H12C	109.5	B2—B7—H7B	101.3 (6)
C6—C13—H13	107.4	B2—B7—B8	106.43 (8)
C6—C13—C14	110.56 (9)	B3—B7—B2	60.39 (6)
C6—C13—C15	112.61 (9)	B3—B7—B6	110.00 (8)
C14—C13—H13	107.4	B3—B7—H7A	123.4 (7)
C14—C13—C15	111.21 (9)	B3—B7—H7B	126.6 (6)
C15—C13—H13	107.4	B3—B7—B8	55.29 (6)
C13—C14—H14A	109.5	B6—B7—H7A	118.4 (7)
C13—C14—H14B	109.5	B6—B7—H7B	46.8 (6)
C13—C14—H14C	109.5	B6—B7—B8	117.92 (8)
H14A—C14—H14B	109.5	H7A—B7—H7B	108.0 (9)
H14A—C14—H14C	109.5	B8—B7—H7A	116.7 (7)
H14B—C14—H14C	109.5	B8—B7—H7B	90.7 (6)
C13—C15—H15A	109.5	B3—B8—B4	60.53 (7)
C13—C15—H15B	109.5	B3—B8—B7	55.26 (6)
C13—C15—H15C	109.5	B3—B8—H8A	129.5 (6)
H15A—C15—H15B	109.5	B3—B8—H8B	122.4 (7)
H15A—C15—H15C	109.5	B3—B8—B9	109.05 (8)
H15B—C15—H15C	109.5	B4—B8—B7	106.14 (8)
B2—B1—H1	118.1 (7)	B4—B8—H8A	101.5 (6)
B2—B1—B4	114.39 (8)	B4—B8—H8B	126.7 (7)
B3—B1—H1	127.4 (7)	B7—B8—H8A	93.2 (6)
B3—B1—B2	59.94 (6)	B7—B8—H8B	116.4 (7)
B3—B1—B4	60.15 (7)	H8A—B8—H8B	106.3 (9)
B4—B1—H1	118.0 (7)	B9—B8—B4	57.54 (7)
B5—B1—H1	114.6 (7)	B9—B8—B7	116.22 (8)
B5—B1—B2	61.14 (6)	B9—B8—H8A	46.0 (6)

B5—B1—B3	108.04 (8)	B9—B8—H8B	120.7 (7)
B5—B1—B4	118.21 (8)	B4—B9—B8	61.23 (7)
B10—B1—H1	115.4 (7)	B4—B9—H8A	105.3 (6)
B10—B1—B2	117.48 (8)	B4—B9—H9A	129.5 (7)
B10—B1—B3	107.47 (8)	B4—B9—B10	61.21 (7)
B10—B1—B4	60.90 (7)	B4—B9—H10A	103.5 (6)
B10—B1—B5	68.96 (7)	B8—B9—H8A	46.3 (6)
B1—B2—H2	118.4 (7)	B8—B9—H9A	127.9 (7)
B1—B2—B5	58.75 (6)	B8—B9—B10	104.90 (8)
B1—B2—B7	106.08 (8)	B8—B9—H10A	116.6 (6)
B3—B2—B1	60.02 (7)	H8A—B9—H9A	109.2 (9)
B3—B2—H2	118.6 (7)	H8A—B9—H10A	94.4 (8)
B3—B2—B5	106.23 (8)	H9A—B9—H10A	109.3 (9)
B3—B2—B7	58.89 (6)	B10—B9—H8A	118.8 (6)
B5—B2—H2	124.3 (7)	B10—B9—H9A	125.1 (7)
B6—B2—B1	111.49 (8)	B10—B9—H10A	45.2 (6)
B6—B2—H2	121.2 (7)	B1—B10—B4	60.51 (7)
B6—B2—B3	112.06 (8)	B1—B10—B5	55.56 (6)
B6—B2—B5	61.44 (6)	B1—B10—B9	109.23 (8)
B6—B2—B7	62.03 (6)	B1—B10—H10A	127.8 (6)
B7—B2—H2	124.9 (7)	B1—B10—H10B	124.5 (7)
B7—B2—B5	105.45 (8)	B4—B10—B5	106.94 (8)
B1—B3—H3	128.1 (7)	B4—B10—B9	57.50 (7)
B1—B3—B4	60.16 (7)	B4—B10—H10A	100.8 (6)
B2—B3—B1	60.04 (6)	B4—B10—H10B	127.6 (7)
B2—B3—H3	117.8 (7)	B5—B10—H10A	92.4 (6)
B2—B3—B4	114.49 (8)	B5—B10—H10B	115.9 (7)
B4—B3—H3	118.6 (7)	B9—B10—B5	117.61 (8)
B7—B3—B1	107.52 (8)	B9—B10—H10A	46.0 (6)
B7—B3—B2	60.72 (6)	B9—B10—H10B	118.8 (7)
B7—B3—H3	114.4 (7)	H10A—B10—H10B	105.8 (9)
C1—C2—C3—C4	0.99 (15)	B3—B4—B10—B5	7.08 (10)
C1—C2—C7—C8	-136.46 (10)	B3—B4—B10—B9	-105.28 (9)
C1—C2—C7—C9	98.85 (11)	B3—B8—B9—B4	33.23 (8)
C1—C6—C13—C14	112.37 (11)	B3—B8—B9—B10	-11.15 (11)
C1—C6—C13—C15	-122.55 (11)	B4—B1—B2—B3	26.63 (8)

C1—B6—B7—B2	-129.54 (12)	B4—B1—B2—B5	-110.05 (9)
C1—B6—B7—B3	-161.77 (10)	B4—B1—B2—B6	-77.19 (10)
C1—B6—B7—B8	137.75 (10)	B4—B1—B2—B7	-11.46 (11)
C2—C1—C6—C5	4.17 (14)	B4—B1—B3—B2	-151.92 (8)
C2—C1—C6—C13	-173.61 (9)	B4—B1—B3—B7	-112.63 (9)
C2—C1—B6—B2	-157.27 (11)	B4—B1—B3—B8	-39.16 (8)
C2—C1—B6—B5	116.02 (11)	B4—B1—B5—B2	103.85 (10)
C2—C1—B6—B7	-72.20 (13)	B4—B1—B5—B6	72.65 (11)
C2—C3—C4—C5	1.79 (15)	B4—B1—B5—B10	-37.09 (8)
C2—C3—C4—C10	178.84 (9)	B4—B1—B10—B5	142.54 (8)
C3—C2—C7—C8	44.35 (12)	B4—B1—B10—B9	31.68 (8)
C3—C2—C7—C9	-80.34 (11)	B4—B3—B7—B2	-103.93 (10)
C3—C4—C5—C6	-1.57 (15)	B4—B3—B7—B6	-72.54 (11)
C3—C4—C10—C11	-77.72 (12)	B4—B3—B7—B8	38.19 (8)
C3—C4—C10—C12	158.39 (10)	B4—B3—B8—B7	-141.31 (8)
C4—C5—C6—C1	-1.43 (15)	B4—B3—B8—B9	-32.08 (8)
C4—C5—C6—C13	176.48 (9)	B4—B8—B9—B10	-44.38 (8)
C5—C4—C10—C11	99.17 (12)	B4—B9—B10—B1	-32.82 (8)
C5—C4—C10—C12	-24.72 (14)	B4—B9—B10—B5	-93.25 (9)
C5—C6—C13—C14	-65.47 (12)	B5—B1—B2—B3	136.68 (8)
C5—C6—C13—C15	59.62 (12)	B5—B1—B2—B6	32.86 (8)
C6—C1—C2—C3	-3.96 (14)	B5—B1—B2—B7	98.59 (8)
C6—C1—C2—C7	176.86 (9)	B5—B1—B3—B2	-39.19 (8)
C6—C1—B6—B2	22.84 (17)	B5—B1—B3—B4	112.73 (9)
C6—C1—B6—B5	-63.87 (14)	B5—B1—B3—B7	0.10 (11)
C6—C1—B6—B7	107.91 (12)	B5—B1—B3—B8	73.57 (10)
C7—C2—C3—C4	-179.79 (9)	B5—B1—B4—B3	-95.58 (10)
C10—C4—C5—C6	-178.52 (9)	B5—B1—B4—B8	-58.05 (11)
B1—B2—B3—B4	-26.66 (8)	B5—B1—B4—B9	6.62 (13)
B1—B2—B3—B7	-136.19 (8)	B5—B1—B4—B10	40.10 (9)
B1—B2—B3—B8	-95.46 (10)	B5—B1—B10—B4	-142.54 (8)
B1—B2—B5—B6	144.91 (8)	B5—B1—B10—B9	-110.86 (9)
B1—B2—B5—B10	32.66 (7)	B5—B2—B3—B1	37.65 (7)
B1—B2—B6—C1	-147.95 (11)	B5—B2—B3—B4	10.99 (11)
B1—B2—B6—B5	-31.88 (8)	B5—B2—B3—B7	-98.53 (8)
B1—B2—B6—B7	97.36 (9)	B5—B2—B3—B8	-57.80 (11)
B1—B2—B7—B3	38.62 (8)	B5—B2—B6—C1	-116.07 (13)

B1—B2—B7—B6	-106.18 (8)	B5—B2—B6—B7	129.24 (8)
B1—B2—B7—B8	6.87 (10)	B5—B2—B7—B3	99.90 (8)
B1—B3—B4—B8	-136.58 (8)	B5—B2—B7—B6	-44.89 (7)
B1—B3—B4—B9	-102.76 (9)	B5—B2—B7—B8	68.15 (9)
B1—B3—B4—B10	-38.19 (7)	B5—B6—B7—B2	43.86 (7)
B1—B3—B7—B2	-38.97 (8)	B5—B6—B7—B3	11.62 (10)
B1—B3—B7—B6	-7.58 (11)	B5—B6—B7—B8	-48.85 (10)
B1—B3—B7—B8	103.15 (9)	B6—C1—C2—C3	176.15 (9)
B1—B3—B8—B4	38.81 (8)	B6—C1—C2—C7	-3.03 (14)
B1—B3—B8—B7	-102.49 (9)	B6—C1—C6—C5	-175.94 (9)
B1—B3—B8—B9	6.73 (11)	B6—C1—C6—C13	6.28 (15)
B1—B4—B8—B3	-38.06 (8)	B6—B2—B3—B1	102.86 (9)
B1—B4—B8—B7	-5.73 (10)	B6—B2—B3—B4	76.20 (11)
B1—B4—B8—B9	105.43 (9)	B6—B2—B3—B7	-33.33 (8)
B1—B4—B9—B8	-97.06 (9)	B6—B2—B3—B8	7.41 (12)
B1—B4—B9—B10	32.47 (8)	B6—B2—B5—B1	-144.91 (8)
B1—B4—B10—B5	-31.62 (7)	B6—B2—B5—B10	-112.26 (8)
B1—B4—B10—B9	-143.99 (9)	B6—B2—B7—B3	144.79 (8)
B1—B5—B6—C1	161.84 (10)	B6—B2—B7—B8	113.04 (8)
B1—B5—B6—B2	31.97 (8)	B7—B2—B3—B1	136.19 (8)
B1—B5—B6—B7	-11.51 (10)	B7—B2—B3—B4	109.53 (9)
B2—B1—B3—B4	151.92 (8)	B7—B2—B3—B8	40.73 (9)
B2—B1—B3—B7	39.29 (8)	B7—B2—B5—B1	-99.70 (8)
B2—B1—B3—B8	112.76 (9)	B7—B2—B5—B6	45.21 (7)
B2—B1—B4—B3	-26.57 (8)	B7—B2—B5—B10	-67.04 (9)
B2—B1—B4—B8	10.96 (11)	B7—B2—B6—C1	114.69 (13)
B2—B1—B4—B9	75.63 (11)	B7—B2—B6—B5	-129.24 (8)
B2—B1—B4—B10	109.11 (9)	B7—B3—B4—B1	95.10 (9)
B2—B1—B5—B6	-31.20 (7)	B7—B3—B4—B8	-41.48 (9)
B2—B1—B5—B10	-140.94 (8)	B7—B3—B4—B9	-7.66 (12)
B2—B1—B10—B4	-104.07 (10)	B7—B3—B4—B10	56.91 (11)
B2—B1—B10—B5	38.47 (8)	B7—B3—B8—B4	141.31 (8)
B2—B1—B10—B9	-72.39 (11)	B7—B3—B8—B9	109.23 (9)
B2—B3—B4—B1	26.62 (8)	B7—B8—B9—B4	93.10 (9)
B2—B3—B4—B8	-109.96 (9)	B7—B8—B9—B10	48.71 (11)
B2—B3—B4—B9	-76.14 (11)	B8—B3—B4—B1	136.58 (8)
B2—B3—B4—B10	-11.56 (11)	B8—B3—B4—B9	33.82 (8)

B2—B3—B7—B6	31.39 (7)	B8—B3—B4—B10	98.40 (9)
B2—B3—B7—B8	142.12 (8)	B8—B3—B7—B2	-142.12 (8)
B2—B3—B8—B4	103.87 (10)	B8—B3—B7—B6	-110.73 (9)
B2—B3—B8—B7	-37.43 (8)	B8—B4—B9—B10	129.53 (8)
B2—B3—B8—B9	71.79 (11)	B8—B4—B10—B1	99.58 (9)
B2—B5—B6—C1	129.87 (12)	B8—B4—B10—B5	67.96 (9)
B2—B5—B6—B7	-43.49 (7)	B8—B4—B10—B9	-44.41 (8)
B2—B6—B7—B3	-32.23 (8)	B8—B9—B10—B1	11.58 (11)
B2—B6—B7—B8	-92.71 (9)	B8—B9—B10—B4	44.40 (8)
B3—B1—B2—B5	-136.68 (8)	B8—B9—B10—B5	-48.85 (11)
B3—B1—B2—B6	-103.82 (9)	B9—B4—B8—B3	-143.49 (9)
B3—B1—B2—B7	-38.09 (7)	B9—B4—B8—B7	-111.16 (8)
B3—B1—B4—B8	37.53 (8)	B9—B4—B10—B1	143.99 (9)
B3—B1—B4—B9	102.20 (9)	B9—B4—B10—B5	112.36 (9)
B3—B1—B4—B10	135.69 (8)	B10—B1—B2—B3	95.16 (10)
B3—B1—B5—B2	38.64 (7)	B10—B1—B2—B5	-41.52 (9)
B3—B1—B5—B6	7.44 (11)	B10—B1—B2—B6	-8.67 (12)
B3—B1—B5—B10	-102.29 (9)	B10—B1—B2—B7	57.07 (11)
B3—B1—B10—B4	-39.43 (8)	B10—B1—B3—B2	-112.13 (9)
B3—B1—B10—B5	103.11 (8)	B10—B1—B3—B4	39.78 (8)
B3—B1—B10—B9	-7.75 (11)	B10—B1—B3—B7	-72.84 (10)
B3—B2—B5—B1	-38.24 (7)	B10—B1—B3—B8	0.62 (11)
B3—B2—B5—B6	106.67 (8)	B10—B1—B4—B3	-135.69 (8)
B3—B2—B5—B10	-5.59 (10)	B10—B1—B4—B8	-98.15 (9)
B3—B2—B6—C1	146.87 (11)	B10—B1—B4—B9	-33.48 (8)
B3—B2—B6—B5	-97.06 (9)	B10—B1—B5—B2	140.94 (8)
B3—B2—B6—B7	32.18 (8)	B10—B1—B5—B6	109.74 (8)
B3—B2—B7—B6	-144.79 (8)	B10—B4—B8—B3	-99.05 (9)
B3—B2—B7—B8	-31.75 (7)	B10—B4—B8—B7	-66.72 (9)
B3—B4—B8—B7	32.33 (7)	B10—B4—B8—B9	44.44 (8)
B3—B4—B8—B9	143.49 (9)	B10—B4—B9—B8	-129.53 (8)
B3—B4—B9—B8	-32.80 (8)	B10—B5—B6—C1	-137.69 (10)
B3—B4—B9—B10	96.72 (9)	B10—B5—B6—B2	92.44 (9)
B3—B4—B10—B1	38.71 (8)	B10—B5—B6—B7	48.95 (10)
